# Association of Early Introduction of Solids With Infant Sleep: A Secondary Analysis of a Randomized Clinical Trial

**DOI:** 10.1001/jamapediatrics.2018.0739

**Published:** 2018-07-09

**Authors:** Michael R. Perkin, Henry T. Bahnson, Kirsty Logan, Tom Marrs, Suzana Radulovic, Joanna Craven, Carsten Flohr, Gideon Lack

**Affiliations:** 1 The Population Health Research Institute, St George's, University of London, London, England; 2 The Immune Tolerance Network, Benaroya Research Institute, Seattle, Washington; 3 The Paediatric Allergy Research Group, the Department of Women and Children’s Health, King’s College London, London, England; 4 The Unit for Population-Based Dermatology Research, St John’s Institute of Dermatology School of Basic and Medical Biosciences, Faculty of Life Sciences & Medicine, King's College London, London, England

## Abstract

**Importance:**

The World Health Organization recommends exclusive breastfeeding for 6 months. However, 75% of British mothers introduce solids before 5 months and 26% report infant waking at night as influencing this decision.

**Objective:**

To determine whether early introduction of solids influences infant sleep.

**Design, Setting, and Participants:**

The Enquiring About Tolerance study was a population-based randomized clinical trial conducted from January 15, 2008, to August 31, 2015, that included 1303 exclusively breastfed 3-month-old infants from England and Wales. Clinical visits took place at St Thomas’ Hospital, London, England, and the trial studied the early introduction of solids into the infant diet from age 3 months.

**Interventions:**

The early introduction group (EIG) continued to breastfeed while nonallergenic and then 6 allergenic foods were introduced. The standard introduction group (SIG) followed British infant feeding guidelines (ie, exclusive breastfeeding to around age 6 months and to avoid any food consumption during this period).

**Main Outcomes and Measures:**

Secondary analysis of an a priori secondary outcome of the effect of early food introduction on infant sleep using the standardized Brief Infant Sleep Questionnaire.

**Results:**

Of the 1303 infants who were enrolled in the Enquiring About Tolerance study, 1225 participants (94%) completed the final 3-year questionnaire (618 SIG [95%] and 607 EIG [93%]). Randomization was effective and there were no significant baseline differences between the 2 groups. Following the early introduction of solids, infants in the EIG slept significantly longer and woke significantly less frequently than infants in the SIG. Differences between the 2 groups peaked at age 6 months. At this point, in the intention-to-treat analysis infants in the EIG slept for 16.6 (95% CI, 7.8-25.4) minutes longer per night and their night waking frequency had decreased from 2.01 to 1.74 wakings per night. Most clinically important, very serious sleep problems, which were significantly associated with maternal quality of life, were reported significantly more frequently in the SIG than in the EIG (odds ratio, 1.8; 95% CI, 1.22-2.61).

**Conclusions and Relevance:**

In a randomized clinical trial, the early introduction of solids into the infant’s diet was associated with longer sleep duration, less frequent waking at night, and a reduction in reported very serious sleep problems.

**Trial Registration:**

isrctn.org Identifier: ISRCTN14254740

## Key Points

**Question:**

Does the early consumption of solids before 6 months of age influence infant sleep?

**Findings:**

In this randomized clinical trial that included 1303 three-month-old infants randomized to early solids introduction vs exclusive breastfeeding, early solids introduction significantly increased sleep duration and reduced nighttime wakings and the reporting of very serious sleep problems.

**Meaning:**

The early introduction of solids resulted in small but significant improvements in infant sleep characteristics.

## Teaser

This randomized clinical trial explores the effect of the early introduction of solid foods vs exclusive breastfeeding on sleep characteristics among 3-month-old infants.

## Introduction

The British government currently advises all mothers to breastfeed exclusively for around the first 6 months of life.^1^ However, the proportion of mothers who achieve 6 months of exclusive breastfeeding is low at around 1% in the last Infant Feeding Survey undertaken in 2010, and three-quarters of mothers (75%) had chosen to introduce solids by the time their baby was 5 months old.^2^

The UK Department of Health advises that infants be introduced to solids when they are ready. They also state that it is normal for infants to wake up in the night and that this is not necessarily a sign of hunger or an indication that solid foods should be introduced.^3^ If infants seem hungrier at any time before age 6 months, it is recommended that additional milk feeds are given.^3^

However, in the 2010 Infant Feeding Survey, the most common reason, given by 52% of respondents, for introducing solids before 6 months was the perception that their baby was no longer satisfied with milk feeds. Twenty-nine percent started introducing solids because they felt their infant was developmentally mature enough (able to sit up and hold food in his or her hand), but a similar proportion of mothers (26%) mentioned their baby waking up in the night as a motivation to begin introducing solids.^2^

It is a commonly held belief that introducing solids early will help babies sleep better.^4,5,6^ However, the choices website of the UK National Health Service states “Starting solid foods won’t make your baby any more likely to sleep through the night”^3^ and the National Childbirth Trust leaflet “Introduction to Solid Foods” states “There is evidence that introducing solids does not affect the length of time babies sleep.”^7^ The latter references the Davis Area Research on Lactation, Infant Nutrition, and Growth study, which was a small, nonrandomized study that assessed sleep (at ages 9 and 18 months), development, and nutrient intake in matched cohorts of 60 breastfed and 45 formula-fed infants.^8^ Comparing the breastfed infants who had been introduced to solids before or after age 6 months, total sleeping time (day and night) at age 9 months was the same in those who had been introduced solids before and after age 6 months, and the age at solid food introduction was not related to the frequency of night feeds.^8^

The Enquiring About Tolerance (EAT) study is a large randomized clinical trial of 1303 infants that examined the effects of the early introduction of 6 allergenic foods from age 3 months as compared with infants encouraged to exclusively breastfeed until age 6 months and who were then introduced to allergenic foods at parental discretion.^9,10^ Infants who were introduced to the 6 allergenic foods were allowed to consume nonallergenic foods as well. The study's primary hypothesis was that early introduction would prevent the development of food allergies to these 6 foods. As part of the study, a detailed validated sleep questionnaire was completed on 15 occasions between ages 3 months and 3 years. Therefore, the EAT cohort provides a unique opportunity to undertake a secondary analysis of sleep data to more definitively answer the question as to whether introducing solids results in improved sleep in infants.

## Method

### Participants

**Figure 1.**
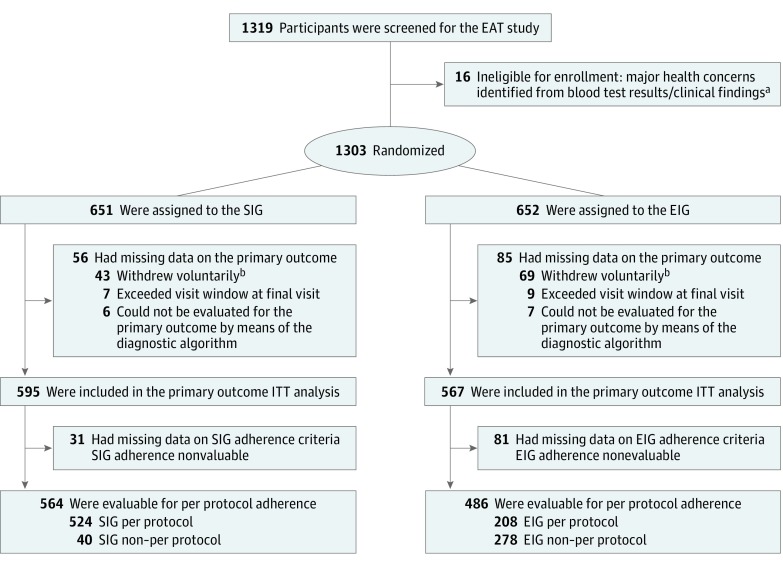
Enquiring About Tolerance (EAT) Enrollment and Randomization Baseline visits occurred when participants were age 3 months. ITT indicates intention-to-treat. The primary outcome for the EAT study was challenge-proven food allergy to 1 or more of the 6 early-introduction foods between age 1 and 3 years. ^a^Eight infants randomized to each group were found to have significant health issues either on blood test results or the clinical examination at the enrollment visit, rendering them ineligible for enrollment; conditions included severe vitamin D deficiency, severe iron deficiency, severe failure to thrive, familial hypercholesterolemia, congenital stridor, epidermolysis bullosa, and cartilage hair hypoplasia syndrome. ^b^Forty-three participants in the standard introduction group (SIG) and 69 participants in the early introduction group (EIG) withdrew voluntarily from the study. Reasons given were as follows: concerns about the blood tests (SIG, 0; EIG, 2), emigration (SIG, 10; EIG, 12), expenses (SIG, 1; EIG, 1), family health issues (SIG, 3; EIG, 0), family issues (SIG, 2; EIG, 4), no reason given (SIG, 11; EIG, 16), lost contact with family (SIG, 15; EIG, 28), too far to travel for study assessments (SIG, 0; EIG, 1) and unhappy participating in the study (SIG, 1; EIG, 5).

A total of 1303 three-month-old infants were recruited from the general population in England and Wales through direct advertising and enrolled between November 2, 2009, and July 30, 2012. Ethical approval for the EAT study was provided by St Thomas’ Hospital Research Ethics Committee and informed consent was obtained from the parents of all children enrolled in the study. The Consolidated Standards of Reporting Trials flow chart for the primary outcome of the EAT study is shown in Figure 1. Full methods, the trial protocol, and the statistical analysis plan (original documents and final versions, with a summary of all changes made to both documents) and other Consolidated Standards of Reporting Trials information for the primary outcome of the EAT study are published elsewhere.^9,10^ The oirignal trial protocol, final protocol, summary of changes to EAT protocol, original SAP, final SAP, and summary of changes to SAP can be found in Supplements 2-7. All children were healthy, exclusively breastfed, and born at term (≥37 weeks’ gestation).

### Procedures

Participants were randomized by simple randomization to the standard or early introduction group. The standard introduction group (SIG) was asked to exclusively breastfeed to around 6 months of age. The early introduction group (EIG) was encouraged to continue breastfeeding but also to introduce nonallergenic foods for the first week and then, while continuing these, to introduce 6 allergenic foods to their infant: cow’s milk, peanut, hen’s egg, sesame, white fish, and wheat. Full details are given in the eMethods in Supplement 1.

All families were sent an online questionnaire each month up to 1 year and then every 3 months until the child reached age 3 years. This recorded the frequency of food consumption (allergenic and nonallergenic) for both groups and included questions about breastfeeding frequency and duration.^9,10^ The online questionnaires included a standardized tool for assessing infant sleep, the Brief Infant Sleep Questionnaire (eTable 1 in Supplement 1), which asks about a child's sleep during the past week.^11^

The 3-month, 1-year, and 3-year interim questionnaires assessed maternal quality of life using the World Health Organization’s (WHO) Quality of Life–BREF instrument.^12^ This is a 26-item version of the WHO Quality of Life–100 and assesses 4 broad domains of quality of life: physical health, psychological health, social relationships, and environment. Domain scores are scaled in a positive direction (ie, higher scores denote a higher quality of life).

### Statistical Analyses

Analyses were undertaken using Stata, version 14 (Stata Corp), R, version 3.4.3 (R Foundation), SAS, version 9.4, and JMP, version 14 (SAS Institute). Longitudinal night time sleep (hours) was fit using linear mixed-effects models with a random intercept. Missing values were multiply imputed and modeled results were pooled across 100 imputed data sets. All model effects were linear except visit month, which was modeled using a flexible restricted cubic spline with 3 knots placed at the 0.10, 0.5, and 0.90 quantiles of the observed distribution of visit months, as recommended by Harrell.^13^ An average treatment effect was estimated over the entire study duration (eTable 2 in Supplement 1). In addition, the age of a peak treatment effect was identified in a second mixed-effects complete case model in which an interaction term was included for assessment month (categorical variable) and randomized treatment assignment. Data were assumed to be missing at random. The mixed-effects model is known to give unbiased estimates when covariates associated with missing data and the outcome are included—this formed the primary basis for covariate selection. The covariates included were race/ethnicity, number of siblings, enrollment eczema severity (assessed with the SCORing Atopic Dermatitis score), hours of sleep at baseline, age at enrollment, and where and how the infant sleeps (eTable 2 in Supplement 1). While the missing-at-random assumption is impossible to verify statistically, an analysis of missing data (eFigure 1 in Supplement 1) demonstrates that the intervention effect was consistent and stronger at the key assessments (12 months and 36 months) when the data were most complete (ie, 88% and 93% response rates, respectively). Furthermore, we asked a second statistician, who was not privy to the completed analyses, to independently conduct several modeling approaches to assess the robustness of our findings. The multiple imputation analysis results (eTable 2 in Supplement 1) and the sensitivity analyses (eTable 3 in Supplement 1) are detailed in the eMethods in Supplement 1. For the secondary outcomes, normal, binomial, and Poisson distributions were used to model sleep in hours, the presence of sleep problems, and the number of night wakings, respectively. A spatial power law correlation matrix was assumed for hours of nighttime sleep and number of night wakings. Last, a repeated-measures logistic regression model with an exchangeable correlation structure was fit after dichotomizing the presence or absence of participant reported sleep problems.

## Results

The randomization was effective and there were no baseline demographic differences between the 2 groups (eTable 4 in Supplement 1).^9,10^ Interim questionnaire noncompletion rates increased over the course of the study and were consistently higher in the EIG compared with the SIG. An analysis of missing data showed that the differential completion rate between the randomized groups did not introduce a bias that overestimates the duration of sleep in the EIG participants (eFigure 1 in Supplement 1).

**Figure 2.**
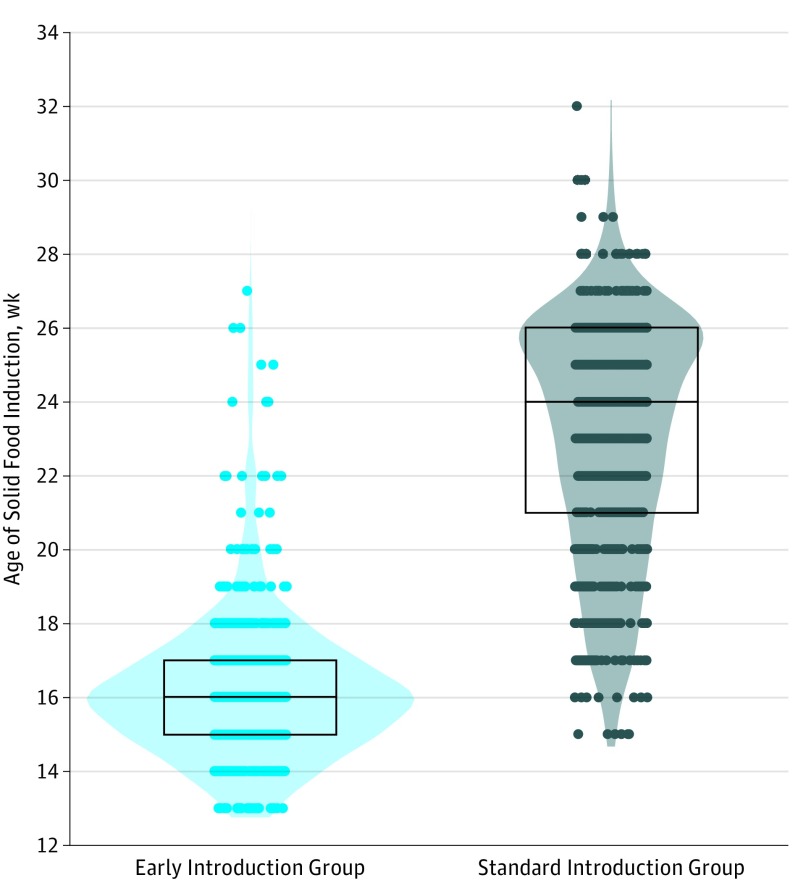
Age of Solid Food Introduction in Infants Participating in the Enquiring About Tolerance (EAT) Study The black bar indicates the median, the box upper hinge the 75th percentile, and the box lower hinge the 25th percentile.

The age at which solids were introduced in the EIG was significantly younger than in the SIG (mean [SD] age EIG, 16.2 [2.15] weeks; SIG, 23.1 [3.18] weeks) (Figure 2). However, while the introduction of allergenic foods before age 6 months in the SIG was minimal, most had introduced nonallergenic solids before this point, albeit significantly later than the EIG, and hence there was a broader distribution of age of solid food introduction within the SIG compared with the EIG (Figure 2). Shortly after age 6 months, infants in both groups were all consuming solids in essentially the same quantities, with no difference in any anthropometric parameter between the 2 groups when measured at age 1 year.^10^

### Nocturnal Sleep Characteristics: Intention-to-Treat Analysis

**Figure 3.**
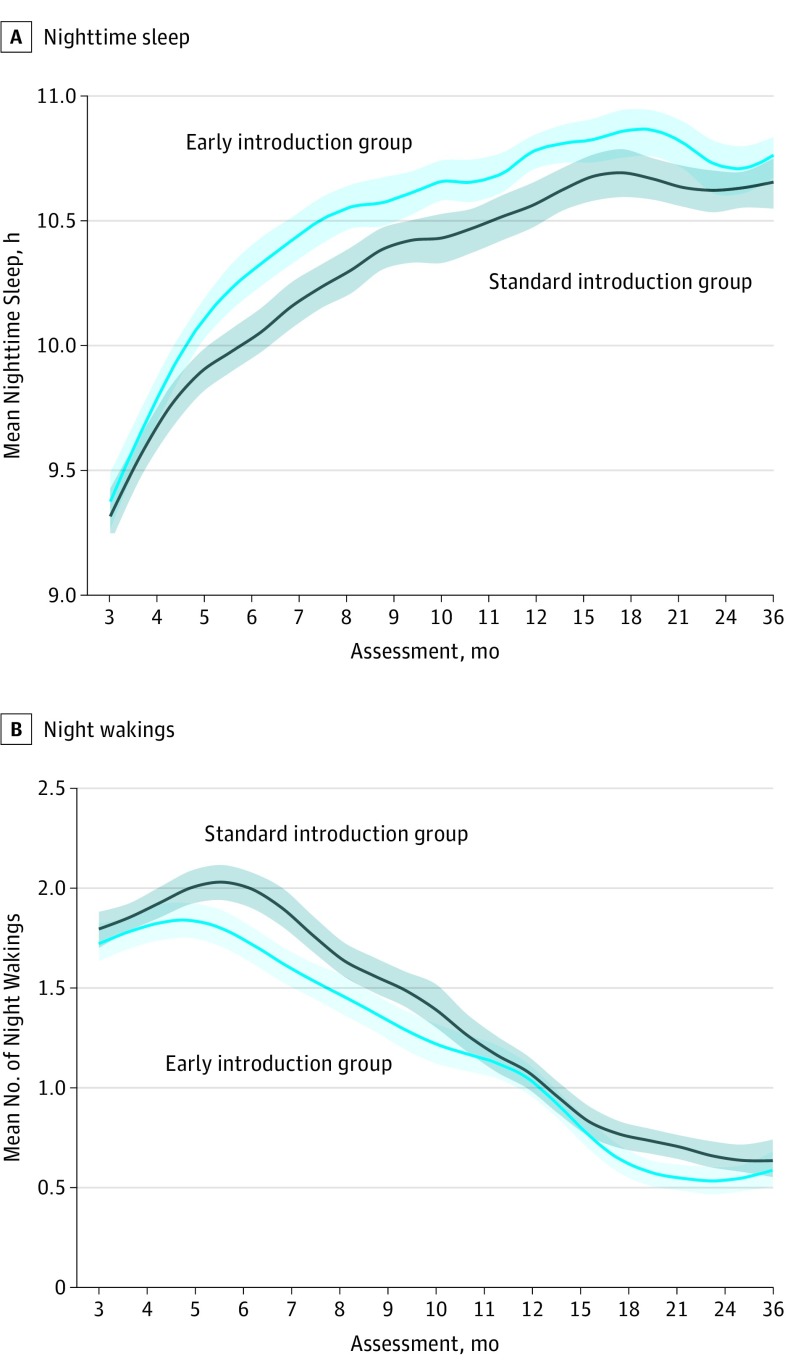
Nocturnal Sleep Characteristics by Study Group in the Intention-to-Treat Unadjusted Analysis A smoothed mean response across all the assessments using a cubic spline with lambda = 0.5. The mean response and confidence region for each randomized group were observed, unadjusted means (ie, they were not derived from the mixed-effects model). The confidence interval was produced by bootstrapping the marginal mean at each assessment for each treatment group.

In the unadjusted intention-to-treat comparison between the groups, infants in the EIG demonstrated a significantly longer duration of nocturnal sleep from age 5 months that persisted beyond age 1 year (Figure 3). The associations between covariates and nocturnal sleep duration and night waking frequency at enrollment are shown in eTables 5 and 6, respectively, and the eResults in Supplement 1. The association between enrollment SCORAD status and longitudinal nocturnal sleep characteristics is reported in eFigure 2 and discussed in the eResults in Supplement 1. Using the multivariable mixed-effects multiple imputation analysis model, it was estimated that infants in the EIG slept a mean of 7.3 minutes (95% CI, 2.0-12.5) more per night on average over the duration of the study, (eTable 2 in Supplement 1). In the multivariable mixed-effects complete case analysis model, the difference peaked at 16.6 (95% CI, 7.8-25.4) minutes more per night at age 6 months. There was no difference in the amount of daytime sleep between the 2 groups (data not shown).

A similar pattern was seen in the unadjusted analysis for the number of night wakings (Figure 3). In the multivariable mixed-effects complete case analysis model, the EIG group experienced a mean (SD) of 9.1% (95% CI, 4%-14%) fewer night wakings over the duration of the study when compared with the SIG group in the intention-to-treat (ITT) analyses (eFigure 3 in Supplement 1).

**Figure 4.**
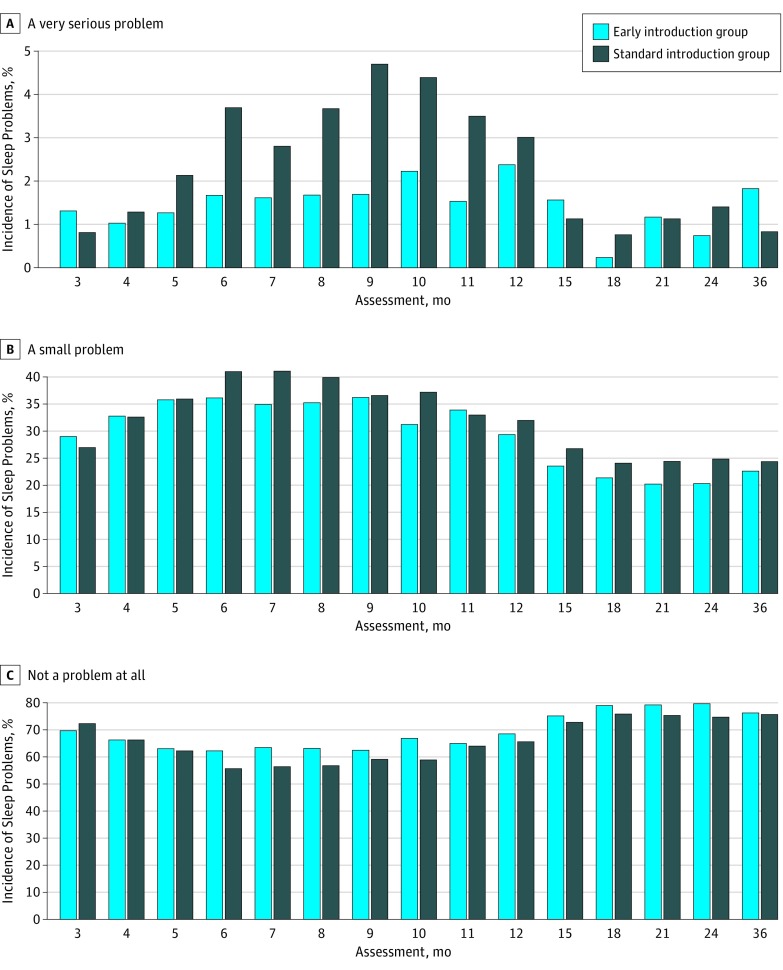
Parent Reporting of a Sleep Problem in Their Child by Study Group (Intention-to-Treat Analysis) In an intention-to-treat analysis, families were significantly more likely to report a sleep problem in their child in the standard introduction group compared with the early introduction group.

The differences observed in nocturnal sleep characteristics were not explained by the introduction of formula milk, which was minimal in both groups before age 6 months (see eResults in Supplement 1). Responses to the generic Brief Infant Sleep Questionnaire question of whether a family considered their child to have a sleep problem varied significantly by study group. In the ITT comparison, families in the SIG were significantly more likely than those in the EIG to report a small problem (odds ratio, 1.2; 95% CI, 1.05-1.41) or a very serious problem (odds ratio, 1.8; 95% CI, 1.22-2.61) with their child’s sleep (Figure 4). Parent's perceptions that their child had a sleep problem were significantly correlated with maternal global and sleep quality of life as well as all 4 quality of life domains (environmental, psychological, social, and physical) (eFigure 4 in Supplement 1). Moreover, parental perception of infant sleep problems correlated with the infant's night time sleep duration and night waking frequency (eFigure 4 in Supplement 1).

### Nocturnal Sleep Characteristics per Protocol Analysis

We have previously shown that families found it difficult to feed their EIG infants the per protocol amount of all 6 allergenic foods in the tight window that they were given to achieve per protocol adherence by age 6 months. The result was that full adherence to the EIG regimen was achieved by only 223 EIG participants (42%) in whom adherence was evaluable (eTable 7 in Supplement 1).^9^ These infants had significantly greater improvement in nocturnal sleep characteristics (eFigure 5 in Supplement 1) and reported sleep problems (eFigure 6 in Supplement 1). An association between degrees of adherence with the EIG recommended weekly dose of allergenic protein consumption and nighttime sleep characteristics was also apparent (eFigure 7 in Supplement 1). These per protocol findings are discussed in the eResults in Supplement 1.

## Discussion

Infants who were introduced to solids earlier slept longer at night and woke less frequently. Throughout the first year of life, families in the EIG reported fewer very serious sleep problems affecting their infant than in the SIG. While the effect of the early introduction of solids on sleep in individual infants is likely to vary, the significant difference in sleep characteristics between the EIG and SIG was apparent in an ITT analysis that should control for the presence of individual differences.

### Strengths and Limitations

The commonly held belief that introducing solids early will help infants sleep better could have produced a reporting bias. Mothers, anticipating improved infant sleep, could have reported better outcomes.^4,5,6^ This potential bias could have been circumvented by assessing infant sleep by undertaking actigraphy, and not using actigraphy could be seen as a limitation of our study. However, to undertake actigraphy through to age 3 years (to match the data provided from the Brief Infant Sleep Questionnaire questionnaire which itself has been validated against actigraphy) was not feasible logistically or financially. It would have also imposed an unacceptable burden on the families.

There are several reasons why we believe such a reporting bias is unlikely to explain these findings. Most importantly, the improvement in sleep was sustained throughout the first year of infancy and beyond. While it is plausible that mothers might overreport better sleep in the key early months (up to age 6 months), it seems very unlikely that beyond age 6 months, when both groups were consuming essentially identical quantities of solids, that this biased reporting would persist. Furthermore, the strong dose-response relationship seen not just in the key early months but through to 1 year and beyond, argues against this simply reflecting a bias.

That the early introduction of solids before age 6 months should result in sustained sleep differences lasting beyond age 1 year, when both groups of infants would be consuming essentially identical quantities of solids, should not be unexpected. The Barker hypothesis, which evolved into the International Society for Developmental Origins of Health and Disease, has a remit “to recognize the broader scope of developmental cues, extending from the oocyte to the infant and beyond, and the concept that the early life environment has widespread consequences for later health.”^14^ If fetal and infant nutrition can influence adult cardiovascular health, then it seems entirely plausible that sleep patterns established in early infancy can persist for several years.

Consistent with the Barker hypothesis, infant sleep is also associated with other health outcomes. Studies in childhood have shown graded inverse associations between sleep duration and levels of adiposity, with increased sleep duration associated with lower levels of obesity^15,16^ and a recent British study has shown a strong inverse graded association in children between sleep duration, adiposity, and diabetes risk markers.^17^ In the latter study of 4525 9- to 10-year-old children, a 1-hour greater sleep duration was associated with a 0.19 lower body mass index (calculated as weight in kilograms divided by height in meters squared) (95% CI, 0.09-0.28 kg/m^2^), 0.03 kg/m^5^ lower fat mass index (95% CI, 0.00-0.05 kg/m^5^), 2.9% lower homeostatic model assessment insulin resistance (95% CI, 1.2%-4.4%), and 0.24% lower fasting glucose level (95% CI, 0.03%-0.44%).^17^

Our results confirm that poor infant sleep is strongly associated with parental quality of life. At age 6 months, when the ITT differences were most significant between the 2 groups, EIG infants were sleeping 17 minutes longer per night, equating to 2 hours of extra sleep per week, and were waking 2 fewer times at night per week. Most significantly, at this point, EIG families were reporting half the rate of very serious sleep problems. It is unknown over what period sleep differences need to be sustained to result in the changes in type 2 diabetes risk markers observed in the British study,^17^ and while the differences observed in the study of British children were small at an individual level, at a population level they are likely to be of more significance.

Sleep patterns at enrollment were associated with the likelihood of EIG participants adhering to the early introduction intervention. Infants who subsequently were adherent to the EIG regimen were sleeping significantly longer and had less night wakings at enrollment. It seems likely that infant maturity links the ability to consume solid food early and to have improved sleeping patterns. However, the ITT results indicate the improvement in sleep extends beyond differences in infant maturity at enrollment.

The heaviest infants at enrollment slept the longest at night. Conversely, while it has been claimed that poor sleep and night wakenings in infancy are not related to hunger, we found that those infants with the highest weight gain between birth and enrollment were most likely to be waking at night. This is consistent with the idea that their rapid weight gain was leading to an enhanced caloric and nutritional requirement, resulting in hunger and disrupted sleep.

Trials randomizing solid food introduction are scarce, and to our knowledge, no systematic reviews in this area exist. There has been 1 randomized clinical trial of feeding infants rice cereal in the bottle before bedtime at age 5 weeks or at age 4 months that did not show a statistically significant effect on sleep.^18^

The Avon Longitudinal Study of Parents and Children (ALSPAC) collected sleep data in their cohort at age 6, 18, and 30 months. Parents recorded the amount of day and night sleep and the number of night wakings. Total sleep in ALSPAC was very similar to that in the EAT SIG, but night wakings at 6 months were much more common in EAT. This may reflect the clear differences in breastfeeding rates at age 6 months: in ALSPAC 31.7% of mothers were still breastfeeding as compared with 97.5% in EAT.^19^ The differential questionnaire completion rate between study groups has the potential to bias the results, but a multiple imputation analysis suggests that bias due to missing data was unlikely to have influenced our results.

We have shown previously that the EAT cohort is representative of the population in England and Wales because the study population was drawn from a wide geographical area.^9^ We have also shown that within an ITT comparison, the early introduction of solids did not adversely influence the duration of breastfeeding in EIG mothers compared with SIG mothers.^9^ A further strength of our study is that all mothers reported sleep patterns contemporaneously. The combined strengths of the number of participants in the EAT study, the highly significant ITT effect, and the enhanced per protocol effect shown in this analysis of sleep data demonstrate the robustness of the results presented. Additionally, the clear dose response between food consumption and better sleep indicates the biological plausibility of these results.

## Conclusions

To our knowledge, we show for the first time in a randomized clinical trial setting that, consistent with the belief of many parents, the early introduction of solids does have a small but significant effect on sleep characteristics. With recent guidelines advocating introducing solids from age 4 to 6 months in some^20^ or all^21^ infants, our results suggest that improved sleep may be a concomitant benefit.

## Notes

Supplement 1. **eMethods.** Details about EAT introduction regimens, instructions about formula milk introduction and monitoring of food consumption. **eResults.** Results on age of introduction of solids, effect on breastfeeding, formula milk introduction rates, differential questionnaire completion, factors influencing nocturnal sleep characteristics, soothing method and location, longitudinal effects on quality of life and longitudinal effect of eczema and results of the per protocol analysis. **eDiscussion**. Further discussion about the influence of enrollment soothing method and sleep location on nocturnal sleep characteristics. **eTable 1. **Brief Infant Sleep Questionnaire (BISQ). **eTable 2. **Multivariable mixed-effects multiple imputation analysis model of night time sleep duration—model estimates summary table. **eTable 3. **Complete case sensitivity analyses of night time sleep duration: comparison of four different multivariable modelling strategies to asses the robustness of the primary analysis results. **eTable 4. **Baseline demographic data. **eTable 5. **Regression analysis of factors influencing duration of nocturnal sleep in the EAT study participants at enrollment (3 months of age: n=1209). **eTable 6. **Regression analysis of factors influencing frequency of night time waking in the EAT Study participants at enrollment (3 months of age: n=1211). **eTable 7. **Overall per protocol compliance criteria in the EAT study. **eFigure 1. **Examination of the proportion of missing sleep assessments and the corresponding mean night time sleep hours. **eFigure 2.** Association between enrollment SCORAD status and nocturnal sleep characteristics by study group (intention to treat analysis). **eFigure 3**. Multivariable mixed-effects complete case analysis model: adjusted differences in nocturnal sleep characteristics between the EIG and SIG in the ITT and PP analyses. **eFigure 4**. Correlation at three time points (3, 12 and 36 months) between infant and maternal sleep and maternal quality of life measures (X-axis) and parent reported infant/child sleep problems (Y-axis). **eFigure 5.** Nocturnal sleep characteristics by study group in the per protocol unadjusted analysis. **eFigure 6.** Parent reported sleep problems in their child by study group (per protocol analysis, p<0·0001). **eFigure 7.** Association between degrees of adherence with the EIG recommended weekly dose of allergenic protein consumption and night time sleep characteristics.

Supplement 2. **Trial Protocol.** Original protocol.

Supplement 3. **Trial Protocol.** Final protocol.

Supplement 4. **Trial Protocol.** Summary of changes to EAT protocol.

Supplement 5. **Trial Protocol.** Original SAP.

Supplement 6. **Trial Protocol.** Final SAP.

Supplement 7. **Trial Protocol.** Summary of changes to SAP.
