# Supplementary material for: Association of Early Introduction of Solids With Infant Sleep: A Secondary Analysis of a Randomized Clinical Trial
Source: JAMA Pediatr. 2018 Jul 9;172(8):e180739. doi: 10.1001/jamapediatrics.2018.0739 (PMC6142923; doi:10.1001/jamapediatrics.2018.0739)
Supplement: Supplement 1. — eMethods. Details about EAT introduction regimens, instructions about formula milk introduction and monitoring of food consumption. eResults. Results on age of introduction of solids, effect on breastfeeding, formula milk introduction rates, differential questionnaire completion, factors influencing nocturnal sleep characteristics, soothing method and location, longitudinal effects on quality of life and longitudinal effect of eczema and results of the per protocol analysis. eDiscussion. Further discussion about the influence of enrollment soothing method and sleep location on nocturnal sleep characteristics. eTable 1. Brief Infant Sleep Questionnaire (BISQ). eTable 2. Multivariable mixed-effects multiple imputation analysis model of night time sleep duration—model estimates summary table. eTable 3. Complete case sensitivity analyses of night time sleep duration: comparison of four different multivariable modelling strategies to asses the robustness of the primary analysis results. eTable 4. Baseline demographic data. eTable 5. Regression analysis of factors influencing duration of nocturnal sleep in the EAT study participants at enrollment (3 months of age: n=1209). eTable 6. Regression analysis of factors influencing frequency of night time waking in the EAT Study participants at enrollment (3 months of age: n=1211). eTable 7. Overall per protocol compliance criteria in the EAT study. eFigure 1. Examination of the proportion of missing sleep assessments and the corresponding mean night time sleep hours. eFigure 2. Association between enrollment SCORAD status and nocturnal sleep characteristics by study group (intention to treat analysis). eFigure 3. Multivariable mixed-effects complete case analysis model: adjusted differences in nocturnal sleep characteristics between the EIG and SIG in the ITT and PP analyses. eFigure 4. Correlation at three time points (3, 12 and 36 months) between infant and maternal sleep and maternal quality of life measures (X-axis) and par [file jamapediatr-172-e180739-s001.pdf]

Supplementary Online Content

Perkin MR, Bahnson HT, Logan K, et al. Association of early introduction of solids with infant sleep: a secondary analysis of a randomized clinical trial. *JAMA Pediatr*. Published online July 9, 2018. doi:10.1001/jamapediatrics.2018.0739

eMethods. Details about EAT introduction regimens, instructions about formula milk introduction and monitoring of food consumption
eResults. Results on age of introduction of solids, effect on breastfeeding, formula milk introduction rates, differential questionnaire completion, factors influencing nocturnal sleep characteristics, soothing method and location, longitudinal effects on quality of life and longitudinal effect of eczema and results of the per-protocol analysis
eDiscussion. Further discussion about the influence of enrollment soothing method and sleep location on nocturnal sleep characteristics
eTable 1. Brief Infant Sleep Questionnaire (BISQ)
eTable 2. Multivariable mixed-effects multiple imputation analysis model of night time sleep duration - model estimates summary table
eTable 3. Complete case sensitivity analyses of night time sleep duration: comparison of four different multivariable modelling strategies to assess the robustness of the primary analysis results
eTable 4. Baseline demographic data
eTable 5. Regression analysis of factors influencing duration of nocturnal sleep in the EAT study participants at enrollment (3 months of age: n=1209)
eTable 6. Regression analysis of factors influencing frequency of night time waking in the EAT study participants at enrollment (3 months of age: n=1211)
eTable 7. Overall per-protocol compliance criteria in the EAT study
eFigure 1. Examination of the proportion of missing sleep assessments and the corresponding mean night time sleep hours
eFigure 2. Association between enrollment SCORAD status and nocturnal sleep characteristics by study group (intention to treat analysis)
eFigure 3. Multivariable mixed-effects complete case analysis model: Adjusted differences in nocturnal sleep characteristics between the EIG and SIG in the ITT and PP analyses
eFigure 4. Correlation at three time points (3, 12 and 36 months) between infant and maternal sleep and maternal quality of life measures (X-axis) and parent reported infant/child sleep problems (Y-axis)
eFigure 5. Nocturnal sleep characteristics by study group in the per-protocol unadjusted analysis
eFigure 6. Parent reported sleep problems in their child by study group (per-protocol analysis)
eFigure 7. Association between degrees of adherence with the EIG recommended weekly dose of allergenic protein consumption and night time sleep characteristics

© 2018 Perkin et al. *JAMA Pediatrics.*

eMethods

Instructions given to participants regarding solid food introduction

EIG introduction regimen
Following normal blood test results at enrollment (complete blood count, bone, liver, renal and lipid profile tests), the EIG infants then proceeded to introduce baby rice and/or pureed fruits or vegetables during the first week until they were established on solid food. They then continued with these solids and additionally introduced cow's milk yogurt on two days of the second week. During weeks three and four of the early introduction regimen, peanut, hard-boiled egg, sesame and white fish (cod) were introduced sequentially in a random order with two new foods introduced per week. Finally, wheat was introduced in week five, reflecting the guidance in place at the time the EAT study commenced on optimal timing of wheat introduction after four months of age[1] and by week six infants were ideally consuming the required amount of all six allergenic foods each week.[2]
The introduction of non-allergenic foods was not restricted during this process, and the allergenic foods could be given in combination with other foods or each other once the allergenic foods had been successfully introduced and well tolerated.
The exact volume of allergen protein necessary to induce oral tolerance is unknown, but participants were asked to consume the equivalent of 2g of each allergenic food protein twice each week (4g of allergen protein per food per week). The full weekly amount for the allergenic foods therefore consisted of two small 40-60g portions of cow's milk yogurt, three rounded teaspoons of peanut butter, one small egg (<53g), three rounded teaspoons of sesame paste, 25g of white fish and two wheat-based cereal biscuits (e.g. Weetabix).[2]

SIG introduction regimen
For the SIG the criteria in eTable 7 reflect a pragmatic interpretation of the current UK status quo – i.e. an incomplete following of the infant feeding recommendations. Thus allergenic food introduction from five months (criterion C) by which point 75% of mothers in the Infant Feeding Survey 2010 (IFS2010)[3] have introduced solids to their baby including rusks (hard, dry infant biscuits) and yogurts and up to 300 mls per day of cow's milk formula consumption after enrollment (criterion D) were considered acceptable. The volume was chosen such that the majority of milk consumed by the infant was still breastmilk. The amount of breastmilk consumed by exclusively breastfed infants was reviewed in a recent systematic review.[4] At 3-4 months of age the mean transfer volume of breastmilk was 779 (standard deviation (SD) 40) grams/day, at 5 months 827 (SD 39) grams/day, and at 6 months 894 (SD 87) grams/day.

Instructions given to participants regarding formula milk introduction

Mothers in both groups were encouraged to adhere to the World Health Organization (WHO) Global Strategy for Infant and Young Child Feeding,[3] endorsed by the UK Government,[5] which recommends breastfeeding up to the age of two years or beyond. The same document recommends exclusive breastfeeding for the first six months. Mothers in the SIG were asked to adhere to the UK Government's more pragmatic target of ***around*** six months exclusive breastfeeding.[6]
Mothers in both groups were discouraged from introducing formula milk, particularly during the key early introduction period (up to six months) as formula milk introduction has been shown to have a deleterious effect on breastfeeding performance.[7] If they did introduce formula milk of their own volition, whilst still breastfeeding, they were encouraged to keep the volume given as small as possible, to ensure that breast milk remained the principal source of milk.

Monitoring of food consumption in the EAT study

An online questionnaire completed monthly until 12 months of age and every three months between 12 and 36 months of age by the infants' parents was the main portal of communicating information about the health and diet of the participants to the study team. Within this online questionnaire, both groups completed a food frequency questionnaire section assessing how frequently foods including the six study allergens were being consumed.
EIG families kept a prospective weekly diary up until one year of age and monthly thereafter to assess the degree to which they were meeting the consumption target of 4g of each allergenic food protein per week.

© 2018 Perkin et al. *JAMA Pediatrics.*

For each of the last four complete weeks preceding the child's monthly birthday and for each of the allergenic foods, parents recorded the percentage of the recommended amount of food their child was consuming (100%, 75%, 50%, 25% or less, not tried yet) with guidance provided on the amount of each food constituting those percentages. This diary data was then entered into the online questionnaires. Finally, at 6, 12 and 36 months of age parents completed a five day food diary recording in detail their child's diet including portion sizes, ingredients and commercial food brands to allow a comprehensive review of the foods (allergenic and non-allergenic) being consumed and a full nutritional breakdown of macro nutrients during the trial.

Statistical analysis

An independent statistician performed a multiple imputation analysis using the default settings in the mice R package. eTable 2 summarizes the multivariable mixed-effects model multiple imputation analysis. Mixed effects models were fit to each imputed dataset and fixed effects estimates and associated variance-covariance matrices were pooled across these model fits. The treatment effect estimates are slightly smaller than the complete case analysis (eTable 3), which could be partly due to the association between missing data and noncompliance. Nevertheless, the treatment effect remained statistically significant and the effect estimates were similar to estimates using non-imputed data.

© 2018 Perkin et al. *JAMA Pediatrics.*

eResults

Age of introduction of solids in the EAT study groups
By four months of age (17 weeks), 2.2% of the SIG had introduced solids versus 66.0% in the EIG, by five months of age (21 weeks) the corresponding figures were 23.3% versus 96.8% , and by six months of age 70.2% versus 99.3%.

Effect on breastfeeding during the key early introduction period
Virtually no mother had stopped breastfeeding by the end of the key early introduction period. In the EIG 97% (593/610) of infants were still being breastfed, alongside solid food consumption, at six months of age. In the SIG 98% (618/633) of mothers were still breastfeeding by the time their child was six months of age.

Formula milk introduction during the key early introduction period
Cow's milk formula introduction was minimal in both groups before six months: 2% in the SIG and 3% in the EIG ever having had cow's milk formula by 4 months and 7% in both groups by 5 months.

Differential questionnaire completion rates
EIG participants consistently completed fewer questionnaires throughout the study and therefore had more missing data on sleep outcomes (eFigure 1). This could potentially introduce a bias on sleep outcome in the EIG by excluding for analysis those participants who had the most disrupted sleep. However, when the non-response rate was relatively low (12 and 36 months) as compared to the adjacent assessments (11, 15, 24 months), the mean hours of sleep within the EIG increased at these time points. If the mean hours of sleep observed at the more complete sleep assessments is indicative of the overall effect, then the non-response would conservatively bias the results towards the null hypothesis.

Factors influencing nocturnal sleep characteristics at enrollment
The differences in nocturnal sleep characteristics at enrollment are explored further in regression models for duration of nocturnal sleep (eTable 5) and night time wakings (eTable 6). These models include potential confounding factors that were assessed in the EAT study, for example, ethnicity, visible eczema at enrollment, number of siblings, maternal education, smoking and quality of life, child care attendance and baby's weight gain and enrollment weight.
When potential confounding factors were mutually adjusted for each other, Black, Asian or Chinese three month old infants had forty minutes less sleep per night. Infants of mixed ethnicity showed a trend towards increased night waking (waking at night approximately 2 times more per week than the Caucasian infants at enrollment, p=0.08).
Visible eczema and its severity at enrollment, after adjustment for other potential confounding factors, was not associated with duration of nocturnal sleep (eTable 5) but moderate to severe eczema was associated with increased night waking at enrollment (eTable 6).
Sibship size was also associated with nocturnal sleep with larger families having more sleep, reaching statistical significance for having one (+0.21 hours per night, p=0.02) or two older siblings (+0.28 hours per night, p=0.02).
The heaviest infants at enrollment (in the top quartile) slept the longest at night (+0.30 hours per night, p=0.05) (eTable 5 and eTable 6). Conversely, those with the highest weight gain between birth and enrollment were most likely to be waking at night (0.21 more night wakings per night, p=0.09).
Breastfeeding duration and frequency in SIG mothers at enrollment were the same in mothers who subsequently introduced solids before six months compared with those subsequently introducing solids after six months (data not shown). There was no association between the number of night wakings at enrollment in the SIG and the age at which solids were subsequently introduced into the infant's diet (data not shown).
Maternal quality of life scores in both the physical and environmental domains at enrollment were significantly associated with duration of infant sleep (eTable 5).
Infants at enrollment had significantly less sleep if they were placed to sleep in the parental bedroom compared with being in their own room. Stronger still was the influence of how the baby was soothed to sleep. If at enrollment they were simply placed in bed alone to go to sleep, they slept for 50 minutes longer per night (eTable 5). Similar effects were seen on night wakings (eTable 6). Infants sleeping in the parental bedroom at enrollment woke significantly more frequently, particularly if they were sleeping in the parental

© 2018 Perkin et al. *JAMA Pediatrics.*

bed waking 1.28 times per night more frequently than those put to bed in their own room. Similarly those infants placed in bed to go to sleep alone were significantly less likely to wake at night at enrollment.

Soothing method and sleeping location post enrollment

At enrollment, marginally more mothers in the EIG fed their baby to sleep. The same at 4 months. Beyond 4 months there was a statistically non-significant trend towards the reverse direction with more SIG mothers feeding their infant to sleep but the absolute difference was small. At the same time there was a non-significant trend over time towards more EIG mothers putting down their baby to fall asleep alone as their soothing method.

Longitudinal effect of eczema

In a longitudinal model, enrollment SCORAD severity was associated with less sleep and more night wakings. Furthermore, the enhanced nocturnal sleep characteristics conferred by the early introduction of solids was observed across all categories of enrollment SCORAD severity (eFigure 2).

Longitudinal maternal quality of life and infant/child sleep characteristics and parent reported infant/child sleep problems

At 3, 12 and 36 months of age there was a strong correlation between both maternal quality of life scores and infant/child sleep characteristics (duration of night time sleep and frequency of night wakings) with parent reporting of their infant/child having a sleep problem at the same time point (eFigure 4).

Nocturnal sleep characteristics - Per-protocol analysis

The 42% of EIG participants who were per-protocol adherent were consuming significant quantities of allergenic foods from an early age, and in these infants the effect on sleep parameters was even more pronounced compared with the 92% of SIG participants who were per-protocol (eFigure 5).

In the mixed-effects model (complete case analysis), over the duration of the study, EIG per-protocol infants were sleeping on average 15.8 minutes longer per night ($p<0.001$) (eFigure 3), with the difference peaking at 27.2 minutes per night at 6 months of age. Over the duration of the study, on average SIG per-protocol infants were waking 31% more frequently than EIG infants ($p<0.001$) (eFigure 3).

The effect on parent reported sleep problems was also stronger in the per-protocol comparison, where the odds ratio of any sleep problem was 1.7 (95% CI 1.39-2.10, $p<0.001$), and the odds ratio of a very serious sleep problem was 3.0 (95% CI 1.77-5.11, $p<.0001$) (eFigure 6).

A significant dose response relationship was observed in the EIG infants between increasing quantity of the recommended weekly dose of allergenic food consumed and night time sleep. At 6 months of age, those in the lowest tertile of food consumption quantity were waking on average 45% more per night than those in the upper tertile, and they slept on average 21 minutes less (eFigure 7).

© 2018 Perkin et al. *JAMA Pediatrics.*

eDiscussion

Soothing method and sleeping location at enrollment

The marked differences in nocturnal sleep at enrollment noted with infant soothing technique and infant sleep location, again suggest the importance of infant maturity in establishing early sleep patterns. It seems likely that infant maturity would influence a mother's decision to put her enrollment (3 month old) infant to sleep in their own room, and also whether to adopt a soothing practice of simply putting the baby to bed alone to allow them to go to sleep.

© 2018 Perkin et al. *JAMA Pediatrics.*

eTable 1. Brief Infant Sleep Questionnaire (BISQ)[8]

BISQ Question	Response options	Variable
Sleeping arrangement	Infant crib in a separate room<br>Infant crib in parents' room<br>In parents' bed<br>Infant crib in room with sibling<br>Other, Specify:	Sleeping Location
In what position does your child sleep most of the time?	On his/her belly<br>On his/her side<br>On his/her back	Sleep Position
How much time does your child spend in sleep during the NIGHT (between 7 in the evening and 7 in the morning)?	Hours: ______ Minutes: ______	Nocturnal Sleep Duration
How much time does your child spend in sleep during the DAY (between 7 in the morning and 7 in the evening)?	Hours: ______ Minutes: ______	Daytime Sleep Duration
Average number of night wakings per night		Number of Night Wakings
How much time during the night does your child spend in wakefulness (from 10 in the evening to 6 in the morning)?	Hours: ______ Minutes: ______	Nocturnal Wakefulness
How long does it take to put your baby to sleep in the evening?	Hours: ______ Minutes: ______	Settling Time
How does your baby fall asleep?	While feeding<br>Being rocked<br>Being held<br>In bed alone<br>In bed near parent	Soothing Method
When does your baby usually fall asleep for the night:	Hours: ______ Minutes: ______	Sleep Onset Time
Do you consider your child's sleep as a problem?	A very serious problem<br>A small problem<br>Not a problem at all	Sleep Problem Rating

The BISQ was developed on the basis of a review of the infant sleep literature in search of meaningful variables, particularly clinical studies based on the use of subjective and objective infant sleep measures. The parents were instructed to refer to their child's sleep during the past week.

© 2018 Perkin et al. *JAMA Pediatrics.*

eTable 2. Multivariable mixed-effects multiple imputation analysis model of night time sleep duration - model estimates summary table

Term	Level	Mixed-effects (multiple imputation)			
		Estimate	SE	95% CI	P-value
Intercept		7.2222	0.2667	6.699, 7.7450	<.001
Enrollment Where Sleeps	*Cot Parent's Room vs Cot Alone*	-0.2755	0.0597	-0.393, -0.1585	<.001
	Parent's Bed vs Cot Alone	-0.5607	0.0918	-0.741, -0.3807	<.001
	Bed Sibling's Room vs Cot Alone	-1.0597	0.2625	-1.574, -0.5449	<.001
Enrollment How Sleeps	*Being Rocked vs While Feeding*	-0.0173	0.1242	-0.261, 0.2265	0.889
	Being Held vs While Feeding	0.0815	0.0803	-0.076, 0.2389	0.310
	Alone in Bed vs While Feeding	0.1808	0.0580	0.067, 0.2944	0.002
	Bed Near Parent vs While Feeding	0.0242	0.0721	-0.117, 0.1656	0.738
Enrollment Sleep Hours		0.2504	0.0172	0.217, 0.2841	<.001
SCORAD		-0.0033	0.0035	-0.010, 0.0035	0.340
Ethnicity	*Mixed vs Asian/Black/Chinese/Other*	0.5356	0.1351	0.271, 0.8006	<.001
Ethnicity	*White vs Asian/Black/Chinese/Other*	0.7027	0.1139	0.479, 0.9263	<.001
Enrollment Weight		-0.0784	0.0301	-0.137, -0.0194	0.009
Visit Month	*Linear*	0.1049	0.0046	0.096, 0.1140	<.001
	Non-linear	-0.1308	0.0070	-0.145, -0.1170	<.001
Study group	*Standard vs Early Introduction Group*	-0.1210	0.0451	-0.209, -0.0325	0.007

© 2018 Perkin et al. *JAMA Pediatrics.*

eTable 3. Complete case sensitivity analyses of night time sleep duration: comparison of four different multivariable modelling strategies to assess the robustness of the primary analysis results

		OLS		OLS (robust)		GLS		Mixed-effects	
**Term**	**Level**	**Estimate**	**SE**	**Estimate**	**SE**	**Estimate**	**SE**	**Estimate**	**SE**
Intercept		7.2	0.13	7.2	0.29	7.4	0.18	7.2	0.26
Enrollment Where Sleeps	*Cot Parent's Room vs Cot Alone*	-0.29	0.028	-0.29	0.049	-0.28	0.04	-0.28	0.059
	Parent's Bed vs Cot Alone	-0.61	0.043	-0.61	0.08	-0.57	0.06	-0.6	0.089
	Bed Sibling's Room vs Cot Alone	-1.1	0.11	-1.1	0.5	-1.2	0.16	-1.1	0.25
Enrollment How Sleeps	*Being Rocked vs While Feeding*	0.017	0.054	0.017	0.12	-0.043	0.076	-0.013	0.11
	Being Held vs While Feeding	0.092	0.037	0.092	0.076	0.084	0.053	0.081	0.077
	Alone in Bed vs While Feeding	0.21	0.027	0.21	0.054	0.2	0.038	0.2	0.056
	Bed Near Parent vs While Feeding	-0.018	0.034	-0.018	0.075	0.016	0.048	0.0087	0.071
Enrollment Sleep Hours		0.26	0.0077	0.26	0.019	0.24	0.011	0.26	0.016
SCORAD		-0.0031	0.0015	-0.0031	0.0034	-0.0034	0.0022	-0.0042	0.0032
Ethnicity	*Mixed vs Asian/Black/Chinese/Other*	0.57	0.063	0.57	0.16	0.55	0.089	0.53	0.13
Ethnicity	*White vs Asian/Black/Chinese/Other*	0.7	0.054	0.7	0.14	0.67	0.076	0.69	0.11
Enrollment Weight		-0.086	0.014	-0.086	0.029	-0.084	0.02	-0.077	0.029
Visit Month	*Linear*	0.1	0.0044	0.1	0.0052	0.11	0.0056	0.1	0.0044
	Non-linear	-0.13	0.0069	-0.13	0.0076	-0.13	0.0085	-0.13	0.0068
Study group	*Standard vs Early Introduction Group*	-0.17	0.021	-0.17	0.043	-0.15	0.03	-0.14	0.044

Complete case sensitivity analyses of night time sleep hours are shown above. The parameter estimates (SEs) from four different modelling strategies are presented along with their standard errors. The OLS (ordinary least squares) approach is not valid with repeated measures and is presented only as a reference. The OLS (robust) model uses the Huber-White method,[9] as implemented in Frank Harrell's rms R package, to correct the variance-covariance matrix from ordinary least squares for clustered observations. The generalized least squares (GLS), provided by the nlme R package, is an extension of the standard linear model allowing for errors to be correlated and have unequal variances. Lastly, the "mixed-effects" column provides estimates from a mixed-effects model using a complete case analysis rather than the multiple imputation method presented in the main body of the manuscript and in eTable 2.

© 2018 Perkin et al. *JAMA Pediatrics.*

eTable 4. Baseline demographic data

		SIG (n=651)<br>% (n/N)	EIG (n=652)<br>% (n/N)
**Demography**			
Mean age at enrollment (months)<br>(SD)		3.39 (n=651)<br>(0.24)	3.38 (n=652)<br>(0.22)
Mean age at three year visit (months)<br>(SD)		37.9 (n=601)<br>(3.3)	37.9 (n=572)<br>(3.4)
Sex:	Male	52.1 (339/651)	48.2 (314/652)
	Female	47.9 (312/651)	51.8 (338/652)
Ethnicity:	White	84.0 (547/651)	85.4 (557/652)
	Black	2.9 (19/651)	3.4 (22/652)
	Asian†	1.7 (11/651)	2.6 (17/652)
	Chinese	0.5 (3/651)	1.2 (8/652)
	Mixed	10.9 (71/651)	7.4 (48/652)
Pet ownership		44.6 (290/650)	40.6 (264/651)
Maternal education:	≤16	6.2 (40/650)	5.2 (34/652)
(age at leaving full-time education)	17-18	13.7 (89/650)	12.7 (83/652)
	>18	80.2 (521/650)	82.1 (535/652)
**Smoking**			
Maternal		3.1 (20/650)	3.4 (22/651)
Paternal		10.9 (71/650)	10.8 (70/651)
**Family history**			
Median maternal age (years):		33 (n=650)<br>(range 19 – 46)	33.5 (n=652)<br>(range 19 – 45)
Siblings	0	38.3 (249/651)	37.3 (243/652)
	1	36.9 (240/651)	39.3 (256/652)
	2	16.4 (107/651)	14.9 (97/652)
	3+	8.5 (55/651)	8.6 (56/652)
**Birth history**			
Mean birth weight grams<br>(SD)		3560 (n=651)<br>(487)	3570 (n=651)<br>(489)
Mode of delivery:*	Vaginal	77.3 (503/651)	72.4 (472/652)
	Caesarean	22.7 (148/651)	27.6 (180/652)
Mean gestational age (weeks)		39.7 (n=651)	39.9 (n=652)
**Participant enrollment atopy status**			
Skin-prick test positive (>0 mm)		N/A	5.1 (33/652)
Visible eczema		24.2 (157/650)	24.5 (160/652)
Median SCORAD<br>(infants with eczema)		7.5 (n=157)<br>(range 3.5 – 49.2)	7.5 (n=160)<br>(range 3.5 – 75.0)
**EIG median age of allergenic food first consumption (weeks)**			
Dairy		-	17.3
Egg		-	19.6
Fish		-	19.6
Sesame		-	19.6
Peanut		-	19.6
Wheat		-	20.6
**Family atopy status (self-reported)**			
Maternal			
Eczema		34.2 (222/650)	34.9 (227/651)
Asthma		26.8 (174/650)	25.8 (168/651)
Maternal atopy‡		63.2 (411/650)	61.9 (403/651)
Paternal			
Eczema		21.1 (137/650)	18.9 (123/651)
Asthma		23.5 (153/650)	21.8 (142/651)
Paternal atopy‡		55.7 (362/650)	50.5 (329/651)
**Maternal allergenic food consumption**			
During pregnancy		100.0 (639/639)	100.0 (631/631)
During breastfeeding		100.0 (639/639)	100.0 (631/631)

* P < 0.05 † Asian refers to Indian, Pakistani and Bangladeshi ‡Eczema, asthma or hay fever

© 2018 Perkin et al. *JAMA Pediatrics.*

eTable 5. Regression analysis of factors influencing duration of nocturnal sleep in the EAT study participants at enrollment (3 months of age: n=1209)

Variable		Number n (%)	Nocturnal sleep Hours (95% CI)	
			Unadjusted	Mutually adjusted*
Ethnicity	*White (Baseline)*	1041 (86.1)	+0	+0
	Mixed	109 (9.0)	-0.42 (-0.71--0.14)	-0.21 (-0.48-0.06)
	Black/Asian/Chinese	59 (4.9)	-1.12 (-1.50--0.75)	-0.64 (-1.01--0.27)
Enrollment visible eczema	*None*	914 (75.6)	+0	+0
	SCORAD 1-15	229 (18.9)	0.06 (-0.15-0.27)	0.03 (-0.17-0.22)
	SCORAD >15	66 (5.5)	-0.19 (-0.55-0.18)	-0.15 (-0.49-0.19)
Number of siblings	*0 (Baseline)*	465 (38.5)	+0	+0
	1	465 (38.5)	0.17 (-0.01-0.36)	0.21 (0.03-0.39)
	2	183 (15.1)	0.25 (0.01-0.50)	0.28 (0.05-0.52)
	3+	96 (7.9)	-0.47 (-0.79--0.15)	-0.12 (-0.44-0.19)
Age completed maternal education	*Post 18 (baseline)*	985 (81.5)	+0	+0
	17-18	159 (13.1)	-0.18 (-0.42-0.07)	-0.16 (-0.39-0.07)
	16	65 (5.4)	-0.51 (-0.88--0.14)	-0.22 (-0.57-0.13)
Attending childcare		27 (2.2)	0.59 (0.03-1.14)	0.33 (-0.19-0.84)
Maternal smoking		151 (12.5)	-0.38 (-0.63--0.13)	-0.13 (-0.37-0.11)
Maternal QOL score physical		-	0.16 (0.12-0.21)	0.09 (0.03-0.14)
Maternal QOL score psychological		-	0.09 (0.05-0.13)	-0.01 (-0.06-0.04)
Maternal QOL score social		-	0.07 (0.04-0.10)	0.00 (-0.03-0.04)
Maternal QOL score environmental		-	0.15 (0.11-0.20)	0.05 (0.00-0.11)
Weight gain: birth to enrollment	*Lowest quartile*	307 (25.4)	+0	+0
	2nd quartile	305 (25.2)	-0.21 (-0.44-0.02)	-0.12 (-0.34-0.11)
	3rd quartile	302 (25.0)	-0.09 (-0.33-0.14)	-0.11 (-0.37-0.15)
	Highest quartile	295 (24.4)	0.07 (-0.17-0.30)	-0.09 (-0.39-0.21)
Enrollment weight	*Lowest quartile*	307 (25.4)	+0	+0
	2nd quartile	304 (25.1)	0.00 (-0.23-0.23)	0.06 (-0.16-0.29)
	3rd quartile	311 (25.7)	0.09 (-0.14-0.32)	0.11 (-0.15-0.36)
	Highest quartile	287 (23.7)	0.28 (0.04-0.51)	0.30 (0.00-0.60)
Where baby sleeps	*Cot/Moses basket in separate room (baseline)*	226 (18.7)	+0	+0
	Cot/Moses basket in parental bedroom	840 (69.5)	-0.56 (-0.78--0.35)	-0.29 (-0.50--0.09)
	In parents' bed	133 (11.0)	-1.01 (-1.32--0.70)	-0.43 (-0.74--0.13)
	Cot/Moses basket in siblings bedroom	10 (0.8)	0.35 (-0.55-1.26)	0.20 (-0.66-1.07)
How baby goes to sleep	*While feeding (baseline)*	485 (40.1)	+0	+0
	Being rocked	53 (4.4)	-0.13 (-0.52-0.26)	-0.15 (-0.53-0.23)
	Being held	127 (10.5)	-0.02 (-0.29-0.25)	0.02 (-0.24-0.29)
	In bed alone	386 (31.9)	1.02 (0.83-1.20)	0.81 (0.63-1.00)
	In bed near parent	158 (13.1)	0.23 (-0.02-0.48)	0.19 (-0.05-0.44)

*Adjusted for all other variables in the table

© 2018 Perkin et al. *JAMA Pediatrics.*

eTable 6. Regression analysis of factors influencing frequency of night time waking in the EAT study participants at enrollment (3 months of age: n=1210)

Variable		Number n (%)	Number of night wakings Hours (95% CI)	
			Unadjusted	Mutually adjusted*
Ethnicity	*White (Baseline)*	1042 (86.1)	+0	+0
	Mixed	109 (9.0)	0.42 (0.18-0.65)	0.20 (-0.02-0.42)
	Black/Asian/Chinese	59 (4.9)	0.49 (0.18-0.81)	-0.02 (-0.31-0.28)
Enrollment visible eczema	*None*	914 (75.5)	+0	+0
	SCORAD 1-15	229 (18.9)	-0.07 (-0.24-0.11)	-0.05 (-0.21-0.11)
	SCORAD >15	67 (5.5)	0.39 (0.09-0.69)	0.33 (0.06-0.60)
Number of siblings	*0 (Baseline)*	465 (38.4)	+0	+0
	1	465 (38.4)	0.12 (-0.04-0.27)	0.07 (-0.07-0.22)
	2	183 (15.1)	-0.13 (-0.34-0.07)	-0.12 (-0.31-0.06)
	3+	97 (8.0)	0.24 (-0.02-0.50)	0.03 (-0.22-0.28)
Age completed maternal education	*Post 18 (baseline)*	986 (81.5)	+0	+0
	17-18	159 (13.1)	-0.08 (-0.28-0.12)	-0.03 (-0.22-0.16)
	16	65 (5.4)	0.09 (-0.21-0.39)	0.00 (-0.28-0.29)
Attending childcare		27 (2.2)	-0.09 (-0.55-0.37)	0.15 (-0.27-0.57)
Maternal smoking		152 (12.6)	0.09 (-0.11-0.30)	-0.03 (-0.23-0.16)
Maternal QOL score physical		-	-0.16 (-0.19--0.12)	-0.18 (-0.23--0.14)
Maternal QOL score psychological		-	-0.03 (-0.06-0.00)	0.09 (0.05-0.13)
Maternal QOL score social		-	-0.05 (-0.07--0.02)	-0.02 (-0.05-0.00)
Maternal QOL score environmental		-	-0.06 (-0.10--0.03)	0.03 (-0.01-0.08)
Weight gain: birth to enrollment	*Lowest quartile*	308 (25.4)	+0	+0
	2nd quartile	305 (25.2)	0.01 (-0.18-0.20)	-0.07 (-0.25-0.12)
	3rd quartile	302 (25.0)	0.07 (-0.12-0.26)	0.07 (-0.14-0.28)
	Highest quartile	295 (24.4)	0.23 (0.04-0.42)	0.21 (-0.03-0.45)
Enrollment weight	*Lowest quartile*	308 (25.5)	+0	+0
	2nd quartile	304 (25.1)	0.06 (-0.13-0.25)	0.03 (-0.15-0.22)
	3rd quartile	311 (25.7)	0.01 (-0.18-0.20)	-0.02 (-0.23-0.19)
	Highest quartile	287 (23.7)	0.12 (-0.07-0.32)	-0.04 (-0.29-0.20)
Where baby sleeps	*Cot/Moses basket in separate room (baseline)*	226 (18.7)	+0	+0
	Cot/Moses basket in parental bedroom	840 (69.4)	0.53 (0.36-0.69)	0.43 (0.27-0.60)
	In parents' bed	134 (11.1)	1.46 (1.22-1.70)	1.28 (1.04-1.53)
	Cot/Moses basket in siblings bedroom	10 (0.8)	-0.03 (-0.75-0.69)	0.00 (-0.70-0.70)
How baby goes to sleep	*While feeding (baseline)*	486 (40.2)	+0	+0
	Being rocked	53 (4.4)	-0.04 (-0.37-0.29)	0.00 (-0.31-0.31)
	Being held	127 (10.5)	-0.04 (-0.27-0.19)	-0.01 (-0.23-0.20)
	In bed alone	386 (31.9)	-0.58 (-0.74--0.43)	-0.34 (-0.49--0.19)
	In bed near parent	158 (13.1)	-0.15 (-0.36-0.06)	-0.14 (-0.34-0.05)

*Adjusted for all other variables in the table

© 2018 Perkin et al. *JAMA Pediatrics.*

eTable 7. Overall per-protocol compliance criteria in the EAT study

Compliance definitions	Compliance evaluable children meeting the compliance definitions
**Standard Introduction Group (SIG)**<br>*(N=606/651 children compliance evaluable)*	
• Criterion A: Exclusive breastfeeding for at least three months duration (water and/or oral rehydration solution allowed)	100% (606/606) (A)<br>12.0% have had water by 3 months of age
• Criterion B: Continued breastfeeding up to five months of age	99.7% (604/606) (B)
• Criterion C: No consumption of peanut, egg, sesame, fish or wheat before five months	97.4% (590/606) (C)
• Criterion D: No introduction of cow's milk formula (or goat's milk formula) (or consumption of less than 300 mls/day) between three months and six months of age	(1) No formula pre six months 85.6% (519/606)<br>(2) Consumption of less than 300mls/day 8.8% (53/606)*<br>(1) or (2) 94.4% (572/606) (D)<br>*median age of introduction of 22 weeks
**Overall SIG per-protocol compliance (meets all criteria)**	**92.1% (558/606) (A, B, C & D)**
**Early Introduction Group (EIG)**<br>*(N=529/652 children compliance evaluable)*	
• Criterion A: Exclusive breastfeeding for three months duration (water and/or oral rehydration solution allowed)	100% (529/529) (A)<br>13.1% have had water by 3 months of age
• Criterion B: Continued breastfeeding up to five months of age	99.6% (527/529) (B)
• Criterion C: Consumption of at least five of the allergenic foods in at least 75% of the recommended amount (3g allergen protein/week), for at least five weeks between three months and six months of age	42.3% (224/529) (C)
**Overall EIG per-protocol compliance (meets all criteria)**	**42.2% (223/529) (A, B & C)**

* Compliance status non-evaluable for 7% (45/651) of the SIG and 19% (123/652) of the EIG participants

© 2018 Perkin et al. *JAMA Pediatrics.*

eFigure 1. Examination of the proportion of missing sleep assessments and the corresponding mean night time sleep hours

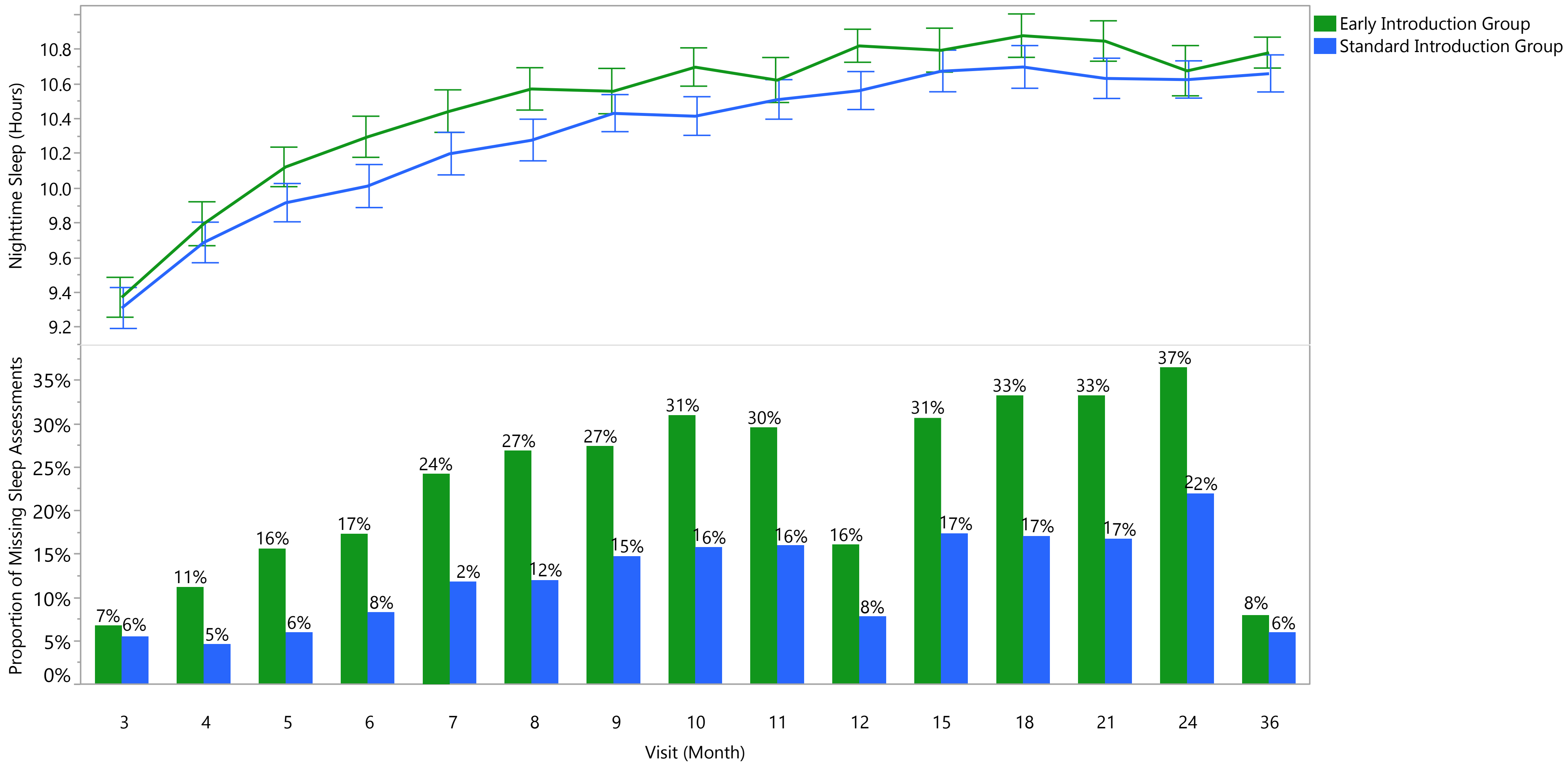

The interim questionnaire non-response rate increased over time in both groups (bar chart, bottom row) and was consistently higher in the EIG than the SIG. The line graph (top row) shows mean hours of night time sleep and 95% confidence intervals in the ITT population. Note the relatively higher post-baseline mean of night time sleep hours in the EIG when the proportion of missing sleep assessments is lower in the EIG. Specifically, when the non-response rate was relatively low (12 and 36 months) compared to the adjacent assessments (11, 15, 24 months) the mean hours of sleep within the EIG increases. If the mean hours of sleep observed at the more complete sleep assessments is indicative of the overall effect, then the non-response would conservatively bias the results towards the null hypothesis. Moreover, the data were assumed to be missing at random (MAR) where the mixed model is known to give unbiased estimates by accounting for the within subject correlation of completed assessments. Lastly, we performed a multivariable mixed-effects multiple imputation analysis (eTable 2) which showed that the treatment effect estimate was robust to missing data. For these reasons and given the untestable assumptions of MAR, the presented ITT analysis is thought to be a conservative estimate of the intervention effect.

© 2018 Perkin et al. *JAMA Pediatrics.*

eFigure 2. Association between enrollment SCORAD status and nocturnal sleep characteristics by study group (intention to treat analysis)

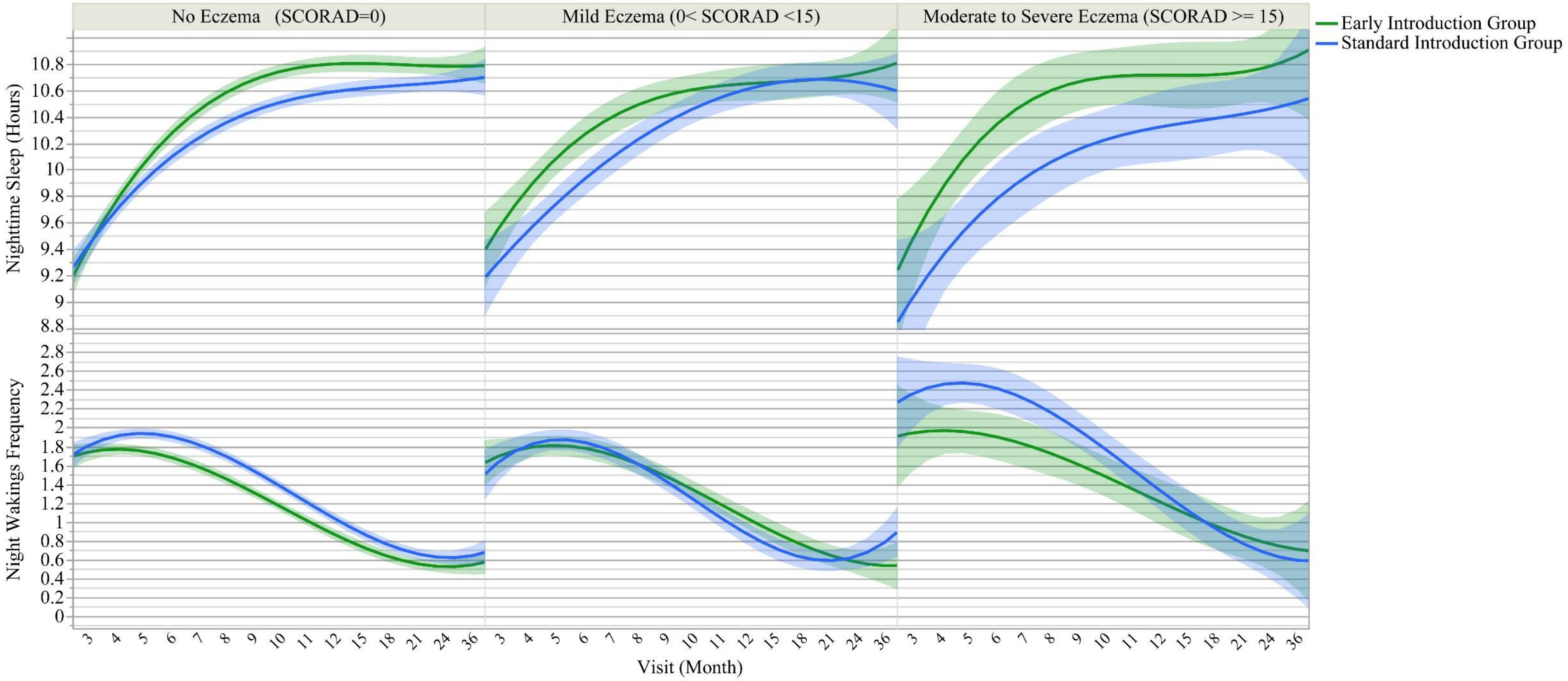

In a multivariate longitudinal model, enrollment SCORAD severity was associated with less sleep and more night wakings and, independently, the early introduction regimen was associated with more sleep and less night wakings. Whilst there is a larger difference in nocturnal sleep duration between study groups in the moderate to severe eczema group compared with the other eczema groups this subgroup difference is not statistically significant. There is no significant interaction effect between the study intervention and SCORAD severity.

© 2018 Perkin et al. *JAMA Pediatrics.*

eFigure 3. Multivariable mixed-effects complete case analysis model: Adjusted differences in nocturnal sleep characteristics between the EIG and SIG in the ITT and PP analyses

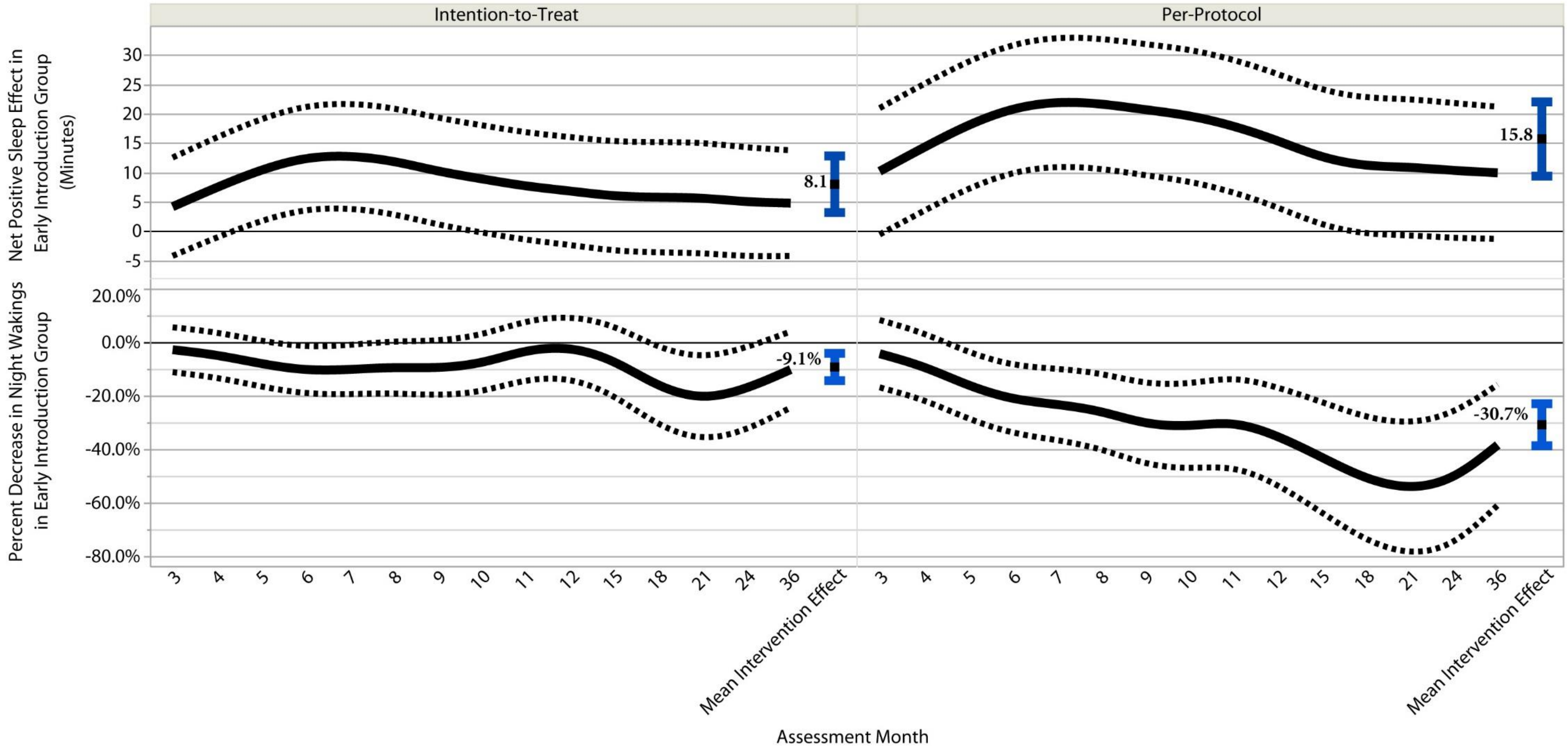

The Mean difference in minutes (EIG-SIG) is shown from the multivariable mixed-effects model. This complete case model is adjusted for: ethnicity, number of siblings, SCORAD at baseline, baseline sleep, age, and where and how the baby goes to sleep. Difference in night wakings are shown as a percentage decrease in wakings in the EIG compared with the SIG.

© 2018 Perkin et al. *JAMA Pediatrics.*

eFigure 4. Correlation at three time points (3, 12 and 36 months) between infant and maternal sleep and maternal quality of life measures (X-axis) and parent reported infant/child sleep problems (Y-axis)

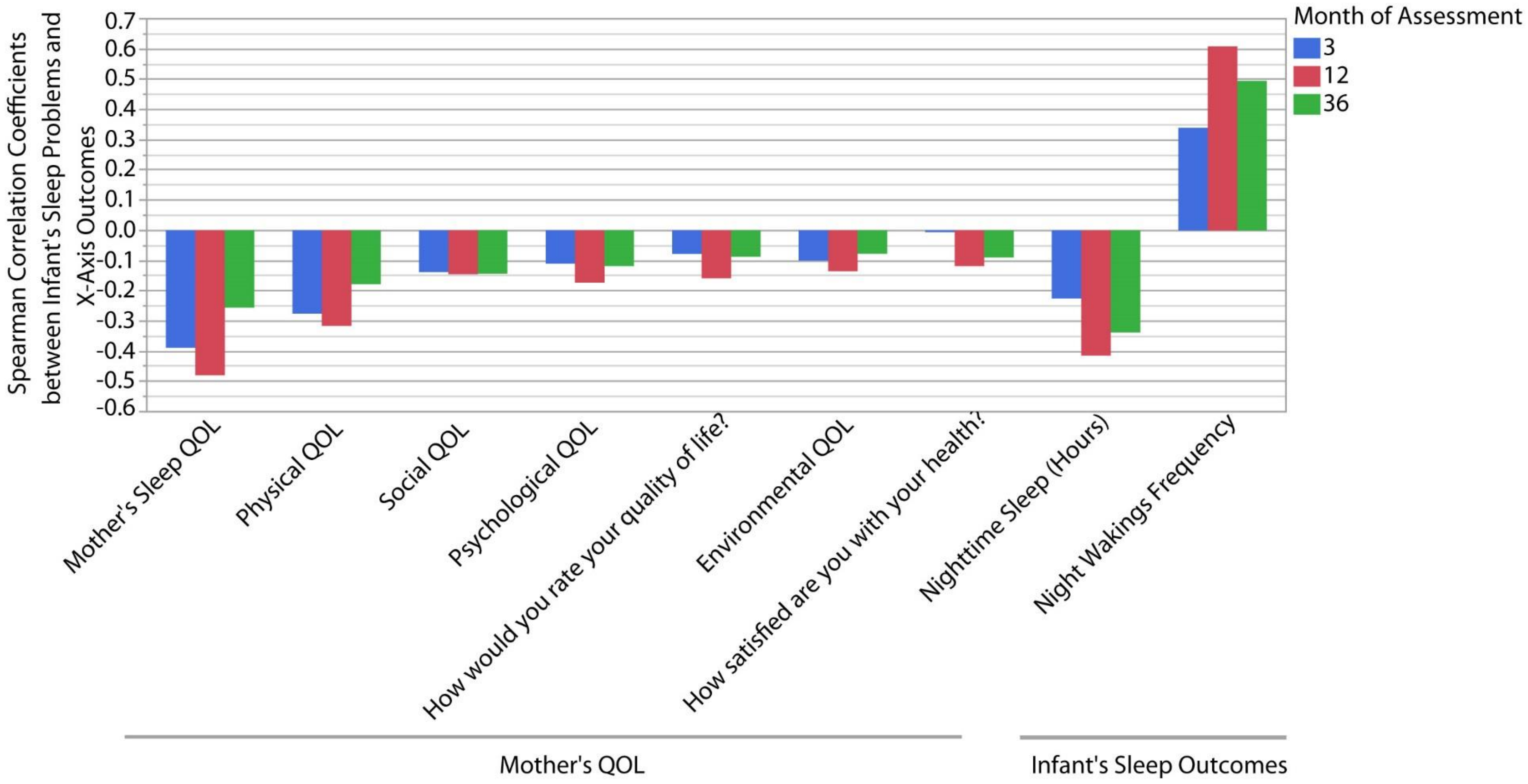

The figure shows the correlation at three time points, 3, 12 and 36 months, between parent perception of their infant/child having a sleep problem (Y-axis) and measures of maternal quality of life and infant/child nocturnal sleep outcomes (X-axis). Maternal quality of life characteristics included in the figure are: overall perception of health and overall perception of quality of life, individual quality of life domain scores (physical, social, psychological and environmental) and the specific maternal quality of sleep question which forms part of the “physical” domain of the quality of life score. Infant/child sleep outcomes include duration of night time sleep and night wakings frequency. These are correlated with the parental BISQ response regarding the consideration of their child having a sleep problem with the three possible responses treated as an ordinal variable (no problem 0, a small problem 1, a very serious problem 2). All correlations are significant ($p < 0.05$) except the three month assessment of Health QOL. The strongest correlations are observed at 12 months of age.

© 2018 Perkin et al. *JAMA Pediatrics.*

eFigure 5. Nocturnal sleep characteristics by study group in the per-protocol unadjusted analysis

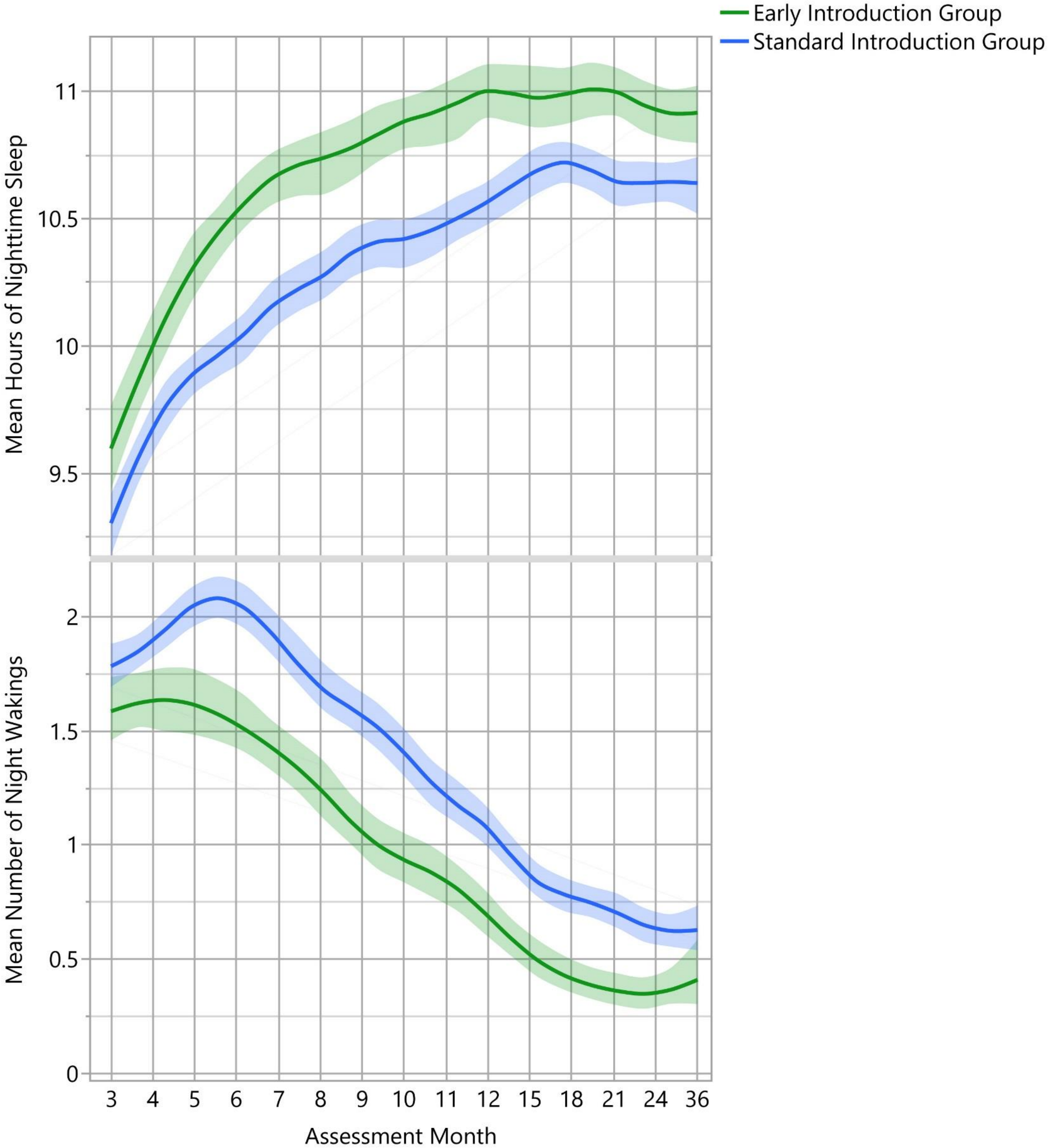

© 2018 Perkin et al. *JAMA Pediatrics.*

eFigure 6. Parent reported sleep problems in their child by study group (per-protocol analysis)

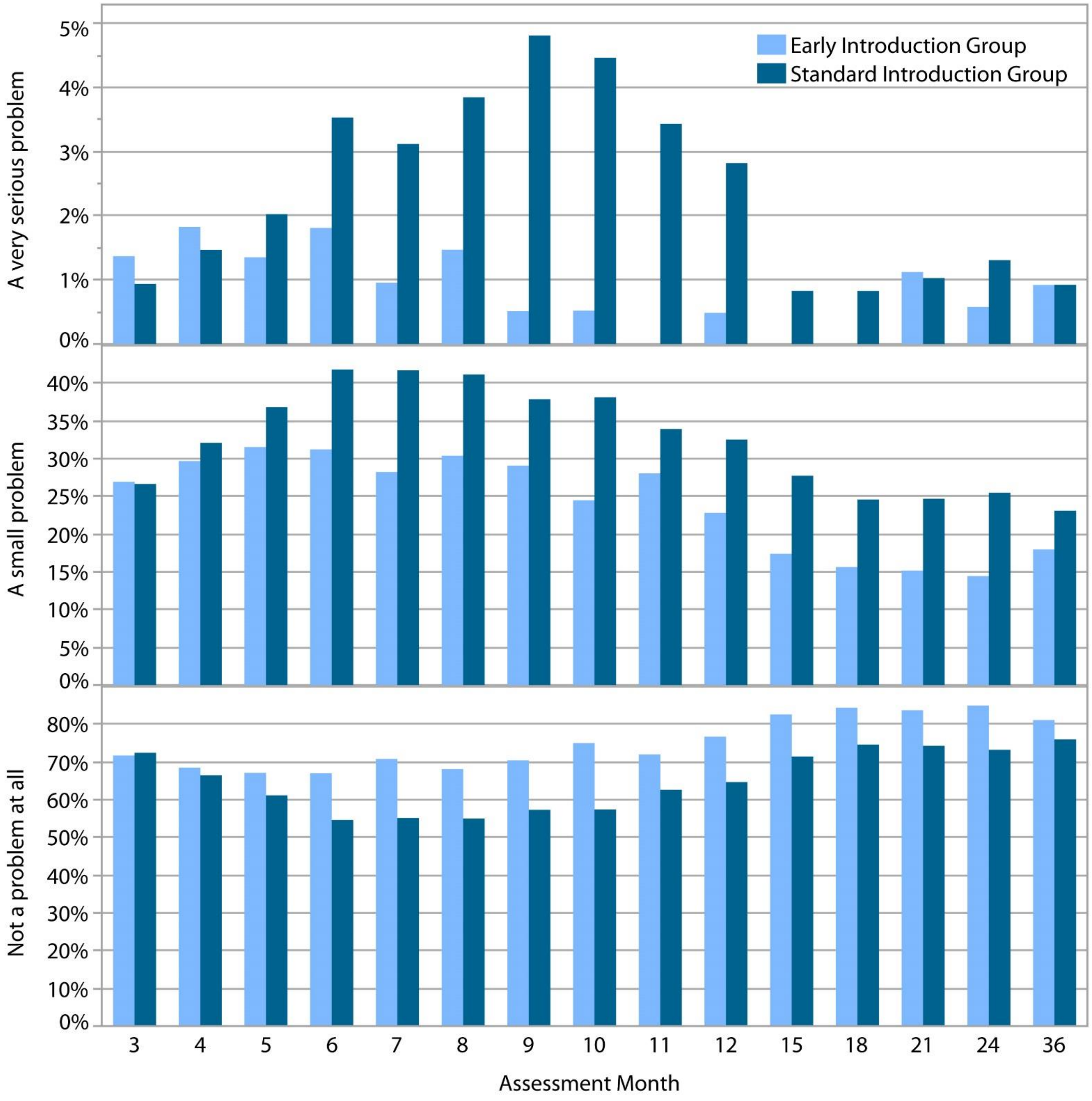

© 2018 Perkin et al. *JAMA Pediatrics.*

eFigure 7. Association between degrees of adherence with the EIG recommended weekly dose of allergenic protein consumption and night time sleep characteristics

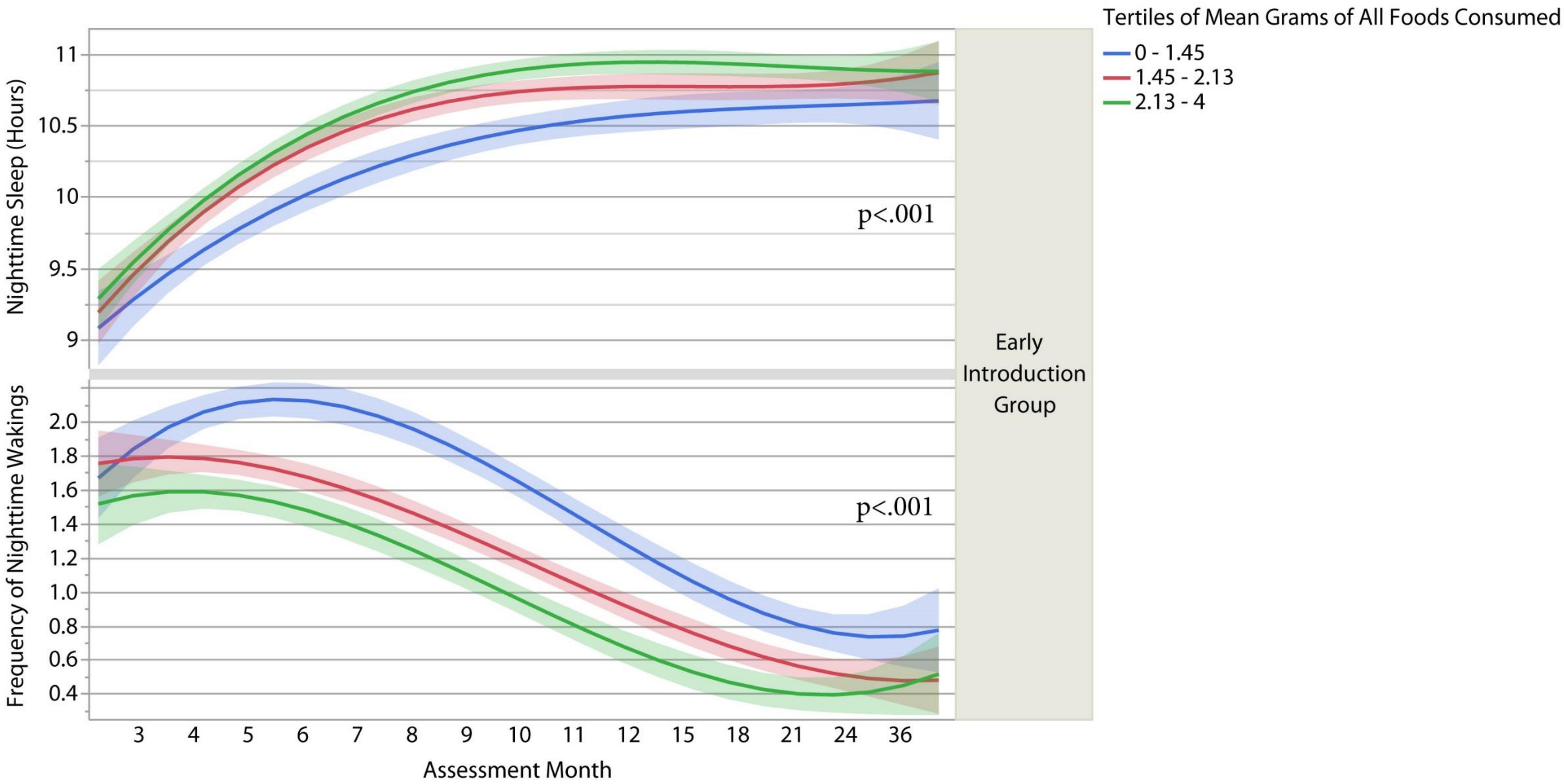

The EIG consumption target was 4g of allergenic food protein for each of the six early introduction foods. The weekly average consumption of the six foods with respect to this 4g target has been calculated during the key early introduction period of between three months and six months of age. This mean consumption has been divided into tertiles and plotted against the nocturnal sleep characteristics. A sustained dose response effect is observed, particularly for the night wakings, with the greater amount of allergenic foods that were being consumed, the less the infant woke at night (p<0.001).

© 2018 Perkin et al. *JAMA Pediatrics.*

References

1. Agostoni C, Decsi T, Fewtrell M, Goulet O, Kolacek S, Koletzko B et al. Complementary feeding: a commentary by the ESPGHAN Committee on Nutrition. J Pediatr Gastroenterol Nutr 2008; 46(1):99-110.

2. Perkin MR, Logan K, Marrs T, Radulovic S, Craven J, Flohr C et al. Enquiring About Tolerance (EAT) study: Feasibility of an early allergenic food introduction regimen. J Allergy Clin Immunol 2016; 137(5):1477-86.

3. McAndrew F, Thompson J, Fellows L, Large A, Speed M, Renfrew MJ. Infant Feeding Survey 2010. 2012. Health and Social Care Information Centre.

4. Reilly JJ, Ashworth S, Wells JC. Metabolisable energy consumption in the exclusively breast-fed infant aged 3--6 months from the developed world: a systematic review. Br J Nutr 2005; 94(1):56-63.

5. Department of Health. Infant Feeding Recommendation. 2003.

6. Department of Health. Weaning - starting solid food. 2008.

7. Hornell A, Hofvander Y, Kylberg E. Solids and formula: association with pattern and duration of breastfeeding. Pediatrics 2001; 107(3):E38.

8. Sadeh A. A brief screening questionnaire for infant sleep problems: validation and findings for an Internet sample. Pediatrics 2004; 113(6):e570-e577.

9. White H. Maximum Likelihood Estimation of Misspecified Models. Econometrica 1982; 50(1):1-25.
