# Supplementary material for: Association of Early Introduction of Solids With Infant Sleep: A Secondary Analysis of a Randomized Clinical Trial
Source: JAMA Pediatr. 2018 Jul 9;172(8):e180739. doi: 10.1001/jamapediatrics.2018.0739 (PMC6142923; doi:10.1001/jamapediatrics.2018.0739)
Supplement: Supplement 2. — Trial Protocol. Original protocol. [file jamapediatr-172-e180739-s002.pdf]

Protocol (T07051)

Randomized controlled trial of early introduction of allergenic foods to induce tolerance in infants

Short Title: Enquiring About Tolerance (EAT) Study

Version 1.02 (April 1st, 2009)

This clinical study is supported by the Food Standards Agency and the Medical Research Council.

PRINCIPAL INVESTIGATOR
Gideon Lack, MD
Professor of Paediatric Allergy
Children's Allergies Department
2nd Floor, Stairwell B, South Wing
St Thomas' Hospital
Lambeth Palace Road
London SE1 7EH
Phone: 020 7188 9730
Fax: 020 7188 9782
E-mail: gideon.lack@kcl.ac.uk

CO-PRINCIPAL INVESTIGATOR
Dr Michael Perkin, PhD
Senior Lecturer in Paediatric Allergy
Children's Allergies Department
2nd Floor, Stairwell B, South Wing
St Thomas' Hospital
Lambeth Palace Road
London SE1 7EH
Phone: 020 7188 8637
Fax: 020 7188 9782
E-mail: michael.perkin@kcl.ac.uk

FOOD STANDARDS AGENCY
Sarah Hardy
Food Standards Agency
Higher Scientific Officer
Aviation House
125 Kingsway
London WC2B 6NH
Phone: 020 7276 8532
Fax : 020 7276 8513
E-mail: sarah.hardy@foodstandards.gsi.gov.uk

MEDICAL RESEARCH COUNCIL
Dr Tom Foulkes PhD
Research Programme Manager
Medical Research Council
20 Park Crescent
London W1B 1AL
Tel: +44 (0) 20 7670 5305
Fax: +44 (0) 20 7637 6003
E-mail: tom.foulkes@headoffice.mrc.ac.uk

Confidentiality Statement

This document is confidential and is to be distributed for review only to investigators, potential investigators, consultants, study staff, and applicable independent ethics committee. The contents of this document shall not be disclosed to others without written authorization from the study co-principal investigators, unless it is necessary to obtain informed consent from potential study participants.

Protocol Approval

Protocol T07051	Version: 1.02
	Protocol Chair: Gideon Lack
Short Title: *Enquiring About Tolerance (EAT) Study*	

ICH/MRC Principles for Good Clinical Practice in Clinical Trials

We confirm that we will adhere to the principles of Good Clinical Practice (GCP) as produced by the International Conference on Harmonisation (ICH) Harmonised Tripartite Guideline for Good Clinical Practice produced in June 1996[1] and adopted by the MRC in March 1998.[2]

- Clinical Trials should be conducted in accordance with the ethical principles that have their origin in the Declaration of Helsinki, and that are consistent with GCP and the applicable regulatory requirement(s).
- Before a trial is initiated, foreseeable risks and inconveniences should be weighed against the anticipated benefit for the individual trial subject.
- The rights, safety, and well-being of the trial subjects are the most important considerations and should prevail over interests of science and society.
- The available non-clinical and clinical information on an investigational product should be adequate to support the trial.
- Clinical trials should be scientifically sound, and described in a clear, detailed protocol.
- A trial should be conducted in compliance with the protocol that has received a favourable independent ethics committee opinion.
- The medical care given to, and medical decisions made on behalf of subjects should always be the responsibility of a qualified physician.
- Each individual involved in conducting a trial should be qualified by education, training, and experience to perform his or her respective task.
- Freely given informed consent should be obtained from every subject prior to clinical trial participation.
- All clinical trial information should be recorded, handled, and stored in a way that allows its accurate reporting, interpretation and verification.
- The confidentiality of records that could identify subjects should be protected, respecting the privacy and confidentiality rules.
- Investigational products should be manufactured, handled, and stored in accordance with applicable good manufacturing practice (GMP).
- Systems with procedures that assure the quality of every aspect of the trial should be implemented.

FSA Joint Code of Practice for Research

We confirm that we are aware of the requirements of the FSA Joint Code of Practice and, in the proposed project, we will use our best efforts to ensure that the procedures used conform to those requirements under the following headings:

- Responsibilities
- Competence
- Project planning
- Quality Control
- Health and safety
- Handling of samples and materials
- Facilities and equipment
- Documentation of procedures and methods
- Research/work records

We understand that the funding body has the right to inspect our procedures and practices against the requirements of the Code of Practice, and that we may be asked to provide documentary evidence of our working practices or provide access and assistance to auditors appointed by the Funding Body.

____

Principal Investigator (Print)

________________________________ ________________

Principal Investigator (Signature) Date

Synopsis

**Title**	Randomized controlled trial of early introduction of allergenic foods to induce tolerance in infants
**Short Title**	Enquiring About Tolerance (EAT) Study
**Sponsors**	Guy's and St Thomas' Foundation NHS Trust & King's College London
**Primary Funders**	Food Standards Agency and the Medical Research Council
**Additional Funders**	National Institute of Health Research
**Conducted by**	Children's Allergies Department, Kings College
**Protocol Chair**	Professor Gideon Lack
**Ethical Approval**	St Thomas' Hospital Research Ethics Committee – 20/10/08 (08/H0802/93)
**Study Registration**	National Institute for Health Research (NIHR) Clinical Research Portfolio: Registration ID 6317<br>International Standard Randomised Controlled Trial Number Register (ISRCTN): 14254740
**Clinical Trials Facility**	Paediatric Allergy Clinical Trials Unit, Evelina Children's Hospital, St. Thomas Hospital, London, UK
**Recruitment Centres**	***Primary centres***<br>Kingston Hospital<br>St Thomas' Hospital<br>***Secondary centres***<br>Queen Charlotte's Hospital<br>Chelsea & Westminster Hospital<br>Royal Free Hospital<br>Kings College Hospital
**Enrolment Objective**	3000 participants
**Study Design**	This is a randomized controlled trial of the early introduction of allergenic foods (and other foods) from 3 months of age in the general population. Mothers will be recruited antenatally from two large antenatal units (Kingston and St Thomas' Hospitals). All participants will be encouraged to breast feed exclusively until three months of age when half of the infants (intervention arm) will be randomized to continue breast feeding and also introduce a number of allergenic foods into the diet from 3 months of age under dietetic direction. Baby rice will be introduced at 3 months of age, followed by cow's milk yoghurt. Subsequently egg, wheat, fish (cod), peanut and sesame will be introduced in random order (but no wheat before 4 months of age).

	Children will consume these foods in recommended quantities until being assessed at one year of age, at which point consumption will be encouraged until completing the study at three years of age. Infants in the intervention arm will be encouraged to consume other foods (allergenic and non-allergenic).<br>The other half (control arm) will be advised to continue exclusively breast feeding until around 6 months of age as per the UK Government weaning advice.<br>All infants will be followed up until 3 years of age by which point the impact of the intervention on the primary outcome (food allergy) and secondary outcomes - asthma (including atopic wheeze), eczema, allergic rhinitis (including aero-allergen sensitization), combined food allergy prevalence (including food sensitization) and the prevalence of combined allergic disease will be known.
**Study Duration**	71 months.
**Study Start Date**	15th January 2008
**Study Recruitment Start**	2nd February 2009
**Final Report Due**	31st July 2014
**Project End Date**	30th September 2014
**Primary Endpoint**	The period prevalence of IgE mediated food allergy to the six intervention foods between one and three years of age in both arms.
**Secondary Endpoints**	***Period (one to three years of age) prevalence food outcomes***<br>The period prevalence of all IgE mediated food allergy between one and three years of age in both arms.<br><br>The period prevalence of all food allergy (IgE and non-IgE mediated) between one and three years of age in both arms.<br><br>The period prevalence of sensitization (defined in Section 3.9) to food between one and three years of age in both arms.<br><br>The total number of foods (of the six intervention foods) to which IgE mediated food allergy has been diagnosed between one and three years of age in both arms amongst children with IgE mediated allergy to one or more of the six intervention foods.<br><br>The total number of foods (any foods) to which IgE mediated food allergy has been diagnosed between one and three years of age in both arms amongst children with IgE mediated allergy to one or more foods.

Cumulative (by three years of age) prevalence food outcomes

The cumulative prevalence of IgE mediated food allergy to the six intervention foods by three years of age.

The cumulative prevalence of all IgE mediated food allergy by three years of age.

The cumulative prevalence of all food allergy (IgE and non-IgE mediated) by three years of age.

The cumulative prevalence of non-IgE mediated food allergy by three years of age.

The cumulative prevalence of sensitization (defined in Section 3.9) to the six foods by three years of age.

The total number of foods (of the six intervention foods) to which IgE mediated food allergy has been diagnosed by three years of age amongst children with IgE mediated allergy to one or more of the six intervention foods.

The total number of foods (any foods) to which IgE mediated food allergy has been diagnosed by three years of age amongst children with IgE mediated allergy to one or more foods.

Other allergic disease outcomes

The point prevalence of eczema at one year and three years of age and cumulative prevalence of eczema by three years of age.

The severity of eczema at one year and three years of age.

The prevalence of allergic rhinitis at three years of age.

The prevalence of inhalant allergen sensitization at one year and at three years of age by skin prick test

The prevalence of inhalant allergen sensitization at one year and at three years of age by specific IgE measurement

The prevalence of the atopic wheeze phenotype at three years of age

Composite allergy outcome

The prevalence of combined allergic disease (a composite of cumulative IgE mediated food allergy to all foods, atopic wheeze phenotype, eczema and allergic rhinitis) at three years of age

The prevalence of combined allergic disease (a composite of cumulative IgE and non-IgE mediated food allergy to all foods, atopic wheeze phenotype, eczema and allergic rhinitis) at three years of age

Safety outcome

Incidence of adverse events and laboratory abnormalities; nutritional evaluations.

Immunological outcomes

Results of cellular and humoral assessments of immune response related to the development of allergy or tolerance to specific allergens (*subject to additional funding*)

Genetic analyses

The association between skin barrier gene defects (such as carriage of the filaggrin skin barrier mutations) and other measures of skin barrier integrity (transepidermal water loss) with all the study outcomes will be assessed.

Inclusion Criteria

1. Pregnant mothers attending their 12/20 week ultrasound scans.
2. Mothers planning on exclusively breast feeding for at least the first 3 months
3. Informed consent obtained from parent or guardian.

Exclusion Criteria

1. Significant antenatal anomaly at 20 week ultrasound scan.
2. Multiple pregnancy
3. Significant congenital disease (enteropathy, congenital heart disease, renal disease)
4. Premature delivery (less than 37 completed weeks gestation)
5. Parents not planning on breast feeding exclusively for at least the first 3 months.
6. Parents planning on moving away from London before their child is three years of age
7. Parents unable to speak and read English
8. Unwillingness or inability to comply with study requirements and procedures.
9. Family intend infant to be on a restricted diet (any of the six intervention foods)

10. ALT or bilirubin >2 times the upper limit of age-related normal value.
11. Urea or creatinine >1.25 times the upper limit of age-related normal value.
12. Platelet count <100,000/ml, haemoglobin <9 g/dl, or investigator-suspected immunocompromise.

Study Intervention

The intervention arm consists of the dietetic controlled introduction of allergenic foods from 3 months of age. Baby rice mixed with breast milk or water will be commenced first, followed by cow's milk based yoghurt. Subsequently egg, wheat, sesame, fish and peanut will be sequentially introduced into the diet in high doses with each food being ingested twice a week achieving a total ingestion of 4 grams or more per week of each food protein by five months of age. Wheat will not be introduced before 4 months of age.

Infants in the intervention arm will be required to consume the allergenic foods until the one year assessment at which point ongoing consumption of all six allergenic foods will be encouraged until the end of the study when at three years of age, subsequent consumption of the allergenic foods will be left to parental choice.

The control arm will follow standard UK Government weaning advice (exclusive breast feeding until around 26 weeks of age) and avoid introducing foods that may cause allergies before six months of age (cow's milk formula, egg, wheat, peanuts, tree nuts, seeds, fish and shell fish).

Glossary of Abbreviations

**BMI**	Body Mass Index
**COMA**	Committee on Medical Aspects of Food Policy
**COT**	Committee On Toxicity of chemicals in food, consumer products and the environment
**CRF**	Case Report Form
**CTCAE**	Common Terminology Criteria for Adverse Events
**DBPCFC**	Double-Blind, Placebo-Controlled Food Challenge
**DH**	Department of Health
**DSMB**	Data Safety Monitoring Board
**FAP**	Facilitated Antigen Presentation
**FFQ**	Food Frequency Questionnaire
**FSA**	Food Standards Agency
**GCP**	Good Clinical Practice
**IDMC**	Independent Data Monitoring Committee
**ISRCTN**	International Standard Randomised Controlled Trial Number Register
**KLH**	Keyhole Limpet Hemocyanin
**MRC**	Medical Research Council
**NCI**	National Cancer Institute
**NIHR CRN**	National Institute for Health Research Clinical Research Network
**P(T)MG**	Project (Trial) Management Group
**POEM**	Patient-Oriented Eczema Measure
**SAE**	Serious Adverse Event
**SPT**	Skin-Prick Test
**SCORAD**	SCORing Atopic Dermatitis
**TEWL**	Trans-Epidermal Water Loss
**UK**	United Kingdom
**UKCRN**	United Kingdom clinical research network
**WHO**	World Health Organization

Contents

1. BACKGROUND 7
1.1 SUMMARY AND RATIONALE FOR TRIAL 7
1.2 AETIOLOGY OF FOOD ALLERGY 7
1.2.1 Increased Prevalence of Food Allergy 7
1.2.2 The State-of-the Art in the Research Area 8
1.2.3 The Scientific Basis for the Work Proposed 9
1.2.4 How this project will advance the state-of-the-art 11
1.3 RATIONALE FOR TRIAL DESIGN 11
1.3.1 General Population 11
1.3.2 Interventional Study 11
1.3.3 Pragmatic Design 11
1.3.4 Justification for Enrolment at 3 Months of Age 13
1.3.5 Justification for Skin Prick Testing Intervention Children Only at 3 month Enrolment Visit 16
1.3.6 Justification for Administering Cow's Milk Protein as a Solid (Yoghurt) 16
1.3.7 Justification for Reviewing All Infants at One Year of Age 18
1.3.8 Justification for the Three Year Assessment as the Principal Outcome 18
1.3.9 Justification for the Challenging All Skin Prick Positive Children at One and Three Year Assessments 18
1.4 RATIONALE FOR IMMUNOLOGICAL ASSESSMENTS 20
1.5 KNOWN AND POTENTIAL RISKS 20
1.5.1 Early Weaning Group 20
1.5.2 Control Group 21
1.5.3 Both Groups 21
2. SCIENTIFIC OBJECTIVES 22
2.1 OBJECTIVE ONE 22
2.2 OBJECTIVE TWO 22
2.3 OBJECTIVE THREE 23
3. STUDY DESIGN 24
3.1 OVERALL STUDY DESIGN 24
3.1.1 Population 25
3.1.2 Backup Recruitment Strategies 25
3.1.3 Maternal participation 25
3.2 INFANT ENROLMENT - SCREENING FOR ALLERGY AT 3 MONTHS 27
3.3 PRIMARY ENDPOINT 28
3.3.1 Definition of IgE mediated food allergy for primary endpoint 28
3.4 SECONDARY ENDPOINTS 28
3.5 DETERMINATION OF FOOD ALLERGY – UNSCHEDULED CLINIC VISITS 30
3.6 DETERMINATION OF FOOD ALLERGY – 1 YEAR ASSESSMENT 33
3.7 DETERMINATION OF FOOD ALLERGY – 3 YEAR ASSESSMENT 34
3.8 DETERMINATION OF SENSITIZATION 34

3.9 STUDY DEFINITIONS....34
3.9.1 Allergic disease definitions....34
3.9.2 Breast feeding definitions....35
3.10 STOPPING RULES....36
3.10.1 Ongoing Review....36
3.10.2 Review of Specific Adverse Events....36
3.11 STUDY DURATION....36
4. ELIGIBILITY....37
4.1 INCLUSION CRITERIA....37
4.2 EXCLUSION CRITERIA....37
4.3 PREMATURE TERMINATION....37
4.3.1 Premature Termination of Trial Interventions....37
4.3.2 Premature Termination from the Trial....37
5. STUDY INTERVENTION....38
5.1 ALLERGENIC DIET ADMINISTRATION OR AVOIDANCE....38
5.2 ASSESSMENT OF COMPLIANCE WITH STUDY INTERVENTION....39
5.3 MODIFICATION OR DISCONTINUATION OF STUDY TREATMENT....39
6. STUDY PROCEDURES....40
6.1 VISIT WINDOWS....40
6.2 GENERAL ASSESSMENTS....40
6.3 QUESTIONNAIRE SCHEDULE....41
6.4 LABORATORY ASSESSMENTS....41
6.5 ALLERGY ASSESSMENTS....42
6.5.1 Allergens Assessed....42
6.5.2 Skin Prick Testing: Procedures and Interpretation....42
6.6 FOOD CHALLENGES....43
6.6.1 Scheduled Challenges....43
6.6.2 Unscheduled Challenges....43
6.6.3 Repeat Challenges....43
6.6.4 Challenges for non-intervention foods....43
6.6.5 Procedure for challenges for suspected IgE mediated food allergy....43
6.6.6 Procedure for challenges for suspected non-IgE mediated food allergy....47
7. TOLERANCE ASSAYS....48
7.1 FROZEN PBMC T-CELL ASSAY....48
7.1.1 Overview....48
7.1.2 Gene Expression Profiling....48
7.1.3 Secreted Cytokines....48
7.1.4 Antigen-specific Proliferative and Cytokine Responses....48
7.1.5 Regulatory T cells....48
7.2 PLASMA ALLERGEN-SPECIFIC IGG AND IGE....49
7.2.1 ELISA-based Techniques....49
7.2.2 Facilitated Antigen Presentation Inhibition....49

7.3 WHOLE BLOOD DNA–HLA GENOTYPES....49
8. ADVERSE EVENTS....50
8.1 OVERVIEW....50
8.2 INDEPENDENT DATA MONITORING COMMITTEE (IDMC)....50
8.2.1 Terms of reference....50
8.2.2 Membership....50
8.2.3 Guidance notes....50
8.3 DEFINITIONS....51
8.3.1 Adverse Event....51
8.3.2 Serious Adverse Event....51
8.3.3 Unexpected Adverse Event....52
8.4 COLLECTING ADVERSE EVENTS....52
8.4.1 Methods of Collection....52
8.4.2 Collecting Serious Adverse Events....52
8.4.3 Recording Adverse Events....52
8.4.4 Recording Serious Adverse Events....52
8.5 GRADING AND ATTRIBUTION OF ADVERSE EVENTS....52
8.5.1 Grading Criteria....52
8.5.2 Attribution Definitions....53
8.6 REPORTING ADVERSE/SERIOUS ADVERSE EVENTS....53
8.6.1 Reporting Timeline....53
8.6.2 Options for Reporting Serious Adverse Events....53
9. STATISTICS....55
9.1 ANALYSIS SAMPLES....55
9.2 ANALYSIS OF ENDPOINTS....55
9.2.1 Analysis of Primary Endpoint....55
9.2.2 Analysis of Secondary Endpoints....55
9.2.3 Additional Analyses....56
9.3 SAMPLE SIZE....56
9.4 LOSS TO FOLLOW-UP....56
9.5 RANDOMIZATION, STRATIFICATION, AND BLINDING....56
9.6 REPORTING DEVIATIONS FROM THE ORIGINAL STATISTICAL PLAN....56
10. IDENTIFICATION AND ACCESS TO SOURCE DATA....57
10.1 IDENTIFYING SOURCE DATA....57
10.2 PERMITTING ACCESS TO SOURCE DATA....57
11. QUALITY CONTROL AND QUALITY ASSURANCE....58
11.1 MANAGEMENT STRUCTURE....58
11.1.1 Trial Steering Committee....59
11.1.2 Project (Trial) Management Group....60
11.1.3 Independent Data Monitoring Committee....60
11.1.4 External Scientific Advisors....60
11.2 DATA HANDLING....61

12. ETHICAL CONSIDERATIONS AND COMPLIANCE WITH GOOD CLINICAL PRACTICE 62
12.1 STATEMENT OF COMPLIANCE 62
12.2 INFORMED CONSENT AND ASSENT 62
12.3 PRIVACY AND CONFIDENTIALITY 62
13. PUBLICATION POLICY 62
14. REFERENCES 63

APPENDIX 1. STUDY OVERVIEW CHART 67
APPENDIX 2. STUDY GANTT CHART 68
APPENDIX 3. SCHEDULE OF EVENTS 69

List of Tables

TABLE 1. FREQUENCY OF ALLERGENIC FOOD CONSUMPTION IN UK INFANTS AGED 8-10 MONTHS 12

TABLE 2. PREVALENCE (%) OF EXCLUSIVE BREASTFEEDING BY COUNTRY (2005) 13

TABLE 3. DURATION OF EXCLUSIVE BREASTFEEDING AMONG MOTHERS WHO FED EXCLUSIVELY AT BIRTH BY COUNTRY (2005) 14

TABLE 4. DISTRIBUTION OF SKIN PRICK RESULTS IN FAIR COHORT AT 1 AND 3 YEARS OF AGE 19

TABLE 5. LIKELY DISTRIBUTION OF SKIN PRICK RESULTS AT 1 AND 3 YEARS OF AGE BASED ON EXTRAPOLATING FAIR DATA TO THE EAT STUDY 19

TABLE 6. LIKELY NUMBER OF FOOD CHALLENGES REQUIRED AT 1 AND 3 YEARS OF AGE BASED ON EXTRAPOLATING FAIR DATA TO THE EAT STUDY USING TWO THRESHOLDS: ALL POSITIVE RESULTS AND >2MM..........20

TABLE 7. CRITERIA THAT DEFINE SELECTED INFANT FEEDING PRACTICES..........35

TABLE 8. CRITERIA FOR DETERMINING THE OUTCOME OF FOOD CHALLENGE..........46

TABLE 9. NCI-CTCAE ATTRIBUTION OF ADVERSE EVENTS..........53

List of Figures

FIGURE 1. PROTOCOL DEFINED EGG ALLERGY AT ENROLMENT INTO LEAP STUDY. 15

FIGURE 2. SENSITISATION TO ALLERGENIC FOODS (IGE>0.35KU/L) AT ENROLMENT ONTO THE LEAP STUDY. 15

FIGURE 3. THE MEDIAN BREASTFEEDING FREQUENCY IN THE SOLIDS AND FORMULA GROUPS. 16

FIGURE 4. THE MEDIAN BREASTFEEDING FREQUENCY BY AGE AT INTRODUCTION OF SOLIDS IN THE SOLIDS GROUP AND OF FORMULA IN THE FORMULA GROUP. 17

FIGURE 5. OVERALL STUDY DESIGN 24

FIGURE 6. THREE MONTH SCREENING VISIT 27

FIGURE 7. ASSESSMENTS TO BE PERFORMED AT UNSCHEDULED CLINIC VISITS- IGE MEDIATED FOOD ALLERGY 31

FIGURE 8. ASSESSMENTS TO BE PERFORMED AT UNSCHEDULED CLINIC VISITS- NON-IGE MEDIATED FOOD ALLERGY 32

FIGURE 9. ONE YEAR AND THREE YEAR ASSESSMENT VISITS – IGE MEDIATED FOOD ALLERGY 33

FIGURE 10. MANAGEMENT STRUCTURE 58

1. BACKGROUND

1.1 SUMMARY AND RATIONALE FOR TRIAL

In the United Kingdom (UK) 6% of children will develop food Allergies (FA).[3] The one year prevalence of allergic disease amongst 6-7 years old children in the UK is: 16.0% eczema, 20.9% asthma and 10.1% allergic rhinitis (AR).[4] The World Health Organization (WHO) Global Strategy for Infant and Young Child Feeding,[5] endorsed by the UK Goverment,[6] recommends exclusive breast feeding (EBF) for the first 6 months. The UK Government weaning information leaflet for parents adopts a more pragmatic target of around 6 months exclusive breast feeding.[7] It also states that if a mother decides to wean before six months there are some foods that should be avoided as they may cause allergies including: "wheat-based foods and other foods containing gluten (e.g. bread, rusks, some breakfast cereals), eggs, fish, shellfish, nuts (and) seeds." There is little evidence that this reduces allergic disease. There are conflicting results of interventional trials of delayed weaning to reduce FA and atopy. Animal models and preliminary human data suggest that high dose oral tolerance to food proteins in early life may prevent allergic sensitization. Three separate studies show that prolonged EBF is a risk factor for developing atopic disease.[8-10] Since the 1970s allergic disease has increased significantly: asthma prevalence doubled and eczema prevalence tripled between 1973 and 1988 in a study in South Wales.[11] These increases have coincided with a two-thirds reduction in early weaning.[12] Thus it may be that delayed weaning has promoted FA and even other atopic diseases. We propose a randomized controlled study in infants to determine whether early weaning and exposure to food allergens (from 3 months of age) prevents the development of FA, eczema, asthma and AR. This study will provide an informed basis for future weaning practices in both atopic and normal infants.

1.2 AETIOLOGY OF FOOD ALLERGY

1.2.1 Increased Prevalence of Food Allergy

Food allergy develops in approximately 6% of children in the UK[3] and the prevalence for particular allergens (peanut) is increasing.[13] The reason for this increase remains unclear. Interventions to prevent food allergy have involved intervening with one or more of the following: antenatal environment (maternal diet); maternal lactation (duration of exclusive breastfeeding and maternal diet whilst lactating); infant diet (timing of introduction of mixed feeding and number and types of foods introduced).

With regard to the introduction of mixed feeding there has been a significant trend since 1975 towards later introduction. The proportion of infants given solids by 8 weeks of age has decreased: 49% in 1975, 24% in 1980 & 1985, and 19% in 1990.[12] It is a simple ecological observation that this decrease to a third of what it was has coincided with up to a three-fold increase in allergy in children.[11] This change has been compounded by a number of different bodies recommending delayed introduction of foods. The American Academy of Pediatrics recommended avoiding solids until six months of age, cow's milk until 1 year of age, egg to 2 years and peanuts, tree nuts and fish to 3 years of age.[14] These guildelines have only recently been rescinded.[15] The European Society for Paediatric Gastroenterology, Hepatology and Nutrition (ESPGHAN) Committee on Nutrition has also recently revised its recommendations.[16] It states that "complementary feeding (i.e., solid foods and liquids other than breast milk or infant formula and follow-on formula) should not be introduced before 17 weeks and not later than 26 weeks." In the UK the Government has published a paper entitled "Infant Feeding Recommendation" which summarises the latest advice with regard to the introduction of solid food.[6] The paper states that the UK Government recommendation on feeding infants is that "exclusive breastfeeding is recommended for the first six months (26 weeks) of an infant's life." The paper also states that: "Certain foods are more likely to upset a baby or cause an allergic reaction than other foods. These foods should not be introduced before six months (COMA

1994).” However, whilst the section on the management of food allergy in the Committee on Medical Aspects of Food Policy (COMA) report contains a specific sentence on not introducing allergenic foods before 6 months of age, it is not referenced.[12] The WHO’s systematic review of the optimal duration of exclusive breastfeeding found no significant reduction in risk of atopic eczema, asthma, or other atopic outcomes amongst those exclusively breast fed for 6 months compared with those exclusively breastfed for 3-4 months followed by mixed feeding.[17] In theory, deferring introduction of an allergenic food prevents a reaction. However, this doesn’t allow for accidental exposures, the possibility of cross reactivity with other foods and possible sensitization by other means (cutaneous or via continued breast milk exposure). It may simply defer presentation of an allergy.

1.2.2 The State-of-the Art in the Research Area

Interventions to reduce food allergy

The outcome of studies using the interventions mentioned above has been generally disappointing. Removing egg and cow’s milk in the 3rd trimester of pregnancy made no difference in two studies.[18, 19] Whilst one out of two studies showed a short term reduction in eczema with avoidance of egg, cow’s milk and fish during lactation, there was no difference in any other outcomes.[20, 21] Of the two studies adopting a multi-intervention approach (pregnancy and lactation exclusion of allergenic foods, soya/casein hydrolysate for 6 months in non breast fed infants and delayed introduction of cow’s milk and solids for 6-11 months) only one achieved a reduced prevalence of food allergy and cow’s milk sensitisation.[22, 23] Data on interventions specifically focusing on introduction of solids is limited. One randomized study showed no difference in fish and citrus allergy by 3 years of age between children with fish introduced early or late (after 1 year of age).[24] A prospective non-randomized study found a reduction in food allergy at 1 year of age in the group fed solids after 6 months compared to before 4 months, but there was no difference in eczema or skin prick sensitization to a panel of food allergens at 5 years of age.[25]

Solids introduction and eczema

Fergusson reported a 2.9 times greater risk of chronic/recurrent eczema amongst children fed 4 or more solids before 4 months of age compared with those not fed solids before 4 months of age. The difference was still apparent at 10 years of age. However, as with virtually all the studies published to date the study, whilst being prospective, was observational only and did not involve a randomized intervention.[26] Whilst this study is often quoted as evidence that early introduction of solids induces allergic disease, a more recent Australian study found no significant association between the duration of breastfeeding or timing of introduction of solid foods and protection against asthma or other allergic disease in 516 children evaluated at age 5 years. However, breastfeeding for 6 months or more and introduction of solid foods after 3 months were both associated with an increased risk of atopy at age 5 years.[27] This is consistent with another recent study. Sariachvili *et al* undertook a nested case-control study in the PIPO cohort (Prospective Cohort on the Influence of Perinatal Factors on the Occurrence of Asthma and Allergies). Early introduction (within the first 4 months) of solid foods was inversely associated with eczema up to 4 years of age (OR_{adj} 0.5, 95% CI 0.3-0.7). Moreover, early exposure to solid foods was associated with a reduced risk for eczema only among children with allergic parents (OR_{adj} 0.4, 95% CI 0.2-0.6), whereas no significant effect was found among children with non-allergic parents (OR_{adj} 0.7, 95% CI 0.4-1.3).[28]

Possible risks of delaying solid introduction

Three observational studies have found that delayed introduction of solids was related to an increased risk of asthma and eczema.[8-10] Reverse causality has been proposed as an explanation. However Zutavern found no evidence of feeding practices playing a different role in the development of asthma and eczema with respect to: mother’s opinion of child’s eczema status at year one; and parental allergy, asthma, and atopy status.[10] Similarly, a recent study found that the delayed introduction of cereal grains (after the age of 6 month) was associated with an increased risk of wheat allergy.[29] Related to this, an EU funded Framework 6 Project has been commenced which aims to determine whether the early introduction of wheat into the diet prevents the

development of coeliac disease and induces tolerance (http://www.preventceliacdisease.com). In this randomized controlled trial the intervention arm will receive daily small amounts of gluten during the period of breastfeeding from the age of 4 months (apparently each dose being 3% of what a normal 6 month old would consume). The control arm receives a milk sugar powder placebo during the period of breastfeeding from the age of 4 months.
Data has also emerged with regard to fish consumption with a study finding that introducing fish before 9 months of age was associated with a reduction in allergic disease, specifically eczema.[30] Similarly, a recent Dutch study found that more delay in introduction of cow milk products was associated with a higher risk for eczema. In addition, a delayed introduction of other food products was associated with an increased risk for atopy development at the age of 2 years. Exclusion of infants with early symptoms of eczema and recurrent wheeze (to avoid reverse causation) did not essentially change the results. The authors concluded that delaying the introduction of cow milk or other food products may not be favourable in preventing the development of atopy.[31]

1.2.3 The Scientific Basis for the Work Proposed

Oral tolerance

There is data from a variety of sources to suggest that delaying the introduction of allergenic foods might not be the right approach. Clinical observations from countries in Southeast Asia and Africa, where high amounts of peanuts are consumed in different snack forms during infancy, suggest a low rate of peanut allergy. As these differences could be due to genetics, we have examined these geographical variations more carefully by comparing the prevalence of peanut allergy in Jewish children in the UK and Israel.[32] The prevalence of peanut allergy in the UK was 1.85%, and the prevalence in Israel was 0.17% ($P < .001$). Despite accounting for atopy, the adjusted risk ratio for peanut allergy between countries was 9.8 (95% CI, 3.1-30.5) in primary school children. Peanut is introduced earlier and is eaten more frequently and in larger quantities in Israel than in the UK. The median monthly consumption of peanut in Israeli infants aged 8 to 14 months is 7.1 g of peanut protein, and it is 0 g in the UK ($P < .001$). The median number of times peanut is eaten per month was 8 in Israel and 0 in the UK ($P < .0001$).

One study of oral tolerance induction in adults showed that oral intake of keyhole limpet hemocyanin (KLH) results in immunological tolerance to KLH antigen.[33] Two studies have attempted to induce tolerance to food allergens.[34, 35] The first was conducted in patients who already had established milk allergy. The result of this study was promising: 71% of highly allergic children were able to tolerate a daily intake of 200 mls of milk after treatment.[34] However, because this was an uncontrolled study, the possibility that these children would have shown spontaneous resolutions cannot be discounted. More recently four children with peanut allergy underwent desensitisation and were subsequently able to tolerate between 10 and 12 peanuts.[35]

There is some evidence that oral exposure to nickel results in tolerance. Numerous studies, both prospective and retrospective, show that early cutaneous exposure to jewelry, particularly through ear piercing, is a risk factor for the development of contact dermatitis to nickel. Three independent studies,[36-38] including one prospective birth cohort study, show that the early application of orthodontic braces made of nickel strongly protects against the development of contact dermatitis to nickel (in one study there was an odds ratio of 0.07). Indeed, after the insertion of fixed orthodontic appliances, the level of nickel both in the saliva and serum of individuals increases significantly, which is thought to result in oral tolerance. Similarly, patients exposed to pancreatic extract by inhalation or contact develop IgE-mediated allergic reactions, whereas patients exposed to this extract by oral route do not.[39]

Animal data

Animal data is also supportive of the hypothesis. In most experimental animals a state of specific systemic hypo-responsiveness is induced by single or multiple intestinal antigen exposure.[40] A PubMed search for publications containing all the text words: immune, tolerance, single, dose, oral and administration, yielded 60 documents (search undertaken 25.02.09). These documents include a review article describing the principle of tolerance induction and its relationship with

dosage and frequency of timing in animal models and this confirms that a single oral dose of antigen is sufficient to induce tolerance.[41] This phenomenon has been demonstrated for different antigens in different experimental models, but the resulting data are consistent: they uniformly show that a single dose of oral protein administration effectively causes immunological tolerance and prevents the expression of related clinical disease. With regard to timing, the animal data suggests that continuous feeding beyond the critical neonatal period leads to induction of tolerance.[40]

Oral tolerance induction in animal models is most potent in its effects on delayed type I hypersensitivity responses; prevention of antibody responses through induction of oral tolerance is less consistent. However, numerous publications point to the fact that in mice a single dose of food allergen (beta-lactoglobulin, ovalbumin, peanut) is particularly effective in preventing the development of subsequent IgE-mediated responses. A recent study showed that naïve mice orally tolerized to beta-lactoglobulin were unable to mount significant IgE responses when they were subsequently sensitized with beta-lactoglobulin injected along with alum intraperitoneally.[42] Similarly, there were no significant T-cell responses to beta-lactoglobulin in the pretolerized animals.

Strid and colleagues fed mice a single intragastric feed of defatted peanut flour at doses varying from 0.2 to 100 mg per mouse.[43] Seven days after the feed, the mice were immunized with 100 µg of peanut antigen emulsified with Complete Freunds Adjuvant. Three weeks later, the mice were given a recall immunization with 100 µg of antigen. The mice were assayed for T-cell proliferation to peanut, cytokine production, delayed-type hypersensitivity responses, and antibody responses. Tolerizing doses of 100 mg of peanut protein resulted in significant reduction of delayed-type hypersensitivity responses and inhibition of proliferative responses to peanut. Mice tolerized to 100 mg of peanut protein showed significantly reduced interferon gamma and IL4 production. Specific IgE responses to peanut following sensitization were almost completely prevented by the single tolerizing dose. However, very low tolerizing doses of peanut, i.e., those below 2 mg per animal, resulted in enhanced delayed-type hypersensitivity responses, T-cell proliferative responses, cytokine production, and IgE production. Doses between 2 and 20 mg of peanut protein induced no difference between the T- and B-cell responses as compared to sham-tolerized animals. Tolerance to peanut was only achieved at doses of 100 mg per animal. Oral tolerance to peanut was shown to be antigen specific. Tolerizing doses of peanut did not promote tolerance to ovalbumin and vice versa.

Exclusive breast feeding and other disease outcomes

The WHO systematic review of the optimal duration of exclusive breast feeding stated that: "Based primarily on an observational analysis of a large randomized trial in Belarus, infants who continue exclusive breastfeeding for 6 months or more appear to have a significantly reduced risk of one or more episodes of gastrointestinal infection".[17] However, the Millennium cohort, a UK based longitudinal study of 15,980 infants has recently reported that the age of introduction of solids had no effect on risk of hospitalization for diarrhoea or lower respiratory tract infection.[44]

Summary

Our study group is currently undertaking a randomized controlled trial of the early introduction of peanuts into the diet of high risk infants to induce tolerance: Learning Early About Peanut (LEAP) Study. The EAT Study is a randomized controlled trial of the early introduction of allergenic foods in a normal population. Recruitment for this study commences after the completion of recruitment of the peanut intervention study. Participants will be encouraged to breast feed exclusively until three months of age when half of the infants will be randomized to introduce a number of allergenic foods into the diet under dietetic direction. Participants will be followed up until 3 years of age by which point the impact of the intervention on the primary outcome (food allergy) and secondary outcomes (eczema, asthma, allergic rhinitis, allergen sensitization) will be known. The scale of this study will provide a unique opportunity as a platform on which to seek additional funding for a diversity of further research. This will include further nutritional and metabolic work. The proposal is also unique in that there have been no randomized interventional studies looking at the effects of early versus late weaning onto

allergenic foods in general. This study will allow the safety and possible beneficial effects on growth and normal development to be assessed. The research project will be physically located in a purpose built six bedded paediatric allergy clinical trials unit housed in the new Evelina children's hospital at Guy's and St Thomas' NHS Foundation Trust. This unit forms part of the MRC & Asthma UK Centre in Allergic Mechanisms of Asthma headed by Professor Tak Lee. This is the first disease specific centre of its kind in the UK to address allergic mechanisms. The centre benefits from a vast collaborative network of principal investigators experienced in both basic laboratory and clinical research.

1.2.4 How this project will advance the state-of-the-art

A positive outcome with the successful induction of tolerance may have implications for weaning policy both nationally and internationally. In addition, a reduction in the number of children with allergies will, in turn, have a significant cost benefit for the administration of healthcare.

1.3 RATIONALE FOR TRIAL DESIGN

1.3.1 General Population

As with any interventional study enrolling a "general" population, we will be subject to a participation bias. In every such study, individuals with a vested interest in the study (a family or personal history of the condition in question) are more likely to participate. This is absolutely unavoidable. We will, however, ensure that all mothers are approached to participate in the study and our information leaflets will stress the importance of all mother's participating. We will ask mother's who decline to participate if they have a personal or family history of allergic disease so we can quantify the degree of participation bias compared with those who enrol.
Conversely, if our population does transpire to be at a higher risk of food allergy than the general population this will have the effect of increasing the power of the study.

1.3.2 Interventional Study

There is considerable confusion in the field of weaning and its effect on allergic disease and child health: Remarkably, there has not been a single large randomised controlled interventional study to address this issue. This is the first such study. We will determine the health and nutritional consequences of introducing foods early and whether this measure is effective in reducing allergic diseases. This is consistent with the WHO recommendation that: "Large randomized trials are recommended to rule out small effects on growth and to confirm the reported health benefits of exclusive breastfeeding for six months or beyond".[17] With conflicting data and health policies in different parts of the world, there is an insufficient evidence base with which to advise parents with regard to allergy prevention and weaning.

1.3.3 Pragmatic Design

This is a pragmatic study consisting of an active intervention versus the status quo which is an incomplete following of the current UK Government weaning recommendations.

Current UK Government weaning recommendations:
UK Government weaning guidelines current state: "If you decide to wean at any time before six months, there are some foods that should be avoided as they may cause allergies... These include wheat-based foods and other foods containing gluten (e.g. bread, rusks, some breakfast cereals), eggs, fish, shellfish, nuts *and* seeds."[7] The guidance also states with regard to high risk families: "Babies are more likely to develop allergies if there is a family history of eczema, asthma or hay fever. For these families, exclusive breastfeeding is particularly recommended for the first six months. Introduce the foods that commonly cause allergies (milk, eggs, wheat, nuts, seeds, fish and shellfish) one at a time so that you can spot any reaction, but don't introduce any of these foods before six months."[7] Currently these infants are also advised to follow the Committee On Toxicity of chemicals in food, consumer products and the environment (COT) recommendations on avoiding peanuts: "Avoid giving peanuts and foods containing peanut products, e.g. peanut

butter or groundnut oil, until your child is three years old."[7] However, the Government are likely to revise these recommendations shortly following on from a recent review of the evidence by the COT and a recommendation to the Health Minster by the FSA board.

Actual allergenic food weaning practice:
Although the UK Government guidelines do not stipulate delaying the introduction of allergenic foods beyond six months of age (with the exception of the COT recommendations for peanut in high risk infants), we know that the current weaning regimen consists of little allergenic food consumption in the first year of life (with the exception of cow's milk products and wheat). Data from the Infant Feeding Survey 2005 show the frequency with which mothers were giving their infants allergenic foods at Stage 3 of the survey (when the infants were between 8 and 10 months of age) (Table 1).[45]

Table 1. Frequency of allergenic food consumption in UK infants aged 8-10 months

Food	Percentage giving food:			
	1/day or more	1-6 times a week	<1/week or never	3/week or more
Cheese, yoghurt, fromage frais	64	26	9	85
Breakfast cereals	82	8	9	88
Bread	36	38	25	58
Eggs	2	23	76	6
Fish (incl. Tuna)	3	45	52	18
Nuts (incl. Ground nuts)	<1	1	99	<1

In the Infant Feeding Survey 2005, among mothers who cited at least one food avoidance by Stage 3 of the Survey, the most common reason was a concern about allergies (43%, a rise of 8 percentage points since the 2000 survey). Avoidance of specific foods as ingredients was common. For egg, 24% avoided it because they considered it harmful and 44% because of concerns about allergies. For dairy products, 17% were concerned about harm, 50% allergies and 8% concerned with eczema. For nuts 33% were concerned about harm and 70% allergies (mothers could report more than one concern). Thus our study will entail a comparison between early high dose introduction versus little, if any, early allergen exposure (with the exception of cow's milk and wheat).

Compliance with UK Government weaning guidelines amongst the control arm
To participate in the study all mothers will have had to exclusively breastfeed for three months prior to randomization. The current WHO definition of exclusive breast feeding is given in Section 3.9.2. However, it will be deemed acceptable for mothers to have given boiled then cooled water, as well as oral rehydration solution, without being considered to have ceased exclusive breastfeeding as this will not have any immunomodulatory effect on the infant.
A pragmatic approach has been taken to compliance with the UK Government weaning guidelines in the control arm with regard to the per protocol analysis (Section 9.1). This is because we anticipate that despite support to continue exclusive breastfeeding, some mothers in the control arm will choose to introduce cow's milk based formula before 6 months of age. In terms of the design of the study, we believe there is no value in trying to achieve a modification of behaviour to such a degree of compliance that it could not be expected to be achieved as a public health measure. In other words there is no point trying to ensure a regimen of complete avoidance if this cannot be realistically carried out in real life outside of the setting of a study intervention.

Objective monitoring of actual food consumption
High food specific IgG levels are known to correlate with increasing consumption of the specific food (Vance et al., 2004; Vance et al., 2005). Therefore a further way of objectively validating the dietary allergenic food intake will involve specific IgG measurement to food allergens. We

will be able to measure IgG levels on stored serum samples (subject to further funding). This allows us to objectively validate the dietary allergenic food intake of the children in both arms of the study and thus will be done on the samples obtained from the whole cohort at the one and three year assessments.

1.3.4 Justification for Enrolment at 3 Months of Age

There are several reasons why it has been decided to enroll infants onto the study at 3 months of age rather than later.

Exclusive breast feeding rates
The Infant Feeding Survey 2005 gave the following historical background to the government's policy on breastfeeding:

"Due to *the* body of evidence on the health benefits of breastfeeding, government policy in the United Kingdom has consistently supported breastfeeding as the best way of ensuring a healthy start for infants. In 1974 a COMA Working Party was set up to review infant feeding practices in the United Kingdom. The recommendations of this Working Party were that all mothers should be encouraged to breastfeed and that mothers should be discouraged from introducing solid foods before four months old. Subsequent reports throughout the 1980s and 1990s continued to endorse these broad recommendations. During this time the infant feeding recommendations in the United Kingdom were broadly in line with the guidance from the World Health Organisation (WHO), which in the 1990 Innocenti Declaration recommended that all infants should be fed exclusively on breast milk from birth up to 4-6 months of age. Early in 2000, the WHO commissioned a systematic review of the published scientific literature on the optimal duration of exclusive breastfeeding. As a result of this review, the WHO revised its guidance to recommend exclusive breastfeeding for the first six months of an infant's life. This revised guidance was adopted by the United Kingdom Health Departments from 2003 onwards."[45]

Thus the UK Government recommends that all mothers should exclusively breastfeed until 6 months of age.[6] However, actual current UK Government weaning literature adopts a more pragmatic approach and encourages mothers to aim for "around" six months exclusive breastfeeding.[7]

Exclusive breastfeeding rates in practice
However, the Infant Feeding Survey 2005 provides clear data which shows that in practice, few mothers achieve either the strict or pragmatic target (Table 2).[45]

Table 2. Prevalence (%) of exclusive breastfeeding by country (2005)

	England	Wales	Scotland	N Ireland	UK
Birth	66	58	61	55	65
1 week	46	38	42	35	45
2 weeks	39	32	37	31	38
3 weeks	34	28	32	25	33
4 weeks	29	21	25	20	28
6 weeks	22	15	19	13	21
2 months (8 weeks)	18	12	17	11	18
3 months (13 weeks)	14	9	12	8	13
4 months (17 weeks)	8	4	6	4	7
5 months (21 weeks)	3	2	3	2	3
6 months (26 weeks)	<1	<1	<1	<1	<1

The figures are only slightly better when restricted to mothers who commenced feeding exclusively at their infants' birth. (Table 3)

Table 3. Duration of exclusive breastfeeding among mothers who fed exclusively at birth by country (2005)

	England	Wales	Scotland	N Ireland	UK
Birth	100	100	100	100	100
1 week	69	66	69	64	69
2 weeks	60	55	60	56	59
3 weeks	52	48	51	46	51
4 weeks	44	37	41	37	43
6 weeks	33	26	31	24	32
2 months (8 weeks)	28	21	28	21	27
3 months (13 weeks)	21	16	20	15	21
4 months (17 weeks)	12	7	10	8	12
5 months (21 weeks)	5	3	4	4	5
6 months (26 weeks)	1	<1	1	<1	<1

Thus by stating in the EAT study eligibility criteria that mothers had to have achieved four months of exclusive breast feeding before being eligible to participate would reduce the number of eligible women by 43% (England 14% to 8% Table 2).

Actual age of introduction of solids:
The actual mean age of introduction of solids in the UK in the Millennium Cohort Study was 3.8 months.[44] This figure is almost identical to that observed in the Infant Feeding Survey 2005 where 51% of infants were reported to have received complementary foods before 4 months of age. Thus our age of introduction of complementary foods in the intervention arm of the EAT study reflects what the majority of parents are doing with their infants in reality.

No evidence of harm from solid introduction from 3 months of age:
Concerns about early introduction of solids in studies undertaken in the 1970's related to two issues: excessive dietary solute load from formula milk and/or solids; and excessive weight gain. One study from 1973 looking at the former found that infants fed artificial milk formula alone or solids and artificial milk formula had higher mean plasma osmolality and a greater percentage of values in the hyperosmolar range than infants who were breast fed.[46] However, the "healthy" group in this comparison were infants of between 1 and 3 months of age who were being breast fed. In the EAT study all infants will be receiving three complete months of exclusive breast feeding. A classic study looking at the latter (cited 150 times) found that artificially fed 6 week old infants in the Sheffield region were heavier than would be predicted from their birth weights and showed a greatly increased incidence of excessive weight gain.[47] Again, the infants of concern were receiving artificial feeds much earlier than the age at which solids will be introduced in our study.

Early occurrence of sensitization to allergenic foods in infants – the LEAP experience
Emerging data from the LEAP study reveals that infants are sensitized far earlier than previously realized. At the point at which 547 infants had been enrolled onto the study, of 115 infants under 6 months of age otherwise eligible to participate, 12 (10.4%) had had to be rejected as they were already too significantly sensitized to peanut (>4mm skin prick test result).

The percentage of infants participating in the LEAP study with protocol defined egg allergy is shown in Figure 1. None of the infants under six months will have actually started consuming egg at this point and yet approaching half have egg allergy.

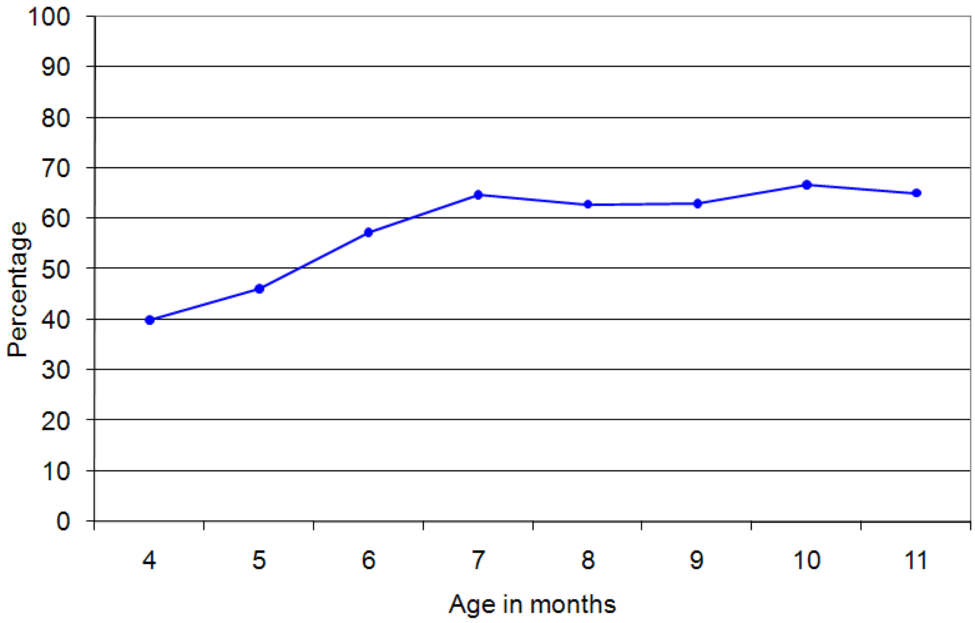

Figure 1. Protocol defined egg allergy at enrolment into LEAP study.

The results of sensitisation rates on specific IgE measurements at enrolment to three of the major food allergens: egg, peanut and cow's milk are shown in Figure 2. 22% of 4 month olds are sensitised to peanut with IgE levels greater than 0.35 KU/L. However, the figure for any specific IgE to peanut is even higher with 40% of 4 month olds having levels greater than 0.1 KU/L).

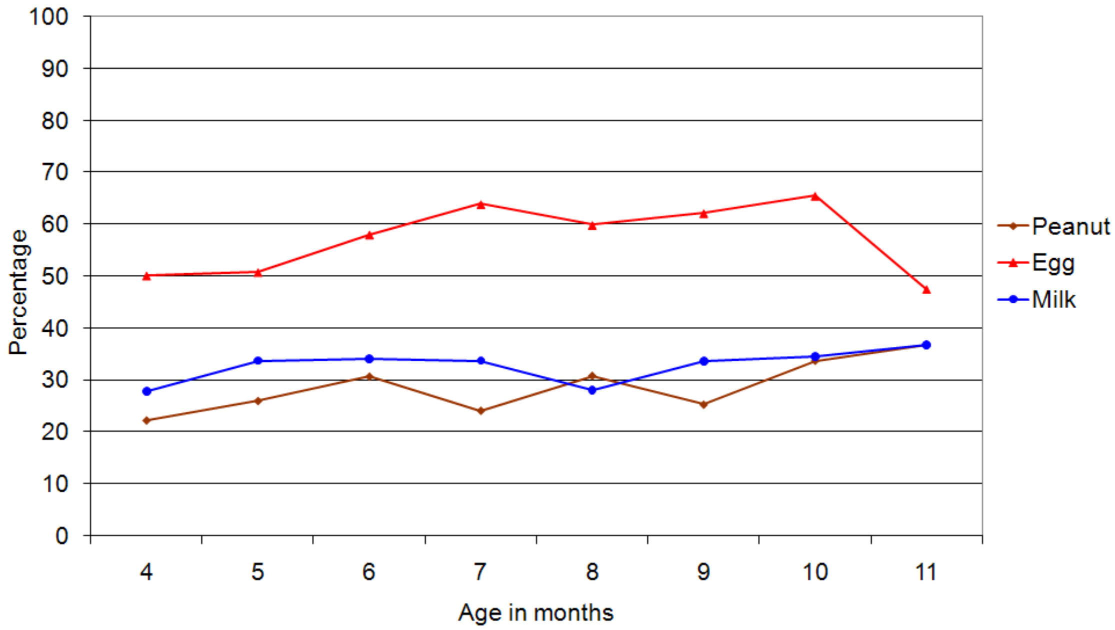

Figure 2. Sensitisation to allergenic foods (IgE>0.35KU/L) at enrolment onto the LEAP Study.

1.3.5 Justification for Skin Prick Testing Intervention Children Only at 3 month Enrolment Visit

The aim is for mothers in the control arm to wean their children in as similar a manner as possible to what they would have done if they were not participating in the study. The principle UK Government advice a mother would receive with regard to weaning her infant is contained in the leaflet mentioned previously. We will ensure all mothers in the control arm are supplied with this leaflet and that the study dieticians provide support with regard to implementing its recommendations.

Skin prick testing the control children would be very likely to change the mothers weaning behaviour significantly. Similarly, whilst blood will be taken from all infants at 3 months of age, specific IgE levels to the six intervention foods will not be measured until the child reaches the end of the study. This ensures that no ethical dilemma is posed by being in possession of information about an individual child's sensitisation which is not being shared with the parent. Just as with the skin prick test results, a mother would not have specific IgE measurements available to influence her weaning regimen if she were not participating in the study.

1.3.6 Justification for Administering Cow's Milk Protein as a Solid (Yoghurt)

In the EAT study, the decision has been taken to give the cow's milk protein exposure as yoghurt rather than cow's milk based formula. The reason for this relates to the differential effect of solid introduction versus formula introduction on pattern and duration of breastfeeding.[48] Hörnell *et al* showed that the introduction of solids was associated with no or minor changes in breastfeeding frequency and suckling duration. Breastfeeding frequency remained constant the first month after the introduction and then declined slowly, while daily suckling duration started to decline slowly when solids were introduced (Figure 3). However, in infants given formula, as soon as regular formula feeds started, the breastfeeding frequency and suckling duration declined swiftly.[48]

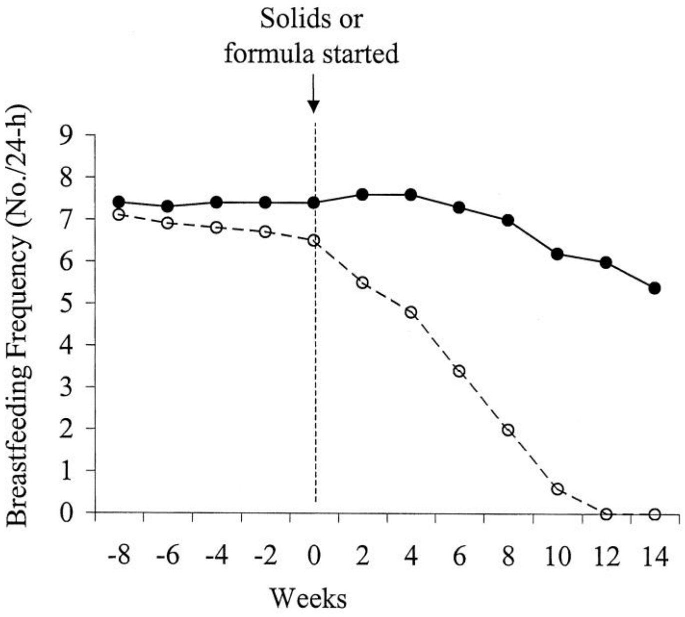

Key: Data are shown from 8 weeks before the start of solids or regular formula feeds to 14 weeks after the start. The 14-day period when solids and regular formula feeds were started is set to zero. ● indicates the solids group; ○ formula group

Figure 3. The median breastfeeding frequency in the solids and formula groups.

Breastfeeding duration was not associated with infants' age at introduction of solids (Figure 4). This is reassuring and suggests that early solid introduction will not impair breastfeeding performance.

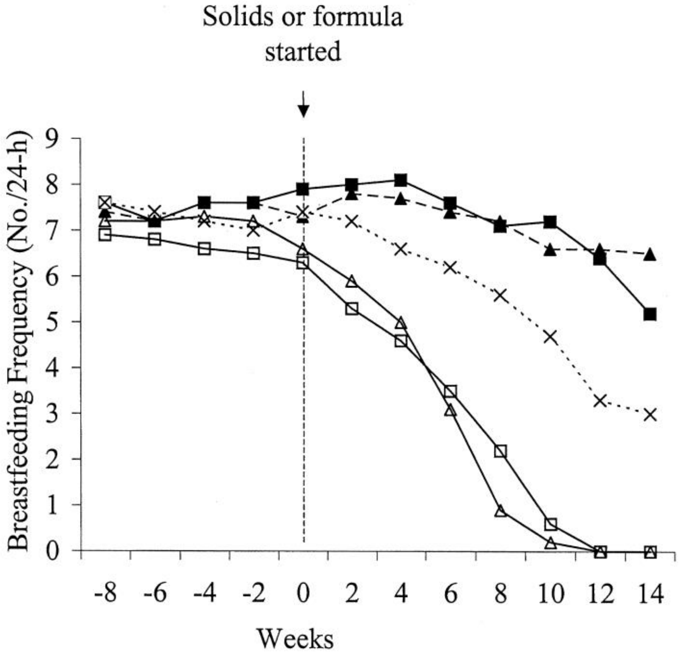

Key: Data are shown from 8 weeks before the start of solids or regular formula feeds to 14 weeks after the start. The 14-day period when solids or formula was started is set to zero. Solids: ■ = <4 months; ▲ = 4 to 6 months; × = 6 months. Formula: □ = <4 months; Δ = 4 months (includes 3 infants starting with regular formula at 6 months).

Figure 4. The median breastfeeding frequency by age at introduction of solids in the solids group and of formula in the formula group.

Allergenicity of cow's milk protein as yoghurt compared with cow's milk based formula

One study has evaluated whether patients with milk allergy could tolerate extensively heated milk products.[49] The study includes a section explaining the relationship between food processing (including heating) and allergenicity:

"Individuals with food allergy may generate specific IgE antibodies against conformational (dependent on tertiary structure) and sequential (linear) epitopes.... The allergenicity of food proteins may be altered by processing. High heat (e.g. baking) was found to reduce allergenicity of many food proteins, presumably by altering the conformation of heat-labile proteins that results in loss of conformational epitopes.[50] The classic examples are birch tree pollen allergen Bet v 1 cross-reactive proteins in apple (Mal d 1) and carrot (Dau c) that in the uncooked form cause oral symptoms (pollen–food allergy syndrome) but after heating are readily tolerated. In contrast, Bet v 1 cross-reactive protein in soybean, Gly m 4 retains allergenicity in heat-processed foods, suggesting that thermostability is highly variable and food-specific, even for the food allergens from the same protein family. Alternatively, in the case of peanut proteins, high temperature may enhance allergenicity as a result of glycation (the Maillard reaction) that induces the formation of Ara h 2 aggregates that are more resistant to gastric digestion and bind IgE antibody more effectively than unheated Ara h 2. Behavior under heating conditions is one of the determinants of allergenicity and may explain the different sensitizing potential of related foods, such as peanut and soybean. In addition to an individual susceptibility to heat and digestion, interactions with other substances present in a complex food, collectively referred to as the food matrix effect, may be crucial. Published data indicate that heating decreases but does not completely eliminate milk allergenicity. The caseins and a-lactalbumin have a higher heat stability than the other whey proteins, b-lactoglobulin and serum albumin. After heating milk at 100°C for 10 minutes, a substantial reduction of allergenicity was noted. Our studies evaluating the effects of heating (time and temperature) and food matrix on allergenicity of casein and whey proteins are ongoing." [49]

In this study unheated milk products used included unheated milk or yoghurt. In producing yoghurt milk is heated to 85°C transiently (to pasteurise the milk) and then cooled to 43°C. This is virtually identical to the pasteurisation process that store bought milk and the cow's milk in infant formula undergoes. This is high-temperature short-time pasteurization which involves heating the milk for a short period (heating to approximately 75°C for up to 30 seconds).

1.3.7 Justification for Reviewing All Infants at One Year of Age

We considered only reviewing a high risk sub-group at 1 year of age. However this fails to circumvent the problem of mothers in the control arm who choose not to introduce an allergenic food into their child's diet before the 3 year assessment. A proportion of these children may have had an allergy to particularly cow's milk or egg which was transient and will therefore no longer be present at the three year assessment. By seeing all children at one year of age and screening them for IgE mediated food allergy to the six intervention foods with skin prick testing this ensures children in both arms of the study are treated identically from this point onwards. This overcomes the inherent design difference between the intervention and control arms in the first year of life, specifically that intervention infants will manifest their IgE mediated food allergy in the first year of life as they are having all six foods introduced into their diet, whereas control infants are likely to not be consuming most of the allergenic foods in the first year of life (based on the Infant Feeding Survey 2005 experience).

1.3.8 Justification for the Three Year Assessment as the Principal Outcome

Most children who develop food allergy will have done so by the age of three. Indeed in the MAS study the sensitization rate to egg and milk had plateaued by 1 year of age, with virtually no new cases beyond 3 years of age.[51] With regard to the egg and milk allergy, the assessment is likely to be more accurate as these children will be assessed more contemporaneously with their condition.

Measuring objective lung function at three years of age is difficult but, in contrast, it is straightforward to determine the prevalence of the atopic wheeze phenotype. The atopic wheeze phenotype children are particularly important as Martinez was the first to show in his seminal paper that they are the children who will progress to develop persistent asthma.[52] This important observation has since been replicated in older children followed through to adulthood.[53] The most recent publication was a paper from the MAS cohort that showed that sensitisation to perennial allergens developing in the first 3 years of life was associated with a loss of lung function at school age.[54] From the Tuscon data the proportion of children that commenced wheezing after the age of 3 was only 15.0% (124/826).[55] The MAS cohort has shown that from the third birthday on, specific IgE levels to inhalant allergens were significantly higher than specific IgE levels to food allergens in children of the same age.[51] Further funding will still allow subsequent respiratory function assessment at 6 years of age.

1.3.9 Justification for the Challenging All Skin Prick Positive Children at One and Three Year Assessments

There was concern that any algorithm to assess for the presence of IgE mediated food allergy at the one and there year assessments that took into account current consumption of the allergenic foods in question would introduce a bias into the study. This is because the intervention arm would consist entirely of current consumers, whilst the control arm would be a mixture of non-consumers and consumers – the proportion of each varying depending on the allergenic food. In order to circumvent this, an algorithm for screening the children for possible IgE mediated food allergy to the six allergenic foods based solely on their skin prick test results at the one and three year assessments was considered desirable. In order to estimate the likely distribution of skin prick test results at these ages, data from the Food Allergy and Intolerance Research (FAIR)

study was kindly shared with the EAT study team (Table 4).[56]

Table 4. Distribution of skin prick results in FAIR cohort at 1 and 3 years of age

FAIR Data		SPT size (mm)			
		0<br>%	>0 - 2<br>%	>2 - <5<br>%	5+<br>%
Milk	1 year	98.4%	0.9%	0.4%	0.3%
	3 year	99.2%	0.3%	0.3%	0.2%
Wheat	1 year	99.7%	0.3%	0.0%	0.0%
	3 year	96.9%	1.6%	1.3%	0.3%
Egg	1 year	95.9%	2.1%	1.2%	0.8%
	3 year	98.0%	0.5%	0.5%	1.1%
Fish	1 year	98.8%	0.9%	0.3%	0.0%
	3 year	99.1%	0.5%	0.2%	0.3%
Peanut	1 year	98.0%	1.4%	0.4%	0.1%
	3 year	97.0%	0.5%	1.1%	1.4%
Sesame	1 year	98.6%	0.9%	0.4%	0.1%
	3 year	97.7%	0.9%	0.0%	0.6%

The FAIR study recruited a whole birth cohort of children (approximately 1,000) born on the Isle of Wight between 1st September 2001 and 31st August 2002 to provide information on the prevalence and incidence of food allergies and intolerance. The children were followed up at the ages of one, two and three years and a diagnosis of food allergy or intolerance made by a combination of history, skin tests and food challenges. These prevalence rates were extrapolated to the anticipated size of the EAT cohort at age 1 (2200 children) and age 3 (2000 children) to determine the likely number of children with positive skin prick test results (Table 5).

Table 5. Likely distribution of skin prick results at 1 and 3 years of age based on extrapolating FAIR data to the EAT Study

EAT Estimate		SPT size (mm)			
		0<br>N=	>0 - 2<br>N=	>2 - <5<br>N=	5+<br>N=
Milk	1 year	2165	20.2	8.7	5.8
	3 year	1984	6.3	6.3	3.1
Wheat	1 year	2194	5.8	0.0	0.0
	3 year	1937	31.3	25.0	6.3
Egg	1 year	2110	46.2	26.0	17.3
	3 year	1959	9.4	9.4	21.9
Fish	1 year	2174	20.2	5.8	0.0
	3 year	1981	9.4	3.1	6.3
Peanut	1 year	2157	31.8	8.7	2.9
	3 year	1941	9.4	21.9	28.1
Sesame	1 year	2168	20.2	8.7	2.9
	3 year	1953	18.8	0.2	12.5

Finally the likely number of food challenges required at 1 and 3 years in the EAT Study based on the extrapolated data was calculated using two different screening thresholds: all positive results and >2mm (Table 6).

Table 6. Likely number of food challenges required at 1 and 3 years of age based on extrapolating FAIR data to the EAT Study using two thresholds: all positive results and >2mm

Number of challenges	1 year		3 years	
	Any positive	>2mm	Any positive	>2mm
Milk	35	14	16	9
Wheat	6	0	63	31
Egg	90	43	41	31
Fish	26	6	19	9
Peanut	0*	0*	59	50
Sesame	0*	0*	31	13
Total	156	63	228	144
Per month	6.5	2.6	9.5	6.0
% of cohort	7.1%	2.9%	10.4%	6.5%

* Challenges not being undertaken to peanut and sesame at 1 year of age in EAT protocol

Based on Table 6, using the more sensitive (but less specific) screening criterion of any positive skin prick test result, 7.1% of the cohort (156 children) would require challenges at the one year assessment and 10.4% of the cohort (228 children) at the three year assessment. The monthly workload induced by this level of challenges is manageable even allowing for a potential doubling in these estimates to take into account the possibility of recruiting a cohort at higher risk of allergic disease than the Isle of Wight cohort.

1.4 RATIONALE FOR IMMUNOLOGICAL ASSESSMENTS

Subject to further funding

We will investigate the immunological evolution of food allergy (and other allergies) using lymphocytes collected sequentially during the follow-up of the cohort. We anticipate that this will enable us to both identify and predict the development of allergic conditions based on in vitro lymphocyte responses to antigenic stimulation.

The lymphocyte work is divided into two broad themes: firstly, antigen specific responses including T cell proliferation in response to allergen stimulation, antigen-specific precursor T cell frequency, intracellular cytokine production, specific IgE or IgG4 or other isotype responses; and secondly, non-antigen specific responses including CD4-CD25+ve cell expression, FOX-p3 expression and other regulatory cell markers. The antigen specific responses are likely to be linked to the cumulative prevalence of the food allergy outcome whilst the non-antigen specific responses are likely to be related to overall atopic disease outcomes.

1.5 KNOWN AND POTENTIAL RISKS

1.5.1 Early Weaning Group

Potential risks associated with the consumption of the allergenic foods from 3 months of age are worsening of eczema, weight gain, nutritional compromise, metabolic abnormalities, and an increased risk of allergy to the allergens.

Four different studies show that the median age of reacting to peanuts is between 14 and 24 months of age. The vast majority of patients react upon first known dietary exposure to peanut.[57-59] This argues strongly against the possibility that peanut allergy is caused by eating peanuts and provides some reassurance that we will not induce peanut allergy by feeding patients peanuts.

Both early (under 4 months) and late (after six months) introduction of wheat has been reported to be associated with coeliac disease.[60] Wheat will therefore not be introduced before four months of age.

1.5.2 Control Group

The control group will be following current UK Government weaning guidelines. In the control group, avoidance of the allergenic foods may result in an increased risk of allergy to the allergens.

1.5.3 Both Groups

Undergoing laboratory assessments may involve a low risk of haemorrhage, haematoma, and infection at the venipuncture site. Risks associated with the food challenges include nausea, vomiting, itching, urticaria, angioedema, asthma, other respiratory symptoms, and anaphylaxis.

2. Scientific Objectives

2.1 OBJECTIVE ONE

Hypothesis

The early introduction of allergenic foods (from 3 months of age) will induce regulatory mechanisms that result in a reduced level of food allergy by 3 years of age. The effect on food sensitization at 3 years of age will be determined.

Objectives

01a) To set up a randomized controlled intervention trial to assess the impact on food allergy through the early introduction of allergenic foods from 3 months of age. To randomize the cohort into two groups, with exclusive breast feeding for 6 months in the control group and breast feeding to 6 months combined with the sequential introduction of allergenic foods from 3 months in the intervention group.

01b) To complete the antenatal recruitment of 3000 mothers, with 2500 infants available for randomization at 3 months of age (1250 infants to be randomized into the control group and 1250 infants to be randomized into the intervention group) with complete assessment of maternal nutrition during pregnancy and throughout breast-feeding.

01c) To follow-up the whole cohort until the primary assessment point at three years of age.

01d) To measure the primary outcome of the period prevalence of IgE mediated food allergy to the six intervention foods between one and three years of age in both arms (Section 3.3).

2.2 OBJECTIVE TWO

Hypothesis

The early introduction (from 3 months of age) of allergenic foods into the infant's diet may lead to a reduction in the prevalence of other allergic conditions by three years of age: specifically asthma (including atopic wheeze), eczema, allergic rhinitis (including aero-allergen sensitization) combined food allergy prevalence (including food sensitization) and the prevalence of combined allergic disease.

Objectives

02a) To assess the prevalence of the secondary end points - asthma (including atopic wheeze), eczema, allergic rhinitis (including aero-allergen sensitization), combined food allergy prevalence (including food sensitization) and the prevalence of combined allergic disease with breast-feeding duration.

02b) To assess the individual prevalence of the secondary end points - asthma, eczema and allergic rhinitis at repeated time points during the three year follow up amongst the intervention group compared with the control children. Allergic symptoms will be assessed monthly until one year of age and 3 monthly until 3 years of age.

02c) To measure the cumulative prevalence at three years of age of any allergic condition: asthma (including atopic wheeze), eczema, allergic rhinitis (including aero-allergen sensitization), combined food allergy prevalence (including food sensitization) in the intervention group compared with the control group.

02d) To measure the prevalence of allergic conditions by 3 years of age by both subjective (parent completed questionnaires of reported symptoms, doctor diagnoses of conditions) and objective (skin prick testing & specific IgE) measures.

02e) To collect and store blood from both the control and intervention group at birth, 3 months, 1 year and 3 years of age. To extract DNA from cord blood to screen for defects in the currently known skin barrier genes as well as to undertake immunological analyses. The blood samples collected will be used to investigate the immunological evolution of food allergy (and other allergies). Where no cord blood was taken, samples for DNA analysis will be taken with the non-invasive Oragene® DNA self-collection kits from buccal mucosa.

2.3 OBJECTIVE THREE

Hypothesis

The early introduction of antigenic foods does not have any deleterious effects.

Objectives

03a) To assess the growth and nutritional status of children in the intervention arm compared with the control group.

03b) To ensure the rapid assessment of any participant with a suspected food allergic reaction.

3. Study Design

3.1 OVERALL STUDY DESIGN

The study design with regard to ascertaining the principal outcome – IgE mediated food allergy, is summarized in Figure 5.

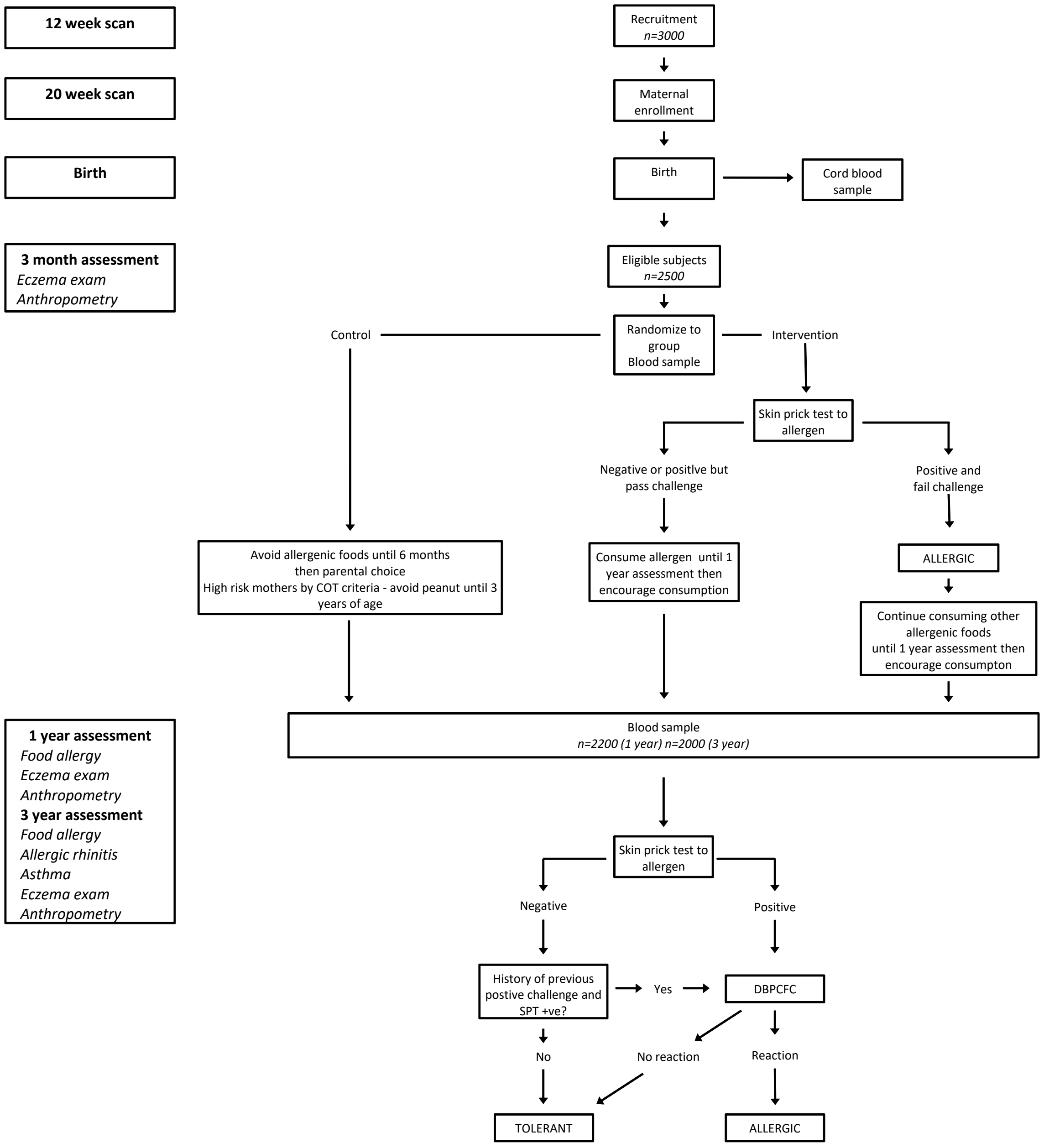

FIGURE 5. OVERALL STUDY DESIGN

An overview of the study and the timing of interim assessments are given in the Study Overview Chart (Appendix 1). The timescale for the project is graphically represented in the Study Gantt Chart (Appendix 2).

3.1.1 Population

Study recruitment will start from two large London antenatal units (Kingston Hospital and St Thomas' Hospital) will participate in the study. Both have over 5000 deliveries a year, thus requiring between 10 and 15% of mothers to participate. Expectant mothers will be approached at their 12 week scan. Information about the study will be provided in the two centres by a full time research assistant. Mothers will then be formally consented and enrolled onto the study at their 20 week scan.

Recruitment is designed to be non-linear, being slower at the onset of the study and accelerating as the study progresses. Thus the actual recruitment time scale that we have used to set the milestones is:

Start of antenatal recruitment	Time	Rate (#/month)
Antenatal recruitment of 500 mothers completed	+ 8 months	62
Antenatal recruitment of 1000 mothers completed	+ 12 months	125
Antenatal recruitment of 1500 mothers completed	+ 15 months	167
Antenatal recruitment of 2000 mothers completed	+ 18 months	167
Antenatal recruitment of 3000 mothers completed	+ 24 months	167

3.1.2 Backup Recruitment Strategies

A number of measures have been taken to try and ensure satisfactory recruitment and retention of participants. Measures to minimize loss to follow-up will include: a central telephone number, pre-printed free-post notification of moving cards and contact details of grand-parents who our previous studies have shown are much less likely to move. Additionally a high proportion of subjects are likely to have e-mail addresses which we will also record. The ease of access to a dedicated team incorporating dieticians, nurses, paediatricians and a dermatologist via a dedicated study direct line will also be a significant incentive to stay in the project. The majority of children have no access to tertiary paediatric allergy or dietetic services and participation in the study will enable parents to access these scarce resources.

The recruitment rate will be assessed on a monthly basis. If the required recruitment rate is not met for three consecutive months a decision would be taken by the trial steering committee to seek to boost recruitment by using one or both of the following mechanisms:

Additional recruitment centres
The study could utilise one or two additional recruitment centres. These would be chosen from amongst the following centres: Queen Charlotte's Hospital, Chelsea & Westminster Hospital, Royal Free Hospital and Kings College Hospital.

Bounty scheme
The Bounty scheme maintains a register of pregnant women in the United Kingdom. This will entail Bounty mailing out an information leaflet about the study with our contact details to pregnant mothers utilising the Bounty register. Mothers, having read the information leaflet, would initially contact a call centre that would screen respondents to ensure they were eligible to participate in the study. Details of eligible potential participants would then be forwarded to the study team who would contact the mother to provide further information and initiate the process of obtaining informed consent for the mothers' participation in the study.

3.1.3 Maternal participation

Maternal diet will be assessed using a Food Frequency Questionnaire (FFQ) based on Carina Venter's FFQ validated[56] for milk, wheat, peanut and fish and Kate Grimshaw's FFQ validated (personal communication) for milk and egg.

In addition, to allow comparison with results from the LEAP Study, the questions used to assess maternal ingestion of peanut in LEAP will also be administered.

This will be completed by the mother at the 20 week ultrasound scan and then at 34 weeks gestation.

3.1.3.1 Maternal antenatal and lactation dietary advice relating to allergen food consumption:

As stated in Section 1.3.3, in 1998 COT issued advice with regard to peanut consumption for pregnant women whose unborn child was regarded as being at high risk of atopy. These were women with a history of atopy themselves, or in the father of the baby or in any sibling of the baby. Pending revision mothers in the EAT Study who fulfil these criteria will be advised to follow the recommendations relating to pregnancy and lactation:

- Pregnant women who are atopic, or for whom the father or any sibling of the unborn child has an atopic disease, may wish to avoid eating peanuts and peanut products during pregnancy
- Breast feeding mothers who are atopic, or those for whom the father or any sibling of the baby has an atopic disease, may wish to avoid eating peanuts and peanut products during lactation

3.1.3.2 Parental allergic sensitization

We will also seek to undertake skin prick testing and obtain a sample of blood from both biological parents when they attend the 3 year assessment clinic with their child. The blood sample will be processed and stored for subsequent specific IgE measurement.

3.1.3.3 Cord blood collection

At antenatal recruitment, mothers will be given the appropriate containers to allow the collection of cord blood at their delivery. The midwife delivering the baby will be asked to collect blood. This will be stored in a fridge prior to collection by the recruitment research assistants in the hospital. Samples will be transferred to the study laboratory and serum will also be collected and stored for future sero-epidemiological work.

The FSA will have ownership of the samples and will licence this out to Kings, for use in a project funded by a third party, subject to FSA approval. The eligibility of any third party and decisions on how to use the samples will be taken by the Steering Committee, of which the FSA is a member.

3.2 INFANT ENROLMENT - SCREENING FOR ALLERGY AT 3 MONTHS

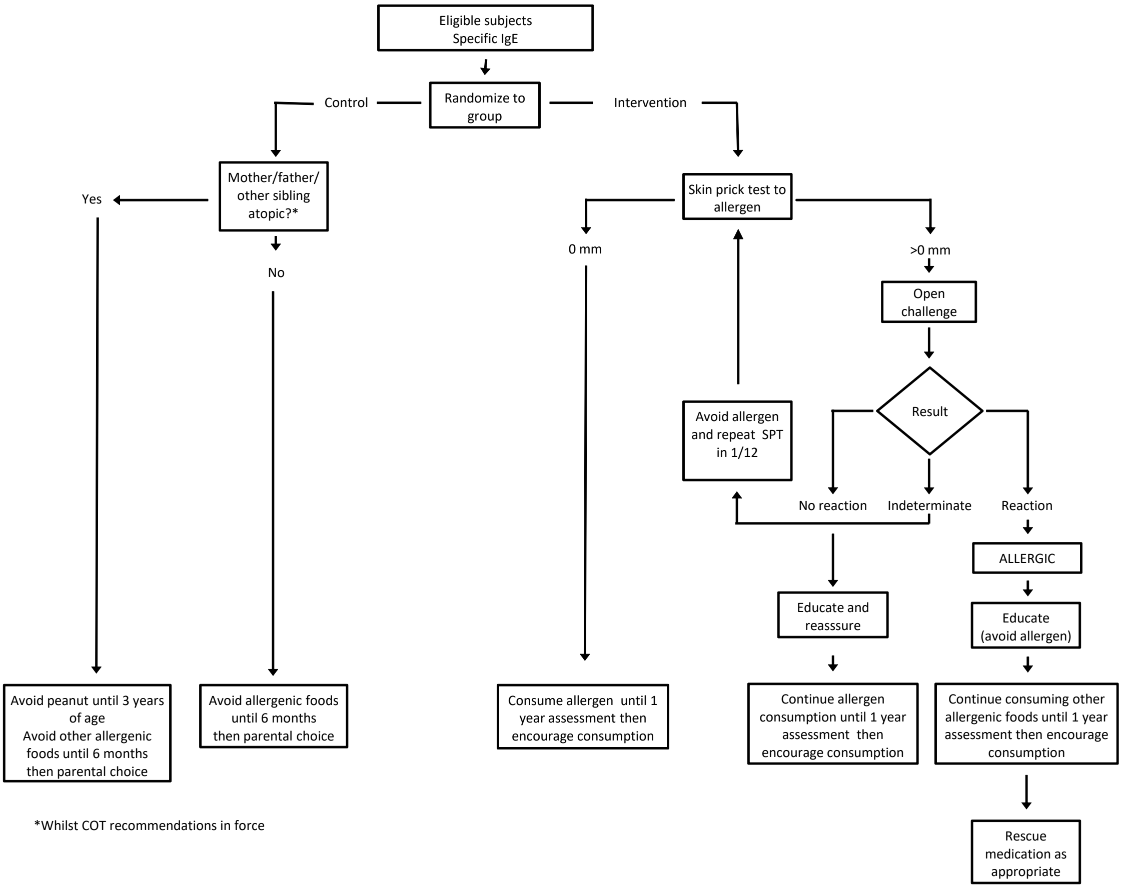

FIGURE 6. THREE MONTH SCREENING VISIT

Infants in the intervention arm will undergo screening for pre-existing food allergies when they attend the three month visit. These infants will undergo skin prick testing in duplicate with the following allergens: raw egg white, whole cow's milk, sesame paste (tahini), fish (cod), wheat, peanut and positive (histamine 10 mg/ml) and negative control (50% glycerol, 50% buffered saline) solutions.

For screening purposes a positive skin prick test will be defined as >0 mm for any allergen. Using a low threshold and whole foods will minimize the risk of false negative results.

3.3 PRIMARY ENDPOINT

The period prevalence of IgE mediated food allergy to the six intervention foods between one and three years of age in both arms.

3.3.1 Definition of IgE mediated food allergy for primary endpoint

1. A positive DBPCFC at 1 year or 3 years of age (as per the algorithm in Figure 9) in a child sensitized to one of the six intervention foods

2. A positive DBPCFC between 1 year and 3 years in a child attending an unscheduled clinic visit (as per the algorithm in Figure 7) in a child sensitized to one of the six intervention foods

Whilst the first 2 categories relate to events between one and three years of age, we will include children potentially outside of this range in two exceptional circumstances:

3. A positive challenge (open or DBPCFC) at between six months and one year of age that occurs in a child who is sensitized to one of the six intervention foods who subsequently refuses a DBPCFC at 1 year and 3 years of age

   Rationale: Below six months only intervention children have challenges so this category is restricted to those infants who are six months old or more.

4. A food allergic history in a child with a SPT ≥5 mm (as per the algorithm in Figure 7)

   Rationale: There will be a small number of children who have an immediate type allergic reaction and are significantly sensitized whose parents refuse to allow them to undergo any further challenge.

When the final outcomes are published, the relative contributions of children to the final outcome in each of these four categories will be presented separately as well as the overall cumulative figure to allow reviewers to independently assess the data.

3.4 SECONDARY ENDPOINTS

Period (one to three years of age) prevalence food outcomes

The period prevalence of all IgE mediated food allergy between one and three years of age in both arms.

The period prevalence of all food allergy (IgE and non-IgE mediated) between one and three years of age in both arms.

The period prevalence of sensitization (defined in Section 3.9) to food between one and three years of age in both arms.

The total number of foods (of the six intervention foods) to which IgE mediated food allergy has been diagnosed between one and three years of age in both arms amongst children with IgE mediated allergy to one or more of the six intervention foods.

The total number of foods (any foods) to which IgE mediated food allergy has been diagnosed between one and three years of age in both arms amongst children with IgE mediated allergy to one or more foods.

Cumulative (by three years of age) prevalence food outcomes

The cumulative prevalence of IgE mediated food allergy to the six intervention foods by three years of age.

The cumulative prevalence of all IgE mediated food allergy by three years of age.

The cumulative prevalence of all food allergy (IgE and non-IgE mediated) by three years of age.

The cumulative prevalence of non-IgE mediated food allergy by three years of age.

The cumulative prevalence of sensitization (defined in Section 3.9) to the six foods by three years of age.

The total number of foods (of the six intervention foods) to which IgE mediated food allergy has been diagnosed by three years of age amongst children with IgE mediated allergy to one or more of the six intervention foods.

The total number of foods (any foods) to which IgE mediated food allergy has been diagnosed by three years of age amongst children with IgE mediated allergy to one or more foods.

Other allergic disease outcomes

The point prevalence of eczema at one year and three years of age and cumulative prevalence of eczema by three years of age.

The modified ISAAC eczema questionnaire will be used as a screening tool throughout the study. In addition, all children will be physically examined for eczema at the 3 assessment points (3 months, 12 months and 3 years), using a photographic protocol based on the UK diagnostic criteria for eczema.

The severity of eczema at one year and three years of age.

The severity of eczema at 3 months, one year and three years of age will be assessed by modified SCORing Atopic Dermatitis score (SCORAD)[61] *and Patient-Oriented Eczema Measure (POEM).*[62]

The prevalence of allergic rhinitis at three years of age.

The prevalence of inhalant allergen sensitization at one year and at three years of age by skin prick test

Based on positive skin prick reactions to one or more of the aero-allergens (house dust mite, cat, dog, 6 grass mix and 3 tree mix)

The prevalence of inhalant allergen sensitization at one year and at three years of age by specific IgE measurement

Based on a positive specific IgE result to a panel of aeroallergens at one or three years of age.

The prevalence of the atopic wheeze phenotype at three years of age

Composite allergy outcome

The prevalence of combined allergic disease (a composite of cumulative IgE mediated food allergy to all foods, atopic wheeze phenotype, eczema and allergic rhinitis) at three years of age

The prevalence of combined allergic disease (a composite of cumulative IgE and non-IgE mediated food allergy to all foods, atopic wheeze phenotype, eczema and allergic rhinitis) at three years of age

Safety outcome

Incidence of adverse events and laboratory abnormalities; nutritional evaluations.

Immunological outcomes

Results of cellular and humoral assessments of immune response related to the development of allergy or tolerance to specific allergens (*subject to additional funding*)

Genetic analyses

The association between skin barrier gene defects (such as carriage of the filaggrin skin barrier mutations) and other measures of skin barrier integrity (transepidermal water loss) with all the study outcomes will be assessed.

3.5 DETERMINATION OF FOOD ALLERGY – UNSCHEDULED CLINIC VISITS

We will both actively seek (using the dietary compliance questionnaires) and passively obtain (by parental report to a dedicated project telephone line) reports of possible food allergic reactions or persistent refusal and aversion. Parents will be encouraged to complete dietary compliance questionnaires online which will facilitate the rapid identification of positive respondents. Hard copies will be available and posted out to those without internet access. If the reactions are felt to be suggestive of food allergy, the parent will be advised to avoid that particular food and the children will attend the paediatric allergy clinical trials unit to undertake an unscheduled clinic visit and follow the algorithms outlined in Figure 7 for suspected IgE mediated reactions and Figure 8 for suspected non-IgE mediated reactions.

Skin prick tests will be to determine an individual child's sensitization status.

Open challenges will be undertaken in those under one year of age and double blind placebo controlled challenges in those over the age of one year.

5mm skin prick threshold at UCV for IgE mediated allergy
Children who attend an UCV with a clear history of a clinical allergic reaction and who are found to have a skin prick response to that food of 5mm or more will be deemed allergic and not undergo a challenge at that UCV (Figure 7). They will, however, be invited to undergo a challenge at their next routine assessment visit (either the 1 year – except for peanut and sesame reactions, or 3 year assessment depending on their age when the clinical allergic reaction occurred). The logic is that some children will subsequently outgrow their allergy (especially for milk and egg) and this needs to be assessed.

The 5mm threshold is based on the limited data that exists for positive predictive values (PPVs) for young children. Based on 555 open food challenges (339 challenges to cow milk, 121 to egg, and 95 to peanut) in 467 children (median age for cow's milk challenge 31.0 months, for egg 36.2 months, and for peanut 57.8 months) SPT wheal diameters that were "100% diagnostic" for allergy were defined to cow's milk (≥8 mm), egg (≥7 mm) and peanut (≥8 mm). In children less than 2 years of age, the corresponding weal diameters were ≥6 mm, ≥5 mm and ≥4 mm, respectively.[63, 64] Thus 5mm is the appropriate threshold for our cohort which covers the age range 3 months to 3 years. The 4mm threshold for peanut is not relevant as we will not be challenging children to peanut until 3 years of age at which point the threshold would have been around 5mm in the study quoted above (having been 4mm for under 2 year of children and 8mm at a median challenge age of 57.8 months).

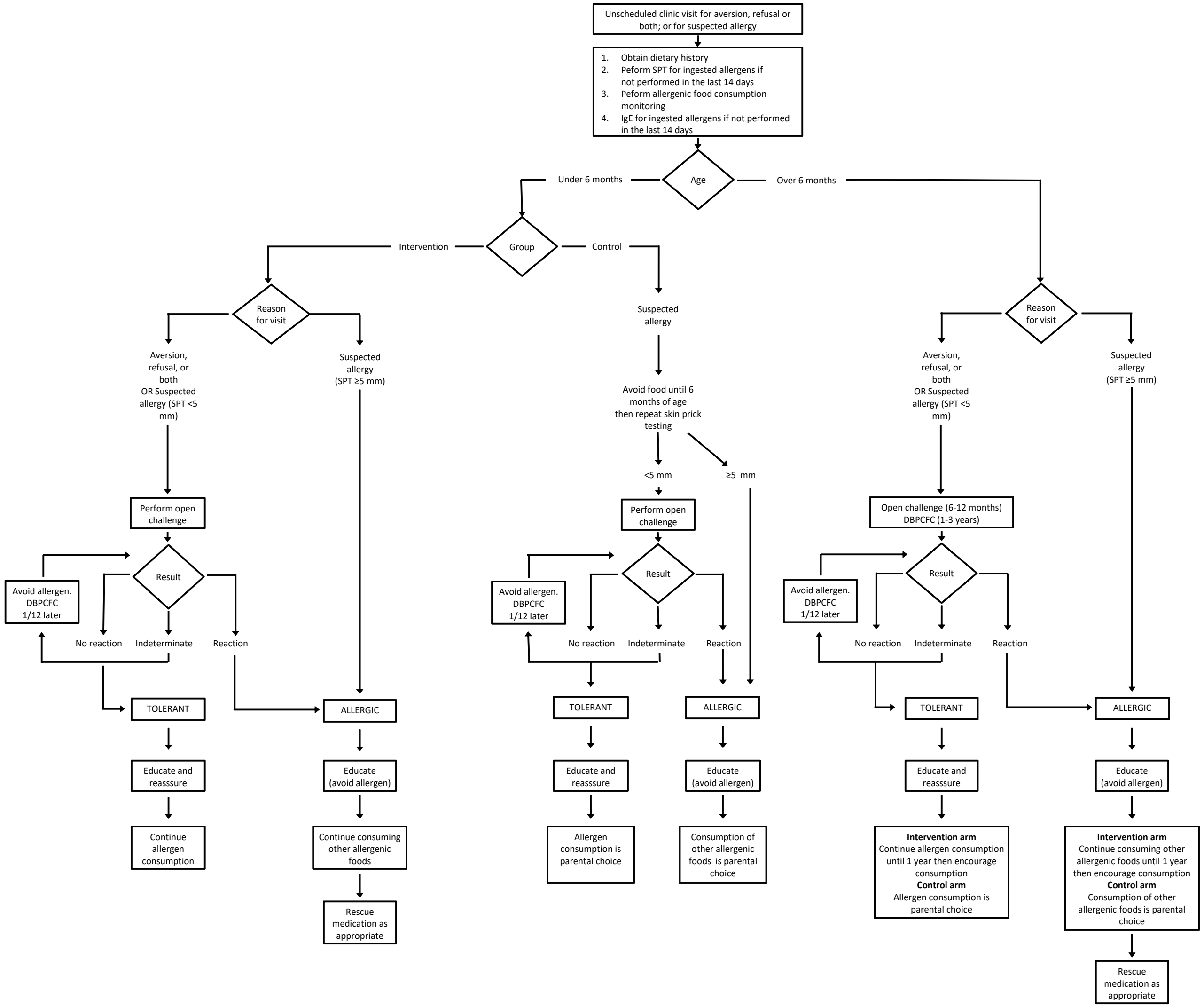

FIGURE 7. ASSESSMENTS TO BE PERFORMED AT UNSCHEDULED CLINIC VISITS- IGE MEDIATED FOOD ALLERGY

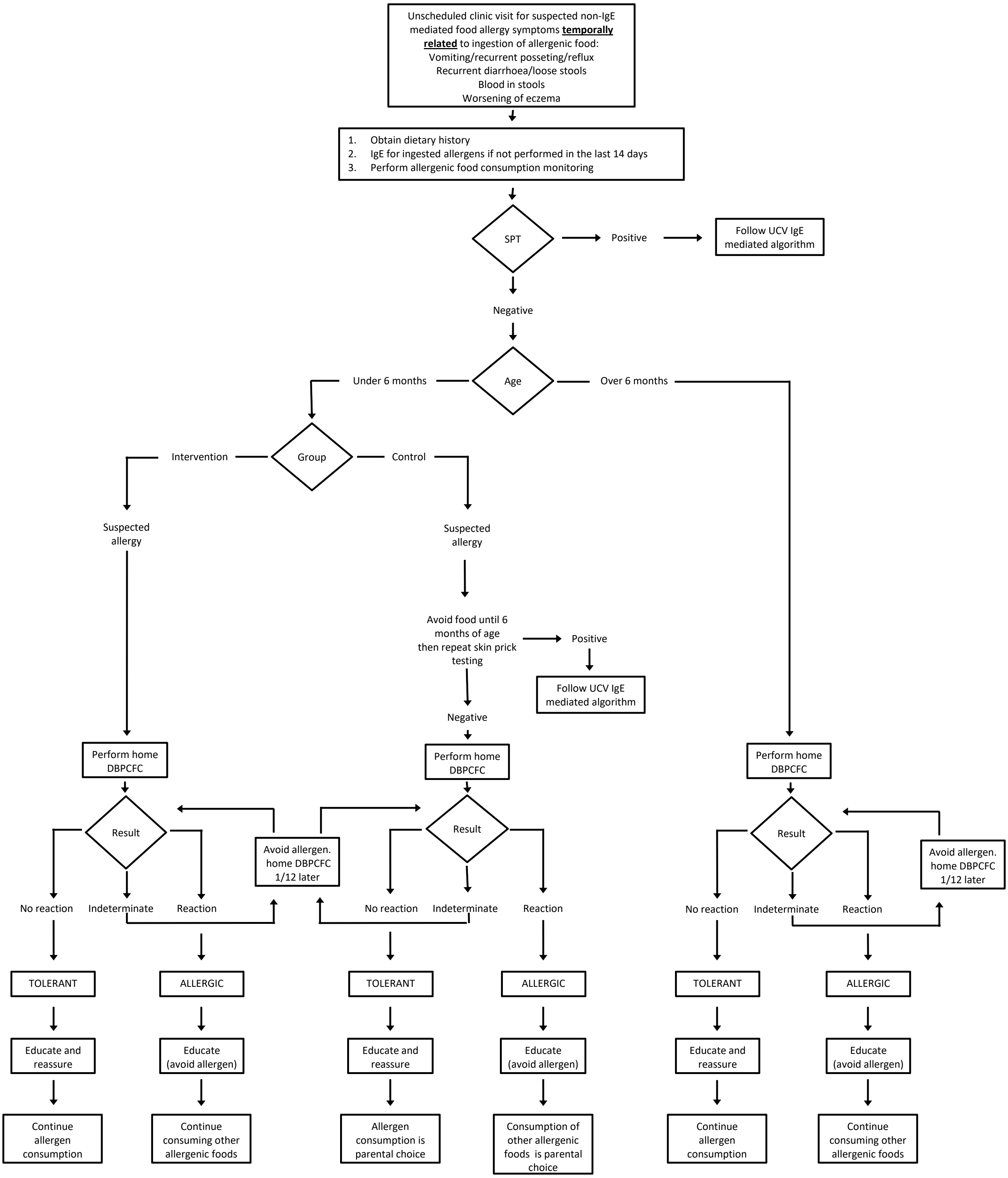

FIGURE 8. ASSESSMENTS TO BE PERFORMED AT UNSCHEDULED CLINIC VISITS- NON-IGE MEDIATED FOOD ALLERGY

3.6 DETERMINATION OF FOOD ALLERGY – 1 YEAR ASSESSMENT

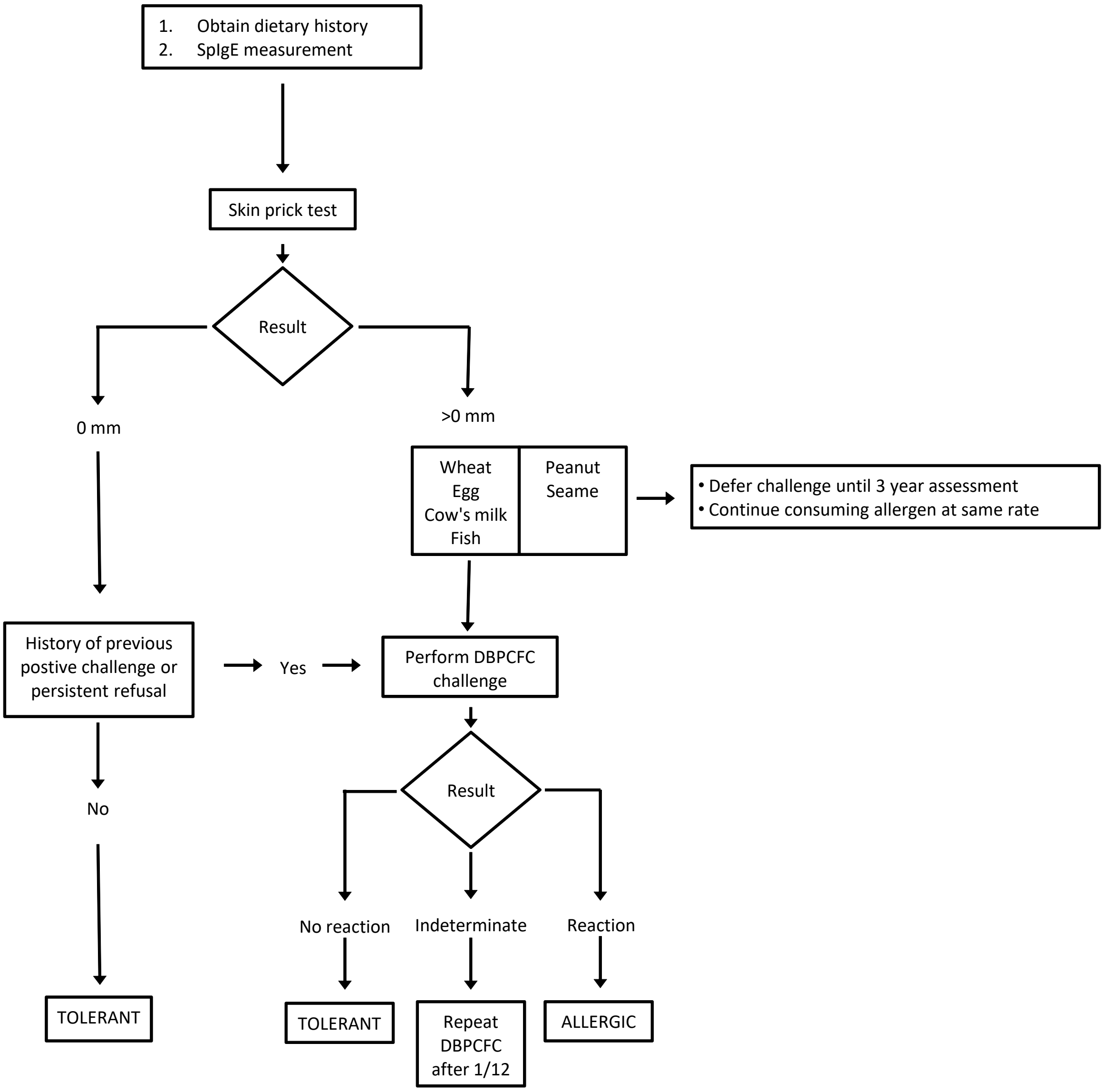

FIGURE 9. ONE YEAR AND THREE YEAR ASSESSMENT VISITS – IGE MEDIATED FOOD ALLERGY

All infants will be invited to attend an interim one year assessment. It is anticipated that approximately 10% of infants will have visible eczema present at the 3 month assessment point. Infants with early onset eczema, particularly if moderate or severe in intensity, have a significantly increased risk of food allergies. In the ALSPAC study the proportion of parent reported eczema by 6 months of age was 42.9%. However, this includes transient and mild eczema. The cohort of 10% of infants with eczema present at 3 months of age in this study will therefore consist of approximately 250 children. The prevalence of food allergy amongst children such as these reported in the literature varies from 27% to 37%, and we estimate a prevalence of 30%. It is estimated that with loss to follow up, there will be 220 high risk children amongst those attending the one year assessment.

The study is designed to have sufficient power to detect a protective effect in this high risk sub-population (see Section 9.3).

Food allergy status will be determined using the same algorithm as the 3 year final assessments (Figure 9). It will then be possible to assess whether the intervention is associated with an altered prevalence of eczema and whether the intervention is effective at reducing food allergy in high risk children.

Peanut and sesame sensitization

Children who are found to be sensitized to peanut and sesame at the 1 year assessment will not undergo a challenge until 3 years of age. In the interim they will be told to maintain their consumption at the same rate. For the intervention arm this will mean continuing to consume peanut and sesame in the recommended quantities. The reason for deferring the challenges is that there is a theoretical risk that undertaking a sesame or peanut challenge in a control infant who has been exposed to little or no sesame or peanut could have a tolerizing effect and induce the same kind of protection that regular peanut and sesame consumption is aiming to achieve in the intervention arm.

3.7 DETERMINATION OF FOOD ALLERGY – 3 YEAR ASSESSMENT

All children not lost to follow up will be invited to attend the final 3 year assessment. They will be assessed as per the algorithm outlined in Figure 9.

Those children with indeterminate responses to their DBPCFC will undergo a repeat DBPCFC one month later.

3.8 DETERMINATION OF SENSITIZATION

At three months of age sensitization will be determined in a serum sample to the six intervention foods: egg white, milk, sesame, fish, wheat, peanut and soya.

At 12 months of age, serum will be obtained from children to measure sensitization to the six intervention foods and to a panel of aero-allergens including: *D. Pteronyssinus*, *D. Farinae*, cat and dog dander, horse dander, timothy grass, cladosporum, silver birch, olive, mugwort and nettle.

At 3 years of age all children will undergo the same assessment as that done at 12 months.

As noted in Section 1.3.5, specific IgE levels to the intervention foods will not be measured until the child reaches the end of the study.

3.9 STUDY DEFINITIONS

3.9.1 Allergic disease definitions

Allergic sensitization. Either allergen-specific IgE >0.1 kU/L, as defined by the CAP System™ (Pharmacia Diagnostics AB); or positive SPT, defined as SPT wheal diameter >0 mm with appropriate controls.

Asthma. A history of cough, wheeze, or shortness of breath that (1) was responsive to therapy with bronchodilators on two or more occasions in the previous 24 months, (2) required one visit to a physician in the previous 24 months, and (3) occurred during the night, during early morning, or upon exercising in the intervals between exacerbations at any time in the previous 12 months.

Eczema. A rash on physical examination which fulfils the UK diagnostic criteria for eczema.

Failure to thrive. Failure to thrive based on Child Growth Foundation thrive charts.

Food allergy. Positive reaction on open challenge (under 1 year) or DBPCFC (over 1 year). IgE mediated if skin prick test results is >0 mm. Non-IgE mediated if skin prick test result is negative.

Life-threatening anaphylaxis. An allergic reaction accompanied by any of the following: hypoxia, as evidenced by central cyanosis or oxygen saturation ≤ 89%; hypotension; loss of consciousness; or admission to intensive care.

Perennial rhinitis. Sensitization to a perennial allergen (house dust mite, cat or dog) and ISAAC rhinitis symptoms experienced when exposed to the relevant allergen.

Seasonal rhinitis. Sensitization to a seasonal allergen (grass or tree pollen, moulds) and ISAAC rhinitis symptoms experienced during the relevant season.

Rhinitis. ISAAC Core Questions: Has your child ever had a problem with sneezing, or a runny, or blocked nose when you DID NOT have a cold or the flu? In the past 12 months, has your child had a problem with sneezing, or a runny, or blocked nose when he/she DID NOT have a cold or the flu? Has your child ever had hayfever?

3.9.2 Breast feeding definitions

Breast feeding definitions are taken from the recent WHO report on indicators for assessing infant and young child feeding practices (Table 1).[65]

Table 7. Criteria that define selected infant feeding practices

**Feeding practice**	Requires that the infant receive	Allows the infant to receive	Does not allow the infant to receive
Exclusive breastfeeding	Breast milk (including milk expressed or from a wet nurse)	ORS, drops, syrups (vitamins, minerals, medicines)	Anything else
Predominant breastfeeding	Breast milk (including milk expressed or from a wet nurse) as the predominant source of nourishment	Certain liquids (water and water-based drinks, fruit juice), ritual fluids and ORS, drops or syrups (vitamins, minerals, medicines)	Anything else<br>(in particular, non-human milk, food-based fluids)
Complementary feeding	Breast milk (including milk expressed or from a wet nurse) and solid or semi-solid foods	Anything else: any food or liquid including non-human milk and formula	NA
Breastfeeding	Breast milk (including milk expressed or from a wet nurse)	Anything else: any food or liquid including non-human milk and formula	NA
Bottle-feeding	Any liquid (including breast milk) or semi-solid food from a bottle with nipple/teat	Anything else: any food or liquid including non-human milk and formula	NA

As stated in Section 1.3.3 a pragmatic approach has been adopted to the definition of exclusive breastfeeding to be used in the EAT study. To reiterate, it will be deemed acceptable for mothers to have given boiled then cooled water, as well as oral rehydration solution, without being considered to have ceased exclusive breastfeeding as this will not have any immunomodulatory effect on the infant.

When the study results are published, the percentage of women who achieved 3 months exclusive breastfeeding by the WHO criteria in Table 7 in each group will be presented.

3.10 STOPPING RULES

3.10.1 Ongoing Review

The steering committee will review safety data on an ongoing basis. Safety data will be forwarded to the EAT Data Monitoring Committee (DMC). The DMC may stop enrolment or participation in the trial at any time if it concludes that there are significant safety concerns (see Section 8).

3.10.2 Review of Specific Adverse Events

3.10.2.1 Stopping Enrolment

Enrolment in the trial will be stopped pending review if:

- Any death occurs
- A participant is admitted to an intensive care unit for an adverse event related (NCI-CTCAE attribution of adverse events Code 4 or 5 – see Section 8.5.2 Table 2) to study intervention.
- A participant randomly assigned to the allergenic food consumption group experiences life-threatening anaphylaxis during the first 3 weeks of administration of the allergenic food.

Enrolment in the trial will be stopped pending review if either of the following occurs for the intervention arm:

- An analysis performed when 200 such participants in the intervention arm have been followed for 3 months demonstrates that the lower bound of the 95% confidence interval for the proportion of participants in the intervention arm with IgE mediated food allergy as determined in an unscheduled clinic visit (see section 3.5 and Figure 7) is greater than 6%.
- An analysis performed when 200 participants per group have been followed up to 12 months of age demonstrates that the rate of serious adverse events is significantly greater at the 0.05 significance level in the allergenic food consumption group than in the control group.
- An analysis performed when 10 such participants in the allergenic food consumption group have been followed until 12 months of age demonstrates that 4 or more participants have experienced a related (NCI-CTCAE attribution of adverse events Code 4 or 5 – see Section 8.5.2 Table 2) serious adverse event.

The analysis for these three categories will be undertaken by the trial statistician and the results conveyed to the DMC.

3.11 STUDY DURATION

Enrolment is expected to take 2 years. Infant/child participation is for 3 years. The study duration is expected to be 5 years 11 months.

4. ELIGIBILITY

4.1 INCLUSION CRITERIA

1. Pregnant mothers attending their 12/20 week ultrasound scans.
2. Mothers planning on exclusively breast feeding for at least the first 3 months
3. Informed consent obtained from parent or guardian.

4.2 EXCLUSION CRITERIA

1. Significant antenatal anomaly at 20 week ultrasound scan.
2. Multiple pregnancy
3. Significant congenital disease (enteropathy, congenital heart disease, renal disease)
4. Premature delivery (less than 37 completed weeks gestation)
5. Parents not planning on exclusively breast feeding for at least the first 3 months.
6. Parents planning on moving away from London before their child is three years of age
7. Parents unable to speak and read English
8. Unwillingness or inability to comply with study requirements and procedures.
9. Family intend infant to be on a restricted diet (any of the six intervention foods)
10. ALT or bilirubin >2 times the upper limit of age-related normal value.
11. Urea or creatinine >1.25 times the upper limit of age-related normal value.
12. Platelet count <100,000/ml, haemoglobin <9 g/dl, or investigator-suspected immunocompromise.

4.3 PREMATURE TERMINATION

4.3.1 Premature Termination of Trial Interventions

Trial intervention will be prematurely terminated for a participant if, in the judgment of the investigator, further participation in the trial would be deleterious to the participant's health.

4.3.2 Premature Termination from the Trial

Participants will be prematurely terminated from the trial for either of the following:

- Withdrawal of consent
- Failure to return

Such participants will not be replaced.

5. STUDY INTERVENTION

5.1 ALLERGENIC DIET ADMINISTRATION OR AVOIDANCE

Infants who are randomized at 3 months of age to the intervention arm of the study will be provided with an individualized feeding plan. Infants will first be offered baby rice mixed with breast milk or water. Then cow's milk based yoghurt will be commenced. Subsequently the other allergenic foods – egg, wheat, fish, sesame and peanut will be introduced in randomized order with the aim of all foods being ingested in the required quantities by five months of age. What will not be introduced before 4 months of age.

The required quantity of each allergenic food to be ingested by five months of age is 4 grams of protein per week. This equates to the following quantities per week:

Cow's milk: Two small pots of yoghurt

Sesame: 2 teaspoons of tahini (sesame paste)

Wheat: 2 Weetabix or 40 grams dry pasta

Egg: 1 small egg

Fish: 20 grams fish

Peanut: 2 teaspoons of peanut butter

Infants in the intervention arm will be required to consume the allergenic foods until 1 year of age, at which point ongoing consumption of all six allergenic foods will be encouraged. This is unlikely to be an issue with regard to cow's milk and wheat and in the majority of families for egg and fish which will be consumed as part of the normal family diet on a regular basis. Sesame and peanut will be the only two foods for whom the encouragement to continue consuming the foods might result in the child consuming a diet significantly different to that which they would have done otherwise. At three years of age, subsequent consumption of the allergenic foods will be left to parental choice.

Participants assigned to the control arm will follow the UK Government weaning advice i.e. exclusive breast feeding until around six months of age and no introduction of allergenic foods before this age.

COT recommendations
As stated in Section 1.3.3, the Government is likely to revise the COT recommendations on peanut consumption during pregnancy, lactation and in infancy in high risk families. However, whilst the recommendations remain active, mothers in the control arm who fulfil the criteria stipulated by COT with regard to being at high risk of atopy (Section 3.1.3.1 above) will follow the postnatal recommendations of the committee:

- In common with the advice for all children, infants with a parent or sibling with atopic disease should, if possible, be breast-fed exclusively for four to six months
- During weaning, and until they are at least three years of age, peanuts and peanut products should be avoided

Methods to facilitate compliance with UK Government weaning advice
When mothers are approaching their due date, they will be sent information detailing the support that is available in their particular hospital with establishing breast feeding. Further encouragement and advice will be provided by the study dieticians at the 3 month clinic assessments. Any mother experiencing difficulty with breast feeding will be able to contact us via a dedicated study helpline and given practical advice to encourage breast feeding for at least 6 months in both groups.

5.2 ASSESSMENT OF COMPLIANCE WITH STUDY INTERVENTION

The study dieticians will be responsible for ascertaining compliance with the intervention. Compliance to the diet will be assessed by parent completed questionnaire with the following frequency:

Age	Frequency	Total number of interim compliance assessments*
3-12 months	Monthly	8
1 year until 3 year assessment	Three monthly	7

* excluding clinic assessments

Each compliance assessment will document:

1) Consumption of the six allergenic foods (both quantity and frequency) in both the intervention arm and the control group. Parents in both arms will be asked for the preceding month/3 months: On average over the period what percentage of the recommended allergenic food quantity their child has consumed? For what percentage of the period has their child being eating the allergenic foods?

2) Breast feeding status – mothers experiencing difficulty with breast feeding will be able to contact us via a dedicated study helpline

3) Consumption of any other foods

4) Additionally the compliance assessments will enquire about a history of any allergic symptoms, including possible type 1 reactions to food, using the validated instruments described in the outcomes section in accordance with the schedule of events (see Appendix 3).

Validation of the dietary component of the compliance questionnaire
The allergenic food frequency questionnaire has been designed specifically for this study. It uses (with permission) data from the food diaries completed by parents of infants in the UK arm of the EuroPrevall study. This data enables lists to be compiled for each allergenic food of which foods constitute the most significant proportion of consumption of that particular allergen.

These lists have been produced and a short food frequency questionnaire compiled. We will be undertaking a validation exercise during the Spring and Summer of 2009. A nutrition student from Glasgow will validate the FFQ against a food diary in 50 parents of infants between 6 months and 1 year of age recruited from St Thomas' Hospital. The compliance FFQ covers monthly periods during the first year of the child's life so the validation exercise will compare the FFQ covering a 2 week period followed by an 8 day food diary (not weighed as we are not using the FFQ for details of macro or micronutrient intake) taken over the next two weeks. The FFQ is covering 2 weeks rather than one month as the infant's diet would be likely to change over a 6 week period (one month FFQ period and two week diary period). To help ensure that the food diary days are representative of what was eaten when the FFQ was filled in we will ask mothers in the validation study to continue to feed their infant in the same manner for the two weeks of the diary and not to introduce any new foods into the infant's diet.

5.3 MODIFICATION OR DISCONTINUATION OF STUDY TREATMENT

Participants in the intervention arm will discontinue consumption of an allergenic food if a confirmed allergic reaction to the allergenic food or an adverse nutritional consequence attributable to consumption of the allergenic food is experienced. They will continue to consume the other allergenic foods and will remain in the intervention arm of the study.

These participants will remain in the study to receive a status assessment at 3 years of age.

6. STUDY PROCEDURES

6.1 VISIT WINDOWS

- Visits described in the schedule of events (Appendix 3) should occur as follows:
- Visit 3 months: within +3 weeks of the planned visit date.
- Visit 12 months: ±2 months of the planned visit date.
- Interim compliance assessments:
  - Between visits 3 and 12: ±14 days.
  - Between visits 12 and 36: ±1 month.
- Visit 36 months: within ±3 months of the planned visit date.
- Unscheduled clinic visit: within +1 month of being notified of episode
- Food challenges: within +1 month of the decision being taken to undertake a challenge

6.2 GENERAL ASSESSMENTS

These general assessments will be performed as per the schedule of events (Appendix 3):

- Informed consent. Written informed consent will be obtained separately for the mother's and child's participation in the study.
- Randomization.
- Dietary education. Parents will be provided with written advice to assist with attaining the UK Government target of around six months exclusive breast feeding in the control arm and at least six months breast feeding in the intervention arm. Dieticians will provide written and verbal information and advice regarding the allergenic weaning diet or standard UK Government weaning advice for the intervention and control groups, respectively.
- Physical examination. Temperature, blood pressure, pulse, respiration.
- Medical history. A history will be taken to determine if the participant has had any clinically significant diseases or medical procedures other than the disease under study.
- Adverse events. Participants will be assessed for adverse events. All adverse events will be recorded on the case report forms (CRFs).
- Concomitant medications. All concomitant medications will be recorded on the CRFs.
- Dietary history. The dietary intake of the children will be formally evaluated at six months, 12 months and 3 years of age utilizing a five day food diary (three week days and a weekend) completed just prior to each time point. The 5-day food diary will capture typical food consumption and provide a breakdown of macro- and micro-nutrient intake and total energy intake.
- Food reaction history. A history will be taken to determine if the participant has had any clinically significant food-induced, immediate-onset allergic reactions.
- Eczema. Both subjective and objective eczema severity criteria will be recorded. The modified SCORAD evaluation will be used at all visits. Parents will also be asked to fill out the Patient-Oriented Eczema Measure (POEM) questionnaire.
- Skin barrier integrity – Trans-Epidermal Water Loss (TEWL) is a measurement which reflects the barrier properties of the stratum corneum and is increased when skin barrier function decreases. TEWL will be measured using the Aquaflux® closed chamber tewameter[66] and parents will be requested not to use any skin care products on their child's skin during the previous 24 hours. Measurements will be taken from uninvolved skin on the volar aspect of the forearm in a room maintained at constant temperature and humidity. Measurements will not be taken if the child is visibly distressed or crying.
- Rhinitis evaluation. Symptoms in accordance with the study definitions for seasonal and perennial rhinoconjunctivitis will be recorded.

- Asthma evaluation. Symptoms in accordance with the study definition for asthma will be recorded.
- Anthropometry. Skin fold thickness, weight and height and body mass index. Head circumference and abdominal circumference.
- Bioelectrical impedance *(subject to further funding)*. Results will be analyzed to determine fat and lean body mass.
- Dexa Scan *(subject to further funding)*. Dual energy x-ray absorptiometry (densitometry) will be performed to measure the amount of bone, muscle, and body fat.

6.3 QUESTIONNAIRE SCHEDULE

The schedule for questionnaire completion by parents participating in the study is outlined in Table 8.

Table 8. Questionnaire schedule

Assessment point	Subject	Duration	Place completed
20 weeks of pregnancy	Maternal diet and health	30 minutes	Antenatal clinic
34 weeks of pregnancy	Maternal diet	20 minutes	Home - online
**Post delivery**			
3 months	Infant health	10 minutes	St Thomas' allergy unit
4-12 months - monthly	Infant health and diet	20 minutes	Home - online
6 months	5 day infant food diary	1 hour	Home
One year assessment	5 day infant food diary	1 hour	St Thomas' allergy unit
15-35 months - 3 monthly	Infant health and diet	20 minutes	Home - online
Three year assessment	Infant health and diet	20 minutes	St Thomas' allergy unit
Three year assessment	5 day infant food diary	1 hour	Home

6.4 LABORATORY ASSESSMENTS

Ten millilitres of blood will be obtained at the 3 month and one year assessments and 20 mls at the three year assessment. These are small quantities of blood that will have no haemodynamic effect on the child. A local anaesthetic can be applied if necessary to minimise discomfort. Routine haematological and chemistry laboratory assessments, which are detailed below, will be performed at the investigation site as per the Schedule of Events (Appendix 3).

- Haematology includes FBC with WBC differential and platelets.
- Serum electrolytes including Ca, PO_4, urea, Cr, total protein, and albumin.
- Serum lipids include cholesterol, triglycerides, and high- and low-density lipoproteins.
- Fasting glucose and insulin.

6.5 ALLERGY ASSESSMENTS

6.5.1 Allergens Assessed

The following allergy assessments will be performed:

6.5.1.1 Skin prick tests

3 month visit

- Intervention group: SPT will be undertaken for the six intervention foods in duplicate: egg (salmonella free raw egg white), milk (pasteurized whole milk), sesame (tahini paste), fish, wheat and peanut. Total (including positive and negative controls: 14).
- Control group: no SPT will be done as the intention is that mothers wean their infants as they would have done if they had not been participating in the study

1 year visit (all infants)

- SPT for the six intervention foods: egg (salmonella free raw egg white), milk (pasteurized whole milk), sesame, fish, wheat and peanut. Other foods: soya and kiwi. Aero-allergens: house dust mite, cat, dog, 6 grass pollen mix and 3 tree pollen mix. Total (including positive and negative controls: 15).

3 year visit (all infants)

- SPT as per 1 year visit. Also tree nuts: Brazil nut, hazel nut, cashew, almond and walnut. Total (including positive and negative controls: 20).

6.5.1.2 Specific IgE measurements

All specific IgE measurements to the six intervention foods will be undertaken when a child has completed the study (at 3 years of age) (Section 1.3.5).

3 month visit (all infants)

- Sensitization to the six intervention foods.

1 year and 3 year visits

- Foods as per the 3 month visit and an aeroallergen panel including: *D. pteronyssinus*, *D. farinae*, cat and dog dander, horse dander, timothy grass, cladosporum, silver birch, olive, mugwort and nettle.

6.5.2 Skin Prick Testing: Procedures and Interpretation

Prior to testing, ensure that the participant has not received short-acting antihistamine medications for at least 48 hours and/or long-acting antihistamine medications for at least 7 days.

The SPT for raw hen's egg white will be performed using Red Lion salmonella-free egg, for milk will be performed with pasteurised whole milk and for sesame with tahini paste. The other SPTs will be performed using Soluprick® extracts (ALK-Abelló, where available). Positive (histamine 10 mg/ml) and negative control (50% glycerol, 50% buffered saline) solutions will be used.

Tests will be performed on the forearm unless unaffected eczema-free skin patches are not available, in which case the skin on the participants back will be used for testing. Using a standardized lancet (ALK Abelló), the skin will be pricked through a drop of the extract, which will then be absorbed. Skin test sites should be measured after 15 minutes. The wheal and flare should be measured at their widest diameters and its perpendicular and the mean measurement recorded.

The positive and negative control tests should be performed and measured prior to allergen SPT.

- If the negative control test is ≥3 mm, the testing should be rescheduled for approximately 7 days time.
- If the histamine positive control is ≤3 mm, then it should be repeated immediately. If the repeat test remains ≤3 mm, then the testing should be rescheduled for approximately 7 days' time.

For the food allergens tested at 3 months of age, the following rules apply:

- The SPTs will be performed in duplicate and the mean of the two tests will be recorded.
- If both results are ≥ 1 mm and there is a > 2 mm difference between the results, a third SPT will be performed and the mean of the two closest results will be recorded.
- If one result is < 1 mm and one result is > 1 mm, a third SPT will be performed. If two of three results are < 1 mm, 0 mm will be recorded as the final result. If two of three results are ≥ 1 mm, the mean of those two results will be recorded as the final result.

6.6 FOOD CHALLENGES

6.6.1 Scheduled Challenges

Infants in the intervention arm will undergo challenges at the 3 month screening visit if they have a positive skin prick test result to an allergenic food (Figure 6). All children will undergo a challenge at the 1 year and 3 year assessments if they have a positive skin prick reaction to one or more of the six intervention foods (Figure 9). The exception to this is sesame and peanut sensitisation at 1 year of age, for which challenges will be deferred until 3 years of age (see Section 3.6).

6.6.2 Unscheduled Challenges

Unscheduled challenges will be performed as indicated at unscheduled clinic visits (see section 3.5 and Figure 7 & 8). Independent of the result of these assessments, participants will continue with all subsequent study assessments. These participants will remain in the study to receive a status assessment at 3 years of age.

6.6.3 Repeat Challenges

If a participant fails to complete a challenge, he/she may be offered an opportunity to repeat the challenge at the investigator's discretion.

6.6.4 Challenges for non-intervention foods

Oral food challenges for allergenic foods other than the six foods in the intervention arm (e.g. kiwi) will be given when clinical history and the results of SPT and IgE for these allergens are inconclusive. These challenges will be performed according to standard clinical practice.

6.6.5 Procedure for challenges for suspected IgE mediated food allergy

6.6.5.1 Step 1: Perform Clinical Assessment

All children will be assessed by a paediatric allergy specialist to determine their suitability for a challenge. A participant's eligibility for a challenge is guided by the following criteria:

- The child has had no acute exacerbation of allergic signs or symptoms within the last week.

- The child has not received:
    - short-acting beta-2 agonists for 12 hours,
    - long-acting beta-2 agonists for 24 hours,
    - short-acting antihistamines in the last 48 hours, or
    - long-acting antihistamines in the last 7 days.
- The child has no concurrent illness.

Prior to conducting challenges, do the following:

- Inform PICU of the challenge taking place.
- Ensure that both oxygen and suction are in working order in the clinical trials unit.
- Ensure that all steps of the anaphylaxis protocol are in place and that all emergency drugs are prescribed.
- Check that the drug box containing emergency medications is complete and that it is readily available.
- Record baseline observations, including temperature, pulse, respiration, Sa02 and auscultation of the chest.
- Record blood pressure on all participants older than 12 months.
- Cannulate the following participants:
    - children with persistent asthma, and
    - children with a history of previous anaphylaxis to foods.
- Ensure that a child with a latex allergy or suspected latex allergy avoids all latex products

6.6.5.2 Step 2: Prepare the Food to be used in the Challenge

The dieticians will prepare the challenge foods and any carrier foods. The foods will be labelled and dated in the ward kitchen.

6.6.5.3 Step 3: Perform the Challenge

6.6.5.3.1 Double-blind, placebo-controlled challenge

Dose assessments and adjustments:

- Prior to the administration of each meal, the child will be evaluated for signs of reaction and vital signs (temperature, pulse, respiratory rate, blood pressure and SaO_2) will be monitored.
- The challenge should be discontinued at any stage if a protocol defined reaction occurs, and action will be taken according to local hospital guidelines.
- The meals will be blinded by a code known to the dietician but not to the participant, nurse, or doctor. After discussion with an investigator, a blinded dose may be repeated if any of the following occur:
    - abdominal pain
    - nausea
    - chest pain
    - abnormal oropharyngeal sensation
    - unexplained behavioral change

Perform a **mixed challenge** as follows:

Infant/Child consuming the food (2 grams of food protein or more twice a week regularly)

- Administer four doses of 0.5, 1.35, 2.0, and 4.0 g (if 3 years old) of allergenic food protein, interchangeably with the placebo challenge doses in 6-8 separate meals over the course of one day.

Infant/Child consuming the food infrequently or never

- Administer six doses of 0.1, 0.25, 0.5, 1.0, 2.0, and 4.0 g (if 3 years old) of allergenic food protein, interchangeably with the placebo challenge doses in 10-12 separate meals over the course of one day.

Both groups

- After each dose, observe the child for 20 minutes.
- An additional dose pair comprising a repeat of the previous dose and a placebo in random order may be given at the discretion of the investigator.

If an allergic reaction occurs following a placebo dose, perform a **separate challenge** as follows:

Day 1:

- Administer the same dose regime as above, all of which are either allergenic food protein or placebo.
- After each dose, observe the child for 20 minutes.

Day 2:

- Administer the same dose regime as above, all of which are either allergenic food protein or placebo. If allergenic food protein was administered on day 1, then administer placebo on day 2, or vice versa.
- After each dose, observe the child for 20 minutes.
- A dose may be repeated at the discretion of the investigator.

6.6.5.3.2 Open challenges (under 1 year of age)

Open challenges will be incremental as all infants having challenges will either be those sensitized at enrolment in the intervention arm (Figure 6) or with aversion and/or refusal in the intervention arm or a suspected allergic reaction (both arms) with a skin prick test response of <5mm (Figure 7).

Incremental open challenge (2.2g):

- Administer five doses of allergenic food protein of 0.1, 0.25, 0.5, 1.35, and 2.0 g (≥6 months) in separate meals

Allergenic food protein may vary and be used interchangeably.

6.6.5.4 Step 4: Determine the Outcome

Outcome of the challenge will be determined by evaluating the participant using the criteria in Table 9.

A positive food challenge will be defined by the presence of either of the following:

- One or more major criteria.
- Two or more minor criteria.

An indeterminate food challenge will be defined by the presence of one minor criterion.

A negative food challenge will be defined by the absence of major or minor criteria.

Table 9. Criteria for determining the outcome of food challenge

**Major Criteria**
Confluent erythematous pruritic rash
Respiratory signs (at least one of the following):<br>wheezing<br>inability to speak<br>stridor<br>dysphonia<br>aphonia
≥3 urticarial lesions
≥1 site of angioedema
Hypotension for age not associated with vasovagal episode
Evidence of severe abdominal pain (such as abnormal stillness or doubling over) that persists for ≥3 minutes
**Minor Criteria**
Vomiting
Diarrhoea
Persistent rubbing of nose or eyes that lasts for ≥3 minutes
Persistent rhinorrhoea that lasts for ≥3 minutes
Persistent scratching that lasts for ≥3 minutes

All symptoms should be of new onset and not due to ongoing disease. Symptoms must occur no later than 2 hours after the last dose.

6.6.5.5 Step 5: Consult with the Family

If the result is negative:

- Advise the family regarding consumption of the allergen with regard to their treatment arm and age.
- No emergency plan is required.

If the result is positive:

- Advise the family that the child must avoid the allergenic food in the diet.
- Advise the family regarding consumption of other allergenic foods with regard to their treatment arm and age.
- Provide a detailed written emergency management plan.
- Provide education on avoidance strategies.
- Provide training in Epi-Pen administration (once weight exceeds 7.5 kg)
- Review the child's inhaler technique if appropriate.
- Encourage the parents to join the UK Anaphylaxis Campaign and Medic-Alert.
- Schedule follow up appointment for the participant in an appropriate allergy clinic.

6.6.5.6 Step 6: Discharge the Participant

Observe the child until:

- 2 hours have elapsed since the top dose of the challenge.
- All symptoms have resolved (if the result was positive).
- The clinician confirms that the child is ready for discharge.

After the observation period is over, remove the cannula if one was installed.

6.6.6 Procedure for challenges for suspected non-IgE mediated food allergy

Parents who report symptoms suggestive of non-IgE mediated food allergy:

- Vomiting/recurrent posseting/reflux
- Recurrent diarrhoea/loose stools
- Blood in stools
- Worsening of eczema

AND the symptoms are **temporally related** to ingestion of allergenic foods (most likely to be cow's milk or egg) will be advised to take out the suspected allergen from the child's diet and the maternal diet if the mother is still breast feeding for a two week period. If this results in no change in the child's symptoms they will be advised to reintroduce the food. If the exclusion results in an improvement they will be asked to contact the paediatric allergy unit to arrange to be assessed and undertake a 10 day (5 days allergen, 5 days placebo) home based DBPCFC if appropriate (skin prick test negative to suspected allergen) (Figure 8).

The protocol used for these challenges is based on that used in EuroPrevall (with permission):

- Children attend the allergy unit for initial assessment and determination of their IgE skin prick status. Positive children follow the IgE mediated UCV algorithm.
- Skin prick negative children will consume the first dose (either allergen or placebo) in hospital to check the acceptability of the preparation to the child
- The parent/s will be given the remaining doses of the challenge to take home
- After each step (5 days of allergen or placebo) the child will be brought into the allergy unit and evaluated by the by the study doctor
- Parents will keep a symptom diary throughout the period of the DBPCFC
- Eczema status will be assessed using SCORAD and POEM at all three points (before, after initial 5 days of allergen/placebo and after 2nd five days of the allergen/placebo).

7. TOLERANCE ASSAYS

Subject to further funding the following would be conducted

7.1 FROZEN PBMC T-CELL ASSAY

7.1.1 Overview

Subject of further funding, cryobanked PBMCs will be used in assays monitoring functional changes in T cells. Samples will be cultured in vitro and stimulated with food antigens to see if there is antigen-specific lymphocyte proliferation and to measure cytokines secreted in response to these antigens. Cells from the stimulated cultures will be pelleted and banked for RNA extraction and the supernatants frozen for future secreted cytokine screening.

7.1.2 Gene Expression Profiling

Gene expression profiling will be performed using RNA isolated from non-stimulated versus in vitro stimulated cells. In this case, genes expressed in response to food and aeroallergen antigens will be compared in participants believed to be tolerant versus those who go on to develop food allergy. Genes of interest include IL-4, IL-5, IL-13, and IFNγ to determine if an allergic participant's T-cell responses are more T_H2-like; whereby a tolerant participant's T cells may respond to antigen in a more T_H1-like fashion. Alternatively, genes involved in mediating immune regulation by T regulatory cells may also be differentially expressed. Of particular interest is FoxP3, which will also be analyzed and directly compared to intracellular cytokine staining. Recently IRF-4, IRF-5, and IRF-7 have been shown to be associated with Toll-like receptor signalling and inflammatory responses. These, among other genes, are potential candidates for study.

7.1.3 Secreted Cytokines

Cytokines secreted by in vitro stimulated cells will be monitored with the Luminex platform, a multiplex assay currently allowing for detection of 30 cytokines and chemokines from very small volumes of cell culture supernatant or serum. This analysis will be compared with gene expression and ICS data to determine if cytokines produced by T cells are released and to examine the difference between secreted cytokine profiles of tolerant and allergic participants.

7.1.4 Antigen-specific Proliferative and Cytokine Responses

Antigen-specific proliferative and cytokine responses will be assessed using in vitro cultures with food and aeroallergens as described above. Using CFSE dye as a label, we will determine how many cell divisions have occurred in response to food and aeroallergens in vitro and determine the phenotype of proliferating cells with multicolor flow cytometry. Proliferative responses may be lower in tolerant versus allergic participants if active regulation is occurring for example. Additionally, intracellular cytokine production will be measured with ICS to determine profiles of cytokines secreted by the proliferating and nonproliferating T cells.

7.1.5 Regulatory T cells

Regulatory T cells play an important role in down-modulating active or inflammatory immune responses. In this case, early antigen exposure may induce tolerance by activating antigen specific regulatory T cells. Currently, regulatory T cells are isolated using CD4 and CD25 surface markers, specifically gating on $CD25^+$ hi cells. $CD4^+CD25^+$ cells are mixed with $CD4^+CD25^-$ cells to evaluate the impact of regulatory T cells on antigen-specific proliferative responses by $CD25^-$ cells. Again, if tolerance to food allergens is achieved, there may be increased regulatory T-cell activity. This assay is likely to evolve given the use of FoxP3 and potentially other markers for enumeration and isolation of regulatory T cells.

7.2 PLASMA ALLERGEN-SPECIFIC IGG AND IGE

7.2.1 ELISA-based Techniques

ELISA-based techniques will be used to measure food and aeroallergen-specific IgG4 and IgE during the study using cryobanked serum samples. Previous studies have shown that children who are sensitized but not allergic to specifically peanut have higher IgG4/IgE ratios than those who are allergic to peanut. Interestingly, the fact that both sensitized and allergic children are IgE positive suggests active regulation by T cells, which results in the higher IgG4 levels in the non-allergic sensitized patients. Higher IgG4/IgE ratios found in sensitized versus allergic patients should correlate with food allergen-induced tolerance in this study.

7.2.2 Facilitated Antigen Presentation Inhibition

Facilitated antigen presentation (FAP) inhibition is a flow cytometric-based assay that can detect participants who have allergen-specific IgG antibodies that interfere with FAP. In FAP, IgE facilitates the presentation of antigen to B cells, subsequently causing allergy-related, T-cell activation. Immunotherapy induces IgG, which competes for allergen-bound IgE, thus inhibiting allergen/IgE complexes from binding Fc receptors on antigen presentation cells (in this case, a B-cell line). Food allergen consumption will increase food allergen-specific IgG, especially IgG4, which interferes with FAP. The time course and magnitude of changes in plasma FAP inhibitory activity will be compared with clinical symptoms, clinical scores, and allergen-specific IgG levels.

7.3 WHOLE BLOOD DNA–HLA GENOTYPES

There are some indications that HLA class II genetic polymorphism may be associated with susceptibility to peanut allergy. DNA will be isolated from trial participants, subjected to sequence-based class II typing, and genotyped for potential SNPs associated with persons susceptible to allergic responses. DNA will also be analysed for skin barrier gene mutations.

8. ADVERSE EVENTS

8.1 OVERVIEW

Safety data will be recorded on a CRF specifically designed for this purpose. All serious adverse events (SAEs) will be reported on an SAE report form as well as on individual CRFs. All safety data will be reviewed periodically by the data monitoring committee (DMC). In addition, SAEs will be reported locally. The DMC has the authority to withdraw any participants and/or terminate the study because of safety findings.

Adverse events that are classified as serious according to the definition of health authorities must be reported promptly and appropriately to the DMC, the FSA, principal investigators in the trial, local ethics committee and any relevant NHS Trust. This section defines the types of adverse events and outlines the procedures for appropriately collecting, grading, recording, and reporting them.

8.2 INDEPENDENT DATA MONITORING COMMITTEE (IDMC)

Also known as the Data Safety Monitoring Board (DSMB).

8.2.1 Terms of reference

- To monitor and review ongoing data from the trial and undertake interim analyses if appropriate
- In the light of the above, and ensuring that the safety, rights and well being of the trial participants are paramount, to report (following each IDMC meeting) to the Trial Steering Committee and to recommend on the continuation of the trial
- To consider any requests for release of interim trial data and to recommend to the TSC on the advisability of this
- In the event of further funding being required, to provide to the TSC appropriate information and advice on the data gathered to date that will not jeopardise the integrity of the study.

8.2.2 Membership

Membership of the EAT Study IDMC has been organized by the FSA/MRC. As per MRC recommendations the membership consists of four individuals with expertise in the areas of paediatric allergy, paediatric research and trial management. One member is a trial experienced statistician.

8.2.3 Guidance notes

- The IDMC will meet at least annually, or more often as appropriate, and meetings will be timed so that reports can be fed into the Trial Steering Committee (TSC) meetings. Meetings will be called for and organised by the Chair of the IDMC. Dates for IDMC meetings will be agreed in advance and only altered with agreement of all members. The IDMC will communicate to the TSC chair to chair. Any oral communication will be backed up by written records.

- The trial statistician will prepare a comprehensive report for the DMC. This will be prepared and circulated well in advance of the meeting to allow DMC members time to study the data. Content of the report will be agreed in advance with the DMC Chair. The trial statistician may be invited by the Chair to attend part of the meeting to present the data; otherwise, no one involved with the trial or TSC will be present to see the unblinded data.
- A confidential report will be made in writing by the Chair of the DMC providing advice to the chair of the Trial Steering Committee on whether the trial should continue or not or be altered. If the DMC recommends that the trial should be stopped at any point, the TSC chair will inform the FSA/MRC.
- If at any stage an extension to the grant is needed the DMC may be requested by the TSC to provide information on the data gathered to date (from this and other studies) and advice on the likelihood that continuation of the trial will allow detection of an important effect. This will be done using methods that do not unblind the trial.
- Before reporting on the results of the trial the DMC will consider not only the interim results as presented by the trial statistician, but also any major new information from other sources thought to be relevant to the trial. It follows that the DMC will not automatically follow pre-assigned statistical rules, although it will be guided by statistical considerations.
- Information provided by the DMC is likely to fall into the following categories:

  (a) Information that might lead to the TSC stopping the trial prematurely in the event of a clear outcome, if this is deemed to be appropriate in the light of the accumulating data from the study, or on the basis of information available from other sources;

  (b) Information that might lead to the TSC modifying the design of the trial, if this is deemed to be appropriate in the light of the accumulating data from the study or on the basis of information available from other sources.

8.3 DEFINITIONS

8.3.1 Adverse Event

An adverse event is any occurrence or worsening of an undesirable or unintended sign, symptom, laboratory finding, or disease that occurs during participation in the trial.

An adverse event will be followed until it resolves or until 30 days after a participant terminates from the study, whichever comes first.

8.3.2 Serious Adverse Event

A Serious Adverse Event (SAE) is any untoward and unexpected medical occurrence or effect that:

- Results in death. A death that occurs during the study or that comes to the attention of the investigator during the protocol-defined follow-up after the completion of therapy must be reported whether it is considered treatment related or not.
- Is life-threatening – refers to an event in which, in the view of the investigator, the subject was at risk of death at the time of the event
- Requires inpatient hospitalization or prolongation of existing hospitalization.
- Results in persistent or significant disability or incapacity
- An event that requires intervention to prevent permanent impairment or damage. An important medical event that may not result in death, be life threatening, or require hospitalization may be considered an SAE when, based on appropriate medical judgment, it may jeopardize the participant and may require medical or surgical intervention to prevent one of the outcomes listed above.

Regardless of the relation of the adverse event to study participation, the event must be reported as a serious adverse event if it meets any of the above definitions.

8.3.3 Unexpected Adverse Event

An adverse event is considered "unexpected" when its nature (specificity) or severity is not consistent with applicable product information, such as safety information provided in the package insert, the investigator's brochure, or the protocol.

8.4 COLLECTING ADVERSE EVENTS

8.4.1 Methods of Collection

Adverse events will be collected from the time the infant is enrolled at 3 months of age until the time the event resolves or until 30 days after the participant completes the study, whichever comes first.

Adverse events may be discovered through any of these methods:

- Observing the participant at the 3 month, 12 month and 3 year visits.
- From responses to the parent completed compliance questionnaires
- From active reporting by the parent via the dedicated study help line (24 hour landline with answer-machine outside of office hours) or via the study email address.

An abnormal value or result from a clinical or laboratory evaluation (e.g., an X-Ray, an ultrasound, or an electrocardiogram) can also indicate an adverse event. If this is the case, then the evaluation that produced the value or result should be repeated until that value or result returns to normal or can be explained and the participant's safety is not at risk. If an abnormal value or result is determined by the investigator to be clinically significant, it must be recorded as an adverse event on the appropriate form(s).

8.4.2 Collecting Serious Adverse Events

Serious adverse events will be collected from the time the infant is randomized until 30 days after he/she completes study participation or until 30 days after he/she prematurely withdraws from the study.

8.4.3 Recording Adverse Events

Throughout the study, the investigator will record all adverse events on the appropriate adverse event CRF regardless of their severity or relation to study medication or study procedure. The investigator will treat participants experiencing adverse events appropriately and observe them at suitable intervals until their symptoms resolve or their status stabilizes.

8.4.4 Recording Serious Adverse Events

Serious adverse events will be recorded on the adverse event CRF and on the SAE form, and health authorities will be notified as outlined in section 8.6.2.

8.5 GRADING AND ATTRIBUTION OF ADVERSE EVENTS

8.5.1 Grading Criteria

The study site will grade the severity of adverse events experienced by study participants according to the criteria set forth in the National Cancer Institute - Common Terminology Criteria for Adverse Events (NCI-CTCAE) Version 3.0. This document provides a common language to describe levels of severity, to analyze and interpret data, and to articulate the clinical significance of all adverse events.

Adverse events will be graded on a scale from 1 to 5 according to the following standards in the NCI-CTCAE manual:

Grade 1 = mild adverse event.

Grade 2 = moderate adverse event.

Grade 3 = severe and undesirable adverse event.

Grade 4 = life-threatening or disabling adverse event.

Grade 5 = death.

All adverse events will be reported and graded whether they are or are not related to disease progression or treatment.

8.5.2 Attribution Definitions

The relation, or attribution, of an adverse event to study participation will be determined by the site investigator. The site investigator will also record the determination of attribution on the appropriate CRF and/or SAE reporting form. The relation of an adverse event to the study treatment will be determined using the descriptors and definitions provided in Table 9.

Table 10. NCI-CTCAE attribution of adverse events

Code	Descriptor	Definition
**Unrelated Category**		
1	Unrelated	The adverse event is clearly not related to study participation.
**Related Categories**		
2	Unlikely	The adverse event is doubtfully related to study participation.
3	Possible	The adverse event may be related to study participation.
4	Probable	The adverse event is likely related to study participation.
5	Definite	The adverse event is clearly related to study participation.

For additional information and a printable version of the NCI-CTCAE manual, consult the NCI-CTCAE web site: http://ctep.cancer.gov/reporting/ctc.html.

8.6 REPORTING ADVERSE/SERIOUS ADVERSE EVENTS

8.6.1 Reporting Timeline

Depending on the nature of the event the reporting procedures below will be followed. Any questions concerning adverse event reporting will be directed to the Chief Investigator in the first instance.

Non serious AEs

All such events, whether expected or not, will be recorded.

Serious AEs

An SAE form will be completed and faxed to the Chief Investigator within 24 hours.

8.6.2 Options for Reporting Serious Adverse Events

All SAEs will be reported to the Research Ethics Committee where in the opinion of the Chief Investigator, the event was:

- “related”, i.e. resulted from the administration of any of the research procedures; and

- “unexpected”, i.e. an event that is not listed in the protocol as an expected occurrence

Reports of related and unexpected SAEs will be submitted within 15 days of the Chief Investigator becoming aware of the event, using the COREC SAE form.

Local investigators will report any SAEs as required by the Local Research Ethics Committee and/or Research & Development Office.

In addition the following bodies will be informed:

- The IDMC
- The FSA/MRC

In the event of an unexpected death which is regarded as related or possibly related to the study, the researcher will notify the Coroner; even if a Coroner's post-mortem is not needed, a PM will be obtained wherever possible.

9. STATISTICS

9.1 ANALYSIS SAMPLES

The following groups will form samples for analysis.

- ***Intention to Treat analysis***

All children allocated to each arm of the intervention regimen will be analyzed together as representing that treatment arm, whether or not they received or completed the prescribed regimen. This will be done after the 3 year assessment for the period prevalence of IgE mediated food allergy to the six intervention foods between one and three years of age in both arms (Section 3.3.1).

- ***Per Protocol analysis***

Definition of compliance

The criteria for the per protocol analysis of the period prevalence of IgE mediated food allergy to the six intervention foods between one and three years of age in both arms (Section 3.3.1) will consist of:

Per protocol intervention arm compliance

Criterion A: Exclusive breast feeding for 3 months duration (water and/or oral rehydration solution would be an acceptable part of the diet).

Criterion B: Continued breast feeding up to 5 months of age

Criterion C: Consumption of at least five of the allergenic foods in at least 75% of the recommended amount, on at least 5 weeks between 3 months and 6 months of age.

Per protocol control arm compliance

Criterion A: Exclusive breast feeding for at least 3 months duration (water and/or oral rehydration solution would be an acceptable part of the diet).

Criterion B: Continued breast feeding up to 5 months of age

Criterion C: No consumption of peanut, egg, sesame, wheat or fish before 5 months

Criterion D: Cow's milk (or goat's milk) consumption of less than 300 mls/day after 3 months

9.2 ANALYSIS OF ENDPOINTS

9.2.1 Analysis of Primary Endpoint

The main analysis will compare the period prevalence of IgE mediated food allergy to the six intervention foods between one and three years of age in both arms (Section 3.3.1) using a two-tailed, chi-square test at the $p<0.05$ level of significance.

9.2.2 Analysis of Secondary Endpoints

The incidence of adverse events and laboratory abnormalities at three years of age will be compared between the intervention and control arms using a two-tailed chi-square test at the $p<0.05$ level of significance. Additional specific secondary endpoints are described in section 3.4.

9.2.3 Additional Analyses

Additional analyses of the risk associated with IgE-mediated food allergy will be performed using multiple logistic regression to identify possible associations of risk with baseline participant characteristics and selected clinical and dietary measures. These analyses will be performed on the incidence of IgE-mediated food allergy at three years of age.

9.3 SAMPLE SIZE

The trial has 90% power to detect a 50% reduction (based on a 2-tailed test) in the absolute prevalence of food allergy by three years of age (from an expected prevalence of 6% in the control group to 3% in the intervention group).

The study is also designed so that it has sufficient power to detect a protective effect in the high risk sub-population. The study has 95% power to detect a reduction from 30% to 10% amongst the 100 (10%) high risk children in the intervention group (defined as those developing moderate or severe eczema in the first year of life) compared with the 100 (10%) high risk children in the control group. However the power is still respectable to detect a 50% reduction in prevalence of food allergy, with 71% power to detect a reduction from 30% to 15%.

The study is not powered to detect a reduction in allergy to individual foods.

9.4 LOSS TO FOLLOW-UP

The antenatal recruitment target of 3000 mothers has been determined in order to allow for a loss of 500 women (16.7%) between maternal consent to participate at 20 weeks of pregnancy and her infant attending the 3 month assessment visit. Of the 2500 infants who are then enrolled, it has been estimated that 20% will have been lost to follow-up by the time of the 3 year final assessment. Thus we estimate that approximately 2200 will attend the 1 year interim assessment visit and 2000 the 3 year final assessment clinic.

The 20% estimate is derived from several sources: In the AllergyFlora study (based in a central London teaching hospital with a similar population) the loss to follow-up between enrolment at birth and 18 months of age was 15.6%. The 20% estimate is also identical to the loss to follow-up observed in the much larger German MAS study, where at the 36 month (3 year) assessment the participation rate was 1050/1314 children (79.9%). In addition, in our immunotherapy intervention studies done by our team which involve regular injections over a 2-3 year period, we have had only a 10% drop-out rate with patients obtained from across the London area.

9.5 RANDOMIZATION, STRATIFICATION, AND BLINDING

Participants will be randomly assigned to treatment using a centrally administered randomization scheme. The randomization will not be stratified given the number of participants. The study participants are unblinded with regard to the intervention. However, assessments such as eczema status will be undertaken by physicians blind to the child's treatment allocation. Also, the study outcomes are based on objective measures of sensitization (skin prick test results which will be measured by study personnel blinded to the child's allocation status) and DBPCFC.

9.6 REPORTING DEVIATIONS FROM THE ORIGINAL STATISTICAL PLAN

The principal features of the study design and the plan for statistical analysis of the data are outlined in this protocol. Any changes in these features will require a protocol amendment which will be subject to review by the IDMC and the study sponsor. These changes will be described in the final report as appropriate.

10. IDENTIFICATION AND ACCESS TO SOURCE DATA

10.1 IDENTIFYING SOURCE DATA

The investigator is required to keep accurate records to ensure that the conduct of the study is fully documented (see section 11). The results of all clinical and clinical laboratory evaluations will be maintained in the participant's medical records and the data will be transferred to clinical CRFs.

Safety data will be recorded on CRFs specifically designed for this purpose. All the SAEs will be reported on an SAE report form as well as on individual CRFs. All data will be reviewed periodically by the IDMC. The IDMC has the authority to withdraw any participants and/or terminate the study because of safety findings.

10.2 PERMITTING ACCESS TO SOURCE DATA

The investigational site participating in this study will maintain the highest degree of confidentiality permitted for the clinical and research information obtained from the participants in this clinical trial. Medical and research records should be maintained at each site in the strictest confidence. However, as a part of the quality assurance and legal responsibilities of an investigation, the investigational site must permit authorized representatives of the sponsor(s) and health authorities to examine (and when required by applicable law, to copy) clinical records for the purpose of quality assurance reviews, audits, and evaluations of the study safety and progress. Unless required by the laws that permit copying of records, only the coded identity associated with documents or with other participant data may be copied (and all personally identifying information must be obscured). Authorized representatives as noted above are bound to maintain the strict confidentiality of medical and research information that is linked to identified individuals. The investigational site will normally be notified before auditing visits occur.

11. QUALITY CONTROL AND QUALITY ASSURANCE

The investigator is required to keep accurate records to ensure that the conduct of the study is fully documented.

Audits and Inspections

The study may be subject to inspection and audit by Kings College London, under their remit as sponsor and other regulatory bodies to ensure adherence to ICH/MRC Principles of Good Clinical Practice[1, 2] and the NHS Research Governance Framework for Health and Social Care (2nd edition).[67]

11.1 MANAGEMENT STRUCTURE

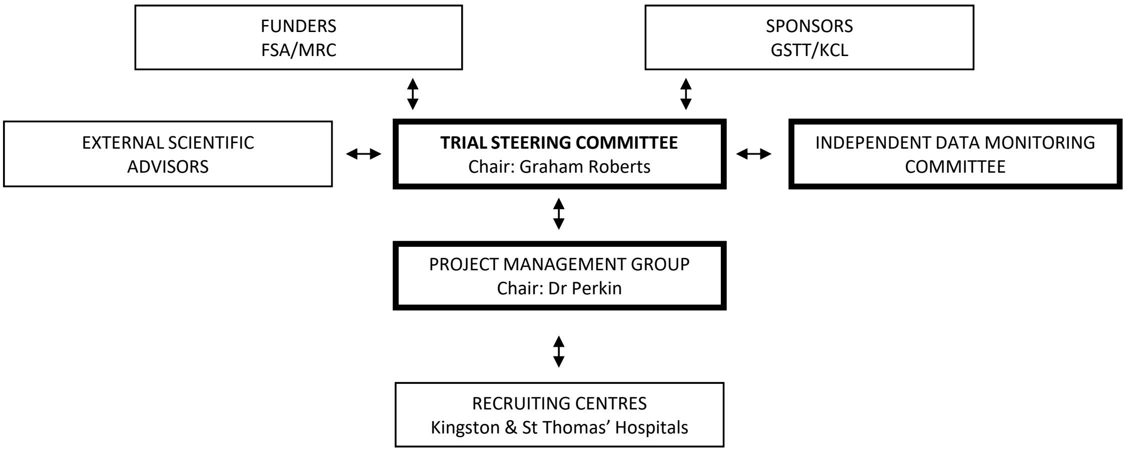

FIGURE 10. MANAGEMENT STRUCTURE

Introduction

The overall management ideals for the EAT Study are:

- That there will be fair representation of all members of the project on the decision making bodies of the project.
- That the management of the study will be completely transparent to its members, and to the FSA and MRC.
- That management will be flexible and responsive to the need for change as the project develops its science base.
- That management will be disciplined and effective.

Management structure (see Figure 10)

The Early Weaning Trial will be co-coordinated by Dr Perkin and Professor Lack. Professor Lack will be the designated co-ordinator for communication with the FSA. Dr Perkin will chair the Project Management Group.

The overall management structure consists of:

- Trial Steering Committee (TSC)
- Project (Trial) Management Group (PMG)
- Independent Data Monitoring Committee (IDMC)

The main roles, responsibilities, composition and meeting frequency of these bodies are described below.

11.1.1 Trial Steering Committee

The TSC is the main decision making body. It has overall responsibility for scientific strategy and direction and has ultimate responsibility for ensuring the project's aims are delivered on time and within budget.

The TSC will include external representatives with appropriate expertise including paediatric nutrition and the design of robust methodologies for collecting dietary data. The members are:

Independent members (voting)

Dr Graham Roberts, Reader in Paediatric Allergy, Southampton University (Chair)

Professor David Strachan, St George's University of London (Vice Chair)

Professor Christine Edwards, University of Glasgow

Mr David Reading, Technical Director, Anaphylaxis Campaign

Dr Mary Fewtrell, Reader in Childhood Nutrition, UCL Institute of Child Health

Dependent Members (voting)

Professor Gideon Lack, Kings College London

Dr Michael Perkin, Kings College London

Professor Anne Greenough, Kings College Hospital

Professor Andy Grieve, Kings College London (Study Statistician)

Professor Ian Kimber, Professor of Toxicology, University of Manchester (on behalf of FSA)

Dr Carsten Flohr, Kings College London (non -voting)

Observers (non-voting)

Ms Sarah Hardy, Food Standards Agency

Ms Joelle Buck, Food Standards Agency

Dr Kirsty England, EAT Study Coordinator

The major roles of the TSC will be:

- To make decisions necessary to ensure successful delivery of the EAT Study.
- To evaluate progress against the agreed timetable and deliverables.
- To administer the budget and monitor spending.

- To develop and implement successful communication between the study staff and external stakeholders (recruitment centres, funders and sponsors).
- To make decisions regarding the allocation and further analyses of biological samples.

The FSA/MRC will be notified of any significant changes to protocol or decisions taken by the TSC that may affect the progress or delivery of the project. In addition the TSC will also be responsible for:

- Development, implementation and evaluation of appropriate policies and procedures to facilitate the protection of knowledge and exploitation of results.
- In consultation with the FSA and MRC as funding bodies, development, implementation and evaluation of appropriate policies and procedures to ensure the effective dissemination of results to appropriate stakeholders.

The TSC will meet as scheduled in the Scope of Work document.

11.1.2 Project (Trial) Management Group

The PMG is responsible for the day to day management of the Early Weaning Trial.

The main roles of the PMG will be:

- To ensure that the goals set by the Trial Steering Committee are met.
- Manage the day to day running of the project.
- Record project activity and monitor that the study milestones are met and that the deliverables are delivered.
- Monitor spending on the study
- Arrange annual financial audit of spending.
- Ensure filing of all documentation accompanying the study arrangement and finances.
- Prepare reports for the Trial Steering Committee and FSA.
- Organise all meetings and provide administrative support to the TSC chairperson.
- Implementation of the study communication strategy.

The PMG will be chaired by Dr Michael Perkin and include Professor Lack and the study manager. The PMG will convene weekly to discuss all operational business. The study also benefits from a full time project manager on another intervention study so the two project managers will be able to support each other (e.g. whilst on annual leave).

11.1.3 Independent Data Monitoring Committee

The study has a completely independent DMC (see Section 8.2).

11.1.4 External Scientific Advisors

The TSC will seek advice when necessary from individuals who are leading international authorities in areas that can contribute to the design, implementation and analysis of the study. Individual members may be identified to attend specific steering group meetings when appropriate.

11.2 DATA HANDLING

Data Collection

All data will be collated centrally, coordinated by the project manager in the project office. Registration forms will be faxed to the project office via the dedicated study fax line. Transcription of all data from paper records to computer will be done independently by two separate people (double entered) to ensure that transcription errors are avoided.

Data Monitoring (including compliance)

CRFs will be monitored for data accuracy by auditing a random sample of up to 10 at Steering Committee meetings. Staff will receive training in the completion of CRF's, fulfilling the clinical trial protocol and training in Good Clinical Practice.

Data Storage

Documents will be stored within a locked cupboard within a locked room. Documents will be maintained in a legible condition, with prompt retrieval possible.

Sample tracking

We will use a bar code based tracking system for sample management. This specialised software system is designed to track where individual samples are for particular patients. This is invaluable for a multi-sampling large study such as this. The individual aliquots of cells, plasma or serum can be tracked so that the exact number of cells or vials left at any particular time point is known.

Archiving

Essential documents will be archived in an easily accessible way and readily available on request. All essential documents will be boxed and clearly labelled with the following information:

- Project/trial name
- Reference Number
- Site Number (if applicable)
- Chief Investigator
- Lead Site
- Date of Archive

The documents will be stored in a secure room, with appropriate environmental controls (and adequate protection from fire, without water sprinkler systems, water etc.), and access only by authorised personnel. All trial related documents will be centrally archived to prevent accidental damage, amendment, loss or destruction. Any change in the ownership and location of documentation will be documented in order to allow the tracking of the stored records. If archived documents are reviewed at a later date, we will record who and what documents were reviewed and the date they were accessed in an archive index/log.

All trial-related documents will be kept for a minimum of 15 years. For general clinical research, essential documents will be kept for a minimum of 10 years. Once this time period has elapsed, a destruction log will be kept for a further 5 years, listing everything that has been destroyed and the reasons why it was destroyed.

12. ETHICAL CONSIDERATIONS AND COMPLIANCE WITH GOOD CLINICAL PRACTICE

12.1 STATEMENT OF COMPLIANCE

We will adhere to the general principles and standards in research as outlined by the General Medical Council in the document: Research: The Role and Responsibilities of Doctors (Feb 2002).

We will adhere to the principles of Good Clinical Practice in Clinical Research produced by the ICH and endorsed by the MRC.[1, 2]

This clinical study will also be conducted in accordance with the FSA Joint Code of Practice for Research.

Ethical approval for the study will also be obtained. All relevant trusts within the National Health System in England will also be informed of the study.

12.2 INFORMED CONSENT AND ASSENT

Consent to enter the study will be sought from each participant only after a full explanation has been given, an information leaflet offered and time allowed for consideration. All participants (or their legally acceptable representative) must read, sign, and date a consent form before entering the study or undergoing any study-specific procedures. The right of the participant to refuse to participate without giving reasons will be respected. All participants are free to withdraw at any time from the protocol treatment without giving reasons and without prejudicing further treatment.

The informed consent form must be revised whenever important new safety information is available, whenever the protocol is amended, and/or whenever any new information becomes available that may affect participation in the trial.

12.3 PRIVACY AND CONFIDENTIALITY

A participant's privacy and confidentiality will be respected throughout the study. Each participant will be assigned a sequential identification number, and these numbers rather than names will be used to collect, store, and report participant information.

13. PUBLICATION POLICY

The co-principal investigators in consultation with the FSA and MRC will produce a publication policy for collaborators and funders to sign with respect to subsidiary studies that may be performed on the cohort.

14. References

1. ICH. Harmonised Tripartitie Guideline for good clinical practice E6(R1). 1996.

2. Medical Research Council. MRC guidelines for good clinical practice in clinical trials. 1998.

3. Thong BY, Hourihane JO. Monitoring of IgE-mediated food allergy in childhood. Acta Paediatr 2004; 93(6):759-764.

4. Asher MI, Montefort S, Bjorksten B et al. Worldwide time trends in the prevalence of symptoms of asthma, allergic rhinoconjunctivitis, and eczema in childhood: ISAAC Phases One and Three repeat multicountry cross-sectional surveys. Lancet 2006; 368(9537):733-743.

5. World Health Organization. Global Strategy for Infant and Young Child Feeding. 2002. Geneva.

6. Department of Health. Infant Feeding Recommendation. 2003.

7. Department of Health. Weaning - starting solid food. 2008.

8. Bergmann RL, Diepgen TL, Kuss O et al. Breastfeeding duration is a risk factor for atopic eczema. Clin Exp Allergy 2002; 32(2):205-209.

9. Sears MR, Greene JM, Willan AR et al. Long-term relation between breastfeeding and development of atopy and asthma in children and young adults: a longitudinal study. Lancet 2002; 360(9337):901-907.

10. Zutavern A, von Mutius E, Harris J et al. The introduction of solids in relation to asthma and eczema. Arch Dis Child 2004; 89(4):303-308.

11. Burr ML, Butland BK, King S, Vaughan-Williams E. Changes in asthma prevalence: two surveys 15 years apart. Arch Dis Child 1989; 64(10):1452-1456.

12. COMA working group on the weaning diet. Weaning and the weaning diet. 1994. London.

13. Grundy J, Matthews S, Bateman B, Dean T, Arshad SH. Rising prevalence of allergy to peanut in children: Data from 2 sequential cohorts. J Allergy Clin Immunol 2002; 110(5):784-789.

14. Committee on Nutrition. Hypoallergenic Infant Formulas. Pediatrics 2000; 106(2):346-349.

15. Greer FR, Sicherer SH, Burks AW. Effects of early nutritional interventions on the development of atopic disease in infants and children: the role of maternal dietary restriction, breastfeeding, timing of introduction of complementary foods, and hydrolyzed formulas. Pediatrics 2008; 121(1):183-191.

16. Agostoni C, Decsi T, Fewtrell M et al. Complementary feeding: a commentary by the ESPGHAN Committee on Nutrition. J Pediatr Gastroenterol Nutr 2008; 46(1):99-110.

17. Kramer MS, Kakuma R. Optimal duration of exclusive breastfeeding. Cochrane Database Syst Rev 2002;(1):CD003517.

18. Falth-Magnusson K, Kjellman NI. Development of atopic disease in babies whose mothers were receiving exclusion diet during pregnancy--a randomized study. J Allergy Clin Immunol 1987; 80(6):868-875.

19. Falth-Magnusson K, Kjellman NI. Allergy prevention by maternal elimination diet during late pregnancy--a 5-year follow-up of a randomized study. J Allergy Clin Immunol 1992; 89(3):709-713.

20. Hattevig G, Kjellman B, Sigurs N, Grodzinsky E, Hed J, Bjorksten B. The effect of maternal avoidance of eggs, cow's milk, and fish during lactation on the development of IgE, IgG, and IgA antibodies in infants. J Allergy Clin Immunol 1990; 85(1 Pt 1):108-115.

21. Herrmann M-E, Dannemann A, Grüters A et al. Prospective study on the atopy preventive effect of maternal avoidance of milk and eggs during pregnancy and lactation. European Journal of Pediatrics 1996; 155(9):770-774.

22. Zeiger RS, Heller S. The development and prediction of atopy in high-risk children: follow-up at age seven years in a prospective randomized study of combined maternal and infant food allergen avoidance. J Allergy Clin Immunol 1995; 95(6):1179-1190.

23. Hide DW, Matthews S, Tariq S, Arshad SH. Allergen avoidance in infancy and allergy at 4 years of age. Allergy 1996; 51(2):89-93.

24. Saarinen UM, Kajosaari M. Does dietary elimination in infancy prevent or only postpone a food allergy? A study of fish and citrus allergy in 375 children. Lancet 1980; 1(8161):166-167.

25. Kajosaari M. Atopy prophylaxis in high-risk infants. Prospective 5-year follow-up study of children with six months exclusive breastfeeding and solid food elimination. Adv Exp Med Biol 1991; 310:453-458.

26. Fergusson DM, Horwood LJ, Shannon FT. Early solid feeding and recurrent childhood eczema: a 10-year longitudinal study. Pediatrics 1990; 86(4):541-546.

27. Mihrshahi S, Ampon R, Webb K et al. The association between infant feeding practices and subsequent atopy among children with a family history of asthma. Clin Exp Allergy 2007; 37(5):671-679.

28. Sariachvili MA, Droste JHJ, Dom S et al. Early exposure to solid foods and the development of eczema in children up to 4 years of age. Pediatr Allergy Immunol. In press.

29. Poole JA, Barriga K, Leung DY et al. Timing of initial exposure to cereal grains and the risk of wheat allergy. Pediatrics 2006; 117(6):2175-2182.

30. Alm B, Aberg N, Erdes L et al. Early introduction of fish decreases the risk of eczema in infants. Arch Dis Child 2009; 94(1):11-15.

31. Snijders BE, Thijs C, van RR, van den Brandt PA. Age at first introduction of cow milk products and other food products in relation to infant atopic manifestations in the first 2 years of life: the KOALA Birth Cohort Study. Pediatrics 2008; 122(1):e115-e122.

32. Du Toit G., Katz Y, Sasieni P et al. Early consumption of peanuts in infancy is associated with a low prevalence of peanut allergy. J Allergy Clin Immunol 2008; 122(5):984-991.

33. Husby S, Mestecky J, Moldoveanu Z, Holland S, Elson CO. Oral tolerance in humans. T cell but not B cell tolerance after antigen feeding. J Immunol 1994; 152(9):4663-4670.

34. Meglio P, Bartone E, Plantamura M, Arabito E, Giampietro PG. A protocol for oral desensitization in children with IgE-mediated cow's milk allergy. Allergy 2004; 59(9):980-987.

35. Clark AT, Islam S, King Y, Deighton J, Anagnostou K, Ewan PW. Successful oral tolerance induction in severe peanut allergy. Allergy 2009.

36. Kerosuo H, Kullaa A, Kerosuo E, Kanerva L, Hensten-Pettersen A. Nickel allergy in adolescents in relation to orthodontic treatment and piercing of ears. Am J Orthod Dentofacial Orthop 1996; 109(2):148-154.

37. Mortz CG, Lauritsen JM, Bindslev-Jensen C, Andersen KE. Nickel sensitization in adolescents and association with ear piercing, use of dental braces and hand eczema. The Odense Adolescence Cohort Study on Atopic Diseases and Dermatitis (TOACS). Acta Derm Venereol 2002; 82(5):359-364.

38. Van Hoogstraten IM, Andersen KE, Von Blomberg BM et al. Reduced frequency of nickel allergy upon oral nickel contact at an early age. Clin Exp Immunol 1991; 85(3):441-445.

39. Twarog FJ, Weinstein SF, Khaw KT, Strieder DJ, Colten HR. Hypersensitivity to pancreatic extracts in parents of patients with cystic fibrosis. J Allergy Clin Immunol 1977; 59(1):35-40.

40. Strobel S. Dietary manipulation and induction of tolerance. J Pediatr 1992; 121(5 Pt 2):S74-S79.

41. Friedman A. Induction of anergy in Th1 lymphocytes by oral tolerance. Importance of antigen dosage and frequency of feeding. Ann N Y Acad Sci 1996; 778:103-110.

42. Frossard CP, Tropia L, Hauser C, Eigenmann PA. Lymphocytes in Peyer patches regulate clinical tolerance in a murine model of food allergy. J Allergy Clin Immunol 2004; 113(5):958-964.

43. Strid J, Thomson M, Hourihane J, Kimber I, Strobel S. A novel model of sensitization and oral tolerance to peanut protein. Immunology 2004; 113(3):293-303.

44. Quigley MA, Kelly YJ, Sacker A. Infant feeding, solid foods and hospitalisation in the first 8 months after birth. Arch Dis Child 2008.

45. Bolling K, Grant C, Hamlyn B, Thornton A. Infant feeding survey 2005. 2005. The Information Centre for health and social care and the UK Health Departments.

46. Davies DP. Plasma osmolality and feeding practices of healthy infants in first three months of life. Br Med J 1973; 2(5862):340-342.

47. Taitz LS. Infantile overnutrition among artificially fed infants in the Sheffield region. Br Med J 1971; 1(5744):315-316.

48. Hornell A, Hofvander Y, Kylberg E. Solids and formula: association with pattern and duration of breastfeeding. Pediatrics 2001; 107(3):E38.

49. Nowak-Wegrzyn A, Bloom KA, Sicherer SH et al. Tolerance to extensively heated milk in children with cow's milk allergy. J Allergy Clin Immunol 2008; 122(2):342-7, 347.

50. Thomas K, Herouet-Guicheney C, Ladics G et al. Evaluating the effect of food processing on the potential human allergenicity of novel proteins: international workshop report. Food Chem Toxicol 2007; 45(7):1116-1122.

51. Kulig M, Bergmann R, Klettke U, Wahn V, Tacke U, Wahn U. Natural course of sensitization to food and inhalant allergens during the first 6 years of life. J Allergy Clin Immunol 1999; 103(6):1173-1179.

52. Martinez FD, Wright AL, Taussig LM, Holberg CJ, Halonen M, Morgan WJ. Asthma and wheezing in the first six years of life. The Group Health Medical Associates. N Engl J Med 1995; 332(3):133-138.

53. Sears MR, Greene JM, Willan AR et al. A longitudinal, population-based, cohort study of childhood asthma followed to adulthood. N Engl J Med 2003; 349(15):1414-1422.

54. Illi S, von ME, Lau S, Niggemann B, Gruber C, Wahn U. Perennial allergen sensitisation early in life and chronic asthma in children: a birth cohort study. Lancet 2006; 368(9537):763-770.

55. Morgan WJ, Stern DA, Sherrill DL et al. Outcome of asthma and wheezing in the first 6 years of life: follow-up through adolescence. Am J Respir Crit Care Med 2005; 172(10):1253-1258.

56. Venter C, Pereira B, Grundy J et al. Incidence of parentally reported and clinically diagnosed food hypersensitivity in the first year of life. J Allergy Clin Immunol 2006; 117(5):1118-1124.

57. Lack G, Fox D, Northstone K, Golding J. Factors associated with the development of peanut allergy in childhood. N Engl J Med 2003; 348(11):977-985.

58. Sicherer SH, Burks AW, Sampson HA. Clinical features of acute allergic reactions to peanut and tree nuts in children. Pediatrics 1998; 102(1):e6.

59. Sicherer SH, Furlong TJ, Munoz-Furlong A, Burks AW, Sampson HA. A voluntary registry for peanut and tree nut allergy: characteristics of the first 5149 registrants. J Allergy Clin Immunol 2001; 108(1):128-132.

60. Norris JM, Barriga K, Hoffenberg EJ et al. Risk of celiac disease autoimmunity and timing of gluten introduction in the diet of infants at increased risk of disease. JAMA 2005; 293(19):2343-2351.

61. Kunz B, et al. Clinical validation and guidelines for the SCORAD index: consensus report of the European Task Force on Atopic Dermatitis. Dermatology 1997; 195:10-19.

62. Charman CR, Venn AJ, Williams HC. The patient-oriented eczema measure: development and initial validation of a new tool for measuring atopic eczema severity from the patients' perspective. Arch Dermatol 2004; 140(12):1513-1519.

63. Sporik R, Hill DJ, Hosking CS. Specificity of allergen skin testing in predicting positive open food challenges to milk, egg and peanut in children. Clin Exp Allergy 2000; 30(11):1540-1546.

64. Hill DJ, Heine RG, Hosking CS. The diagnostic value of skin prick testing in children with food allergy. Pediatr Allergy Immunol 2004; 15(5):435-441.

65. WHO. Indicators for assessing infant and young child feeding practices: conclusions of a consensus meeting held 6-8 November 2007 in Washington D.C., USA. 2008. WHO Press.

66. Nikolovski J, Stamatas GN, Kollias N, Wiegand BC. Barrier function and water-holding and transport properties of infant stratum corneum are different from adult and continue to develop through the first year of life. J Invest Dermatol 2008; 128(7):1728-1736.

67. Department of Health. Research governance framework for health and social care (2nd edtn). 2005.

Appendix 1. Study Overview Chart

EAT (*Enquiring About Tolerance*) Study Overview

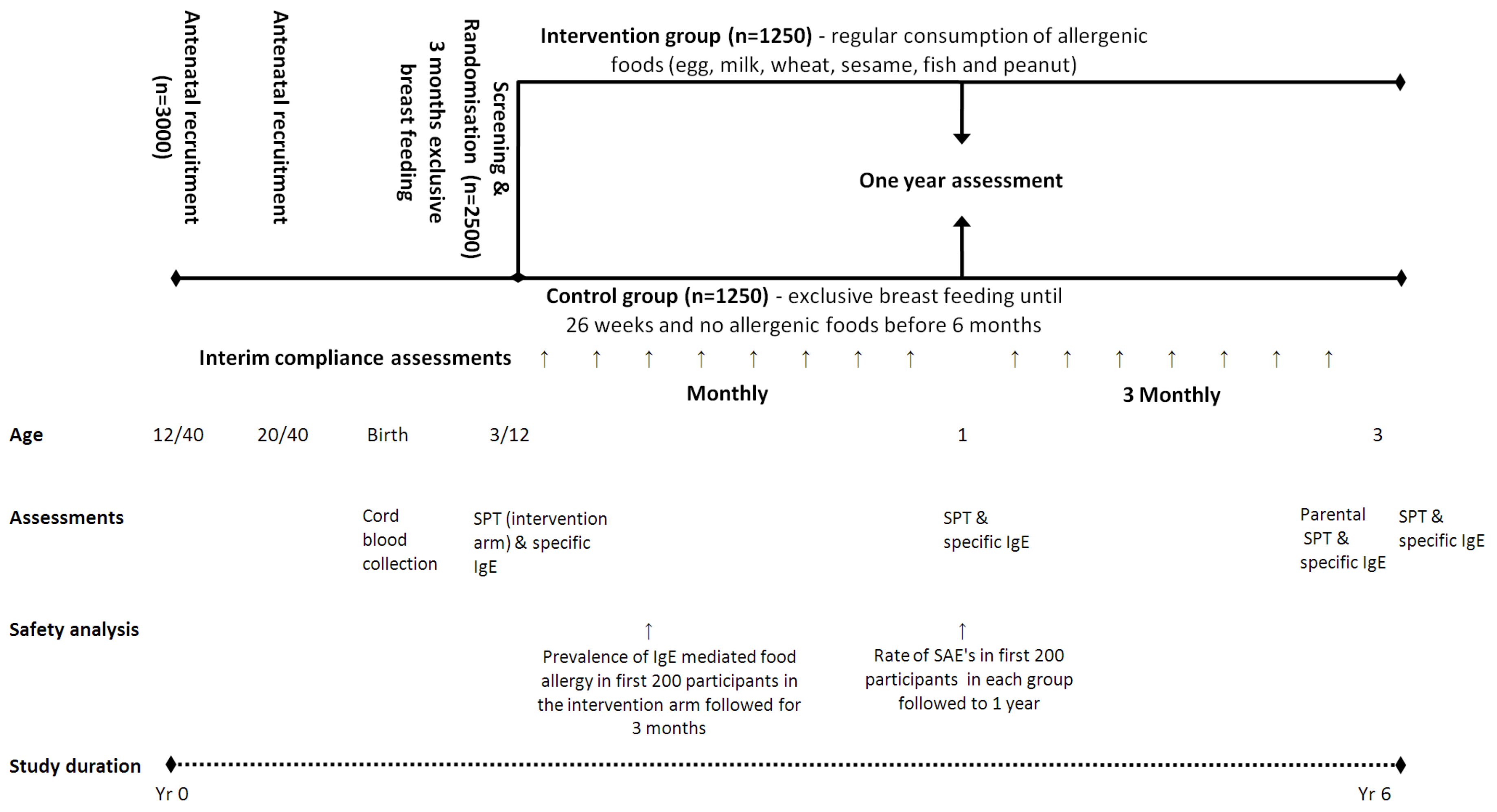

Appendix 2. Study Gantt Chart

Gantt chart: T07051 Randomized controlled trial of early introduction of allergenic foods to induce tolerance in infants

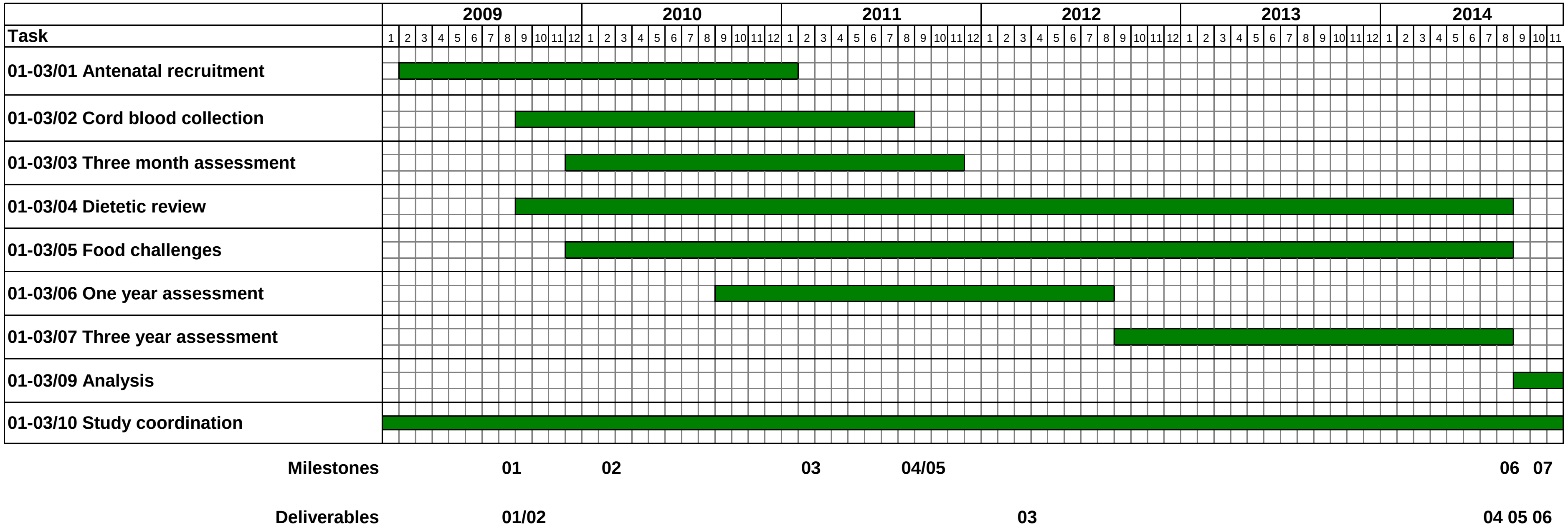

Appendix 3. Schedule of Events

Age	12/40	20/40	34/40	0	3/12 Initial assessment	4-11/12 Monthly	One year assessment	15-33/12 3 Monthly	36/12 Final assessment	Unscheduled visit assessment
Invitation & information	X									
Informed consent		Maternal			Infant					
General Assessments										
Physical examination					X		X		X	X
Medical history		X	X		X	X	X	X	X	X
Adverse events						X	X	X	X	X
Concomitant medications			X		X	X	X	X	X	X
TEWL measurement					X		X		X	
Eczema evaluation					X	X	X	X	X	
Rhinitis evaluation							X		X	
Asthma evaluation					X		X		X	
Anthropometry					X	X	X	X	X	
Laboratory assessments										
Haematology					X		X		X	
Serum chemistries					X		X		X	
Serum lipids									X	
Fasting glucose and insulin									X	
Allergy assessments										
Skin prick testing (parental)									Parental	
Specific IgE (parental)									Parental	
Skin prick testing (child)					Intervention foods*		Extended panel†		Extended panel‡	X
Specific IgE (child)					Intervention foods		Extended panel§		Extended panel§	X
Diet										
Dietary education					X	X	X	X		X
FFQ		Maternal	Maternal		Maternal					
5 day food diary						6/12	X		X	
Food reaction history						X	X	X	X	X
Compliance assessment						X	X	X	X	X
Immunologic assessments *(subject to funding)*										
Frozen PBMC T-cell assay				X	X		X		X	
Plasma allergen specific IgG and IgE				X	X		X		X	
Whole blood DNA-HLA haplotypes				X	X				X	

* Intervention arm only

† Intervention foods and soya and kiwi. Aero-allergens: house dust mite, cat, dog, 6 grass pollen mix and 3 tree pollen mix

‡ As per 1 year visit. Also tree nuts: Brazil nut, hazel nut, cashew, almond and walnut.

§ Intervention foods and an aeroallergen panel including: D. pteronyssinus, D. farinae, cat and dog dander, horse dander, timothy grass, cladosporum, silver birch, olive, mugwort and nettle
