# Supplementary material for: Association of Early Introduction of Solids With Infant Sleep: A Secondary Analysis of a Randomized Clinical Trial
Source: JAMA Pediatr. 2018 Jul 9;172(8):e180739. doi: 10.1001/jamapediatrics.2018.0739 (PMC6142923; doi:10.1001/jamapediatrics.2018.0739)
Supplement: Supplement 3. — Trial Protocol. Final protocol. [file jamapediatr-172-e180739-s003.pdf]

Protocol (T07051)

Randomized controlled trial of early introduction of allergenic foods to induce tolerance in infants

Short Title: Enquiring About Tolerance (EAT) Study

Version 5.00 (19th March, 2014)

This clinical study is supported by the Food Standards Agency and the Medical Research Council.

PRINCIPAL INVESTIGATOR
Gideon Lack, MD
Professor of Paediatric Allergy
Children's Allergies Department
2nd Floor, Stairwell B, South Wing
St Thomas' Hospital
Lambeth Palace Road
London SE1 7EH
Phone: 020 7188 9730
Fax: 020 7188 9782
E-mail: gideon.lack@kcl.ac.uk

CO-PRINCIPAL INVESTIGATOR
Dr Michael Perkin, PhD
Senior Lecturer in Paediatric Allergy
Children's Allergies Department
2nd Floor, Stairwell B, South Wing
St Thomas' Hospital
Lambeth Palace Road
London SE1 7EH
Phone: 020 7188 8637
Fax: 020 7188 9782
E-mail: michael.perkin@kcl.ac.uk

FOOD STANDARDS AGENCY
Shuhana Begum
Food Standards Agency
Higher Scientific Officer
3B Aviation House
125 Kingsway
London WC2B 6NH
Phone: 020 7276 8516
Fax : 020 7276 8910
E-mail: shuhana.begum@foodstandards.gsi.gov.uk

MEDICAL RESEARCH COUNCIL
Dr Karen Finney PhD
Programme Manager: Nutrition and Metabolic Medicine
Medical Research Council
14th Floor, One Kemble Street
London WC2B 4AN
Tel: +44 (0) 20 7670 5305
E-mail: karen.finney@headoffice.mrc.ac.uk

Confidentiality Statement

This document is confidential and is to be distributed for review only to investigators, potential investigators, consultants, study staff, and applicable independent ethics committee. The contents of this document shall not be disclosed to others without written authorization from the study co-principal investigators, unless it is necessary to obtain informed consent from potential study participants.

Protocol Approval

Protocol T07051	Version:5.00
	Protocol Chair: Gideon Lack
Short Title: *Enquiring About Tolerance (EAT) Study*	

ICH/MRC Principles for Good Clinical Practice in Clinical Trials

We confirm that we will adhere to the principles of Good Clinical Practice (GCP) as produced by the International Conference on Harmonisation (ICH) Harmonised Tripartite Guideline for Good Clinical Practice produced in June 1996[1] and adopted by the MRC in March 1998.[2]

- Clinical Trials should be conducted in accordance with the ethical principles that have their origin in the Declaration of Helsinki, and that are consistent with GCP and the applicable regulatory requirement(s).
- Before a trial is initiated, foreseeable risks and inconveniences should be weighed against the anticipated benefit for the individual trial subject.
- The rights, safety, and well-being of the trial subjects are the most important considerations and should prevail over interests of science and society.
- The available non-clinical and clinical information on an investigational product should be adequate to support the trial.
- Clinical trials should be scientifically sound, and described in a clear, detailed protocol.
- A trial should be conducted in compliance with the protocol that has received a favourable independent ethics committee opinion.
- The medical care given to, and medical decisions made on behalf of subjects should always be the responsibility of a qualified physician.
- Each individual involved in conducting a trial should be qualified by education, training, and experience to perform his or her respective task.
- Freely given informed consent should be obtained from every subject prior to clinical trial participation.
- All clinical trial information should be recorded, handled, and stored in a way that allows its accurate reporting, interpretation and verification.
- The confidentiality of records that could identify subjects should be protected, respecting the privacy and confidentiality rules.
- Investigational products should be manufactured, handled, and stored in accordance with applicable good manufacturing practice (GMP).
- Systems with procedures that assure the quality of every aspect of the trial should be implemented.

FSA Joint Code of Practice for Research

We confirm that we are aware of the requirements of the FSA Joint Code of Practice and, in the proposed project, we will use our best efforts to ensure that the procedures used conform to those requirements under the following headings:

- Responsibilities
- Competence
- Project planning
- Quality Control
- Health and safety
- Handling of samples and materials
- Facilities and equipment
- Documentation of procedures and methods
- Research/work records

We understand that the funding body has the right to inspect our procedures and practices against the requirements of the Code of Practice, and that we may be asked to provide documentary evidence of our working practices or provide access and assistance to auditors appointed by the Funding Body.

____
Principal Investigator (Print)

________________________________ ________________
Principal Investigator (Signature) Date

Synopsis

**Title**	Randomized controlled trial of early introduction of allergenic foods to induce tolerance in infants
**Short Title**	Enquiring About Tolerance (EAT) Study
**Sponsors**	Guy's and St Thomas' Foundation NHS Trust & King's College London
**Primary Funders**	Food Standards Agency and the Medical Research Council
**Additional Funders**	National Institute of Health Research
**Conducted by**	Children's Allergies Department, Kings College
**Protocol Chair**	Professor Gideon Lack
**Ethical Approval**	St Thomas' Hospital Research Ethics Committee – 20/10/08 (08/H0802/93)
**Study Registration**	National Institute for Health Research (NIHR) Clinical Research Portfolio: Registration ID 6317<br>International Standard Randomized Controlled Trial Number Register (ISRCTN): 14254740
**Clinical Trials Facility**	Paediatric Allergy Clinical Trials Unit, Evelina Children's Hospital, St. Thomas' Hospital, London, UK
**Recruitment Centres**	***Antenatal centres***<br>Kingston Hospital<br>St Thomas' Hospital<br>***Postnatal***<br>Direct mailing to members of the Bounty Parenting Club
**Enrolment Objective**	2500 three month old infants (and their parents)<br>**Revised objective (contract variation September 2010):**<br>1302 three month old infants (and their parents)
**Study Design**	This is a randomized controlled trial of the early introduction of allergenic foods (and other foods) from three months of age in the general population. Mothers will be recruited using two methods: antenatally from two large antenatal units (Kingston and St Thomas' Hospitals); and postnatally using invitations mailed to mothers who are members of the Bounty Parenting Club. All participants will be encouraged to breastfeed exclusively until at least three months of age when half of the infants (early introduction group) will be randomized to continue breastfeeding and also introduce a number of allergenic foods into the diet from three months of age under dietetic direction. Baby rice or a pureed fruit/vegetable will be introduced at three months of age, followed by cow's milk yoghurt. Subsequently egg, fish (cod), peanut and sesame will be introduced in random order, with wheat introduced

	last (and not before four months of age). Children will be asked to consume these foods in recommended quantities until being assessed at one year of age, at which point consumption will be encouraged until completing the study at three years of age. Infants in the early introduction group will be encouraged to consume other foods (allergenic and non-allergenic).<br>The other half (standard introduction group) will be advised to continue exclusively breastfeeding until around six months of age as per the UK Government infant feeding advice.<br>All infants will be followed up until three years of age by which point the impact of the intervention on the primary outcome (food allergy) and secondary outcomes - asthma (including atopic wheeze), eczema, allergic rhinitis (including aero-allergen sensitization), combined food allergy prevalence (including food sensitization) and the prevalence of combined allergic disease will be known.
**Study Duration**	88 months.
**Study Start Date**	15th January 2008
**Study Recruitment Start**	2nd February 2009 (Antenatal)<br>1st October 2009 (Bounty mail outs)
**Final Report Due**	31st March 2015
**Project End Date**	31st May 2015
**Primary Endpoint**	The period prevalence of IgE mediated food allergy to the six intervention foods between one and three years of age in both arms.
**Secondary Endpoints**	***Period (one to three years of age) prevalence food outcomes***<br>The period prevalence of all IgE mediated food allergy between one and three years of age in both arms.<br><br>The period prevalence of all food allergy (IgE and non-IgE mediated) between one and three years of age in both arms.<br><br>The period prevalence of sensitization (defined in Section 3.9) to food between one and three years of age in both arms.<br><br>The total number of foods (of the six intervention foods) to which IgE mediated food allergy has been diagnosed between one and three years of age in both arms amongst children with IgE mediated allergy to one or more of the six intervention foods.<br><br>The total number of foods (any foods) to which IgE mediated food allergy has been diagnosed between one and three years of age in both arms amongst children with IgE mediated allergy to one or more foods.

Cumulative (by three years of age) prevalence food outcomes

The cumulative prevalence of IgE mediated food allergy to the six intervention foods by three years of age.

The cumulative prevalence of all IgE mediated food allergy by three years of age.

The cumulative prevalence of all food allergy (IgE and non-IgE mediated) by three years of age.

The cumulative prevalence of non-IgE mediated food allergy by three years of age.

The cumulative prevalence of sensitization (defined in Section 3.9) to the six foods by three years of age.

The total number of foods (of the six intervention foods) to which IgE mediated food allergy has been diagnosed by three years of age amongst children with IgE mediated allergy to one or more of the six intervention foods.

The total number of foods (any foods) to which IgE mediated food allergy has been diagnosed by three years of age amongst children with IgE mediated allergy to one or more foods.

Other allergic disease outcomes

The point prevalence of eczema at one year and three years of age and cumulative prevalence of eczema by three years of age.

The severity of eczema at one year and three years of age.

The prevalence of allergic rhinitis at three years of age.

The prevalence of inhalant allergen sensitization at one year and at three years of age by skin prick test.

The prevalence of inhalant allergen sensitization at one year and at three years of age by specific IgE measurement.

The prevalence of the atopic wheeze phenotype at three years of age.

Composite allergy outcome

The prevalence of combined allergic disease (a composite of cumulative IgE mediated food allergy to all foods, atopic wheeze phenotype, eczema and allergic rhinitis) at three years of age.

The prevalence of combined allergic disease (a composite of cumulative IgE and non-IgE mediated food allergy to all foods, atopic wheeze phenotype, eczema and allergic rhinitis) at three years of age.

Safety outcome

Incidence of adverse events and laboratory abnormalities; nutritional evaluations.

Immunological outcomes

Results of cellular and humoral assessments of immune response related to the development of allergy or tolerance to specific allergens (*subject to additional funding*)

Microbiome outcomes

The association between the bowel and skin microbiomes and the development of food allergy and other atopic disease.

Genetic analyses

The association between skin barrier gene defects (such as carriage of the filaggrin skin barrier mutations) and other measures of skin barrier integrity (transepidermal water loss) with all the study outcomes will be assessed.

Inclusion Criteria

Either

1. Pregnant mothers attending their 12/20 week ultrasound scans.

Or

2. Mothers planning on exclusively breastfeeding for at least the first three months.
3. Informed consent obtained from parent or guardian.

Exclusion Criteria

1. Significant antenatal anomaly at 20 week ultrasound scan.
2. Multiple pregnancy.
3. Significant congenital disease (enteropathy, congenital heart disease, renal disease).
4. Significant postnatal health concerns.
5. Any history of stridor irrespective of cause.
6. Premature delivery (less than 37 completed weeks gestation).

7. Parents not planning on breastfeeding exclusively for at least the first three months.
8. Parents planning on moving abroad before their child is three years of age.
9. Both parents unable to speak and read English.
10. Unwillingness or inability to comply with study requirements and procedures.
11. Family intend infant to be on a restricted diet (any of the six intervention foods)
12. ALT or bilirubin >2 times the upper limit of age-related normal value.

    ALT >144 (male) >126 (female),[3] Bilirubin >42
13. Urea or creatinine >1.25 times the upper limit of age-related normal value.

    Urea >10.3, Creatinine >60
14. Platelet count <100,000/ml, haemoglobin <9 g/dl, or investigator-suspected immunocompromise.
15. Significant vitamin D deficiency (alkaline phosphatase >1000).
16. Infant participating in another study prior to enrolment.

Study Intervention

The early introduction group consists of the dietetic controlled introduction of allergenic foods from three months of age. A pureed fruit/vegetable or baby rice mixed with breast milk or water will be commenced first, followed by cow's milk based yoghurt. Subsequently egg, sesame, fish, and peanut will be introduced in a randomized order, with wheat introduced last. These six allergenic foods will be sequentially introduced into the diet with each food being ingested twice a week achieving a total ingestion of four grams or more per week of each food protein by five months of age. Wheat will not be introduced before four months of age.

Infants in the early introduction group will be required to consume the allergenic foods until the one year assessment at which point ongoing consumption of all six allergenic foods will be strongly encouraged until the end of the study when at three years of age, subsequent consumption of the allergenic foods will be left to parental choice.

The standard introduction group will follow standard UK Government infant feeding advice (exclusive breastfeeding until around 26 weeks of age) and avoid introducing foods that may cause allergies before six months of age (cow's milk, egg, wheat, peanuts, tree nuts, seeds, fish and shell fish).

Glossary of Abbreviations

**ALSPAC**	Avon Study of Parents And Children
**BMI**	Body Mass Index
**CWT**	Caroline Walker Trust
**COMA**	Committee on Medical Aspects of Food Policy
**COT**	Committee On Toxicity of chemicals in food, consumer products and the
**CRF**	Case Report Form
**CTCAE**	Common Terminology Criteria for Adverse Events
**DBPCFC**	Double-Blind, Placebo-Controlled Food Challenge
**DH**	Department of Health
**DSMB**	Data Safety Monitoring Board
**EIG**	Early Introduction Group
**FA**	Food Allergy
**FAP**	Facilitated Antigen Presentation
**FFQ**	Food Frequency Questionnaire
**FSA**	Food Standards Agency
**GCP**	Good Clinical Practice
**IDMC**	Independent Data Monitoring Committee
**ISAAC**	International Study of Asthma and Allergies in Childhood
**ISRCTN**	International Standard Randomized Controlled Trial Number Register
**KLH**	Keyhole Limpet Hemocyanin
**LEAP**	Learning Early About Peanut allergy
**MRC**	Medical Research Council
**NCI**	National Cancer Institute
**NDNS**	National Diet and Nutrition Survey
**NIHR CRN**	National
**OCC**	Open Cumulative Challenge
**OIC**	Open Incremental Challenge
**P(T)MG**	Project (Trial) Management Group
**POEM**	Patient-Oriented Eczema Measure
**SAE**	Serious Adverse Event
**SIG**	Standard Introduction Group
**SPT**	Skin-Prick Test
**SCORAD**	SCORing Atopic Dermatitis
**TEWL**	Trans-Epidermal Water Loss
**TSC**	Trial Steering Committee
**UCV**	Unscheduled Clinic Visit
**UK**	United Kingdom
**UKCRN**	United Kingdom clinical research network
**WHO**	World Health Organization

Contents

1. BACKGROUND 7
1.1 SUMMARY AND RATIONALE FOR TRIAL 7
1.2 AETIOLOGY OF FOOD ALLERGY 7
1.2.1 Increased Prevalence of Food Allergy 7
1.2.2 The State-of-the-Art in the Research Area 8
1.2.3 The Scientific Basis for the Work Proposed 9
1.2.4 How this Project will Advance the State-of-the-Art 11
1.3 RATIONALE FOR TRIAL DESIGN 11
1.3.1 General Population 11
1.3.2 Interventional Study 11
1.3.3 Pragmatic Design 11
1.3.4 Justification for Enrolment at three Months of Age 13
1.3.5 Justification for Skin Prick Testing Intervention Children Only at Three Month Enrolment Visit 16
1.3.6 Justification for Administering Cow's Milk Protein as a Solid (Yoghurt) 16
1.3.7 Justification for Reviewing All Infants at One Year of Age 18
1.3.8 Justification for the Three Year Assessment as the Principal Outcome 18
1.3.9 Justification for the Challenging All Skin Prick Positive Children at One and Three Year Assessments 19
1.3.10 Amendment to EAT food challenge protocols (June 2011) 21
1.4 RATIONALE FOR IMMUNOLOGICAL ASSESSMENTS 29
1.5 RATIONALE FOR COLLECTION OF STOOL SAMPLES (AUGUST 2011) 30
1.5.1 The Scientific Basis for the Work Proposed 30
1.5.2 Study hypotheses and outcomes: 32
1.5.3 Sample Selection 32
1.5.4 Stool collection 33
1.5.5 Sample processing 34
1.6 KNOWN AND POTENTIAL RISKS 34
1.6.1 Early Introduction Group 34
1.6.2 Standard Introduction Group 35
1.6.3 Both Groups 35
2. SCIENTIFIC OBJECTIVES 36
2.1 OBJECTIVE ONE 36
2.2 OBJECTIVE TWO 36
2.3 OBJECTIVE THREE 37
3. STUDY DESIGN 38
3.1 OVERALL STUDY DESIGN 38
3.1.1 EAT Study Population 39
3.1.2 Maternal participation 41
3.2 INFANT ENROLMENT - SCREENING FOR ALLERGY AT THREE MONTHS 42

3.3 PRIMARY ENDPOINT....43
3.3.1 Definition of IgE mediated food allergy for primary endpoint....43
3.4 SECONDARY ENDPOINTS....43
3.5 DETERMINATION OF FOOD ALLERGY – UNSCHEDULED CLINIC VISITS....45
3.6 DETERMINATION OF FOOD ALLERGY – ONE YEAR ASSESSMENT....48
3.7 DETERMINATION OF FOOD ALLERGY – THREE YEAR ASSESSMENT....49
3.8 DETERMINATION OF SENSITIZATION....49
3.9 STUDY DEFINITIONS....51
3.9.1 Allergic Disease Definitions....51
3.9.2 Infant Feeding Definitions....51
3.10 STOPPING RULES....52
3.10.1 Ongoing Review....52
3.10.2 Review of Specific Adverse Events....52
3.11 STUDY DURATION....53
4. ELIGIBILITY....54
4.1 INCLUSION CRITERIA....54
4.2 EXCLUSION CRITERIA....54
4.3 PREMATURE TERMINATION....54
4.3.1 Premature Termination of Trial Interventions....54
4.3.2 Premature Termination from the Trial....54
5. STUDY INTERVENTION....55
5.1 ALLERGENIC DIET ADMINISTRATION OR AVOIDANCE....55
5.2 ASSESSMENT OF COMPLIANCE WITH STUDY INTERVENTION....56
5.3 MODIFICATION OR DISCONTINUATION OF STUDY TREATMENT....56
6. STUDY PROCEDURES....57
6.1 VISIT WINDOWS....57
6.2 GENERAL ASSESSMENTS....57
6.3 QUESTIONNAIRE SCHEDULE....58
6.4 LABORATORY ASSESSMENTS....58
6.5 ALLERGY ASSESSMENTS....59
6.5.1 Allergens Assessed....59
6.5.2 Skin Prick Testing: Procedures and Interpretation....59
6.6 FOOD CHALLENGES....60
6.6.1 Scheduled Challenges....60
6.6.2 Unscheduled Challenges....60
6.6.3 Repeat Challenges....60
6.6.4 Minimum Intervals between Challenges....60
6.6.5 Challenges for Non-Intervention Foods....63
6.6.6 Challenge Sequence for Multiply Sensitized Participants....63
6.6.7 Procedure for Challenges for Suspected IgE Mediated Food Allergy....63
6.6.8 Procedure for Challenges for Suspected Non-IgE Mediated Food Allergy....67
6.6.9 Procedure for Suspected Coeliac Disease....68

6.6.10 Procedure for Suspected Food Protein Induced Enterocolitis Syndrome (FPIES) Reactions 68
7. TOLERANCE ASSAYS 70
7.1 FROZEN PBMC T-CELL ASSAY 70
7.1.1 Overview....70
7.1.2 Gene Expression Profiling 70
7.1.3 Secreted Cytokines....70
7.1.4 Antigen-specific Proliferative and Cytokine Responses 70
7.1.5 Regulatory T cells 70
7.2 PLASMA ALLERGEN-SPECIFIC IGG AND IGE 71
7.2.1 ELISA-based Techniques 71
7.2.2 Facilitated Antigen Presentation Inhibition....71
7.3 WHOLE BLOOD DNA–HLA GENOTYPES....71
8. ADVERSE EVENTS 72
8.1 OVERVIEW 72
8.2 INDEPENDENT DATA MONITORING COMMITTEE (IDMC)....72
8.2.1 Terms of reference 72
8.2.2 Membership 72
8.2.3 Guidance notes....72
8.3 DEFINITIONS 73
8.3.1 Adverse Event....73
8.3.2 Serious Adverse Event....73
8.3.3 Unexpected Adverse Event....74
8.4 COLLECTING ADVERSE EVENTS 74
8.4.1 Methods of Collection....74
8.4.2 Collecting Serious Adverse Events....74
8.4.3 Recording Adverse Events....74
8.4.4 Recording Serious Adverse Events....74
8.5 GRADING AND ATTRIBUTION OF ADVERSE EVENTS 74
8.5.1 Grading Criteria 74
8.5.2 Attribution Definitions....75
8.6 REPORTING ADVERSE/SERIOUS ADVERSE EVENTS....75
8.6.1 Reporting Timeline 75
8.6.2 Options for Reporting Serious Adverse Events 76
9. STATISTICS....77
9.1 ANALYSIS SAMPLES 77
9.2 ANALYSIS OF ENDPOINTS....77
9.2.1 Analysis of Primary Endpoint....77
9.2.2 Analysis of Secondary Endpoints 77
9.2.3 Additional Analyses....78
9.3 SAMPLE SIZE 78
9.3.1 Full cohort....78
9.3.2 High risk subgroup 78

9.4 LOSS TO FOLLOW-UP....78
9.5 RANDOMIZATION, STRATIFICATION, AND BLINDING....79
9.6 REPORTING DEVIATIONS FROM THE ORIGINAL STATISTICAL PLAN....79
10. IDENTIFICATION AND ACCESS TO SOURCE DATA....80
10.1 IDENTIFYING SOURCE DATA....80
10.2 PERMITTING ACCESS TO SOURCE DATA....80
11. QUALITY CONTROL AND QUALITY ASSURANCE....81
11.1 MANAGEMENT STRUCTURE....81
11.1.1 Trial Steering Committee....82
11.1.2 Project (Trial) Management Group....83
11.1.3 Independent Data Monitoring Committee....84
11.1.4 External Scientific Advisors....84
11.2 DATA HANDLING....84
12. ETHICAL CONSIDERATIONS AND COMPLIANCE WITH GOOD CLINICAL PRACTICE....86
12.1 STATEMENT OF COMPLIANCE....86
12.2 INFORMED CONSENT AND ASSENT....86
12.3 PRIVACY AND CONFIDENTIALITY....86
13. COMMUNICATION POLICY....87
13.1 PUBLICATION POLICY....87
13.2 COMMUNICATION WITH PARTICIPANTS....87
14. REFERENCES....88

APPENDIX 1. STUDY OVERVIEW CHART – *POST CONTRACT VARIATION SEPTEMBER 2010*....95

APPENDIX 2. STUDY GANTT CHART – *POST CONTRACT VARIATION SEPTEMBER 2010*....96

APPENDIX 3. SCHEDULE OF EVENTS....97

List of Tables

TABLE 1. FREQUENCY OF ALLERGENIC FOOD CONSUMPTION IN UK INFANTS AGED 8-10 MONTHS 12

TABLE 2. PREVALENCE (%) OF EXCLUSIVE BREASTFEEDING BY COUNTRY (2005) 14

TABLE 3. DURATION OF EXCLUSIVE BREASTFEEDING AMONG MOTHERS WHO FED EXCLUSIVELY AT BIRTH BY COUNTRY (2005) 14

TABLE 4. DISTRIBUTION OF SKIN PRICK RESULTS IN FAIR COHORT AT ONE AND THREE YEARS OF AGE ..19

TABLE 5. LIKELY DISTRIBUTION OF SKIN PRICK RESULTS AT ONE AND THREE YEARS OF AGE BASED ON EXTRAPOLATING FAIR DATA TO THE EAT STUDY 20

TABLE 6. LIKELY NUMBER OF FOOD CHALLENGES REQUIRED AT ONE AND THREE YEARS OF AGE BASED ON EXTRAPOLATING FAIR DATA TO THE EAT STUDY USING TWO THRESHOLDS: ALL POSITIVE RESULTS AND >2MM 21

TABLE 7. SUMMARY OF RESULTS OF AUDIT PERFORMED 09.03.11 ON ALL 1 YEAR VISIT DBPCFCS CONDUCTED 22

TABLE 8. FURTHER ANALYSIS OF CHALLENGES DEEMED INDETERMINATE 22

TABLE 9. ESTIMATED AGE APPROPRIATE PORTION SIZES FOR UK CHILDREN 1 YEAR AND 3 YEARS OF AGE..........24

TABLE 10. APPROXIMATE GRAMS OF PROTEIN IN ESTIMATED AGE APPROPRIATE PORTION SIZES FOR A 1 YEAR OLD 25

TABLE 11. APPROXIMATE GRAMS OF PROTEIN IN ESTIMATED AGE APPROPRIATE PORTION SIZES FOR A 3 YEAR OLD 25

TABLE 12. SUMMARY OF DIFFERENCES BETWEEN SELECTED STUDIES AND CLINICAL PRACTICE AREAS. .26

TABLE 13. SUMMARY OF PROPOSED AMENDMENTS TO EAT STUDY CHALLENGE PROTOCOLS AT ONE AND THREE YEAR VISITS. 27

TABLE 14. CRITERIA THAT DEFINE SELECTED INFANT FEEDING PRACTICES..........52

TABLE 15. QUESTIONNAIRE SCHEDULE 58

TABLE 16. CHALLENGE PROGRAMME – SCHEDULED CHALLENGE VISITS..........61

TABLE 17. CHALLENGE PROGRAMME – UNSCHEDULED CHALLENGE VISITS 62

TABLE 18. CHALLENGE PROGRAMME – FPIES 62

TABLE 18. CRITERIA FOR DETERMINING THE OUTCOME OF FOOD CHALLENGE..........66

TABLE 19. NCI-CTCAE ATTRIBUTION OF ADVERSE EVENTS 75

List of Figures

FIGURE 1. PROTOCOL DEFINED EGG ALLERGY AT ENROLMENT INTO LEAP STUDY. 15

FIGURE 2. SENSITISATION TO ALLERGENIC FOODS (IGE>0.35KU/L) AT ENROLMENT ONTO THE LEAP STUDY. 16

FIGURE 3. THE MEDIAN BREASTFEEDING FREQUENCY IN THE SOLIDS AND FORMULA GROUPS. 17

FIGURE 4. THE MEDIAN BREASTFEEDING FREQUENCY BY AGE AT INTRODUCTION OF SOLIDS IN THE SOLIDS GROUP AND OF FORMULA IN THE FORMULA GROUP. 17

FIGURE 5. OVERALL STUDY DESIGN 38

FIGURE 6. THREE MONTH SCREENING VISIT 42

FIGURE 7. ASSESSMENTS TO BE PERFORMED AT UNSCHEDULED CLINIC VISITS- IGE MEDIATED FOOD ALLERGY 46

FIGURE 8. ASSESSMENTS TO BE PERFORMED AT UNSCHEDULED CLINIC VISITS- NON-IGE MEDIATED FOOD ALLERGY 47

FIGURE 9. ONE YEAR ASSESSMENT VISIT – IGE MEDIATED FOOD ALLERGY 48

FIGURE 10. THREE YEAR ASSESSMENT VISITS – IGE MEDIATED FOOD ALLERGY 50

FIGURE 11. MANAGEMENT STRUCTURE 81

1. BACKGROUND

1.1 SUMMARY AND RATIONALE FOR TRIAL

In the United Kingdom (UK) 6% of children will develop food allergies (FA).[4] The one year prevalence of allergic disease amongst six to seven years old children in the UK is: 16.0% eczema, 20.9% asthma and 10.1% allergic rhinitis (AR).[5] The World Health Organization (WHO) Global Strategy for Infant and Young Child Feeding,[6] endorsed by the UK Goverment,[7] recommends exclusive breastfeeding (EBF) for the first six months. The UK Government infant feeding information leaflet for parents, "Weaning – starting solid food", adopts a more pragmatic target of around six months exclusive breastfeeding.[8] It also states that if a mother decides to introduce complementary foods before six months there are some foods that should be avoided as they may cause allergies including: "wheat-based foods and other foods containing gluten (e.g. bread, rusks, some breakfast cereals), eggs, fish, shellfish, nuts (and) seeds." There is little evidence that this reduces allergic disease. There are conflicting results of interventional trials of delayed introduction of complementary foods to reduce FA and atopy. Animal models and preliminary human data suggest that high dose oral tolerance to food proteins in early life may prevent allergic sensitization. Three separate studies show that prolonged EBF is a risk factor for developing atopic disease.[9-11] Since the 1970s allergic disease has increased significantly: asthma prevalence doubled and eczema prevalence tripled between 1973 and 1988 in a study in South Wales.[12] These increases have coincided with a two-thirds reduction in early introduction of complementary foods.[13] Thus it may be that delayed introduction of complementary foods has promoted FA and even other atopic diseases. We propose a randomized controlled study in infants to determine whether early introduction of complementary foods and exposure to food allergens (from three months of age) prevents the development of FA, eczema, asthma and AR. This study will provide an informed basis for future infant feeding practices in both atopic and normal infants.

1.2 AETIOLOGY OF FOOD ALLERGY

1.2.1 INCREASED PREVALENCE OF FOOD ALLERGY

Food allergy develops in approximately 6% of children in the UK[4] and the prevalence for particular allergens (peanut) is increasing.[14] The reason for this increase remains unclear. Interventions to prevent food allergy have involved intervening with one or more of the following: antenatal environment (maternal diet); maternal lactation (duration of exclusive breastfeeding and maternal diet whilst lactating); infant diet (timing of introduction of mixed feeding and number and types of foods introduced).

With regard to the introduction of mixed feeding there has been a significant trend since 1975 towards later introduction. The proportion of infants given solids by eight weeks of age has decreased: 49% in 1975, 24% in 1980 & 1985, and 19% in 1990.[13] It is a simple ecological observation that this decrease to a third of what it was has coincided with up to a three-fold increase in allergy in children.[12] This change has been compounded by a number of different bodies recommending delayed introduction of foods. The American Academy of Pediatrics recommended avoiding solids until six months of age, cow's milk until one year of age, egg to two years and peanuts, tree nuts and fish to three years of age.[15] These guidelines have only recently been rescinded.[16] The European Society for Paediatric Gastroenterology, Hepatology and Nutrition (ESPGHAN) Committee on Nutrition has also recently revised its recommendations.[17] It states that "complementary feeding (i.e., solid foods and liquids other than breast milk or infant formula and follow-on formula) should not be introduced before 17 weeks and not later than 26 weeks." In the UK the Government has published a paper entitled "Infant Feeding Recommendation" which summarises the latest advice with regard to the introduction of solid food.[7] The paper states that the UK Government recommendation on feeding infants is that "exclusive breastfeeding is recommended for the first six months (26 weeks) of an infant's life."

The paper also states that: "Certain foods are more likely to upset a baby or cause an allergic reaction than other foods. These foods should not be introduced before six months (COMA 1994)." However, whilst the section on the management of food allergy in the Committee on Medical Aspects of Food Policy (COMA) report contains a specific sentence on not introducing allergenic foods before six months of age, it is not referenced.[13] The WHO's systematic review of the optimal duration of exclusive breastfeeding found no significant reduction in risk of atopic eczema, asthma, or other atopic outcomes amongst those exclusively breastfed for six months compared with those exclusively breastfed for three to four months followed by mixed feeding.[18] In theory, deferring introduction of an allergenic food prevents a reaction. However, this doesn't allow for accidental exposures, the possibility of cross reactivity with other foods and possible sensitization by other means (cutaneous or via continued breast milk exposure). It may simply defer presentation of an allergy.

1.2.2 THE STATE-OF-THE-ART IN THE RESEARCH AREA

Interventions to reduce food allergy

The outcome of studies using the interventions mentioned above has been generally disappointing. Removing egg and cow's milk in the third trimester of pregnancy made no difference in two studies.[19;20] Whilst one out of two studies showed a short term reduction in eczema with avoidance of egg, cow's milk and fish during lactation, there was no difference in any other outcomes.[21;22] Of the two studies adopting a multi-intervention approach (pregnancy and lactation exclusion of allergenic foods, soya/casein hydrolysate for six months in non breastfed infants and delayed introduction of cow's milk and solids for 6-11 months) only one achieved a reduced prevalence of food allergy and cow's milk sensitisation.[23;24] Data on interventions specifically focusing on introduction of solids is limited. One randomized study showed no difference in fish and citrus allergy by three years of age between children with fish introduced early or late (after one year of age).[25] A prospective non-randomized study found a reduction in food allergy at one year of age in the group fed solids after six months compared to before four months, but there was no difference in eczema or skin prick sensitization to a panel of food allergens at five years of age.[26]

Solids introduction and eczema

Fergusson reported a 2.9 times greater risk of chronic/recurrent eczema amongst children fed four or more solids before four months of age compared with those not fed solids before four months of age. The difference was still apparent at ten years of age. However, as with virtually all the studies published to date the study, whilst being prospective, was observational only and did not involve a randomized intervention.[27] Whilst this study is often quoted as evidence that early introduction of solids induces allergic disease, a more recent Australian study found no significant association between the duration of breastfeeding or timing of introduction of solid foods and protection against asthma or other allergic disease in 516 children evaluated at age five years. However, breastfeeding for six months or more and introduction of solid foods after three months were both associated with an increased risk of atopy at age five years.[28] This is consistent with another recent study. Sariachvili *et al* undertook a nested case-control study in the PIPO cohort (Prospective Cohort on the Influence of Perinatal Factors on the Occurrence of Asthma and Allergies). Early introduction (within the first four months) of solid foods was inversely associated with eczema up to four years of age (OR_{adj} 0.5, 95% CI 0.3-0.7). Moreover, early exposure to solid foods was associated with a reduced risk for eczema only among children with allergic parents (OR_{adj} 0.4, 95% CI 0.2-0.6), whereas no significant effect was found among children with non-allergic parents (OR_{adj} 0.7, 95% CI 0.4-1.3).[29]

Possible risks of delaying solid introduction

Three observational studies have found that delayed introduction of solids was related to an increased risk of asthma and eczema.[9-11] Reverse causality has been proposed as an explanation. However Zutavern found no evidence of feeding practices playing a different role in the development of asthma and eczema with respect to: mother's opinion of child's eczema status at year one; and parental allergy, asthma, and atopy status.[11] Similarly, a recent study found that the delayed introduction of cereal grains (after the age of six month) was associated with an

increased risk of wheat allergy.[30] Related to this, an EU funded Framework 6 Project has been commenced which aims to determine whether the early introduction of wheat into the diet prevents the development of coeliac disease and induces tolerance (http://www.preventceliacdisease.com). In this randomized controlled trial the intervention arm will receive daily small amounts of gluten during the period of breastfeeding from the age of four months (apparently each dose being 3% of what a normal six month old would consume). The control arm receives a milk sugar powder placebo during the period of breastfeeding from the age of four months.

Data has also emerged with regard to fish consumption with a study finding that introducing fish before nine months of age was associated with a reduction in allergic disease, specifically eczema.[31]

Similarly, a recent Dutch study found that more delay in introduction of cow milk products was associated with a higher risk for eczema. In addition, a delayed introduction of other food products was associated with an increased risk for atopy development at the age of two years. Exclusion of infants with early symptoms of eczema and recurrent wheeze (to avoid reverse causation) did not essentially change the results. The authors concluded that delaying the introduction of cow milk or other food products may not be favourable in preventing the development of atopy.[32]

1.2.3 THE SCIENTIFIC BASIS FOR THE WORK PROPOSED

Oral tolerance

There is data from a variety of sources to suggest that delaying the introduction of allergenic foods might not be the right approach. Clinical observations from countries in Southeast Asia and Africa, where high amounts of peanuts are consumed in different snack forms during infancy, suggest a low rate of peanut allergy. As these differences could be due to genetics, we have examined these geographical variations more carefully by comparing the prevalence of peanut allergy in Jewish children in the UK and Israel.[33] The prevalence of peanut allergy in the UK was 1.85%, and the prevalence in Israel was 0.17% ($P < .001$). Despite accounting for atopy, the adjusted risk ratio for peanut allergy between countries was 9.8 (95% CI, 3.1-30.5) in primary school children. Peanut is introduced earlier and is eaten more frequently and in larger quantities in Israel than in the UK. The median monthly consumption of peanut in Israeli infants aged 8 to 14 months is 7.1 g of peanut protein, and it is 0 g in the UK ($P < .001$). The median number of times peanut is eaten per month was eight in Israel and zero in the UK ($P < .0001$).

One study of oral tolerance induction in adults showed that oral intake of keyhole limpet hemocyanin (KLH) results in immunological tolerance to KLH antigen.[34] Two studies have attempted to induce tolerance to food allergens.[35;36] The first was conducted in patients who already had established milk allergy. The result of this study was promising: 71% of highly allergic children were able to tolerate a daily intake of 200 mls of milk after treatment.[35] However, because this was an uncontrolled study, the possibility that these children would have shown spontaneous resolutions cannot be discounted. More recently four children with peanut allergy underwent desensitisation and were subsequently able to tolerate between 10 and 12 peanuts.[36]

There is some evidence that oral exposure to nickel results in tolerance. Numerous studies, both prospective and retrospective, show that early cutaneous exposure to jewellery, particularly through ear piercing, is a risk factor for the development of contact dermatitis to nickel. Three independent studies,[37-39] including one prospective birth cohort study, show that the early application of orthodontic braces made of nickel strongly protects against the development of contact dermatitis to nickel (in one study there was an odds ratio of 0.07). Indeed, after the insertion of fixed orthodontic appliances, the level of nickel both in the saliva and serum of individuals increases significantly, which is thought to result in oral tolerance. Similarly, patients exposed to pancreatic extract by inhalation or contact develop IgE-mediated allergic reactions, whereas patients exposed to this extract by oral route do not.[40]

Animal data

Animal data is also supportive of the hypothesis. In most experimental animals a state of specific systemic hypo-responsiveness is induced by single or multiple intestinal antigen exposure.[41] A PubMed search for publications containing all the text words: immune, tolerance, single, dose, oral and administration, yielded 60 documents (search undertaken 25.02.09). These documents include a review article describing the principle of tolerance induction and its relationship with dosage and frequency of timing in animal models and this confirms that a single oral dose of antigen is sufficient to induce tolerance.[42] This phenomenon has been demonstrated for different antigens in different experimental models, but the resulting data are consistent: they uniformly show that a single dose of oral protein administration effectively causes immunological tolerance and prevents the expression of related clinical disease. With regard to timing, the animal data suggests that continuous feeding beyond the critical neonatal period leads to induction of tolerance.[41]

Oral tolerance induction in animal models is most potent in its effects on delayed type I hypersensitivity responses; prevention of antibody responses through induction of oral tolerance is less consistent. However, numerous publications point to the fact that in mice a single dose of food allergen (beta-lactoglobulin, ovalbumin, peanut) is particularly effective in preventing the development of subsequent IgE-mediated responses. A recent study showed that naïve mice orally tolerized to beta-lactoglobulin were unable to mount significant IgE responses when they were subsequently sensitized with beta-lactoglobulin injected along with alum intraperitoneally.[43] Similarly, there were no significant T-cell responses to beta-lactoglobulin in the pretolerized animals.

Strid and colleagues fed mice a single intragastric feed of defatted peanut flour at doses varying from 0.2 to 100 mg per mouse.[44] Seven days after the feed, the mice were immunized with 100 µg of peanut antigen emulsified with Complete Freunds Adjuvant. Three weeks later, the mice were given a recall immunization with 100 µg of antigen. The mice were assayed for T-cell proliferation to peanut, cytokine production, delayed-type hypersensitivity responses, and antibody responses. Tolerizing doses of 100 mg of peanut protein resulted in significant reduction of delayed-type hypersensitivity responses and inhibition of proliferative responses to peanut. Mice tolerized to 100 mg of peanut protein showed significantly reduced interferon gamma and IL4 production. Specific IgE responses to peanut following sensitization were almost completely prevented by the single tolerizing dose. However, very low tolerizing doses of peanut, i.e., those below 2 mg per animal, resulted in enhanced delayed-type hypersensitivity responses, T-cell proliferative responses, cytokine production, and IgE production. Doses between 2 and 20 mg of peanut protein induced no difference between the T- and B-cell responses as compared to sham-tolerized animals. Tolerance to peanut was only achieved at doses of 100 mg per animal. Oral tolerance to peanut was shown to be antigen specific. Tolerizing doses of peanut did not promote tolerance to ovalbumin and vice versa.

Exclusive breastfeeding and other disease outcomes

The WHO systematic review of the optimal duration of exclusive breastfeeding stated that: "Based primarily on an observational analysis of a large randomized trial in Belarus, infants who continue exclusive breastfeeding for six months or more appear to have a significantly reduced risk of one or more episodes of gastrointestinal infection".[18] However, the Millennium cohort, a UK based longitudinal study of 15,980 infants has recently reported that the age of introduction of solids had no effect on risk of hospitalization for diarrhoea or lower respiratory tract infection.[45]

Summary

Our study group is currently undertaking a randomized controlled trial of the early introduction of peanuts into the diet of high risk infants to induce tolerance: Learning Early About Peanut (LEAP) Study. The EAT Study is a randomized controlled trial of the early introduction of allergenic foods in a normal population. Recruitment for this study commences after the completion of recruitment of the peanut intervention study. Participants will be encouraged to breastfeed exclusively until three months of age when half of the infants will be randomized to introduce a number of allergenic foods into the diet under dietetic direction. Participants will be

followed up until three years of age by which point the impact of the intervention on the primary outcome (food allergy) and secondary outcomes (eczema, asthma, allergic rhinitis, allergen sensitization) will be known. The scale of this study will provide a unique opportunity as a platform on which to seek additional funding for a diversity of further research. This will include further nutritional and metabolic work. The proposal is also unique in that there have been no randomized interventional studies looking at the effects of early introduction of allergenic foods in general. This study will allow the safety and possible beneficial effects on growth and normal development to be assessed. The research project will be physically located in a purpose built six bedded paediatric allergy clinical trials unit housed in the Evelina Children's Hospital at Guy's and St Thomas' NHS Foundation Trust. This unit forms part of the MRC & Asthma UK Centre in Allergic Mechanisms of Asthma headed by Professor Tak Lee. This is the first disease specific centre of its kind in the UK to address allergic mechanisms. The centre benefits from a vast collaborative network of principal investigators experienced in both basic laboratory and clinical research.

1.2.4 HOW THIS PROJECT WILL ADVANCE THE STATE-OF-THE-ART

A positive outcome with the successful induction of tolerance may have implications for infant feeding policy both nationally and internationally. In addition, a reduction in the number of children with allergies will, in turn, have a significant cost benefit for the administration of healthcare.

1.3 RATIONALE FOR TRIAL DESIGN

1.3.1 GENERAL POPULATION

As with any interventional study enrolling a "general" population, we will be subject to a participation bias. In every such study, individuals with a vested interest in the study (a family or personal history of the condition in question) are more likely to participate. This is absolutely unavoidable. We will, however, ensure that all mothers are approached to participate in the study and our information leaflets will stress the importance of all mothers participating. We will ask mothers who decline to participate if they have a personal or family history of allergic disease so we can quantify the degree of participation bias compared with those who enrol.
Conversely, if our population does transpire to be at a higher risk of food allergy than the general population this will have the effect of increasing the power of the study.

1.3.2 INTERVENTIONAL STUDY

There is considerable confusion in the field of infant feeding and its effect on allergic disease and child health: Remarkably, there has not been a single large randomized controlled interventional study to address this issue. This is the first such study. We will determine the health and nutritional consequences of introducing foods early and whether this measure is effective in reducing allergic diseases. This is consistent with the WHO recommendation that: "Large randomized trials are recommended to rule out small effects on growth and to confirm the reported health benefits of exclusive breastfeeding for six months or beyond".[18] With conflicting data and health policies in different parts of the world, there is an insufficient evidence base with which to advise parents with regard to allergy prevention and infant feeding.

1.3.3 PRAGMATIC DESIGN

This is a pragmatic study consisting of an active intervention versus the status quo which is an incomplete following of the current UK Government infant feeding recommendations.

Current UK Government infant feeding recommendations:
UK Government infant feeding guidelines currently state: "If you decide to wean at any time before six months, there are some foods that should be avoided as they may cause allergies... These include wheat-based foods and other foods containing gluten (e.g. bread, rusks, some breakfast cereals), eggs, fish, shellfish, nuts *and* seeds."[8] The guidance also states with regard to high risk families: "Babies are more likely to develop allergies if there is a family history of

eczema, asthma or hay fever. For these families, exclusive breastfeeding is particularly recommended for the first six months. Introduce the foods that commonly cause allergies (milk, eggs, wheat, nuts, seeds, fish and shellfish) one at a time so that you can spot any reaction, but don't introduce any of these foods before six months."[8] Until the 25th August 2009 these infants were also advised to follow the Committee On Toxicity of chemicals in food, consumer products and the environment (COT) recommendations on avoiding peanuts: "Avoid giving peanuts and foods containing peanut products, e.g. peanut butter or groundnut oil, until your child is three years old."[8] However, the Government revised these recommendations on this date following on from a review of the evidence by the COT and a recommendation to the Health Minister by the FSA board. The revised guidance states: "If your child already has a known allergy, such as a diagnosed food allergy or diagnosed eczema, or if there is a history of allergy in your child's immediate family (if the child's parents, brothers or sisters have an allergy such as asthma, eczema, hay fever, or other types of allergy), then your child has a higher risk of developing peanut allergy. In these cases you should talk to your GP, health visitor or medical allergy specialist before you give peanuts or foods containing peanuts to your child for the first time."

Actual allergenic food consumption:
Although the UK Government guidelines do not stipulate delaying the introduction of allergenic foods beyond six months of age (with the exception of the previous COT recommendations for peanut in high risk infants), we know that the current feeding regimen consists of little allergenic food consumption in the first year of life (with the exception of cow's milk products and wheat). Data from the Infant Feeding Survey 2005 show the frequency with which mothers were giving their infants allergenic foods at Stage three of the survey (when the infants were between eight and ten months of age) (Table 1).[46]

Table 1. Frequency of allergenic food consumption in UK infants aged 8-10 months

Food	Percentage giving food:			
	1/day or more	1-6 times a week	<1/week or never	3/week or more
Cheese, yoghurt, fromage frais	64	26	9	85
Breakfast cereals	82	8	9	88
Bread	36	38	25	58
Eggs	2	23	76	6
Fish (incl. Tuna)	3	45	52	18
Nuts (incl. Ground nuts)	<1	1	99	<1

In the Infant Feeding Survey 2005, among mothers who cited at least one food avoidance by Stage three of the Survey, the most common reason was a concern about allergies (43%, a rise of eight percentage points since the 2000 survey). Avoidance of specific foods as ingredients was common. For egg, 24% avoided it because they considered it harmful and 44% because of concerns about allergies. For dairy products, 17% were concerned about harm, 50% allergies and 8% concerned with eczema. For nuts 33% were concerned about harm and 70% allergies (mothers could report more than one concern). Thus our study will entail a comparison between early high dose introduction versus little, if any, early allergen exposure (with the exception of cow's milk and wheat).

Compliance with UK Government infant feeding guidelines amongst the standard introduction group
To participate in the study all mothers will have had to exclusively breastfeed for at least three months prior to randomization. The current WHO definition of exclusive breastfeeding is given in Section 3.9.2. However, it will be deemed acceptable for mothers to have given boiled then cooled water, as well as oral rehydration solution, without being considered to have ceased exclusive breastfeeding as this will not have any immunomodulatory effect on the infant.
A pragmatic approach has been taken to comply with the UK Government infant feeding

guidelines in the standard introduction arm with regard to the per protocol analysis (Section 9.1). This is because we anticipate that despite support to continue exclusive breastfeeding, some mothers in the standard introduction arm will choose to introduce cow's milk based formula before six months of age. In terms of the design of the study, we believe there is no value in trying to achieve a modification of behaviour to such a degree of compliance that it could not be expected to be achieved as a public health measure. In other words there is no point trying to ensure a regimen of complete avoidance if this cannot be realistically carried out in real life outside of the setting of a study intervention.

Objective monitoring of actual food consumption

High food specific IgG levels are known to correlate with increasing consumption of the specific food (Vance et al., 2004; Vance et al., 2005). Therefore a further way of objectively validating the dietary allergenic food intake will involve specific IgG measurement to food allergens. We will be able to measure IgG levels on stored serum samples (subject to further funding). This allows us to objectively validate the dietary allergenic food intake of the children in both arms of the study and thus will be done on the samples obtained from the whole cohort at the one and three year assessments.

1.3.4 JUSTIFICATION FOR ENROLMENT AT THREE MONTHS OF AGE

There are several reasons why it has been decided to enrol infants onto the study at three months of age rather than later.

Exclusive breastfeeding rates

The Infant Feeding Survey 2005 gave the following historical background to the government's policy on breastfeeding:

"Due to *the* body of evidence on the health benefits of breastfeeding, government policy in the United Kingdom has consistently supported breastfeeding as the best way of ensuring a healthy start for infants. In 1974 a COMA Working Party was set up to review infant feeding practices in the United Kingdom. The recommendations of this Working Party were that all mothers should be encouraged to breastfeed and that mothers should be discouraged from introducing solid foods before four months old. Subsequent reports throughout the 1980s and 1990s continued to endorse these broad recommendations. During this time the infant feeding recommendations in the United Kingdom were broadly in line with the guidance from the World Health Organisation (WHO), which in the 1990 Innocenti Declaration recommended that all infants should be fed exclusively on breast milk from birth up to four to six months of age. Early in 2000, the WHO commissioned a systematic review of the published scientific literature on the optimal duration of exclusive breastfeeding. As a result of this review, the WHO revised its guidance to recommend exclusive breastfeeding for the first six months of an infant's life. This revised guidance was adopted by the United Kingdom Health Departments from 2003 onwards."[46]

Thus the UK Government recommends that all mothers should exclusively breastfeed until six months of age.[7] However, actual current UK Government infant feeding literature adopts a more pragmatic approach and encourages mothers to aim for "around" six months exclusive breastfeeding.[8]

Exclusive breastfeeding rates in practice

However, the Infant Feeding Survey 2005 provides clear data which shows that in practice, few mothers achieve either the strict or pragmatic target (Table 2).[46]

Table 2. Prevalence (%) of exclusive breastfeeding by country (2005)

	England	Wales	Scotland	N Ireland	UK
Birth	66	58	61	55	65
1 week	46	38	42	35	45
2 weeks	39	32	37	31	38
3 weeks	34	28	32	25	33
4 weeks	29	21	25	20	28
6 weeks	22	15	19	13	21
2 months (8 weeks)	18	12	17	11	18
3 months (13 weeks)	14	9	12	8	13
4 months (17 weeks)	8	4	6	4	7
5 months (21 weeks)	3	2	3	2	3
6 months (26 weeks)	<1	<1	<1	<1	<1

The figures are only slightly better when restricted to mothers who commenced feeding exclusively at their infants' birth. (Table 3)

Table 3. Duration of exclusive breastfeeding among mothers who fed exclusively at birth by country (2005)

	England	Wales	Scotland	N Ireland	UK
Birth	100	100	100	100	100
1 week	69	66	69	64	69
2 weeks	60	55	60	56	59
3 weeks	52	48	51	46	51
4 weeks	44	37	41	37	43
6 weeks	33	26	31	24	32
2 months (8 weeks)	28	21	28	21	27
3 months (13 weeks)	21	16	20	15	21
4 months (17 weeks)	12	7	10	8	12
5 months (21 weeks)	5	3	4	4	5
6 months (26 weeks)	1	<1	1	<1	<1

Thus by stating in the EAT study eligibility criteria that mothers had to have achieved four months of exclusive breastfeeding before being eligible to participate would reduce the number of eligible women by 43% (England 14% to 8% Table 2).

Actual age of introduction of solids:

The actual mean age of introduction of solids in the UK in the Millennium Cohort Study was 3.8 months.[45] This figure is almost identical to that observed in the Infant Feeding Survey 2005 where 51% of infants were reported to have received complementary foods before four months of age. Thus our age of introduction of complementary foods in the early introduction group of the EAT study reflects what the majority of parents are doing with their infants in reality.

No evidence of harm from solid introduction from three months of age:
Concerns about early introduction of solids in studies undertaken in the 1970's related to two issues: excessive dietary solute load from formula milk and/or solids; and excessive weight gain. One study from 1973 looking at the former found that infants fed artificial milk formula alone or solids and artificial milk formula had higher mean plasma osmolality and a greater percentage of values in the hyperosmolar range than infants who were breastfed.[47] However, the "healthy" group in this comparison were infants of between one and three months of age who were being breastfed. In the EAT study all infants will be receiving three complete months of exclusive breastfeeding. A classic study looking at the latter (cited 150 times) found that artificially fed six week old infants in the Sheffield region were heavier than would be predicted from their birth weights and showed a greatly increased incidence of excessive weight gain.[48] Again, the infants of concern were receiving artificial feeds much earlier than the age at which solids will be introduced in our study.

Early occurrence of sensitization to allergenic foods in infants – the LEAP experience
Emerging data from the LEAP study reveals that infants are sensitized far earlier than previously realized. At the point at which 547 infants had been enrolled onto the study, of 115 infants under six months of age otherwise eligible to participate, 12 (10.4%) had had to be rejected as they were already too significantly sensitized to peanut (>4mm skin prick test result).

The percentage of infants participating in the LEAP study with protocol defined egg allergy is shown in Figure 1. None of the infants under six months will have actually started consuming egg at this point and yet approaching half have egg allergy.

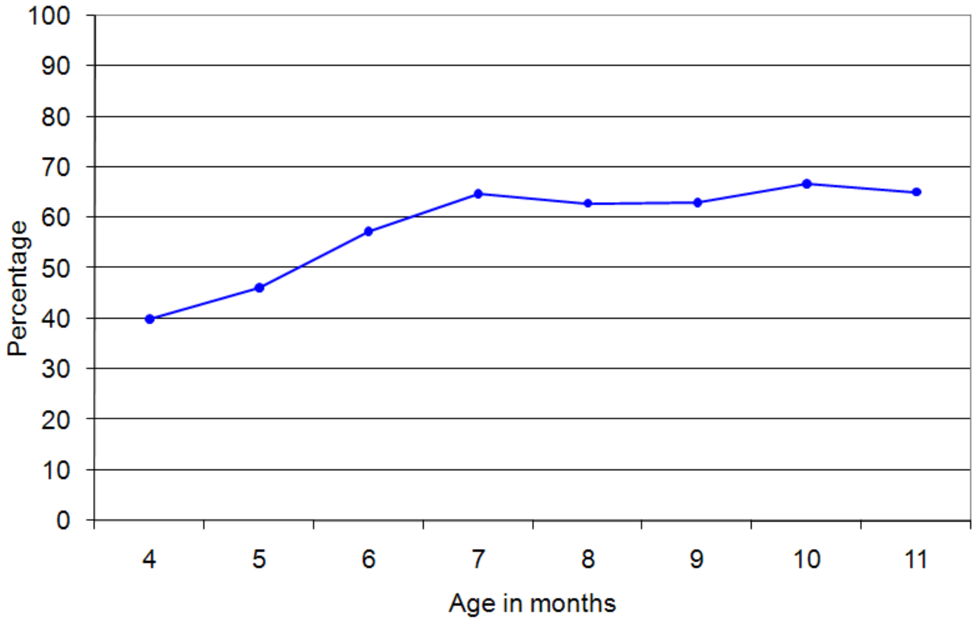

Figure 1. Protocol defined egg allergy at enrolment into LEAP study.

The results of sensitisation rates on specific IgE measurements at enrolment to three of the major food allergens: egg, peanut and cow's milk are shown in Figure 2. 22% of four month olds are sensitised to peanut with IgE levels greater than 0.35 KU/L. However, the figure for any specific IgE to peanut is even higher with 40% of four month olds having levels greater than 0.1 KU/L).

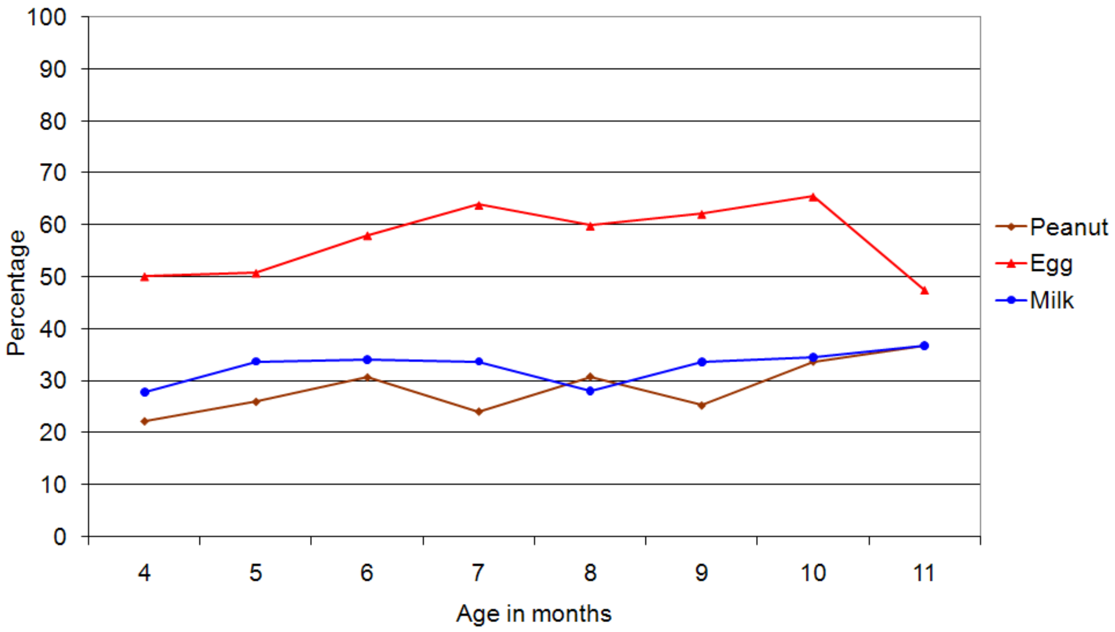

Figure 2. Sensitisation to allergenic foods (IgE>0.35KU/L) at enrolment onto the LEAP Study.

1.3.5 JUSTIFICATION FOR SKIN PRICK TESTING INTERVENTION CHILDREN ONLY AT THREE MONTH ENROLMENT VISIT

The aim is for mothers in the standard introduction arm to introduce complementary foods to their children in as similar a manner as possible to what they would have done if they were not participating in the study. The principle and most recent UK Government advice has been incorporated into a tailored dietetic educational booklet which will be provided to all mothers in the standard introduction arm and the study dietitians will provide support with regard to implementing the recommendations. The government infant feeding leaflet will be made available if mothers would like a copy.

Skin prick testing the standard introduction children would be very likely to change the mothers' infant feeding behaviour significantly. Similarly, whilst blood will be taken from all infants at three months of age, specific IgE levels to the six intervention foods will not be measured until the child reaches the end of the study. This ensures that no ethical dilemma is posed by being in possession of information about an individual child's sensitisation which is not being shared with the parent. Just as with the skin prick test results, a mother would not have specific IgE measurements available to influence her feeding regimen if she were not participating in the study.

1.3.6 JUSTIFICATION FOR ADMINISTERING COW'S MILK PROTEIN AS A SOLID (YOGHURT)

In the EAT study, the decision has been taken to give the cow's milk protein exposure as yoghurt rather than cow's milk based formula. The reason for this relates to the differential effect of solid introduction versus formula introduction on pattern and duration of breastfeeding.[49] Hörnell *et al* showed that the introduction of solids was associated with no or minor changes in breastfeeding frequency and suckling duration. Breastfeeding frequency remained constant the first month after the introduction and then declined slowly, while daily suckling duration started to decline slowly when solids were introduced (Figure 3). However, in infants given formula, as soon as regular formula feeds started, the breastfeeding frequency and suckling duration declined swiftly.[49]

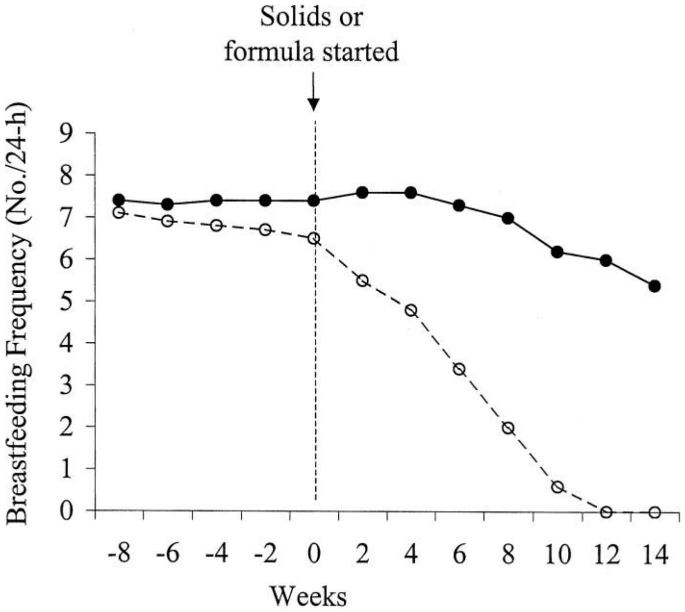

Key: Data are shown from 8 weeks before the start of solids or regular formula feeds to 14 weeks after the start. The 14-day period when solids and regular formula feeds were started is set to zero. ● indicates the solids group; ○ formula group

Figure 3. The median breastfeeding frequency in the solids and formula groups.

Breastfeeding duration was not associated with infants' age at introduction of solids (Figure 4). This is reassuring and suggests that early solid introduction will not impair breastfeeding performance.

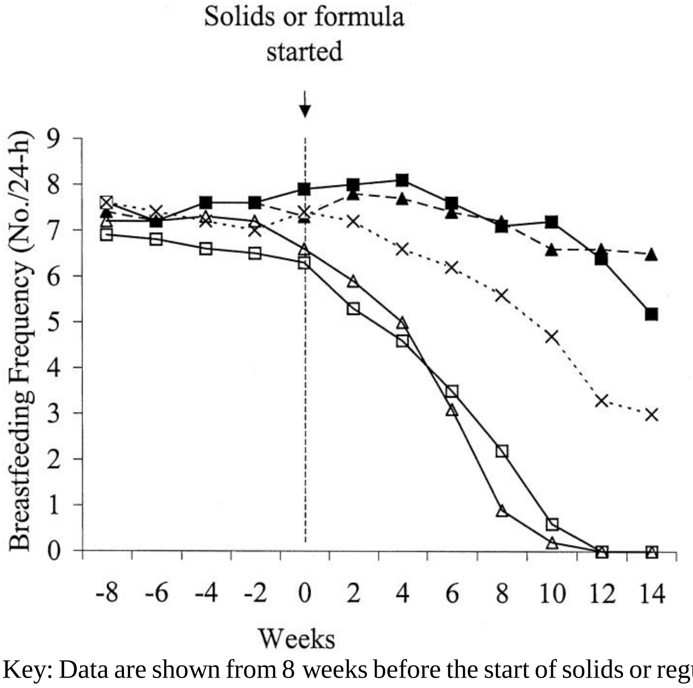

Key: Data are shown from 8 weeks before the start of solids or regular formula feeds to 14 weeks after the start. The 14-day period when solids or formula was started is set to zero. Solids: ■ = <4 months; ▲ = 4 to 6 months; × = 6 months. Formula: □ = <4 months; Δ = 4 months (includes three infants starting with regular formula at 6 months).

Figure 4. The median breastfeeding frequency by age at introduction of solids in the solids group and of formula in the formula group.

Allergenicity of cow's milk protein as yoghurt compared with cow's milk based formula

One study has evaluated whether patients with milk allergy could tolerate extensively heated milk products.[50] The study includes a section explaining the relationship between food processing (including heating) and allergenicity:

"Individuals with food allergy may generate specific IgE antibodies against conformational (dependent on tertiary structure) and sequential (linear) epitopes.... The allergenicity of food proteins may be altered by processing. High heat (e.g. baking) was found to reduce allergenicity of many food proteins, presumably by altering the conformation of heat-labile proteins that results in loss of conformational epitopes.[51] The classic examples are birch tree pollen allergen Bet v 1 cross-reactive proteins in apple (Mal d 1) and carrot (Dau c) that in the uncooked form cause oral symptoms (pollen–food allergy syndrome) but after heating are readily tolerated. In contrast, Bet v 1 cross-reactive protein in soybean, Gly m 4 retains allergenicity in heat-processed foods, suggesting that thermostability is highly variable and food-specific, even for the food allergens from the same protein family. Alternatively, in the case of peanut proteins, high temperature may enhance allergenicity as a result of glycation (the Maillard reaction) that induces the formation of Ara h 2 aggregates that are more resistant to gastric digestion and bind IgE antibody more effectively than unheated Ara h 2. Behavior under heating conditions is one of the determinants of allergenicity and may explain the different sensitizing potential of related foods, such as peanut and soybean. In addition to an individual susceptibility to heat and digestion, interactions with other substances present in a complex food, collectively referred to as the food matrix effect, may be crucial. Published data indicate that heating decreases but does not completely eliminate milk allergenicity. The caseins and a-lactalbumin have a higher heat stability than the other whey proteins, b-lactoglobulin and serum albumin. After heating milk at 100°C for ten minutes, a substantial reduction of allergenicity was noted. Our studies evaluating the effects of heating (time and temperature) and food matrix on allergenicity of casein and whey proteins are ongoing." [50]

In this study unheated milk products used included unheated milk or yoghurt. In producing yoghurt milk is heated to 85°C transiently (to pasteurise the milk) and then cooled to 43°C. This is virtually identical to the pasteurisation process that store bought milk and the cow's milk in infant formula undergoes. This is high-temperature short-time pasteurization which involves heating the milk for a short period (heating to approximately 75°C for up to 30 seconds).

1.3.7 JUSTIFICATION FOR REVIEWING ALL INFANTS AT ONE YEAR OF AGE

We considered only reviewing a high risk sub-group at one year of age. However this fails to circumvent the problem of mothers in the standard introduction arm who choose not to introduce an allergenic food into their child's diet before the three year assessment. A proportion of these children may have had an allergy to particularly cow's milk or egg which was transient and will therefore no longer be present at the three year assessment. By seeing all children at one year of age and screening them for IgE mediated food allergy to the six intervention foods with skin prick testing this ensures children in both arms of the study are treated identically from this point onwards. This overcomes the inherent design difference between the intervention and standard introduction arms in the first year of life, specifically that intervention infants will manifest their IgE mediated food allergy in the first year of life as they are having all six foods introduced into their diet, whereas standard introduction infants are likely to not be consuming most of the allergenic foods in the first year of life (based on the Infant Feeding Survey 2005 experience).

1.3.8 JUSTIFICATION FOR THE THREE YEAR ASSESSMENT AS THE PRINCIPAL OUTCOME

Most children who develop food allergy will have done so by the age of three. Indeed in the MAS study the sensitization rate to egg and milk had plateaued by 1 year of age, with virtually no new cases beyond three years of age.[52] With regard to the egg and milk allergy, the assessment is likely to be more accurate as these children will be assessed more contemporaneously with their condition.

Measuring objective lung function at three years of age is difficult but, in contrast, it is

straightforward to determine the prevalence of the atopic wheeze phenotype. The atopic wheeze phenotype children are particularly important as Martinez was the first to show in his seminal paper that they are the children who will progress to develop persistent asthma.[53] This important observation has since been replicated in older children followed through to adulthood.[54] The most recent publication was a paper from the MAS cohort that showed that sensitisation to perennial allergens developing in the first three years of life was associated with a loss of lung function at school age.[55] From the Tuscon data the proportion of children that commenced wheezing after the age of three was only 15.0% (124/826).[56] The MAS cohort has shown that from the third birthday on, specific IgE levels to inhalant allergens were significantly higher than specific IgE levels to food allergens in children of the same age.[52] Further funding will still allow subsequent respiratory function assessment at six years of age.

1.3.9 JUSTIFICATION FOR THE CHALLENGING ALL SKIN PRICK POSITIVE CHILDREN AT ONE AND THREE YEAR ASSESSMENTS

There was concern that any algorithm to assess for the presence of IgE mediated food allergy at the one and three year assessments that took into account current consumption of the allergenic foods in question would introduce a bias into the study. This is because the early introduction group would consist entirely of current consumers, whilst the standard introduction arm would be a mixture of non-consumers and consumers – the proportion of each varying depending on the allergenic food. In order to circumvent this, an algorithm for screening the children for possible IgE mediated food allergy to the six allergenic foods based solely on their skin prick test results at the one and three year assessments was considered desirable. In order to estimate the likely distribution of skin prick test results at these ages, data from the Food Allergy and Intolerance Research (FAIR) study was kindly shared with the EAT study team (Table 4).[57]

Table 4. Distribution of skin prick results in FAIR cohort at one and three years of age

FAIR Data		SPT size (mm)			
		**0<br>%**	**>0 - 2<br>%**	**>2 - <5<br>%**	**5+<br>%**
Milk	1 year	98.4%	0.9%	0.4%	0.3%
	3 year	99.2%	0.3%	0.3%	0.2%
Wheat	1 year	99.7%	0.3%	0.0%	0.0%
	3 year	96.9%	1.6%	1.3%	0.3%
Egg	1 year	95.9%	2.1%	1.2%	0.8%
	3 year	98.0%	0.5%	0.5%	1.1%
Fish	1 year	98.8%	0.9%	0.3%	0.0%
	3 year	99.1%	0.5%	0.2%	0.3%
Peanut	1 year	98.0%	1.4%	0.4%	0.1%
	3 year	97.0%	0.5%	1.1%	1.4%
Sesame	1 year	98.6%	0.9%	0.4%	0.1%
	3 year	97.7%	0.9%	0.0%	0.6%

The FAIR study recruited a whole birth cohort of children (approximately 1,000) born on the Isle of Wight between 1st September 2001 and 31st August 2002 to provide information on the prevalence and incidence of food allergies and intolerance. The children were followed up at the ages of one, two and three years and a diagnosis of food allergy or intolerance made by a combination of history, skin tests and food challenges. These prevalence rates were extrapolated to the anticipated size of the EAT cohort at age one (2200 children) and age three (2000 children) to determine the likely number of children with positive skin prick test results (Table 5). The amended numbers (in light of the September 2010 contract variation - see Section

3.1.1.3) are given in red in Table 5 and assume 1172 children at age one and 1106 children at age three.

Table 5. Likely distribution of skin prick results at one and three years of age based on extrapolating FAIR data to the EAT Study

EAT Estimate		SPT size (mm)			
		0<br>N=	>0 - 2<br>N=	>2 - <5<br>N=	5+<br>N=
Milk	1 year	2165<br>1153	20.2<br>10.5	8.7<br>4.7	5.8<br>3.5
	3 year	1984<br>1097	6.3<br>3.3	6.3<br>3.3	3.1<br>2.2
Wheat	1 year	2194<br>1168	5.8<br>3.5	0.0<br>0.0	0.0<br>0.0
	3 year	1937<br>1072	31.3<br>17.7	25.0<br>14.4	6.3<br>3.3
Egg	1 year	2110<br>1124	46.2<br>24.6	26.0<br>14.1	17.3<br>9.4
	3 year	1959<br>1084	9.4<br>5.5	9.4<br>5.5	21.9<br>12.2
Fish	1 year	2174<br>1158	20.2<br>10.5	5.8<br>3.5	0.0<br>0.0
	3 year	1981<br>1096	9.4<br>5.5	3.1<br>2.2	6.3<br>3.3
Peanut	1 year	2157<br>1149	31.8<br>16.4	8.7<br>4.7	2.9<br>1.2
	3 year	1941<br>1073	9.4<br>5.5	21.9<br>12.2	28.1<br>15.5
Sesame	1 year	2168<br>1156	20.2<br>10.5	8.7<br>4.7	2.9<br>1.2
	3 year	1953<br>1081	18.8<br>10.0	0.2<br>0.0	12.5<br>6.6

Finally the likely number of food challenges required at one and three years in the EAT Study based on the extrapolated data was calculated using two different screening thresholds: all positive results and >2mm (Table 6). Again, the amended numbers (in light of the September 2010 contract variation - see Section 3.1.1.3) are given in red in Table 6.

Table 6. Likely number of food challenges required at one and three years of age based on extrapolating FAIR data to the EAT Study using two thresholds: all positive results and >2mm

Number of challenges	1 year		3 years	
	Any positive	>2mm	Any positive	>2mm
Milk	35<br>19	14<br>8	16<br>9	9<br>5
Wheat	6<br>3	0<br>0	63<br>35	31<br>18
Egg	90<br>48	43<br>23	41<br>23	31<br>18
Fish	26<br>14	6<br>4	19<br>11	9<br>5
Peanut	?*<br>?*	?*<br>?*	59<br>33	50<br>28
Sesame	?*<br>?*	?*<br>?*	31<br>17	13<br>7
Total	156<br>84	63<br>35	228<br>128	144<br>81
Per month	6.5<br>3.0	2.6<br>1.2	9.5<br>4.6	6.0<br>2.9
% of cohort	7.1%<br>7.2%	2.9%<br>3.0%	11.4%<br>11.6%	7.2%<br>7.3%

* Number of challenges to peanut and sesame at one year of age uncertain as determined by counsumpton status

Based on Table 6, using the more sensitive (but less specific) screening criterion of any positive skin prick test result, 7.1% of the cohort (156 children) would require challenges at the one year assessment (7.2%, 84 children revised figures) and 11.4% of the cohort (228 children) at the three year assessment (11.6%, 128 children revised figures). The monthly workload induced by this level of challenges is manageable even allowing for a potential doubling in these estimates to take into account the possibility of recruiting a cohort at higher risk of allergic disease than the Isle of Wight cohort.

1.3.10 AMENDMENT TO EAT FOOD CHALLENGE PROTOCOLS (JUNE 2011)

1.3.10.1 RATIONALE FOR AMENDMENT

By 09 March 2011, the EAT Study had conducted 16 double blind placebo controlled food challenges (DBPCFCs) related to the one Year EAT Study scheduled clinic visits. An audit was taken of these challenges and the results are presented in Tables 7 and 8.

Audit findings
Although the findings may change in the future as a greater number and variety of DBPCFCs for the different intervention foods are performed, the following conclusions can be made from the challenge experience so far:

- A significant percentage of the total number of challenges (31.3%) are ending with an indeterminate result.
- The number of DBPCFC egg challenges currently exceed that of DBPCFC cow's milk challenges
- All the indeterminate results were found in the DBPCFC egg challenges.

- Participants with indeterminate challenge results varied from 14-16 months of age, with the majority stopping after 4-5 blinded doses.

The latter observation rules out the possibility that it is the younger ages (for example 12-13 months of age) that are having an issue with completing the challenges fully, and also suggests that the total volume of food provided may be an issue for a certain proportion of children. This is despite the fact that the challenge material used for egg has been worked to be as condensed as possible, to maximise the amount of food protein provided with the least amount of masking vehicle as possible.

Table 7. Summary of results of audit performed 09.03.11 on all 1 year visit DBPCFCs conducted

**Allergenic Food Tested**	**Consuming Status**	**No. of challenges conducted**	**Age Challenged (in months)**	**Gender (M, F)**	**Final DBPCFC result**
Egg	Infrequent/Never	10	13 ¼ - 16 ½	M (7)<br>F (3)	Positive (6)<br>Negative (1)<br>Indeterminate (3)
Egg	Frequent	2	14 - 15 ¾	M (1)<br>F (1)	Indeterminate (2)
Milk	Infrequent/Never	4	13 - 14 ½	M (4)	Positive (4)

Table 8. Further analysis of challenges deemed indeterminate

Total number of DBPCFCs performed by audit date:	16 (12 egg, 4 milk)
Number of indeterminate challenges:	5 (all from egg)
Percentage of all milk and egg challenges (n=16) performed with indeterminate result:	31.3%
Percentage of the egg challenges (n=12) with an indeterminate result:	42.7%
Percentage of infrequent/never consumer egg challenges (n=10) with indeterminate result:	30%
Percentage of frequent consumer egg challenges (n=2) with indeterminate result:	100%

Original food challenge protocol regime

When examining just the DBPCFC egg challenges, this shows that almost half (43%) of the challenges done to the audit date had been indeterminate. This is likely due to the original protocol specifying children classified as ‘infrequent/never’ consumers at the one year visit would need to consume 7 blinded doses (4 active doses and 3 placebo doses) equivalent to 2.85 grams of food protein, followed by an open dose of 3 grams of food protein. Children classified as ‘frequent’ consumers at the one year visit would need to consume 5 blinded doses (3 active doses and 2 placebo doses) equivalent to 3.85 grams of food protein, followed by an open dose of 2 grams of food protein. In both situations, the total food protein intake is 5.85 grams of protein.

Currently, though it is considered the gold standard for egg challenges to use raw egg since this is the most allergenic form, for our challenges we have chosen to use raw pasteurised whole hen’s egg powder to avoid the risk of salmonella infection. The corresponding amount of powder (to be equivalent to the desired egg protein in grams) is then mixed with a hypoallergenic amino acid based powder and a natural colouring agent to mask the appearance between the placebo

and the active versions. On the day of the DBPCFCs, each dose is then mixed with a minimal amount of a masking food (usually a fruit or vegetable puree) before it is offered to the participant. However, if each dose is mixed with at least a few teaspoons of masking solids, the cumulative quantities given to a one year old over 5-7 doses can be substantial, especially if the child also needs to eat an open final portion at the end.

As of the audit date, no DBPCFCs to wheat or fish had been performed as part of the one year EAT Study visit, but identified participants were scheduled to return in the following months to have these challenges performed.

Other issues with original EAT food challenge regime
There are a number of other issues with the original challenge regime:

- Duration: The challenges performed up to the audit date had been found to be very time and labour intensive, taking up to an average of 6-7 hours to complete (especially if a young child needs to have a nap in between the doses).
- Inconclusive: It is inefficient in terms of staff time and expense to have indeterminate DBPCFC results as this means the same participants would need to be brought back at a later date to repeat the food challenge(s).
- Perceived pointlessness: A number of 'frequent' consuming children in both the early introduction and control groups have been encountered who have had positive skin prick tests to a particular EAT Study intervention food at the one year visit. These participants have had to be brought back for a food challenge, since on the day of the one year clinic visit it has been too late in the day to start a DBPCFC to the particular intervention food(s). However, it has been difficult to persuade these mothers to return for an unscheduled clinic visit as parents often feel it is rather pointless to bring a child back for a food challenge to a food they are currently consuming regularly and in decent quantities in their diets.

1.3.10.2 DETERMINATION OF AGE APPROPRIATE PORTIONS

The principal goal of the EAT Study oral food challenges is to ascertain that a participant can tolerate an **age appropriate portion** of the allergen containing food with cow's milk dairy, hen's egg, peanut, sesame, wheat or fish on the study visit date and present no IgE-mediated reactions, so that they will be able to return home and continue consuming age appropriate portions of this food on a regular basis.

This is consistent with the recent 'Work Group report: Oral food challenge testing' by the Adverse Reactions to Food Committee of the American Academy of Allergy, Asthma and Immunology which emphasises that since **the goal of the oral food challenge is to test the patient's tolerance to a specific food in the quantity and form that will be subsequently given in the diet, the total quantity tested should approximate the regular, age-appropriate serving size of the food**.[58] If the age appropriate portions of United Kingdom (UK) children at one year and three years of age are determined, then the grams of food protein found in these portions can be calculated and the overall challenge design and dosages can be more accurately determined.

Portion size – literature review
Unfortunately, determining typical food portion sizes for one and three year old UK children is a challenging task. It is widely recognised that assigning a typical or average portion to all toddlers is difficult, since the appetite and intake of any one individual toddler can vary enormously on a day to day basis, and variations also exist between toddlers based on their age, gender and activity levels. A review was conducted of current available sources related to the portion or serving sizes of UK children:[59-63]

- Caroline Walker Trust (CWT) recommendations
- Infant and Toddler Forum

- National Diet and Nutrition Survey (NDNS) data: This is mainly based on weighed food records for children between 1 ½-4 ½ years of age obtained between 1992-1993, as data is not available yet from the rolling NDNS programme).

Not surprisingly, these sources vary slightly in their recommendations and some limitations are also noted (for example, typical food portion weights in grams from the NDNS data are only reported for the combined age range of one to three years, and this data is slightly dated though it is based on weighed food records and a large nationally representative sample).

UK one and three year old children portion size – conclusion

However, after further consultations with various UK experts in the field of paediatric nutrition and allergy, with expertise and experience working with food portion sizes, it was determined to use the following estimated portion sizes for one year and three year old children for the six EAT Study intervention foods (Table 9).[64-66]

Table 9. Estimated age appropriate portion sizes for UK children 1 year and 3 years of age

Intervention Foods	1 Year of Age	3 Years of Age
Cow's milk	100 ml	110 ml
Egg	½ medium boiled egg	¾ medium boiled egg
Fish	1 fish finger	1 ½ fish fingers
Wheat	½ slice of bread	¾ slice of bread
Peanut	1 ½ teaspoons smooth peanut butter	2 ¼ teaspoons smooth peanut butter
Sesame	1 tablespoon houmous	2 tablespoons houmous

These portion sizes are based largely on the portion sizes listed on the Infant and Toddler Forum website, as these are derived from a combined analysis of the CWT, NDNS, Avon Longitudinal Study of Pregnancy and Childhood (ALSPAC) data, expert clinical experience and then further modified based on expert recommendations.

Based on these estimated age appropriate portion sizes, the tables below list the average grams of food protein (and the dry food equivalent) present in a sampling of products currently and commonly available in the UK market, using semi-skimmed milk, a medium lion stamped egg, standard sized 100% cod fillet fish fingers, medium sliced wholemeal bread, smooth peanut butter and plain full-fat houmous containing on average 13% tahini in its ingredients (Table 10 and Table 11).

Based on these findings, it can be seen that the grams of protein for the different portion sizes at each age varies widely from 0.5-3.5 grams of food protein for one year olds to 1.0-4.88 grams of food protein for a three year old just looking at examples of the six intervention foods. The dried food equivalents were calculated after comparing the protein content reported for 10 grams of selected dry food products in the Nowak et al. paper with the protein content of selected dried food products currently being used or available on the UK market.[58] For skimmed milk powder and whole hen's egg powder, the protein content was found to be equivalent. For wheat flour, it was decided to use the value given in the Nowak et al. paper (1.3 grams of protein per 10 grams of wheat flour) after taking into account the fact that in the UK there are many wheat flour brands and products which can differ greatly from as much as 0.94 grams to 1.52 grams of protein per 10 grams of flour. For peanut flour, we have chosen to report the protein content of the product we are currently using (12% fat light roast peanut flour with a minimum of 50% peanut protein from Old Virginia Byrd Mill), as the value reported in the Nowak et al. paper was slightly higher (5.25 grams of protein per 10 grams of peanut flour).

Table 10. Approximate grams of protein in estimated age appropriate portion sizes for a 1 year old

Intervention Foods	1 Year of Age	Protein (g)	Grams of dry food (g)
Cow's milk	100 ml	3.5 g	9.7 g skimmed milk powder
Egg	½ medium boiled egg	3.25 g	6.8 g whole hen's egg powder
Fish	1 fish finger	1.96 g	No available data
Wheat	½ slice of bread	2.1 g	16.2 g wheat flour
Peanut	1 ½ teaspoons smooth peanut butter	2.1 g	4.2 g defatted peanut flour
Sesame	1 tablespoon houmous	0.5 g	No available data

Table 11. Approximate grams of protein in estimated age appropriate portion sizes for a 3 year old

Intervention Foods	3 Year of Age	Protein (g)	Grams of dry food (g)
Cow's milk	110 ml	3.85 g	10.7 g skimmed milk powder
Egg	¾ medium boiled egg	4.88 g	10.2 g whole hen's egg powder
Fish	1 ½ fish fingers	2.94 g	No available data
Wheat	¾ slice of bread	3.11 g	23.9 g wheat flour
Peanut	2 ¼ teaspoons smooth peanut butter	3.09 g	6.2 g defatted peanut flour
Sesame	2 tablespoons houmous	1 g	No available data

Food Challenge Design –review

Before determining the amendments needed for the challenge protocols, a review was undertaken of food challenge practice in other studies as well as in experienced allergy units, focusing on those relevant to children between one and three years of age. This review found a wide variation in the methods used as well as the amount of food protein challenged. There were also limited studies on successful oral challenges conducted for young children, especially those being conducted using DBPCFCs. The findings are summarised in Table 12.[58;64;67-71]

Open versus DBPCFC

The methods used (open oral challenges versus DBPCFCs) and the ages in which these challenges are performed differ considerably. For example, at Mt. Sinai Medical Centre, DBPCFCs are only conducted in children over two years of age.[64] This is similar to the currently ongoing STEP/STAR randomised trials studying whether early introduction of egg from four months of age can reduce prevalence of IgE-mediated allergy to egg at one year of age, where only open challenges are performed as the end point in children who turn 12 months of age.[71] In the LEAP Study, two challenge protocols exists at the endpoint when participants turn five years of age. If a participant is deemed low risk (SPT and clinically negative to a possible IgE-mediated peanut allergy), then an open dose of 5 grams of peanut protein will be given. However, in all other circumstances, a LEAP participant would need to undergo a condensed DBPCFC (containing both blinded placebo and active doses) including an open final dose in one day, with a total intake of 9.35 grams of peanut protein.[66]

Table 12. Summary of differences between selected studies and clinical practice areas.

	Name of Study/Hospital				
	**Clinical & Research Practice at Mt Sinai Medical Centre, USA**	**Comparison of open vs double blind food challenges (Venter et al, 2007)**	**EuroPrevall**	**STEP/STAR trials (Debra Palmer, Sue Prescott)**	**LEAP Study**
**Challenge Type**	-Do not conduct DBPCFC in children under 2 years<br>-2 separate days (1 day: placebo, 1 day: active, with an open top dose of allergen given at the end of 2nd day)	-Various challenge protocols<br>-'1 day' DBPCFC for immediate reactions: conducted on 2 separate days (1 day: placebo, 1 day: active, with a normal portion of food containing allergen given at the end of 2nd day)	-DBPCFCs are conducted on 2 separate days (1 day: placebo, 1 day: active)<br><br>-No open final dose is given of an age appropriate portion	-Only open challenges are performed<br>2 challenge protocols:<br>-Low risk (SPT neg): 1 dose ½ egg equivalent given<br>-High risk: ½ egg equivalent split into 6 doses	-2 challenge protocols (1 open and 1 DBPCFC):<br>-if SPT neg, 5g protein as 1 open dose<br>-DBPCFC: 4.35g pro (blinded) + 5g open top dose = total 9.35g pro
**Age Range**	> 2 yrs DBPCFC	1-15 years of age	Infants under 6 months to 30 months	12 months of age	5 years of age (v. 60 appointments)
**Milk**	For DBPCFC, use 10 grams of dried food as per Nowak et al. paper, then give final open normal portion of food (spread out in 2-3 portions over 30-60 min)	Portions of food equivalent to 8-10g of dried food were given, then a final open normal portion of food on the 2nd day after a 2 hour observation period	4.3g protein	--	--
**Egg**			4.3g protein	Raw pasteurised egg powder (3 tsp) equivalent to ½ egg	--
**Peanut**			4.3g protein	--	5g or 9.35g protein
**Wheat**			4.3g protein	--	--
**Fish**			4.3g protein	--	--
**Sesame**			--	--	--
**Comments/Notes**	Since DBPCFC are not conducted in children under 2 years of age, smaller portions for 1-2 year olds are not used.	-For children who refuse the normal portion of food, parents were asked to give a normal portion of this food to the child at home, on the same day.<br>-Out of 8 positive '1 day' DBPCFCs, six were done in 1 year olds	-Statistically determined	-Not conducting DBPCFC due to expense.	-Under 3 years of age (36 months), 4g of protein are given in an open challenge for unscheduled clinic visits

Duration of challenge (one or two days)

It is worth noting though, that in the majority of instances (in the clinical and research practices at Mt. Sinai Medical Centre, EuroPrevall study, and Venter et al. 2007 paper), DBPCFCs are usually conducted on 2 separate days, with one day given for the placebo doses and one day for the active doses. This arrangement, though more costly, would be beneficial as it allows a greater total amount of food protein to be provided incrementally, and a greater chance that the child can finish all the required doses, even if an open final dose is given at the end.

Total amount of food consumed

There appears to be even less consensus in terms of the total amount of food protein to be given in an oral food challenge, especially from one to three years of age. Of the selected studies, only EuroPrevall is conducting DBPCFCs in a large cohort of participants from six to thirty months of age, which is similar to the EAT Study. For EuroPrevall, the total quantity of food protein challenged in all the allergenic foods studied is a fixed 4.3 grams of food protein (note in addition that no open top final dose of the allergenic food in an age appropriate portion is given at the end).[67;68] At Mt. Sinai Medical Centre, the clinical and research practice is to follow what is laid out in the Nowak et al. work group report on oral food challenge testing which states that in one approach, the practice is to use 8-10 grams of a dried food (like milk powder, egg powder, peanut powder, wheat flour) or 16-20 grams of meat/fish, followed by an age appropriate serving. However, when the actual grams of food protein are calculated from 10 grams of a dried food, it is found that the protein quantities vary widely from 1.3 grams of wheat protein to 3.6 grams of milk protein, to 5.25 grams of peanut protein.[58;64]

1.3.10.3 PROPOSED AMENDMENTS TO EAT FOOD CHALLENGE PROTOCOLS

Due to size of the geographic catchment area of EAT recruits (throughout England and Wales), undertaking anything other than a one day challenge procedure for the one year and three year EAT Study visits is not feasible. Our aim is therefore to combine placebo and active doses all in one day, and include a top open dose similar to an age appropriate portion size, while ensuring that the total quantity to be ingested remains reasonable. This ensures each participant will be able to eat the entire amount given during the challenge and complete the challenge within a reasonable time frame including a two hour observation period. Challenge protocols would be needed that take into account the large possible variability in appetites and typical intakes, so as to minimise possible indeterminate results. The following table summarises the proposed amendments to the EAT Study challenge protocols at the one and three year visits, based on the estimated age appropriate portion sizes for one and three year old UK children and the corresponding approximate grams of food protein contained in these portions (Table 13).

Table 13. Summary of proposed amendments to EAT Study challenge protocols at one and three year visits.

	1 year	3 year
Frequent Consumer: one open dose	4.3 grams of food protein	5.3 grams of food protein
Infrequent/Never Consumer: Blinded doses (3 active & 2 placebo doses) + open dose	1.8 grams protein (in blinded section) + 2.5 grams protein (in open dose) = 4.3g protein total	1.8 grams protein (in blinded section) + 3.5 grams protein (in open dose) = 5.3g protein total

Proposed amendment – amount consumed

In these amended challenge protocols, it is proposed to keep the total grams of food protein challenged for the six intervention foods the same at 4.3 grams of food protein for the one year EAT Study visits, and at 5.3 grams of food protein for the three year EAT Study visits. This would be similar to the current challenge protocols in that a fixed amount of grams of food

protein is given at the one year and at the three year visit, although the overall total grams of food protein given would be slightly lower at each of these visit points. The total grams of food protein given at the one year visit would also be equivalent to the amount challenged for participants between 6-30 months in the EuroPrevall study. The two tiers of challenge protocols will be kept, depending on the frequency of consumption of the allergenic food in question (see Section 3.6).

Proposed amendment – number of doses for frequent consumers

A key change will be in the number of doses given in both amended challenge protocols. Under the existing 'frequent' consumer challenge protocol, participants consume a total of 5 blinded doses (3 active and 2 placebo doses) as well as an additional open dose of 2 grams of food protein at the one year visit and 4 grams of food protein at the three year visit. In the amended protocol, it is proposed that this be changed to an open oral challenge of one dose of an allergenic food with a total of 4.3 grams of food protein at the one year visit and 5.3 grams of food protein at the three year visit. This change will make the total grams of food protein to be ingested more achievable. The benefits of such a change are numerous:

- Immediacy: Any EAT participant coming for the 1 year visit with a positive SPT to an intervention food and classified as a 'frequent' consumer, would be able to undergo an oral open challenge on the spot to the food in question.
- Cost and labour: The amendments would significantly reduce the overall length of the visit, and reduce any additional expense, staff time and efforts needed in having to reschedule and arrange another day for the participant to come back.
- Improved parental compliance: The proposed change would also increase participant compliance as parents are more likely to agree to an open challenge there and then, instead of having to make necessary travel and work arrangements to be able to return to the clinic on another day.

Acceptable food vehicle: For these participants classified as 'frequent' consumers, this proposed benefit would also be advantageous as participants would be only asked to consume an open portion of food in a form that they are familiar with and enjoy on a regular basis (for example full-fat cow's milk or yoghurt).

Proposed amendment – number of doses for infrequent/never consumers

For participants classified as 'Infrequent or Never' consumers with a positive SPT to an intervention food (this does not apply to participants in the standard introduction group with a positive SPT result to peanut or sesame), the current protocol stipulates that these participants would need to undergo a DBPCFC consisting of 7 blinded doses (4 active and 3 placebo doses) and an open top dose of 3 grams of food protein at the one year visit and 5 grams of food protein at the three year visit.

It is proposed that this be amended to 5 blinded doses (3 active and 2 placebo doses) and an open final dose of either 2.5 grams of food protein or 3.5 grams of food protein will be given at the one year and three year visits respectively. The blinded active doses will be increased incrementally: 0.1 grams then 0.4 grams and finally 1.3 grams of food protein (total is 1.8 grams of food protein).

By keeping to a DBPCFC design, the challenge protocol in 'infrequent or never' consumers would help to reduce bias, false positive challenges and possible subjective symptoms to a certain extent, though in children under 3 years of age, the likelihood of subjective symptoms is very low.[64;66;69] To ensure the rigorousness of this DBPCFC design, the minimum recommended ratio of placebo to active doses should be kept to 2:3, as this would reduce the likelihood of participants (or the parents or staff) guessing the right sequence of the challenge by chance from 50% (as in the case of 1 active and 1 placebo session) to about 5%.[58]

In addition, it would be important to have an open incremental challenge regime ready for identified participants who persistently refuse to take the blinded doses in the DBPCFCs (e.g. faddy/fussy eaters). This incremental open challenge regime would be similar in food protein to

the DBPCFC with 4 doses of the following amounts: 0.1 grams, 0.4 grams, 1.3 grams, followed by one final dose of 2.5 grams of food protein (at the one year visit) or 3.5 grams of food protein (at the three year visit).

For ‘infrequent or never’ consumers with a negative challenge result in either arm of the study, a goal of an age appropriate portion twice a week (for example, for egg this would be aiming for a portion of at least ½ an egg twice a week for children between 1-3 years of age) will be advised.

Confirmation of this reintroduction and the absence of symptoms within 6 weeks of the challenge will be obtained, to ensure that they are indeed eating and tolerating consumption of the recommended amounts in estimated age appropriate portions. This will help to rule out any possible false negative challenge results.

Proposed amendment – summary
The overall effect of these proposed changes in both challenge protocols is a reduced length of time to complete the challenges and a smaller overall quantity of food that participants would need to ingest. This would result in a higher completion rate with more determinate results and improved compliance, as well as achieve the goal of determining if participants exhibit IgE-mediated reactions to consuming the set quantities of allergenic food in both challenge protocols.

Though it is recognised that the total grams of food protein participants in both challenge protocols will be asked to consume is larger in all cases to that found in an estimated age appropriate portion, it would be possible in both challenge scenarios to spread out the open top dose if needed into two or three smaller servings to be given over the space of 30-60 minutes, in accordance with certain established clinical and research practices.[58;64] This would greatly improve intake of the required amount of allergenic food for a young child with a small appetite, especially if the smaller servings can be slightly spread out over time and also possibly given in different forms (for example some full-fat cow’s milk and some cow’s milk yoghurt).

To encourage completion of either challenge protocol, we will also strongly encourage parents coming for the one and three year visits to ensure their children (regardless of whether they are considered high risk or low risk) either do not eat two hours prior to the visit appointment or at least have only a light breakfast if it is a morning appointment. This would ensure that the participants will be sufficiently hungry if a challenge needs to be performed on the visit day.

1.4 RATIONALE FOR IMMUNOLOGICAL ASSESSMENTS

Subject to further funding

We will investigate the immunological evolution of food allergy (and other allergies) using lymphocytes collected sequentially during the follow-up of the cohort. We anticipate that this will enable us to both identify and predict the development of allergic conditions based on in vitro lymphocyte responses to antigenic stimulation.

The lymphocyte work is divided into two broad themes: firstly, antigen specific responses including T cell proliferation in response to allergen stimulation, antigen-specific precursor T cell frequency, intracellular cytokine production, specific IgE or IgG4 or other isotype responses; and secondly, non-antigen specific responses including CD4-CD25+ve cell expression, FOX-p3 expression and other regulatory cell markers. The antigen specific responses are likely to be linked to the cumulative prevalence of the food allergy outcome whilst the non-antigen specific responses are likely to be related to overall atopic disease outcomes.

1.5 RATIONALE FOR COLLECTION OF STOOL SAMPLES (AUGUST 2011)

1.5.1 THE SCIENTIFIC BASIS FOR THE WORK PROPOSED

It has been observed that children who have a higher number of older siblings have less hay fever and eczema. It has been suggested that microbial transmission within families may be protective against allergic diseases.[72] Other indirect exposures to an enhanced microbial load, such as living on a farm, pets, and day care attendance have also been associated with a reduced allergy risk. In addition, the consumption of unpasteurised farm milk and endotoxin exposure have shown protective effects against allergic sensitisation and allergic disease in some settings.[73] However, whilst chronic infection with helminth parasites can have a protective effect on eczema and asthma where such infections are endemic, the search for specific viruses and bacteria conferring a protective effect against allergic disease in affluent countries has been largely disappointing.[74] The inverse association between allergies and indirect measures of microbial exposures is therefore likely to be due to a general increase in exposure to non-pathogenic microbes. This would also explain the risk increase in allergic disease associated with the use of broad spectrum antibiotics.{Flohr, 2005 263 /id} While asthma, hay fever, and eczema have received considerable attention in the context of the hygiene hypothesis, little is known about how microbial exposure, including the gut microflora, may influence food allergy. Two studies suggest a higher risk of food sensitisation in babies born by caesarean section compared to vaginal delivery.[75;76] One prospective cohort study has demonstrated that communal child-care under 6 months or age and living with an animal is associated with a reduced risk of egg-challenge proven allergy (Koplin 2011 EAACI Abstract). Diet, including the age of introduction of complementary foods is also likely to influence the bowel flora.[77;78] It has been postulated that the protective effect of unpasteurised milk is mediated via an influence on the bowel flora.[79] However, the mechanisms underlying these associations remain poorly understood and need to be explored as part of prospective infant cohorts.

The human microbiome and its assessment

A microbiome may be defined as the totality of living organisms, from microbes to viruses, which live in a specified environment. In relation to the human body, the differing physiological and anatomical environments mostly define the symbiotic communities which arise, which are dynamic over time.[80] For example, at least 160 species live in the intestine and perform key symbiotic functions, such as fermentation of food to maximise host nutritional extraction and regulation of inflammatory and immune-modulatory responses.[81;82]

The microbiome may be assessed using culture-dependent or culture-independent techniques. Culture dependent techniques are prone to a range of different biases. Culture-independent techniques lack many of these biases and identify organisms by detecting molecular signatures that are species specific in DNA extracted from cells in clinical samples.

Recent pyro-sequencing technological developments allow the characterisation of these sequences at a previously unprecedented scale. This “next-generation” sequencing allows a high proportion of sequences (around 500 bases in length) to be identified against a database of bacterial sequences giving a relatively less-biased assessment of what bacterial species are present. It should be noted that without prior sample treatment, this approach cannot distinguish between DNA from dead and living bacteria.

Associations between microbiota and allergic disease

The role that the postnatal microbial environment, in particular the gut microbiome, plays in directing the immune system towards tolerance and away from the Th2 phenotype characteristic of the foetus is only beginning to be understood. Indirect evidence for the role of the gut microbiota comes from comparison between faecal bacteria of children with and without eczema. Culture-dependent methods have demonstrated species differences between infants with and without eczema both during and preceding the onset of their disease.[83;84] Culture-independent techniques have shown that children with reduced intestinal diversity on faecal sampling are predisposed towards an eczematous phenotype.[85;86] Eczema has been postulated to predispose to food allergy and data from the EAT study has recently shown that the same filaggrin-variant genes predispose both to eczema and to food sensitisation.[75;87;88] Animal models are supportive of the notion that intestinal bacteria influence the immunological response to food antigens.

Animal models characterising how gut microbiota influence food tolerance

Germ free animal models show a predisposition towards developing food allergy. Germ free mice are primed towards an allergic phenotype, possibly as a result of their poorly developed gut-associated lymphoid tissue (GALT), with reduced numbers of intraepithelial T cells and IgA-secreting B cells.[89-91] Studies of germ free animals inoculated with specific gut commensals have demonstrated that gut microbiota are able to promote food tolerance if TGF-β secreting T cells are recruited to Peyer's patches.[92]

Putative mechanisms of microbial influence in food allergy

There are many ways in which gut microbiota may protect against the development of food allergy:

Specific bacterial species

Particular species have been highlighted as potentially protective against or associated with allergic disease, such as enterobacteria being associated with eczema and lactobacilli and bifidobacteria being associated with healthy clinical phenotypes.[83;84] However, when trying to replicate such culture-dependent findings across three European infant cohorts, no substantial association was found between specific bacteria and the later development of eczema and food sensitisation.[93]

Species diversity and abundance

Diversity may be defined as the number of species present within the gut microbiome, whereas abundance represents the total number of any particular bacteria. Studies suggest that the gut biome diversity, rather than the presence of single species, may protect against development of food allergy. For instance, culture-independent gel-based techniques have shown that a significant lack of species diversity predisposes to the development of eczema and atopy.[85;86] Unfortunately, these studies did not comment on abundance of any intestinal bacteria, as they were designed to assess relative proportions of species rather than the absolute quantity of bacteria. It is therefore currently unclear whether individuals with a greater variety of species also have a greater abundance of microbiota, protecting them from allergic disease.

Furthermore, gut microbiota vary over time, in accordance with diet and life events.[94] This raises the possibility that rapid turnover of gut species may be associated with less allergic disease. Certainly, greater variety of gut species and more rapid species turnover has been described in infants from less developed countries, perhaps influenced by less hygienic life-style, which may modulate long-term health prospects.[95] The microbiological techniques and sample sizes in the studies published to date are not sufficient to assess whether gut bacterial turnover promotes food tolerance.

Functional capabilities of gut microbiota

Functional capability of gut microbiota may influence food allergy outcomes. The gut microbiota perform a number of biological functions, such as fermentation of food, to allow maximal nutritional extraction and production of short-chain fatty acids, which reduces local mucosal inflammation.[82] Initial ingestion of food prompts sustained alterations in gut microbiota

characteristics, with elevated faecal short chain fatty acid levels, increased carbohydrate metabolism, vitamin biosynthesis and xenobiotic degradation.[94] Faecal samples from infants with cow's milk protein allergy have higher levels of butyric acid and branched short-chain fatty acids than healthy controls, associated with different microbiota profiles when assessed by species-specific oligonucleotide probes.[96] This suggests the function of microbiota may also exert a tolerogenic influence.

1.5.2 STUDY HYPOTHESES AND OUTCOMES:

Main hypotheses

- Children with more diverse gut bacterial species and higher bacterial load have a lower risk of food allergy at 1 year of age, as defined by positive food challenge
- Children with a more diverse gut bacterial species and higher bacterial load have a lower risk of food sensitisation at 3 months of age, as defined by any skin prick test positivity or specific IgE (sIgE) to food >0.1 IU/ml

In addition, the relationship between both indirect and direct measures of microbial exposure and the risk of food sensitisation and allergy at 1 year will be assessed:

Indirect measures

Mode of delivery, number of siblings, personal hygiene practice (frequency of washing, and the use of baby wipes, soap and detergents), early day care attendance, pet ownership (during pregnancy and the first year of the child's life), ante- and postnatal antibiotic exposure as well as childhood vaccinations.

Direct measures

Episodes of respiratory (URTI/LRTIs), gastrointestinal infections and other acute illnesses during the first year of life are related to the risk of food sensitisation and allergy at 1 year. Pathogen serology, such as RSV, measles, enterovirus, rotavirus and chicken pox.

Primary outcome: Any challenge proven food allergy at 1 year of age
Secondary outcomes: Sensitisation to any of the EAT Study foods at 1 year of age

Finally, we will examine the potential impact of indirect and direct measures of microbial exposures on the gut microflora (species diversity and bacterial load). Should we find that certain environmental exposures influence the composition of the gut microbiome, we will additionally examine for any interactions between environmental measures and gut flora on food allergy risk at 1 year of age.

The bowel flora work would be conducted in collaboration with the Department of Molecular Microbiology at KCL (led by Ken Bruce, Geraint Rogers and Damian Rivett), which has extensive experience in doing microflora work and DNA extraction from stool samples).
The EAT TSC includes individuals with experience in the statistical analysis of microbiome data.

1.5.3 SAMPLE SELECTION

Cross sectional component of proposed work

Identification of food allergy-specific gut microbiota though a cross-sectional comparison of faecal bacteria in children at 1 year of age who are food sensitized and/or have eczema present at the one year visit.

It is estimated that 500 already enrolled 1 year-old children will attend for their one year visit from 1st October. All those who are sensitised to one or more of the intervention foods and/or have active eczema will be invited to give a stool sample.

Non-sensitised, non-eczematous controls will be selected from the infants prospectively recruited after the 1st October.

Longitudinal component of proposed work
Identification of the emergence of food-allergy associated gut microbiota characteristics using a case-control longitudinal follow-up of 3-month enrolled babies.

From October, the remaining 300 3-month babies will be consented for the collection of three faecal samples. Both early and standard introduction infants will be asked for a faecal sample at 3 months, 5 months and 1 year of age. The latter two samples coincide with completion of food diaries.

Likely group numbers that will be available:
- 3-month food-sensitised (skin prick positive) in early eating group – 8% of 150 = 12
- 3-month eczematous, non-sensitised group – approximately 20% of 300 = 60
- 3-month non-sensitised non-eczematous group – approximately 200

To maximise the power of this study design, it is important to ensure that good quality faecal samples are collected from all sensitised infants. This group may be supplemented by identifying further children in the SIG who have sIgE > 0.1 IU/ml from 3-month serum samples.

This case-control model will allow comparison of :-

- 3-month, 5-month and 1-year old infants' gut microbiota who are food-sensitised either by SPT or sIgE>0.1 IU/ml (cases) versus non-sensitised eczema (control A) versus healthy non-sensitised controls (control B) to characterise microbiota of emerging food allergy.

- 150 samples from 5-month infants' gut microbiota characteristics who are in the early eating intervention arm versus 150 samples from exclusively breastfed arm, to identify the effect of early food introduction on the gut microbiota

1.5.4 STOOL COLLECTION

After taking the families' consent for EAT participation and faecal sample collection, study staff will collect faecal samples from a nappy parents will be asked to bring with them to their 3 month recruitment visit. EAT staff will use this opportunity to show parents the ideal technique for sample collection and then dispose of the nappy. One sample will be collected using the same technique and equipment used by parents for collecting later samples, and will be used for DNA extraction of bacterial gut constituents. A further sample will be collected by staff, for extraction of bacterial RNA. This second sample will only be collected by study staff from dirty nappies which are brought to the unit, because RNA is less stable than DNA and requires faster processing. The two samples will identify which gut bacteria are metabolically active at the time of sample processing. By comparing bacterial DNA and RNA, inferences may be drawn regarding whether DNA identified bacteria are living shortly after defaecation, and also to gain insight into which species are most active and whether these correlate to allergy associated microbiota.

A further sample will be collected by families at home when their infant is 5 months of age. In order for this to take place families will leave their enrolment visit with one unique-number-identified faecal collection pack (or two if they did not bring a dirty nappy to the unit). Each pack will contain the faecal collection kit, with a pre-stamped Jiffy Bag and sample container, suitable for Royal Mail pillar-box postage. The kit will comprise gloves, sealable bags, 300 microgram mini-scoop and sterile universal tube already labelled with the anonymised identification number and specific weight of tube and scoop.

At 5-months of age, parents will be prompted to collect a morning dirty nappy from their child. They will collect the faecal sample using the described technique and drop the mini-scoop into the collection tube. They will write the date of collection on the label on the collection tube, under their unique number identifier. They will put the collection sample in its Royal Mail packaging and drop the package into their nearest pillar-box at their earliest convenience, which will bring the sample to the King's College London microbiology laboratories at Waterloo. To facilitate the collection of stool samples in the home setting, an instructional video will be posted on the EAT study website to demonstrate safe faecal collection technique using a prepared collection kit.

Families already enrolled onto EAT prior to stool collection beginning, will be asked if they are willing to bring a dirty nappy to their 1 year appointment and consent will then be sought prior to sample collection. If no sample is obtained and the child is sensitised and/or has eczema at the one year visit, they will be offered a sample collection pack to take home with them.

1.5.5 SAMPLE PROCESSING

Stool samples intended for DNA extraction will be frozen down to -80ºC on receipt by the laboratory staff. On a weekly basis, samples for DNA extraction will be thawed, have their DNA extracted using standardized laboratory procedures and the DNA amplified using standardized PCR protocol (Rogers *et al.*, 2005; Green *et al.*, 2006; Ellis *et al.*, submitted). After a suitable batch size of extracted DNA has been assembled, the extracted DNA samples will undergo amplification and pyro-sequencing. The sequencing data output will be jointly analysed by Bio-informatician and Microbiology teams.

At Waterloo, King's College London Laboratories, quantification of overall bacterial load in Colony Forming Unit equivalents per gram of stool will be determined for each sample. Terminal restriction fragment length polymorphism will be used in the initial phases (Rogers *et al.*, 2009) to assess the variety and abundance of bacterial species in the samples. This work will validate collection and processing methodologies e.g. the longest time-frame for which faecal samples may rest at room temperature without quantitative or qualitative impact on microbial signal.

Samples intended for RNA extraction will only be collected directly from parents by EAT study staff and will undergo RNA extraction immediately on arrival to the laboratory. The sub-types of RNA will be quantified and comparisons drawn with bacterial data derived from DNA.

1.6 KNOWN AND POTENTIAL RISKS

1.6.1 EARLY INTRODUCTION GROUP

Potential risks associated with the consumption of the allergenic foods from three months of age are worsening of eczema, weight gain, nutritional compromise, metabolic abnormalities, and an increased risk of allergy to the allergens.

Four different studies show that the median age of reacting to peanuts is between 14 and 24 months of age. The vast majority of patients react upon first known dietary exposure to peanut.[97-99] This argues strongly against the possibility that peanut allergy is caused by eating peanuts and provides some reassurance that we will not induce peanut allergy by feeding patients peanuts.

Both early (under four months) and late (after six months) introduction of wheat has been reported to be associated with coeliac disease.[100] Wheat will therefore not be introduced before four months of age.

1.6.2 STANDARD INTRODUCTION GROUP

The standard introduction group will be following current UK Government infant feeding guidelines. In the standard introduction group, avoidance of the allergenic foods may result in an increased risk of allergy to the allergens.

1.6.3 BOTH GROUPS

Undergoing laboratory assessments may involve a low risk of haemorrhage, haematoma, and infection at the venipuncture site. Risks associated with the food challenges include nausea, vomiting, itching, urticaria, angioedema, asthma, other respiratory symptoms, and anaphylaxis.

2. SCIENTIFIC OBJECTIVES

2.1 OBJECTIVE ONE

Hypothesis

The early introduction of allergenic foods (from three months of age) will induce regulatory mechanisms that result in a reduced level of food allergy by three years of age. The effect on food sensitization at three years of age will be determined.

Objectives

01a) To set up a randomized controlled intervention trial to assess the impact on food allergy through the early introduction of allergenic foods from three months of age. To randomize the cohort into two groups, with exclusive breastfeeding for six months in the standard introduction group and breastfeeding to around six months combined with the sequential introduction of allergenic foods from three months in the intervention group.

01b) To complete the antenatal recruitment of 3000 mothers, with 2500 infants available for randomization at three months of age (1250 infants to be randomized into the standard introduction group and 1250 infants to be randomized into the intervention group) with complete assessment of maternal nutrition during pregnancy and throughout breastfeeding.

Contract variation September 2010

01b) To complete the recruitment of 1302 mothers, with 1302 infants available for randomization at three months of age (651 infants to be randomized into the standard introduction group and 651 infants to be randomized into the intervention group) with retrospective assessment of maternal nutrition during pregnancy and throughout the first three months of breastfeeding.

01c) To follow-up the whole cohort until the primary assessment point at three years of age.

Contract variation September 2010

01c) To follow-up the whole cohort (final cohort size of 1106 required - 553 infants in each arm) until the primary assessment point at three years of age.

01d) To measure the primary outcome of the period prevalence of IgE mediated food allergy to the six intervention foods between one and three years of age in both arms (Section 3.3).

2.2 OBJECTIVE TWO

Hypothesis

The early introduction (from three months of age) of allergenic foods into the infant's diet may lead to a reduction in the prevalence of other allergic conditions by three years of age: specifically asthma (including atopic wheeze), eczema, allergic rhinitis (including aero-allergen sensitization) combined food allergy prevalence (including food sensitization) and the prevalence of combined allergic disease.

Objectives

02a) To assess the prevalence of the secondary end points - asthma (including atopic wheeze), eczema, allergic rhinitis (including aero-allergen sensitization), combined food allergy prevalence (including food sensitization) and the prevalence of combined allergic disease with breastfeeding duration.

02b) To assess the individual prevalence of the secondary end points - asthma, eczema and allergic rhinitis at repeated time points during the three year follow up amongst the intervention group compared with the standard introduction children. Allergic symptoms will be assessed monthly until one year of age and three monthly until three years of age.

02c) To measure the cumulative prevalence at three years of age of any allergic condition: asthma (including atopic wheeze), eczema, allergic rhinitis (including aero-allergen sensitization), combined food allergy prevalence (including food sensitization) in the intervention group compared with the standard introduction group.

02d) To measure the prevalence of allergic conditions by three years of age by both subjective (parent completed questionnaires of reported symptoms, doctor diagnoses of conditions) and objective (skin prick testing & specific IgE) measures.

02e) To collect and store blood from both the standard introduction and early introduction group at birth, three months, one year and three years of age. To extract DNA from cord blood to screen for defects in the currently known skin barrier genes as well as to undertake immunological analyses. The blood samples collected will be used to investigate the immunological evolution of food allergy (and other allergies). Where no cord blood was taken, samples for DNA analysis will be taken with the non-invasive Oragene® DNA self-collection kits from buccal mucosa.

2.3 OBJECTIVE THREE

Hypothesis

The early introduction of antigenic foods does not have any deleterious effects.

Objectives

03a) To assess the growth and nutritional status of children in the early introduction group compared with the standard introduction group.

03b) To ensure the rapid assessment of any participant with a suspected food allergic reaction.

3. Study Design

3.1 OVERALL STUDY DESIGN

The study design with regard to ascertaining the principal outcome – IgE mediated food allergy, is summarized in Figure 5.

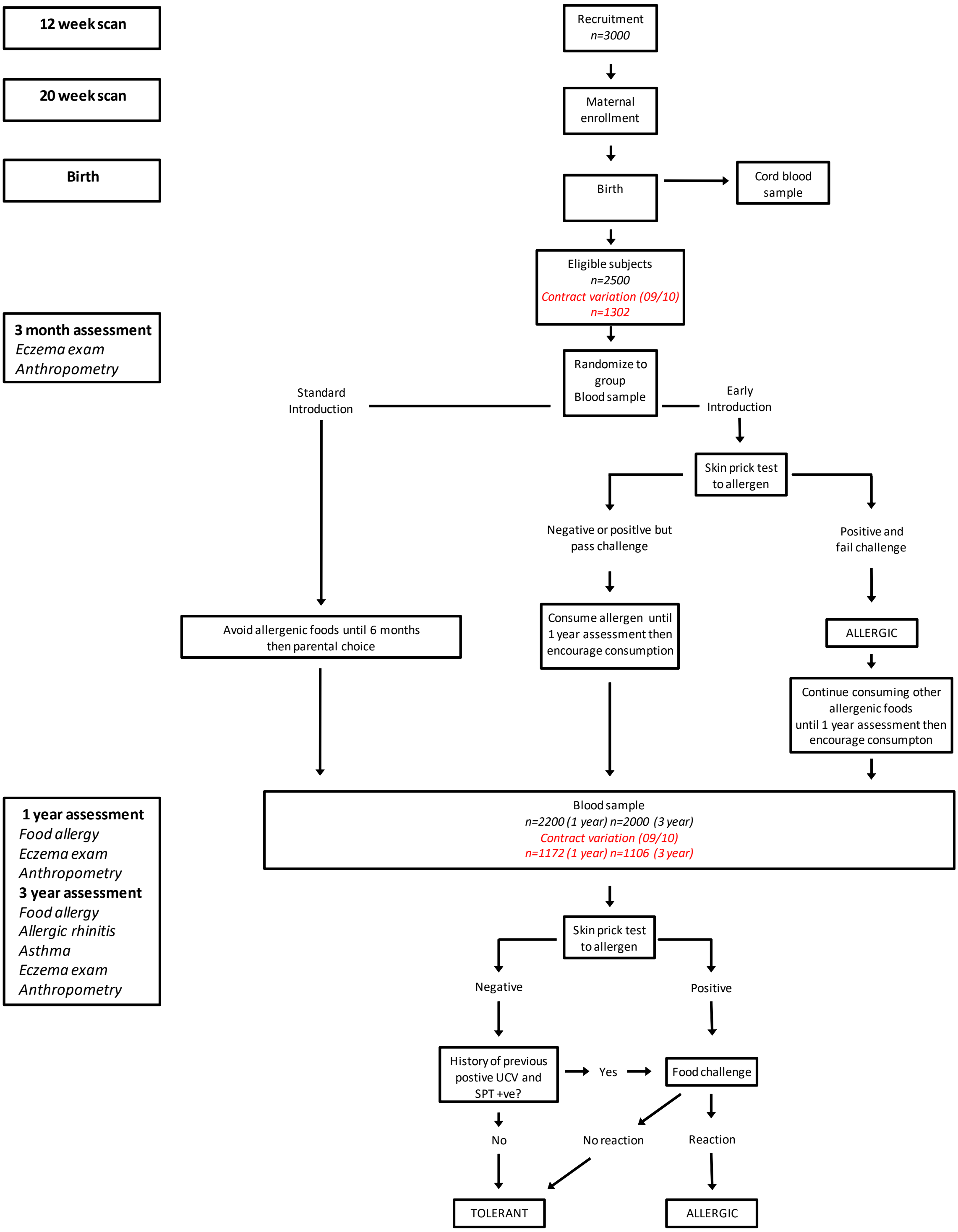

FIGURE 5. OVERALL STUDY DESIGN

An overview of the study and the timing of interim assessments are given in the Study Overview Chart (Appendix 1). The timescale for the project is graphically represented in the Study Gantt Chart (Appendix 2).

3.1.1 EAT STUDY POPULATION

3.1.1.1 ANTENATAL RECRUITMENT

Study antenatal recruitment will start from two large London antenatal units (Kingston Hospital and St Thomas' Hospital) participating in the study. Both have over 5000 deliveries a year, thus requiring between 10 and 15% of mothers to participate. Expectant mothers will be approached at their 12 week scan. Information about the study will be provided in the two centres by a full time research assistant. Mothers will then be formally consented and enrolled onto the study at their 20 week scan.

Recruitment is designed to be non-linear, being slower at the onset of the study and accelerating as the study progresses. Thus the actual recruitment time scale that we have used to set the milestones is:

Start of antenatal recruitment	Time	Rate (#/month)
Antenatal recruitment of 500 mothers completed	+ 8 months	62
Antenatal recruitment of 1000 mothers completed	+ 12 months	125
Antenatal recruitment of 1500 mothers completed	+ 15 months	167
Antenatal recruitment of 2000 mothers completed	+ 18 months	167
Antenatal recruitment of 3000 mothers completed	+ 24 months	167

3.1.1.2 BACKUP RECRUITMENT STRATEGIES

A number of measures have been taken to try and ensure satisfactory recruitment and retention of participants. Measures to minimize loss to follow-up will include: a central telephone number and contact details of grand-parents who our previous studies have shown are much less likely to move. Additionally a high proportion of subjects are likely to have e-mail addresses which we will also record. The ease of access to a dedicated team incorporating dietitians, nurses, paediatricians and a dermatologist via a dedicated study direct line will also be a significant incentive to stay in the project. The majority of children have no access to tertiary paediatric allergy or dietetic services and participation in the study will enable parents to access these scarce resources.

The recruitment rate will be assessed on a monthly basis. If the required recruitment rate is not met for three consecutive months a decision would be taken by the trial steering committee to seek to boost recruitment by using one or both of the following mechanisms:

Additional recruitment centres
The study could utilise one or two additional recruitment centres. These would be chosen from amongst the following centres: Queen Charlotte's Hospital, Chelsea & Westminster Hospital, Royal Free Hospital and King's College Hospital.

Bounty scheme
The Bounty scheme runs the largest Parenting Club in the United Kingdom. This will entail Bounty mailing out an information leaflet about the study with our contact details to mothers with infants aged five and ten weeks in England and Wales in areas with high breastfeeding rates utilising the Bounty register. Mothers, having read the information leaflet, would initially contact our study team research recruiters who would screen respondents to ensure they were eligible to participate in the study and initiate the process of obtaining informed consent for the mothers' participation in the study.

3.1.1.3 BOUNTY RECRUITMENT (CONTRACT VARIATION SEPTEMBER 2010)

Antenatal recruitment commenced on the 4th February 2009 and continued at both recruitment centres (St Thomas' Hospital and Kingston Hospital) until 15th April 2009 when recruitment at St Thomas' Hospital was suspended after seven weeks due to a staff resignation for personal reasons and was not replaced due to insufficient interest among women who had been approached previously. It was already known that the demographic was less affluent at St Thomas' but it had been anticipated that the number of deliveries performed at the hospital (one of the largest delivery units in London) would mitigate for the less favourable demographics. However, there was a great reluctance amongst non-Caucasian mothers to entertain participating. Antenatal recruitment continued in the sole recruiting centre (Kingston Hospital) until 5th September 2009 when it ceased following the resignation of the remaining recruiter. The two resignations in conjunction with insufficient recruitment resulted in a decision, with agreement of the FSA, to switch to postnatal recruitment using the Bounty Parenting Club to achieve higher enrolment levels.

A pilot study of postnatal recruitment commenced on Monday 5th October 2009 via the direct mailing of approx 12,000 families with infants between five and ten weeks of age. This pilot study assessed the result of three monthly mail outs in October, November and December 2009. The result of the pilot study was reviewed by the EAT TSC and the FSA on 22nd February 2010.

At this meeting the following observations were made:

- A higher risk cohort appears to be being recruited onto the study than originally anticipated, with over 30% of infants having visible eczema at three month enrolment. This would suggest that the rate of food allergy in the cohort at three years of age is likely to be much higher than the 6% originally predicted (upon which power calculations were based). The data from Hill's EPAAC Study Group, as well as other relevant published data, were discussed at length to try and extrapolate what food allergy prevalence might be seen in the EAT study standard introduction group at three years of age.

- Drop out on the study to date has been less than 1%. The study team had factored in a 20% drop out, as it was considered that many mothers would not be able to comply with the intervention, which does not appear to be the case. A revised dropout rate of 15% for sample size calculation purposes was agreed

- When the EAT study was originally set up, the calculated recruitment figurers were based on 90% power. However, the FSA and TSC statistician suggested that 80% power is the standard figure that is used in statistical analysis and would be acceptable for this study. It was therefore agreed by the FSA and the study team that 80% instead of 90% would be used to calculate the number of infants required in the final cohort size to meet the study objectives.

Conclusion

It was agreed by both the study team and FSA that a rate of 8% food allergy prevalence, 80% power and a 15% drop out rate would be used to calculate the number of infants required for the final cohort size. Using these figures, it was calculated that 1302 infants would need to be recruited onto the study (651 infants in each arm) to ensure the necessary power was obtained. This would yield a final cohort size of 1106 infants (553 infants in each arm) after drop out.

Extrapolating the recruitment ratio achieved in October 2009 in the pilot mail out period of 1.36 recruits/1000 mail and assuming 37,500 leaflets were sent out each month, this would result in 51 infants being recruited per month. This would yield a final cohort size of 1180 infants within the original duration of planned study recruitment. The study team would still require an additional 122 infants, to be recruited onto the study (1302 – 1180). It was therefore agreed to extend the length of recruitment for four months. This contract variation was formally agreed between the EAT study team and the principal funders in September 2010.

3.1.2 MATERNAL PARTICIPATION

Maternal diet will be assessed using a Food Frequency Questionnaire (FFQ) based on Carina Venter's FFQ validated[57] for milk, wheat, peanut and fish and Kate Grimshaw's FFQ validated (personal communication) for milk and egg.

In addition, to allow comparison with results from the LEAP Study, the questions used to assess maternal ingestion of peanut in LEAP will also be administered.

This will be completed by the mother at the 20 week ultrasound scan, at 34 weeks gestation and when the mother attends the three month assessment visit.

Mothers recruited postnatally will complete the same diet questionnaire to assess both pregnancy and lactation consumption of the principle allergenic foods.

3.1.2.1 MATERNAL ANTENATAL AND LACTATION DIETARY ADVICE RELATING TO ALLERGEN FOOD CONSUMPTION:

As stated in Section 1.3.3, in 1998 COT issued advice with regard to peanut consumption for pregnant women whose unborn child was regarded as being at high risk of atopy. These were women with a history of atopy themselves, or in the father of the baby or in any sibling of the baby. Women recruited prior to the 25th August 2009 who fulfilled these criteria were advised to follow the recommendations relating to pregnancy and lactation:

- Pregnant women who are atopic, or for whom the father or any sibling of the unborn child has an atopic disease, may wish to avoid eating peanuts and peanut products during pregnancy
- Breastfeeding mothers who are atopic, or those for whom the father or any sibling of the baby has an atopic disease, may wish to avoid eating peanuts and peanut products during lactation

Following the revision of the guidelines issued on the 25th August 2009 women participating in the study at that point or who subsequently enrol will be advised that there is no longer any restriction on the consumption of peanuts during pregnancy and lactation as part of a healthy balanced diet, unless they themselves are allergic to peanuts.

3.1.2.2 PARENTAL ALLERGIC SENSITIZATION

We will also seek to undertake skin prick testing and obtain a sample of blood from both biological parents when they attend the three year assessment clinic with their child. The blood sample will be processed and stored for subsequent specific IgE measurement.

3.1.2.3 CORD BLOOD COLLECTION

At antenatal recruitment, mothers will be given the appropriate containers to allow the collection of cord blood at their delivery. The midwife delivering the baby will be asked to collect blood. This will be stored in a fridge prior to collection by the recruitment research assistants in the hospital. Samples will be transferred to the study laboratory and serum will also be collected and stored for future sero-epidemiological work.

The FSA will have ownership of the samples and will licence this out to Kings, for use in a project funded by a third party, subject to FSA approval. The eligibility of any third party and decisions on how to use the samples will be taken by the Steering Committee, of which the FSA is a member.

3.2 INFANT ENROLMENT - SCREENING FOR ALLERGY AT THREE MONTHS

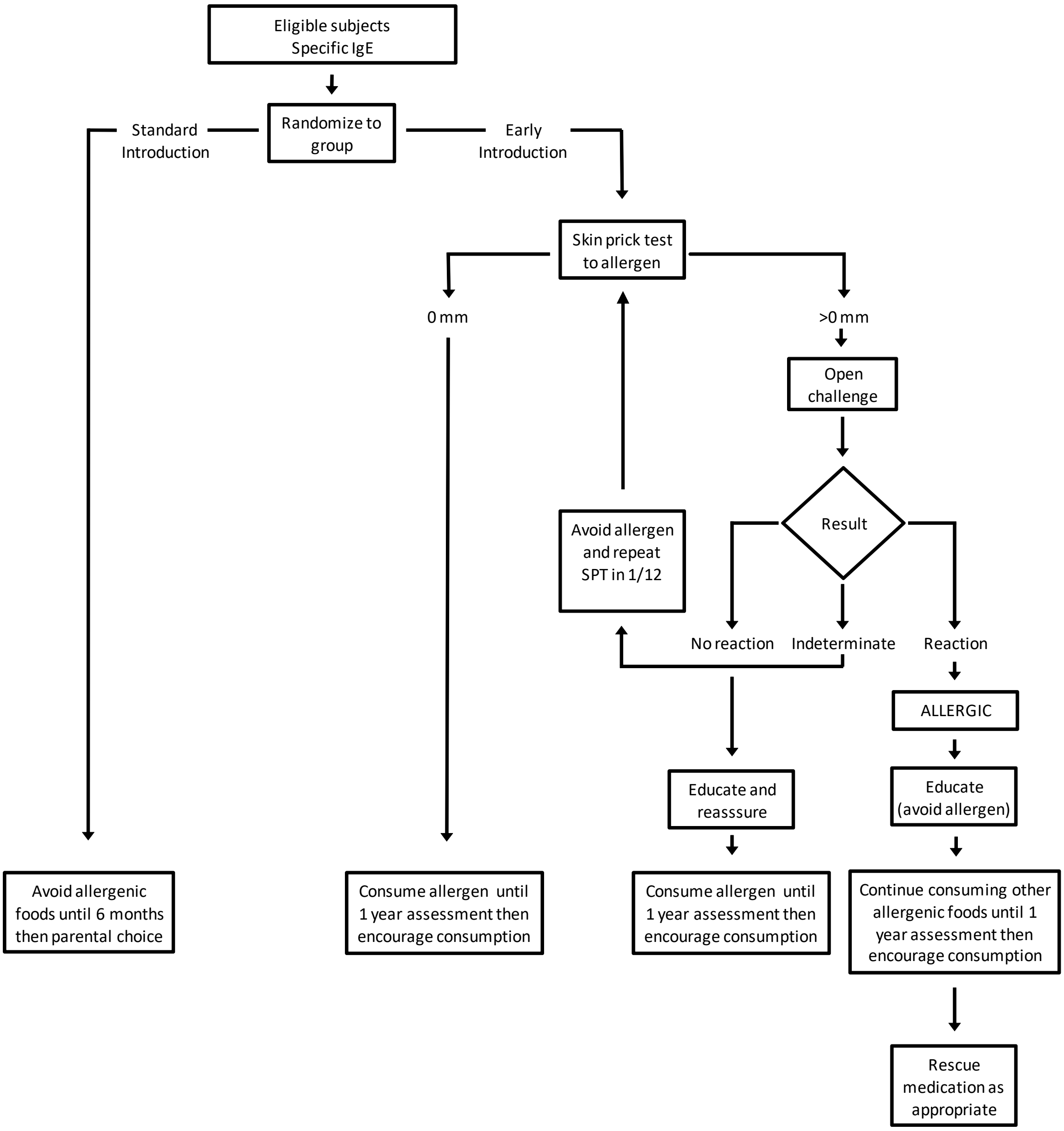

FIGURE 6. THREE MONTH SCREENING VISIT

Infants in the early introduction group will undergo screening for pre-existing food allergies when they attend the three month visit. These infants will undergo skin prick testing in duplicate with the following allergens: raw egg white, whole cow's milk, sesame paste (tahini), fish (cod), wheat, peanut and positive (histamine 10 mg/ml) and negative control (50% glycerol, 50% buffered saline) solutions.

For screening purposes a positive skin prick test will be defined as >0 mm for any allergen. Using a low threshold and whole foods will minimize the risk of false negative results. When screen positive early introduction infants return for their food challenge they will have their skin prick testing repeated with the same allergens as above, but also for commercial egg, sesame and milk solutions where relevant in order to establish the predictive value in this young age group of whole food and commercial skin prick allergens.

3.3 PRIMARY ENDPOINT

The period prevalence of IgE mediated food allergy to the six intervention foods between one and three years of age in both arms.

3.3.1 DEFINITION OF IGE MEDIATED FOOD ALLERGY FOR PRIMARY ENDPOINT

1. A positive DBPCFC at one year or three years of age (as per the algorithm in Figure 9) in a child sensitized to one of the six intervention foods.
2. A positive DBPCFC between one year and three years in a child attending an unscheduled clinic visit (as per the algorithm in Figure 7) in a child sensitized to one of the six intervention foods.

Whilst the first two categories relate to events between one and three years of age, we will include children potentially outside of this range in two exceptional circumstances:

3. A positive challenge (open or DBPCFC) at between six months and one year of age that occurs in a child who is sensitized to one of the six intervention foods who subsequently refuses a DBPCFC at one year and three years of age

   Rationale: Below six months only intervention children have challenges so this category is restricted to those infants who are six months old or more.

4. A food allergic history in a child with a SPT ≥5 mm (as per the algorithm in Figure 7)

   Rationale: There will be a small number of children who have an immediate type allergic reaction and are significantly sensitized whose parents refuse to allow them to undergo any further challenge.

When the final outcomes are published, the relative contributions of children to the final outcome in each of these four categories will be presented separately as well as the overall cumulative figure to allow reviewers to independently assess the data.

3.4 SECONDARY ENDPOINTS

Period (one to three years of age) prevalence food outcomes

The period prevalence of all IgE mediated food allergy between one and three years of age in both arms.

The period prevalence of all food allergy (IgE and non-IgE mediated) between one and three years of age in both arms.

The period prevalence of sensitization (defined in Section 3.9) to food between one and three years of age in both arms.

The total number of foods (of the six intervention foods) to which IgE mediated food allergy has been diagnosed between one and three years of age in both arms amongst children with IgE mediated allergy to one or more of the six intervention foods.

The total number of foods (any foods) to which IgE mediated food allergy has been diagnosed between one and three years of age in both arms amongst children with IgE mediated allergy to one or more foods.

Cumulative (by three years of age) prevalence food outcomes

The cumulative prevalence of IgE mediated food allergy to the six intervention foods by three years of age.

The cumulative prevalence of all IgE mediated food allergy by three years of age.

The cumulative prevalence of all food allergy (IgE and non-IgE mediated) by three years of age.

The cumulative prevalence of non-IgE mediated food allergy by three years of age.

The cumulative prevalence of sensitization (defined in Section 3.9) to the six foods by three years of age.

The total number of foods (of the six intervention foods) to which IgE mediated food allergy has been diagnosed by three years of age amongst children with IgE mediated allergy to one or more of the six intervention foods.

The total number of foods (any foods) to which IgE mediated food allergy has been diagnosed by three years of age amongst children with IgE mediated allergy to one or more foods.

Other allergic disease outcomes

The point prevalence of eczema at one year and three years of age and cumulative prevalence of eczema by three years of age.

The modified International Study of Asthma and Allergies in Childhood (ISAAC) eczema questionnaire will be used as a screening tool throughout the study. In addition, all children will be physically examined for eczema at the three assessment points (three months, 1 year and three years), using a photographic protocol based on the UK diagnostic criteria for eczema.[101]

The severity of eczema at one year and three years of age.

The severity of eczema at three months, one year and three years of age will be assessed by modified SCORing Atopic Dermatitis score (SCORAD)[102] *and Patient-Oriented Eczema Measure (POEM).*[103]

The prevalence of allergic rhinitis at three years of age.

The prevalence of inhalant allergen sensitization at one year and at three years of age by skin prick test.

Based on positive skin prick reactions to one or more of the aero-allergens (house dust mite, cat, dog, six grass mix and three tree mix).

The prevalence of inhalant allergen sensitization at one year and at three years of age by specific IgE measurement.

Based on a positive specific IgE result to a panel of aeroallergens at one or three years of age.

The prevalence of the atopic wheeze phenotype at three years of age.

Composite allergy outcome

The prevalence of combined allergic disease (a composite of cumulative IgE mediated food allergy to all foods, atopic wheeze phenotype, eczema and allergic rhinitis) at three years of age.

The prevalence of combined allergic disease (a composite of cumulative IgE and non-IgE mediated food allergy to all foods, atopic wheeze phenotype, eczema and allergic rhinitis) at three years of age.

Safety outcome

Incidence of adverse events and laboratory abnormalities; nutritional evaluations.

Immunological outcomes

Results of cellular and humoral assessments of immune response related to the development of allergy or tolerance to specific allergens (*subject to additional funding*).

Microbiome outcomes

The association between the bowel and skin microbiomes and the development of food allergy and other atopic disease.

Genetic analyses

The association between skin barrier gene defects (such as carriage of the filaggrin skin barrier mutations) and other measures of skin barrier integrity (transepidermal water loss) with all the study outcomes will be assessed.

3.5 DETERMINATION OF FOOD ALLERGY – UNSCHEDULED CLINIC VISITS

We will both actively seek (using the interim questionnaires) and passively obtain (by parental report to a dedicated project telephone line) reports of possible food allergic reactions or persistent refusal and aversion. Parents will be encouraged to complete interim questionnaires online which will facilitate the rapid identification of positive respondents. Hard copies will be available and posted out to those without internet access. If the reactions are felt to be suggestive of food allergy, the parent will be advised to avoid that particular food and the children will attend the paediatric allergy clinical trials unit to undertake an unscheduled clinic visit and follow the algorithms outlined in Figure 7 for suspected IgE mediated reactions and Figure 8 for suspected non-IgE mediated reactions.

Skin prick tests will be undertaken (using a commercial skin prick solution) to determine an individual child's sensitization status.

Open challenges will be undertaken in those under one year of age and double blind placebo controlled challenges in those one year of age or older.

5mm skin prick threshold at UCV for IgE mediated allergy

Children who attend an UCV with a clear history of a clinical allergic reaction and who are found to have a skin prick response (using a commercial skin prick solution) to that food of 5mm or more will be deemed allergic and not undergo a challenge at that UCV (Figure 7). A minimum time interval will be allowed to elapse before consideration of a further challenge is undertaken (Section 6.6.4). Further challenges will be undertaken in accordance with Tables 15 and 16. The underlying principal being that some children will subsequently outgrow their allergy (especially for milk and egg) and this needs to be assessed.

The 5mm threshold is based on the limited data that exists for positive predictive values (PPVs) for young children. Based on 555 open food challenges (339 challenges to cow milk, 121 to egg, and 95 to peanut) in 467 children (median age for cow's milk challenge 31.0 months, for egg 36.2 months, and for peanut 57.8 months) SPT wheal diameters that were "100% diagnostic" for allergy were defined to cow's milk (≥8 mm), egg (≥7 mm) and peanut (≥8 mm). In children less than two years of age, the corresponding weal diameters were ≥6 mm, ≥5 mm and ≥4 mm, respectively.[104;105] Thus 5mm is the appropriate threshold for our cohort which covers the age range three months to three years. The 5mm threshold will also be used for peanut throughout the EAT age range given data from the LEAP study with infants with 4mm responses in the consumption arm (between 5 and 11 months of age) being able to introduce peanut successfully into their diet.

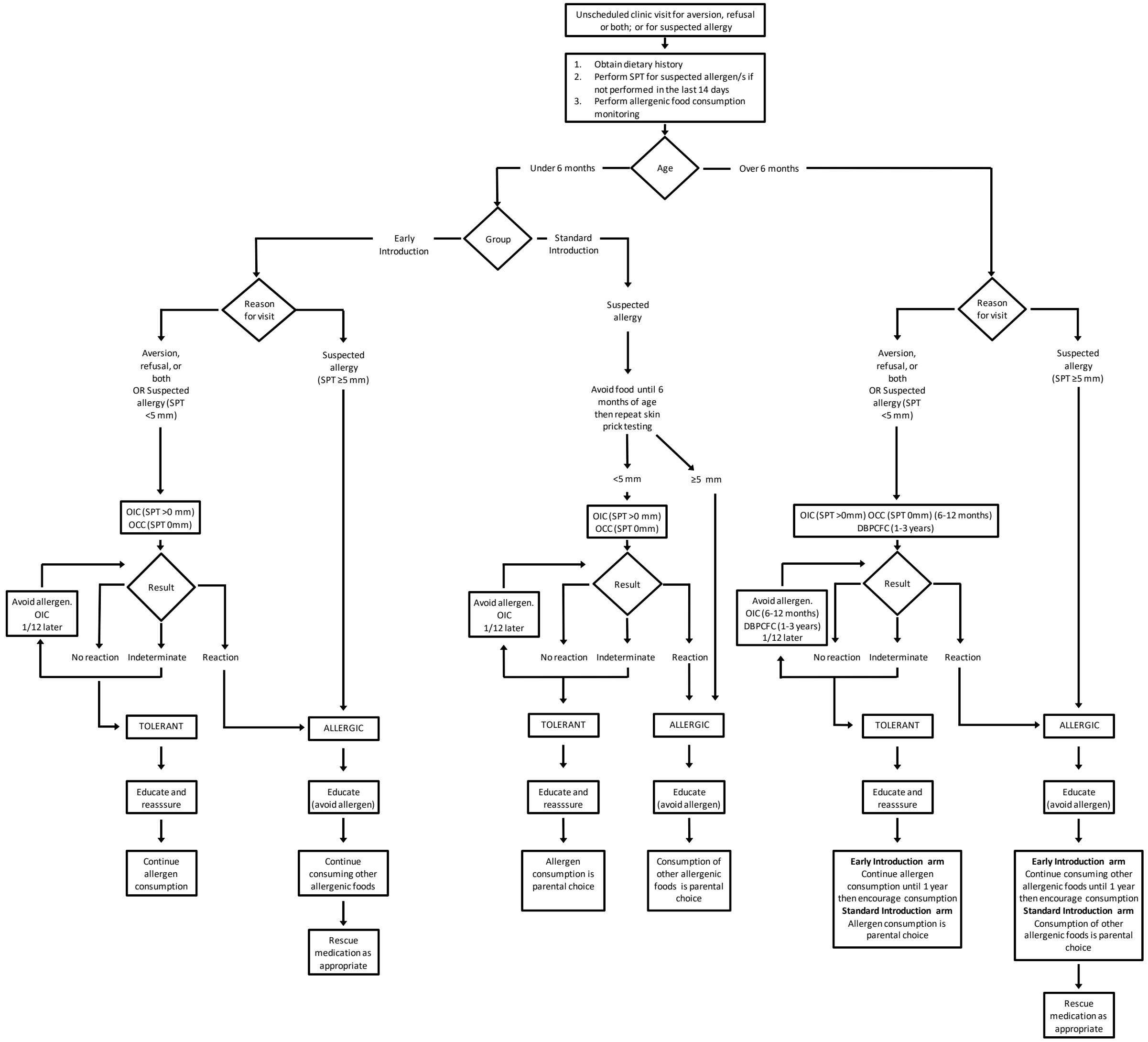

FIGURE 7. ASSESSMENTS TO BE PERFORMED AT UNSCHEDULED CLINIC VISITS- IGE MEDIATED FOOD ALLERGY

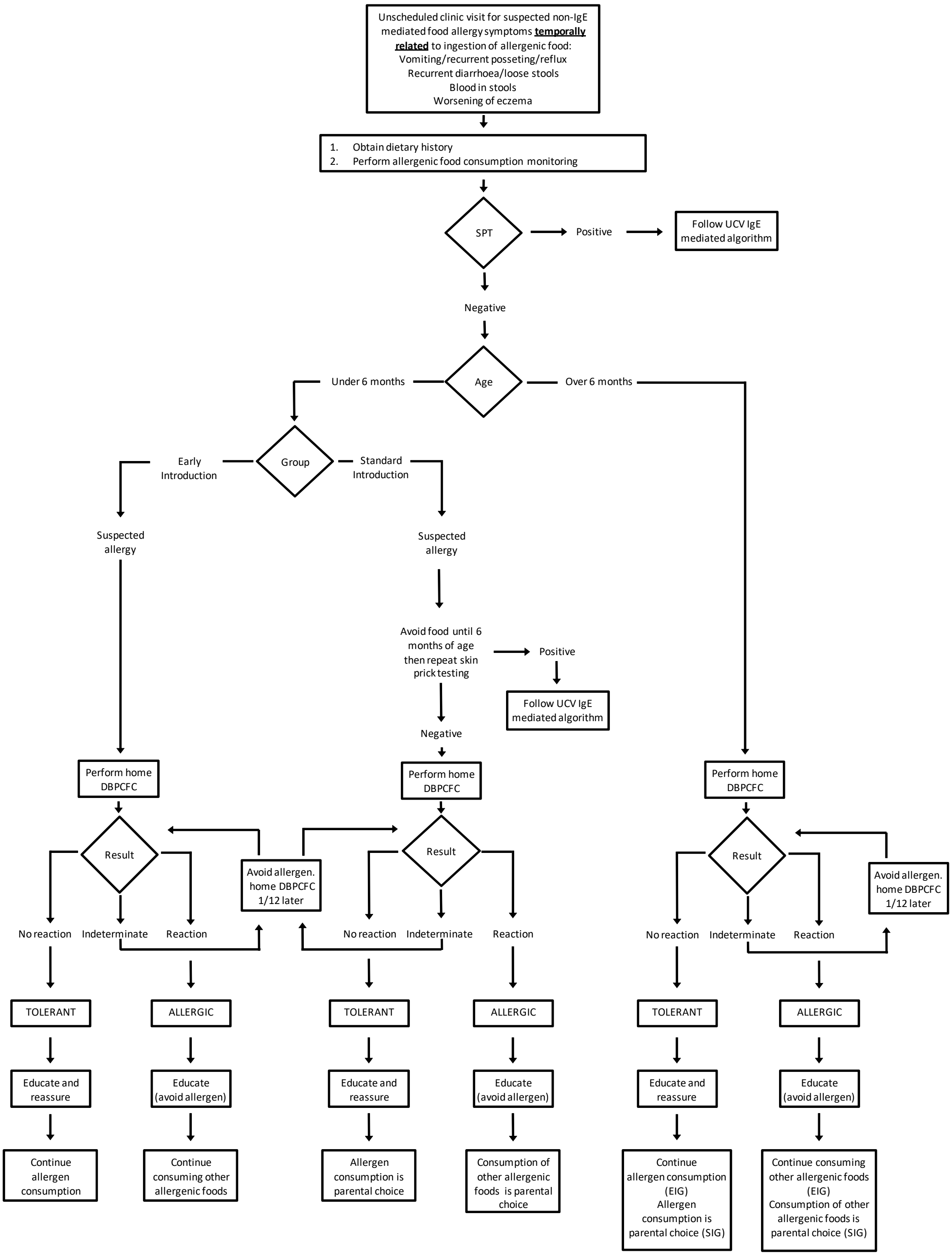

FIGURE 8. ASSESSMENTS TO BE PERFORMED AT UNSCHEDULED CLINIC VISITS- NON-IGE MEDIATED FOOD ALLERGY

3.6 DETERMINATION OF FOOD ALLERGY – ONE YEAR ASSESSMENT

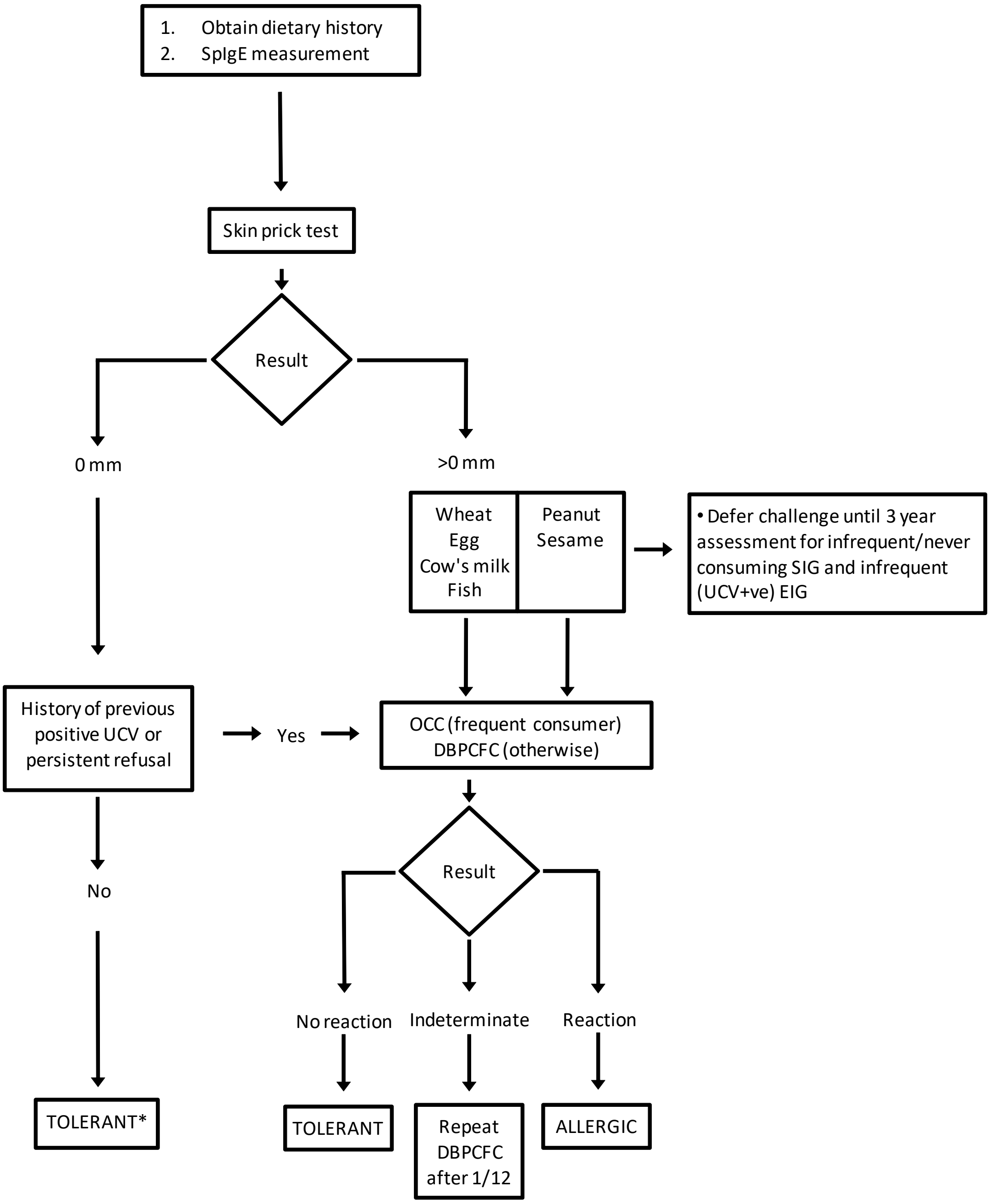

* If has eaten standard EAT portion (≥2 grams in a single portion) in the past.

FIGURE 9. ONE YEAR ASSESSMENT VISIT – IGE MEDIATED FOOD ALLERGY

All infants will be invited to attend an interim one year assessment. It is anticipated that approximately 25% of infants will have visible eczema present at the three month assessment point. Infants with early onset eczema, particularly if moderate or severe in intensity, have a significantly increased risk of food allergies. In the ALSPAC study the proportion of parent reported eczema by six months of age was 42.9%. However, this includes transient and mild eczema. The cohort of 25% of infants with eczema present at three months of age in this study will therefore consist of approximately 325 children. The prevalence of food allergy amongst children such as these reported in the literature varies from 27% to 37%, and we estimate a

prevalence of 30%. It is estimated that with loss to follow up, there will be 300 high risk children amongst those attending the one year assessment.
The study is designed to have sufficient power to detect a protective effect in this high risk sub-population (see Section 9.3).
Food allergy status will be determined using the algorithm in Figure 9. It will be possible to assess whether the intervention is associated with an altered prevalence of eczema and whether the intervention is effective at reducing food allergy in high risk children.

Peanut and sesame sensitization

Children who are found to be sensitized to peanut and sesame at the one year assessment will undergo assessment in accordance with Table 16. The decision to challenge and type of challenge undertaken will be based on group and frequency of consumption status.

Consumption frequency definitions:
Frequent:

1. Consuming at least one EAT portion (≥2 grams of food protein) of the food within the last month.
2. History of ever having consumed more than three EAT portions (≥2 grams of food protein at a time) of the food.

Infrequent/Never:

1. Does not fulfil the above two criteria.

Frequent consumers will undergo an open cumulative challenge. If the challenge result is negative, they will be told to maintain their consumption. For the participants in the early introduction group they will be encouraged to consume peanut and sesame in the recommended quantities. There are several scenarios where mothers will be advised to avoid giving peanut/sesame to their infant until they are reviewed at the three year visit with no challenge being undertaken at the one year visit (Table 16). The reason for deferring the challenges is that there is a theoretical risk that undertaking a sesame or peanut challenge in these infants who have been exposed to little or no sesame or peanut could have a tolerizing effect and induce the same kind of protection that regular peanut and sesame consumption is aiming to achieve in the early introduction group.

3.7 DETERMINATION OF FOOD ALLERGY – THREE YEAR ASSESSMENT

All children not lost to follow up will be invited to attend the final three year assessment. They will be assessed as per the algorithm outlined in Figure 10.

Those children with indeterminate responses to their DBPCFC will undergo a repeat DBPCFC one month later.

3.8 DETERMINATION OF SENSITIZATION

At three months of age sensitization will be determined in a serum sample to the six intervention foods: egg white, milk, sesame, fish, wheat and peanut.

At 12 months of age, serum will be obtained from children to measure sensitization to the six intervention foods and to a panel of aero-allergens including: *D. Pteronyssinus*, *D. Farinae*, cat and dog dander, horse dander, timothy grass, cladosporum, silver birch, olive, mugwort and nettle.

At three years of age all children will undergo the same assessment as that done at 12 months.

As noted in Section 1.3.5, specific IgE levels to the intervention foods will not be measured until the child reaches the end of the study.

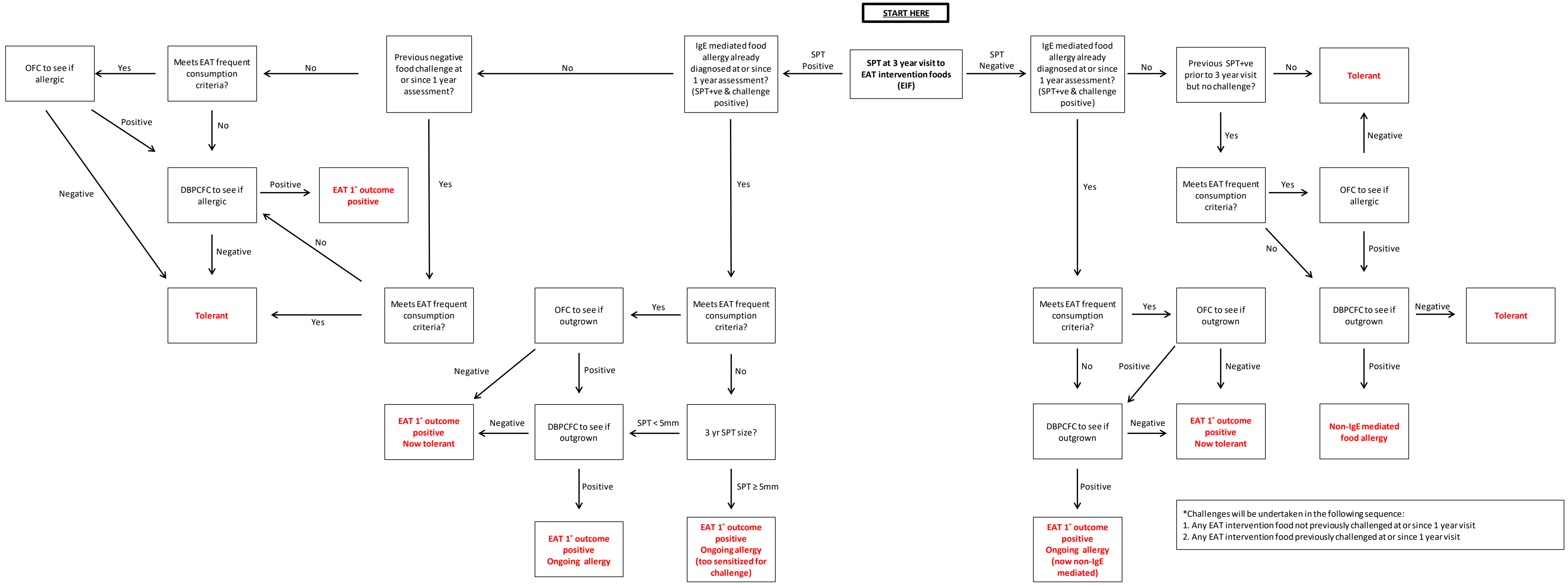

FIGURE 10. THREE YEAR ASSESSMENT VISITS – IGE MEDIATED FOOD ALLERGY

3.9 STUDY DEFINITIONS

3.9.1 ALLERGIC DISEASE DEFINITIONS

Allergic sensitization. Either allergen-specific IgE >0.1 kU/L, as defined by the CAP System™ (Pharmacia Diagnostics AB); or positive SPT, defined as SPT wheal diameter >0 mm with appropriate controls.

Asthma. ISAAC Core Question: Has your child ever had asthma? **Eczema.** A rash on physical examination which fulfils the UK diagnostic criteria-based photographic protocol of the ISAAC Phase Two.

Failure to thrive. Failure to thrive based on Child Growth Foundation thrive charts.

Food allergy. Positive reaction on open challenge (under one year) or DBPCFC (over one year). IgE mediated if skin prick test results is >0 mm. Non-IgE mediated if skin prick test result is negative.

Life-threatening anaphylaxis. An allergic reaction accompanied by any of the following: hypoxia, as evidenced by central cyanosis or oxygen saturation ≤ 89%; hypotension; loss of consciousness; or admission to intensive care.

Perennial rhinitis. Sensitization to a perennial allergen (house dust mite, cat or dog) and ISAAC rhinitis symptoms experienced when exposed to the relevant allergen.

Seasonal rhinitis. Sensitization to a seasonal allergen (grass or tree pollen, moulds) and ISAAC rhinitis symptoms experienced during the relevant season.

Rhinitis. ISAAC Core Questions: Has your child ever had a problem with sneezing, or a runny, or blocked nose when you DID NOT have a cold or the flu? In the past 12 months, has your child had a problem with sneezing, or a runny, or blocked nose when he/she DID NOT have a cold or the flu? Has your child ever had hay fever?

Wheeze. ISAAC Core Question: Has your child ever had wheezing or whistling in the chest". Wheezing in this context means "breathing that makes a high-pitched whistling or squeaking sound from the chest, not the throat".[106]

3.9.2 INFANT FEEDING DEFINITIONS

The term "weaning" will be avoided where possible in the EAT study. Its meaning is widely acknowledged as being ambiguous. The DH leaflet on infant feeding states that: "Weaning means introducing a range of foods gradually until your baby is eating the same foods as the rest of your family."[8] In contrast the Oxford English Dictionary (2nd edition) defines weaning as: "To accustom (a child or young animal) to the loss of its mother's milk; to cause to cease to be suckled." The intention of the EAT study is for the early introduction of allergenic foods to have no adverse impact on duration of breastfeeding. To call the early introduction group the early weaning group could suggest that it is seeking the cessation of breastfeeding if weaning is interpreted in line with the Oxford English Dictionary.

The group following the government's infant feeding recommendations will be termed the "standard introduction group". This also serves to convey the message that the EAT Study is in essence a comparison of two timings of introduction of the allergenic foods: early versus standard. The latter meaning the government's recommendations i.e. not before six months of age.

Infant feeding definitions are taken from the recent WHO report on indicators for assessing infant and young child feeding practices (Table 1).[107]

Table 14. Criteria that define selected infant feeding practices

Feeding practice	Requires that the infant receive	Allows the infant to receive	Does not allow the infant to receive
Exclusive breastfeeding	Breast milk (including milk expressed or from a wet nurse)	ORS, drops, syrups (vitamins, minerals, medicines)	Anything else
Predominant breastfeeding	Breast milk (including milk expressed or from a wet nurse) as the predominant source of nourishment	Certain liquids (water and water-based drinks, fruit juice), ritual fluids and ORS, drops or syrups (vitamins, minerals, medicines)	Anything else (in particular, non-human milk, food-based fluids)
Complementary feeding	Breast milk (including milk expressed or from a wet nurse) and solid or semi-solid foods	Anything else: any food or liquid including non-human milk and formula	NA
Breastfeeding	Breast milk (including milk expressed or from a wet nurse)	Anything else: any food or liquid including non-human milk and formula	NA
Bottle-feeding	Any liquid (including breast milk) or semi-solid food from a bottle with nipple/teat	Anything else: any food or liquid including non-human milk and formula	NA

As stated in Section 1.3.3 a pragmatic approach has been adopted to the definition of exclusive breastfeeding to be used in the EAT study. To reiterate, it will be deemed acceptable for mothers to have given boiled then cooled water, as well as oral rehydration solution, without being considered to have ceased exclusive breastfeeding as this will not have any immunomodulatory effect on the infant.
When the study results are published, the percentage of women who achieved three months exclusive breastfeeding by the WHO criteria in Table 7 in each group will be presented.

3.10 STOPPING RULES

3.10.1 ONGOING REVIEW

The steering committee will review safety data on an ongoing basis. Safety data will be forwarded to the EAT Independent Data Monitoring Committee (IDMC). The IDMC may stop enrolment or participation in the trial at any time if it concludes that there are significant safety concerns (see Section 8).

3.10.2 REVIEW OF SPECIFIC ADVERSE EVENTS

3.10.2.1 STOPPING ENROLMENT

Enrolment in the trial will be stopped pending review if:

- Any death occurs
- A participant is admitted to an intensive care unit for an adverse event related (NCI-CTCAE attribution of adverse events Code four or five – see Section 8.5.2 Table 2) to study intervention.
- A participant randomly assigned to the allergenic food consumption group experiences life-threatening anaphylaxis during the first three weeks of administration of the allergenic food.

Enrolment in the trial will be stopped pending review if either of the following occurs for the early introduction group:

- An analysis performed when 200 such participants in the early introduction group have been followed for three months demonstrates that the lower bound of the 95% confidence interval for the proportion of participants in the early introduction group with IgE mediated food allergy as determined in an unscheduled clinic visit (see section 3.5 and Figure 7) is greater than 6%.

  Amendment

  In light of the reduced recruitment target it was jointly agreed by the IDMC and EAT TSC that this analysis would be undertaken when 100 such participants in the early introduction group have been followed for three months, rather than 200.
- An analysis performed when 200 participants per group have been followed up to 12 months of age demonstrates that the rate of serious adverse events is significantly greater at the 0.05 significance level in the allergenic food consumption group than in the standard introduction group.
- An analysis performed when ten such participants in the allergenic food consumption group have been followed until 12 months of age demonstrates that four or more participants have experienced a related (NCI-CTCAE attribution of adverse events Code four or five – see Section 8.5.2 Table 2) serious adverse event.

The analysis for these three categories will be undertaken by the trial statistician and the results conveyed to the IDMC.

3.11 STUDY DURATION

Enrolment is expected to take two years and four months. Infant/child participation is for three years. The study duration is expected to be seven years and four months.

4. ELIGIBILITY

4.1 INCLUSION CRITERIA

Either

1. Pregnant mothers attending their 12/20 week ultrasound scans.

   Or

2. Mothers planning on exclusively breastfeeding for at least the first three months
3. Informed consent obtained from parent or guardian.

4.2 EXCLUSION CRITERIA

1. Significant antenatal anomaly at 20 week ultrasound scan.
2. Multiple pregnancy
3. Significant congenital disease (enteropathy, congenital heart disease, renal disease)
4. Significant postnatal health concerns
5. Any history of stridor irrespective of cause
6. Premature delivery (less than 37 completed weeks gestation)
7. Parents not planning on exclusively breastfeeding for at least the first three months.
8. Parents planning on moving abroad before their child is three years of age
9. Both parents unable to speak and read English
10. Unwillingness or inability to comply with study requirements and procedures.
11. Family intend infant to be on a restricted diet (any of the six intervention foods)
12. ALT or bilirubin >2 times the upper limit of age-related normal value.

    ALT >144 (male) >126 (female),[3] *Bilirubin >42*
13. Urea or creatinine >1.25 times the upper limit of age-related normal value.

    Urea >10.3, Creatinine >60
14. Platelet count <100,000/ml, haemoglobin <9 g/dl, or investigator-suspected immunocompromise.
15. Significant vitamin D deficiency (alkaline phosphatise >1000 kU/L).
16. Infant participating in another study prior to enrolment

4.3 PREMATURE TERMINATION

4.3.1 PREMATURE TERMINATION OF TRIAL INTERVENTIONS

Trial intervention will be prematurely terminated for a participant if, in the judgment of the investigator, further participation in the trial would be deleterious to the participant's health.

4.3.2 PREMATURE TERMINATION FROM THE TRIAL

Participants will be prematurely terminated from the trial for either of the following:

- Withdrawal of consent
- Failure to return

Such participants will not be replaced.

5. STUDY INTERVENTION

5.1 ALLERGENIC DIET ADMINISTRATION OR AVOIDANCE

Infants who are randomized at three months of age to the early introduction group of the study will be provided with an individualized feeding plan. Infants will first be offered a pureed fruit/vegetable or baby rice mixed with breast milk or water. Then cow's milk based yoghurt will be commenced. Subsequently the other allergenic foods – egg, fish, sesame and peanut will be introduced in randomized order with the aim of all foods being ingested in the required quantities by five months of age. Wheat will be introduced last and not before four months of age.

The required quantity of each allergenic food to be ingested by five months of age is four grams of food protein per week. This approximately equates to the following quantities per week:

Cow's milk: Two small pots of yoghurt

Sesame: 3 teaspoons of tahini (sesame paste)

Wheat: two wheat based biscuit cereal (e.g. Weetabix)

Egg: one small egg

Fish: ¼ fish fillet or two fishfingers (25 grams fish)

Peanut: 3 teaspoons (rounded) of peanut butter

Infants in the early introduction group will be required to consume the allergenic foods until 1 year of age, at which point ongoing consumption of all six allergenic foods will be strongly encouraged. This is unlikely to be an issue with regard to cow's milk and wheat and in the majority of families for egg and fish which will be consumed as part of the normal family diet on a regular basis. Sesame and peanut will be the only two foods for whom the encouragement to continue consuming the foods might result in the child consuming a diet significantly different to that which they would have done otherwise. At three years of age, subsequent consumption of the allergenic foods will be left to parental choice.

Participants assigned to the standard introduction group will follow the UK Government infant feeding advice i.e. exclusive breastfeeding until around six months of age and no introduction of allergenic foods before this age.

COT recommendations

As stated in Section 1.3.3, the Government revised the COT recommendations on peanut consumption during pregnancy, lactation and in infancy in high risk families on the 25th August 2009. Up until this date, mothers in the standard introduction group who fulfilled the criteria stipulated by COT with regard to being at high risk of atopy (Section 3.1.3.1 above) have been advised to follow the postnatal recommendations of the committee:

- In common with the advice for all children, infants with a parent or sibling with atopic disease should, if possible, be breastfed exclusively for four to six months
- During weaning, and until they are at least three years of age, peanuts and peanut products should be avoided

After 25th August 2009, mothers in the standard introduction arm will follow the new guidance which states: "If your child already has a known allergy, such as a diagnosed food allergy or diagnosed eczema, or if there is a history of allergy in your child's immediate family (if the child's parents, brothers or sisters have an allergy such as asthma, eczema, hay fever, or other types of allergy), then your child has a higher risk of developing peanut allergy. In these cases you should talk to your GP, health visitor or medical allergy specialist before you give peanuts or foods containing peanuts to your child for the first time."

Methods to facilitate compliance with UK Government infant feeding advice
When mothers are approaching their due date, they will be sent information detailing the support that is available in their particular hospital with establishing breastfeeding. Further encouragement and advice will be provided by the study dietitians at the three month clinic assessments. Any mother experiencing difficulty with breastfeeding will be able to contact us via a dedicated study helpline and given practical advice to encourage breastfeeding for at least six months in both groups.

5.2 ASSESSMENT OF COMPLIANCE WITH STUDY INTERVENTION

The study dietitians will monitor compliance with the intervention. Compliance with the diet will be assessed by parent completed questionnaire with the following frequency:

Age	Frequency	Total number of interim compliance assessments*
3-12 months	Monthly	8
1 year until 3 year assessment	Three monthly	7

* excluding clinic assessments

Each compliance assessment will document:

1) Consumption of the six allergenic foods (both quantity and frequency) in both the early introduction group and the standard introduction group. 2) Breastfeeding status – mothers experiencing difficulty with breastfeeding will be able to contact us via a dedicated study helpline.

3) Consumption of any other foods

4) Additionally the compliance assessments will enquire about a history of any allergic symptoms, including possible type 1 reactions to food, using the validated instruments described in the outcomes section in accordance with the schedule of events (see Appendix 3).

Validation of the dietary component of the compliance questionnaire
The allergenic food frequency questionnaire has been designed specifically for this study. It uses (with permission) data from the food diaries completed by parents of infants in the UK arm of the EuroPrevall study. This data enables lists to be compiled for each allergenic food of which foods constitute the most significant proportion of consumption of that particular allergen.

These lists have been produced and a short food frequency questionnaire compiled. We will validate the FFQ against a food diary in 50 parents. The compliance FFQ covers monthly periods during the first year of the child's life so the validation exercise will compare the FFQ covering a one month period followed by a food diary (not weighed as we are not using the FFQ for details of macro or micronutrient intake) taken over the next two weeks. To help ensure that the food diary days are representative of what was eaten when the FFQ was filled in we will ask mothers in the validation study to continue to feed their infant in the same manner for the two weeks of the diary and not to introduce any new foods into the infant's diet.

5.3 MODIFICATION OR DISCONTINUATION OF STUDY TREATMENT

Participants in the early introduction group will discontinue consumption of an allergenic food if a confirmed allergic reaction to the allergenic food or an adverse nutritional consequence attributable to consumption of the allergenic food is experienced. They will continue to consume the other allergenic foods and will remain in the early introduction group of the study.

These participants will remain in the study to receive a status assessment at three years of age.

6. STUDY PROCEDURES

6.1 VISIT WINDOWS

- Visits described in the schedule of events (Appendix 3) should occur as follows:
- Visit three months: within +4 weeks of the planned visit date.
- Visit 12 months: +3 months of the planned visit date.
- Interim compliance assessments:
  - Between visits 3 and 12: ±14 days.
  - Between visits 12 and 36: ±1 month.
- Visit 36 months: within -6/+12 months of the planned visit date.
- Unscheduled clinic visit: within +1 month of being notified of episode
- Food challenges: within +1 month of the decision being taken to undertake a challenge

6.2 GENERAL ASSESSMENTS

General assessments will take place on our dedicated paediatric clinical trials facility in the Evelina Hospital at St Thomas'. For the final three year visit we will offer a home visit where families refuse or are unable to return to St Thomas'.These general assessments will be performed as per the schedule of events (Appendix 3):

- Informed consent. Written informed consent will be obtained for the mother's and child's participation in the study.
- Randomization.
- Dietary education. Parents will be provided with written advice to assist with attaining the UK Government target of around six months exclusive breastfeeding in the standard introduction group and at least six months breastfeeding in the early introduction group. Dietitians will provide written and verbal information and advice regarding the early introduction of the allergenic foods or standard UK Government infant feeding advice for the intervention and standard introduction groups, respectively.
- Medical history. A history will be taken to determine if the participant has had any clinically significant diseases or medical procedures.
- Adverse events. Participants will be assessed for adverse events. All adverse events will be recorded on the case report forms (CRFs).
- Concomitant medications. Concomitant medications will be recorded on the CRFs.
- Dietary history. The dietary intake of the children will be formally evaluated at six months, 12 months and three years of age utilizing a five day food diary (three week days and a weekend) completed just prior to each time point. The five day food diary will capture typical food consumption and provide a breakdown of macro- nutrient intake and total energy intake.
- Food reaction history. A history will be taken to determine if the participant has had any clinically significant food-induced, immediate-onset allergic reactions.
- Eczema. Both subjective and objective eczema severity criteria will be recorded. The modified SCORAD evaluation will be used at all visits. Parents will also be asked to fill out the Patient-Oriented Eczema Measure (POEM) questionnaire.
- Skin barrier integrity – Trans-Epidermal Water Loss (TEWL) is a measurement which reflects the barrier properties of the stratum corneum and is increased when skin barrier function decreases. TEWL will be measured using the Aquaflux® closed chamber tewameter[108] and parents will be requested not to use any skin care products on their child's skin during the previous 24 hours. Measurements will be taken from uninvolved skin on the volar aspect of the forearm in a room maintained at constant temperature and humidity. Measurements will not be taken if the child is visibly distressed or crying.

- Rhinitis evaluation. Symptoms in accordance with the study definitions for seasonal and perennial rhinoconjunctivitis will be recorded.
- Asthma evaluation. Symptoms in accordance with the study definition for asthma will be recorded.
- Anthropometry. Skin fold thickness, weight and height and body mass index. Head circumference and abdominal circumference.
- Blood pressure
- Bioelectrical impedance *(subject to further funding)*. Results will be analyzed to determine fat and lean body mass.
- Dexa Scan *(subject to further funding)*. Dual energy x-ray absorptiometry (densitometry) will be performed to measure the amount of bone, muscle, and body fat.

6.3 QUESTIONNAIRE SCHEDULE

The schedule for questionnaire completion by parents participating in the study is outlined in Table 8. Mothers will be able to complete all of the questionnaires on line (paper versions will be available). An on line version of the food diaries is being developed for the study using the WISP software package produced by Tinuviel.

Table 15. Questionnaire schedule

Assessment point	Subject	Duration
20 weeks of pregnancy*	Maternal diet and health	30 minutes
34 weeks of pregnancy*	Maternal diet	20 minutes
**Post delivery**		
3 months	Family health, diet and environment	30 minutes
3 months†	Maternal diet in pregnancy	20 minutes
3 months	Maternal diet whilst breast feeding	20 minutes
4-12 months - monthly	Infant health and diet	20 minutes
6 months	5 day infant food diary	1 hour
One year assessment	5 day infant food diary	1 hour
15-35 months - 3 monthly	Infant health and diet	20 minutes
Three year assessment	Infant health and diet	20 minutes
Three year assessment	5 day infant food diary	1 hour

* Antenatally recruited mothers

† Postnatally recruited mothers

6.4 LABORATORY ASSESSMENTS

Ten millilitres of blood will be obtained at the three month and one year assessments and 20 mls at the three year assessment. These are small quantities of blood that will have no haemodynamic effect on the child. A local anaesthetic will be applied for the one year and three year samples to minimise discomfort. For the three month sample, mothers will be encouraged to breastfeed their infants during the procedure given the evidence for the analgesic effect of a concurrent breastfeed.[109] Routine haematological and chemistry laboratory assessments, which are detailed below, will be performed at the investigation site as per the Schedule of Events (Appendix 3).

- Haematology includes FBC with WBC differential and platelets.
- Serum electrolytes including Ca, PO_4, urea, Cr, total protein, and albumin.
- Serum lipids include cholesterol, triglycerides, and high- and low-density lipoproteins.
- Glycosylated haemogloblin
- Skin swabs
- Coeliac screen: IgA tissue transglutaminase antibody screen.

6.5 ALLERGY ASSESSMENTS

6.5.1 ALLERGENS ASSESSED

The following allergy assessments will be performed:

6.5.1.1 SKIN PRICK TESTS

Three month visit

- Intervention group: SPT will be undertaken for the six intervention foods in duplicate: egg (salmonella free raw egg white), milk (pasteurized whole milk), sesame (tahini paste), fish, wheat and peanut. Total (including duplicate positive and negative controls): 16.
- Standard introduction group: no SPT will be done as the intention is that mothers wean their infants as they would have done if they had not been participating in the study.

One year visit (all infants)

- SPT for the six intervention foods (all commercial SPT solutions): egg, milk, sesame, fish, wheat and peanut. Other foods: soya and kiwi. Aero-allergens: house dust mite, cat, dog, six grass pollen mix and three tree pollen mix. Total (including positive and negative controls: 15).

Three year visit (all infants)

- SPT as per one year visit. Also tree nuts: Brazil nut, hazel nut, cashew, almond and walnut and salmonella free raw egg white. Total (including positive and negative controls: 20).

6.5.1.2 SPECIFIC IGE MEASUREMENTS

All specific IgE measurements to the six intervention foods will be undertaken when a child has completed the study (at three years of age) (Section 1.3.5).

Three month visit (all infants)

- Sensitization to the six intervention foods.

One year and three year visits

- Foods as per the three month visit and an aeroallergen panel including: *D. pteronyssinus, D. farinae*, cat and dog dander, horse dander, timothy grass, cladosporum, silver birch, olive, mugwort and nettle.

6.5.2 SKIN PRICK TESTING: PROCEDURES AND INTERPRETATION

Prior to testing, ensure that the participant has not received short-acting antihistamine medications for at least 48 hours and/or long-acting antihistamine medications for at least seven days.

For the three month visit, the SPT for raw hen's egg white will be performed using Red Lion salmonella-free egg, for milk will be performed with pasteurised whole milk and for sesame with tahini paste. The other SPTs will be performed using commercial SPT solutions. Positive (histamine 10 mg/ml) and negative control (50% glycerol, 50% buffered saline) solutions will be used.

Tests will be performed on the forearm unless unaffected eczema-free skin patches are not available, in which case the skin on the participants back will be used for testing. Using a standardized lancet (ALK Abelló), the skin will be pricked through a drop of the extract, which will then be absorbed. Skin test sites should be measured after 15 minutes. The wheal (and flare at three months of age) should be measured at their widest diameters and its perpendicular and the mean measurement recorded.

The positive and negative control tests should be performed and measured prior to allergen SPT.

- If the negative control test is ≥3 mm, the testing should be rescheduled for approximately seven days time.
- If the histamine positive control is negative, then it should be repeated immediately. If the repeat test remains negative, then the testing should be rescheduled for approximately seven days' time.

For the food allergens tested at three months of age, the following rules apply:

- The SPTs will be performed in duplicate and the mean of the two tests will be recorded.
- If both results are ≥ 1 mm and there is a > 2 mm difference between the results, a third SPT will be performed and the mean of the two closest results will be recorded.
- If one result is < 1 mm and one result is > 1 mm, a third SPT will be performed. If two of three results are < 1 mm, 0 mm will be recorded as the final result. If two of three results are ≥ 1 mm, the mean of those two results will be recorded as the final result.

6.6 FOOD CHALLENGES

The food challenge programme for participants in the study is summarized in Table 16 (scheduled challenges) and Table 17 (unscheduled challenges) and Figures 9 & 10.

6.6.1 SCHEDULED CHALLENGES

Infants in the early introduction group will undergo challenges at the three month screening visit if they have a positive skin prick test result to an allergenic food (Figure 6). All children who have a positive skin prick test to one or more of the six intervention foods at the one year and/or three year assessments, or a history of a positive challenge under one year of age will be considered for a challenge, the timing and type of the challenge being in accordance with Table 16 and Figures 9 & 10.

6.6.2 UNSCHEDULED CHALLENGES

Unscheduled challenges will be performed as indicated at unscheduled clinic visits (see section 3.5 and Figure 7 & 8). Independent of the result of these assessments, participants will continue with all subsequent study assessments. These participants will remain in the study to receive a status assessment at three years of age.

6.6.3 REPEAT CHALLENGES

If a participant fails to complete a challenge (termed an incomplete challenge as opposed to an indeterminate challenge), he/she may be offered an opportunity to repeat the challenge at the investigator's discretion.

6.6.4 MINIMUM INTERVALS BETWEEN CHALLENGES

If a participant has a positive food challenge, a time interval will be set before any attempt is made at a further challenge. The interval has been determined by several factors: the proximity to a previous challenge that a parent would be willing to contemplate undertaking a further challenge and ensuring that it is possible to correctly identify the child's allergic status during the primary outcome period of between one and three years of age. The following minimal time intervals will be observed: cow's milk – 6 months, other intervention foods - 1 year.

Table 16. Challenge programme – scheduled challenge visits

Event	Allergy status	Food	Arm	Consumption frequency	Type of challenge	Dose regime (g protein)	Section
3 month visit	SPT+ve	All	EI	Not applicable	Open incremental challenge	2.0 g (0.1, 0.2, 0.5, 1.2)	6.6.7.1
1 year visit	SPT-ve	All	Both	Infrequent/Never (UCV+ve)	DBPCFC*	4.3g (0.1, 0.4, 1.3, 2.5)	6.6.7.3.2
	SPT+ve	All	Both	Frequent	Open cumulative challenge	4.3g	6.6.7.3.1
		E M F W	EI	Infrequent (Enrolment challenge +ve)	DBPCFC*	4.3g (0.1, 0.4, 1.3, 2.5)	6.6.7.3.2
		E M F W	Both	Infrequent/Never (UCV+ve)	DBPCFC*	4.3g (0.1, 0.4, 1.3, 2.5)	6.6.7.3.2
		E M F W	Both	Infrequent (No reaction)<br>Infrequent (UCV-ve)<br>Never	DBPCFC	4.3g (0.1, 0.4, 1.3, 2.5)	6.6.7.3.2
		S P	SI	Infrequent<br>Never	Deferred until 3 year visit – avoidance advised	-	
		S P	EI	Infrequent<br>(UCV+ve)<br>(Enrolment challenge +ve)	Deferred until 3 year visit – avoidance advised		
		S P	EI	Infrequent (No reaction)<br>Infrequent (UCV-ve)<br>Never	DBPCFC	4.3g (0.1, 0.4, 1.3, 2.5)	6.6.7.3.2
3 year visit	Any SPT	All	Both	Infrequent/Never (UCV+ve ≥1 yr)	DBPCFC*	5.3g (0.1, 0.4, 1.3, 3.5)	6.6.7.3.2
	SPT+ve	All	Both	Frequent	Open cumulative challenge	5.3g	6.6.7.3.1
		All	Both	Infrequent (No reaction)<br>Infrequent (UCV-ve)<br>Never	DBPCFC	5.3g (0.1, 0.4, 1.3, 3.5)	6.6.7.3.2

*NB Observe minimum interval since +ve UCV/+ve enrolment challenge (M 6mth, E W F S P 1 yr) (Section 6.6.6)

Table 17. Challenge programme – unscheduled challenge visits

Event	Allergy status	Food	Arm	Consumption frequency	Type of challenge	Dose regime (g protein)	Section
UCV (<1yr)	SPT+ve ≥5mm	All	Both	All	Challenge not done<br>Deemed allergic	-	
	SPT+ve <5mm	All	Both	All	Open incremental challenge	2.0 g<br>(0.1, 0.2, 0.5, 1.2)	6.6.7.1
	SPT-ve	All	Both	All	Open cumulative challenge	2.0g	6.6.7.2
	Any SPT	All	Both	Previous indeterminate challenge to food	Open incremental challenge	2.0 g<br>(0.1, 0.2, 0.5, 1.2)	6.6.7.1
UCV (1yr+)	SPT+ve ≥5mm	All	Both	All	Challenge not done<br>Deemed allergic	-	
	SPT+ve <5mm	All	Both	All	DBPCFC	4.3g<br>(0.1, 0.4, 1.3, 2.5)	6.6.7.4
	SPT-ve	All	Both	All	Open cumulative challenge	4.3g	6.6.7.4
	Any SPT	All	Both	Previous indeterminate challenge to food	DBPCFC	4.3g<br>(0.1, 0.4, 1.3, 2.5)	6.6.7.4

Table 18. Challenge programme – FPIES

Event	Allergy status	Food	Arm	Consumption frequency	Type of challenge	Dose regime (g protein)	Section
FPIES	Severe	All	Both	All	FPIES challenge<br>Low initial dose	1st 0.06g/kg<br>2nd 2g	6.6.10
	Non-Severe	All	Both	All	FPIES challenge<br>Standard initial dose	1st 0.3g/kg (max 3g)<br>2nd 2g	6.6.10

*NB Observe minimum interval since suspected FPIES event – 1 yr (Section 6.6.10)

6.6.5 CHALLENGES FOR NON-INTERVENTION FOODS

Oral food challenges for allergenic foods other than the six foods in the early introduction group (e.g. soya, kiwi and raw egg white) will be given when both the clinical history and the results of SPT for these allergens are inconclusive. Children with significant positive results to these foods (SPT ≥5mm) will be advised to avoid the food. Challenges, if required, will be performed according to standard clinical practice. Quantity of food protein offered for these foods will be the equivalent to an age appropriate portion.

6.6.6 CHALLENGE SEQUENCE FOR MULTIPLY SENSITIZED PARTICIPANTS

Participants found to be sensitized to multiple intervention foods at the 3 month enrolment visit will be challenged to the most sensitized food first and then in descending order of sensitization. However, wheat will not be given before 4 months of age and the order may need adjusting accordingly.

Participants found to be sensitized to multiple intervention foods at the one year visit will be challenged in the following order.

- Foods which the child is frequently consuming will be challenged first. Challenge/s will take place on the day of the one year visit if logistically possible.

  Infrequently or never consumed foods will be challenged in the order: milk, egg, wheat and fish, the rationale being that milk is likely to be outgrown most quickly, followed by egg and then the other foods. NB if children have had a previous +ve UCV the minimum interval between challenges to the same food must be adhered to (see Section 6.6.4). This is likely to require the participant to return after the one year visit to undertake the challenge. This would be done in accordance with the one year visit protocol (i.e. a raised skin prick test result does not preclude undertaking a challenge).

6.6.7 PROCEDURE FOR CHALLENGES FOR SUSPECTED IGE MEDIATED FOOD ALLERGY

Step 1: Perform Clinical Assessment

Forthcoming challenges will be discussed by the EAT study team at the weekly team meetings. On the day of the challenge, all children will be assessed by the attending EAT study physician to determine their suitability for a challenge. The EAT study team supervising a challenge will include a nurse, dietitian and the EAT attending physician. All EAT clinical staff will ensure that they undertake their annual training in paediatric resuscitation. A participant’s eligibility for a challenge is guided by the following criteria:

- The child has had no acute exacerbation of allergic signs or symptoms within the last week.
- The child has not received:
  - short-acting beta-2 agonists for 12 hours,
  - long-acting beta-2 agonists for 24 hours,
  - short-acting antihistamines in the last 48 hours, or
  - long-acting antihistamines in the last seven days.
- The child has no concurrent illness.

Prior to conducting challenges, do the following:

- Ensure that both oxygen and suction are in working order in the clinical trials unit.
- Ensure that all steps of the anaphylaxis protocol are in place and that all emergency drugs are prescribed.
- Check that the drug box containing emergency medications is complete and that it is readily available.

- Record baseline observations, including temperature, pulse, respiration, Sa02 and auscultation of the chest.
- High risk challenges. The following individuals should have a cannula placed prior to the challenge: participants with IgE mediated immediate hypersensitivity symptoms to an intervention food allergen **and** a history of life-threatening anaphylaxis (defined as an allergic reaction accompanied by any of the following: hypoxia, as evidenced by central cyanosis or oxygen saturation ≤89%; hypotension; loss of consciousness; or admission to intensive care), or a reaction to any food which caused dehydration and required intravenous fluid resuscitation.
- PICU should be informed for high risk challenges
- Ensure that a child with a latex allergy or suspected latex allergy avoids all latex products.

Step 2: Prepare the Food to be used in the Challenge

The dietitians will prepare the challenge foods and any carrier foods. The foods will be labelled in terms of allergen name and quantity of food protein in the ward kitchen.

Step 3: Perform the Challenge

6.6.7.1 CHALLENGES UNDER ONE YEAR – INCREMENTAL OPEN CHALLENGE (2.0G)

Infants who are sensitized at enrolment in the early introduction group (Figure 6) or with aversion and/or refusal in the early introduction group or a suspected allergic reaction (both arms) with a skin prick test response of >0mm and <5mm ((Figure 7) or those who have an indeterminate challenge (cumulative or incremental) will undergo an incremental open challenge with the intention that the total amount of food protein consumed is the equivalent of a single EAT recommended portion of the intervention foods (2 grams of food protein).

- Administer four doses of allergenic food protein of 0.1, 0.2, 0.5 and 1.2 g in separate meals

6.6.7.2 CHALLENGES UNDER ONE YEAR - CUMULATIVE OPEN CHALLENGE (2.0 G):

In cases where the skin prick test result at an unscheduled clinic visit to the suspected allergenic food is negative and the history is of a reported acute food reaction, participants will be offered a cumulative single dose open challenge.

- Administer a total dose of allergenic food protein of 2.0 g in a single meal (may be split into 2 separate meals with an interval of 30min-1 hour if needed depending on the infant's appetite.

6.6.7.3 ONE AND THREE YEAR VISIT CHALLENGES

The type of challenge will be determined by the frequency with which the participant is consuming the food in question using the definitions given in section 3.6 previously:

Frequent consumers:

1. Consuming at least one EAT portion (≥2 grams of food protein) of the food within the last month.
2. History of ever having consumed more than three EAT portions (≥2 grams of food protein at a time) of the food

Infrequent/Never:

1. Does not fulfil the above two criteria.

6.6.7.3.1 FREQUENT CONSUMERS

- Administer one open dose (4.3g at less than three years and 5.3g food protein at three years).
- The open dose may be split into 2-3 smaller servings to be given over a period of 30-60 minutes.
- In the event of a positive open challenge, the participant will return for a subsequent DBPCFC.
- Up to two foods will be assessed in one day in the event of a participant being found to be sensitized to more than one food that they are frequently consuming at the one and three year visits.

6.6.7.3.2 INFREQUENT/NEVER CONSUMERS

- Administer three active doses of 0.1, 0.4, 1.3 g food protein, interspersed with two placebo doses and then one open dose (2.5g at less than three years and 3.5g food protein at three years).
- The meals will be blinded by a code known to the dietitian but not to the participant, nurse, or doctor. After discussion with an investigator, the previous dose may be repeated if any of the following occur:
    - abdominal pain
    - nausea
    - chest pain
    - abnormal oropharyngeal sensation
    - unexplained behavioural change
- After each dose, observe the child for 20 minutes.
- Doses will be administered by a member of the EAT Study team who is blinded to the doses.
- The open final dose may be split into 2-3 smaller servings to be given over a period of 30-60 minutes.
- In special circumstances, if a child persistently refuses to take the blinded doses (e.g. a fussy/faddy eater), a decision can be made by the team to switch to an open incremental challenge regime of 0.1, 0.4, 1.3, 2.5 g food protein at one year of age, and to an incremental challenge regime of 0.1, 0.4, 1.3, 3.5 g food protein at three years of age.
- An additional active low dose (with matching placebo dose) of 0.025g food protein will be added to the protocol for infrequent/never consuming peanut/sesame participants at the three year visit who are found to be significantly sensitized (SPT ≥5mm) and who have never undergone a previous double blind challenge to the food. This is based on the LEAP experience of undertaking high risk challenges in their 5 year old children who are significantly sensitized.

Separate challenge

If an allergic reaction occurs following a placebo dose, perform a **separate challenge** as follows:

Day 1:

- Administer the same dose regime as above, all of which are either allergenic food protein or placebo.
- After each dose, observe the child for 20 minutes.

Day 2:

- Administer the same dose regime as above, all of which are either allergenic food protein

or placebo. If allergenic food protein was administered on day 1, then administer placebo on day 2, or vice versa.

- After each dose, observe the child for 20 minutes.
- A dose may be repeated at the discretion of the investigator.

Allergenic food protein may vary and be used interchangeably.

Both groups

- Prior to the administration of each meal, the child will be evaluated for signs of reaction and vital signs (temperature, pulse, respiratory rate and SaO_2) will be monitored.
- The challenge should be discontinued at any stage if a protocol defined reaction occurs, and action will be taken according to local hospital guidelines.
- Total food protein intake is 4.3g at less than three years and 5.3g at three years.

6.6.7.4 UNSCHEDULED VISIT CHALLENGES AT 1 YEAR OF AGE OR MORE

Participants will undergo a DBPCFC if SPT is positive at <5mm as per section 6.6.6.4.2. If the SPT is negative and the history is of an acute food reaction, participants will undergo an open cumulative challenge with 4.3g of food protein.

<u>Step 4: Determine the Outcome</u>

Outcome of the challenge will be determined by evaluating the participant using the criteria in Table 18.

Table 19. Criteria for determining the outcome of food challenge

Major Criteria
Confluent erythematous pruritic rash
Respiratory signs (at least one of the following):<br>wheezing<br>inability to speak<br>stridor<br>dysphonia<br>aphonia
≥3 urticarial lesions
≥1 site of angioedema
Hypotension for age not associated with vasovagal episode
Evidence of severe abdominal pain (such as abnormal stillness or doubling over) that persists for ≥3 minutes
**Minor Criteria**
Vomiting
Diarrhoea
Persistent rubbing of nose or eyes that lasts for ≥3 minutes
Persistent rhinorrhoea that lasts for ≥3 minutes
Persistent scratching that lasts for ≥3 minutes

A positive food challenge will be defined by the presence of either of the following:

- One or more major criteria.
- Two or more minor criteria.

An indeterminate food challenge will be defined by the presence of one minor criterion.

A negative food challenge will be defined by the absence of major or minor criteria.

All symptoms should be of new onset and not due to ongoing disease. Symptoms must occur no later than two hours after the last dose.

Step 5: Consult with the Family

If the result is negative:

- Advise the family regarding consumption of the allergen with regard to their study arm and age.
- No emergency plan is required.

If the result is positive:

- Advise the family that the child must avoid the allergenic food in the diet.
- Advise the family regarding consumption of other allergenic foods with regard to their treatment arm and age.
- Provide a detailed written emergency management plan.
- Provide education on avoidance strategies.
- Provide training in Epi-Pen administration (once weight exceeds 7.5 kg) if participant has history of wheezing or anaphylaxis.
- Review the child's inhaler technique if appropriate.
- Encourage the parents to join the UK Anaphylaxis Campaign and Medic-Alert.
- Schedule follow up appointment for the participant in an appropriate allergy clinic.

Step 6: Discharge the Participant

Observe the child until:

- Two hours have elapsed since the top dose of the challenge (one hour for frequent consumer open challenges).
- All symptoms have resolved (if the result was positive).
- The clinician confirms that the child is ready for discharge.

After the observation period is over, remove the cannula if one was installed.

6.6.8 PROCEDURE FOR CHALLENGES FOR SUSPECTED NON-IGE MEDIATED FOOD ALLERGY

Parents who report symptoms suggestive of non-IgE mediated food allergy:

- Vomiting/recurrent posseting/reflux
- Recurrent diarrhoea/loose stools
- Blood in stools
- Worsening of eczema

AND the symptoms are **temporally related** to ingestion of allergenic foods (most likely to be cow's milk or egg) will be advised to take out the suspected allergen from the child's diet and the maternal diet if the mother is still breastfeeding for a two week period. If this results in no change in the child's symptoms they will be advised to reintroduce the food. If the exclusion results in an improvement they will be asked to contact the paediatric allergy unit to arrange to be assessed and undertake a ten day (five days allergen, five days placebo) home based DBPCFC if appropriate (skin prick test negative to suspected allergen) (Figure 8).

The protocol used for these challenges is based on that used in EuroPrevall (with permission):

- Children attend the allergy unit for initial assessment and determination of their IgE skin prick status. Positive children follow the IgE mediated UCV algorithm.

- Skin prick negative children will consume half of the first dose (either allergen or placebo) in hospital to check the acceptability of the preparation to the child
- The parent/s will be given the remaining doses of the challenge to take home
- After each step (five days of allergen or placebo) and the interim gap period (1 week) the child will undergo a study doctor guided parental assessment
- Parents will keep a symptom diary throughout the period of the DBPCFC
- Eczema status will be assessed using SCORAD and POEM at all three points (before, after initial five days of allergen/placebo and after 2nd five days of the allergen/placebo).

6.6.9 PROCEDURE FOR SUSPECTED COELIAC DISEASE

Parents who report symptoms suggestive of coeliac disease will have testing undertaken for measuring IgA tissue transglutaminase.

- Chronic or intermittent diarrhoea
- Failure to thrive or faltering growth
- Persistent or unexplained gastrointestinal symptoms including nausea and vomiting
- Prolonged fatigue
- Recurrent abdominal pain, cramping or distension
- Sudden or unexpected weight loss
- Unexplained iron-deficiency anaemia, or other unspecified anaemia

As IgA anti-endomyseal antibodies particularly, but also IgA tissue tansglutaminase antibodies can be negative under two years of age consideration will be given to measuring IgA anti-gliadin antibodies in this age group.

In order for any of serological tests for coeliac disease to be valid the participant will need to be consuming a diet which contains gluten in more than one meal every day for at least six weeks prior to testing.

6.6.10 PROCEDURE FOR SUSPECTED FOOD PROTEIN INDUCED ENTEROCOLITIS SYNDROME (FPIES) REACTIONS

A typical FPIES reaction will be suspected when the following criteria are fulfilled:[110]

(1) Age younger than 9 months of age at initial diagnosis
(2) Repeated exposure to the incriminated food elicits diarrhoea and/or repetitive vomiting within 24 hours without any other cause for the symptoms
(3) There are no symptoms other than gastrointestinal symptoms elicited by the incriminated food
(4) Removal of the offending protein from the diet results in resolution of the symptoms.

Subjects fulfilling the clinical criteria of FPIES who are older than 9 months of age at diagnosis or who have IgE antibodies to the incriminated food will be considered to have “atypical” FPIES.[110]

These children will not undergo an immediate food challenge at their initial UCV. Further challenges will not take place until at least a year has elapsed since the FPIE reaction in accordance with best recommended practice.

Challenges for food protein–induced enterocolitis syndrome should be administered in a setting in which intravenous access can be secured and prolonged observation is possible because severe gastrointestinal reactions may occur hours after the challenge.[58] In non–IgE-mediated food protein–induced enterocolitis syndrome in which there is a low risk for immediate reactions, with symptoms usually starting within 1 to 4 hours after food ingestion, the entire portion of the challenge may be administered gradually in 3 feedings over a period of 45 minutes (Table 18). The total challenge dose is calculated as 0.3 g protein/kg body weight, not to exceed 3 g protein or 10 g whole food. In patients with a previous history of severe reactions, a lower starting dose of 0.06 g protein/ kg body weight will be used.[58] If the patient remains asymptomatic for 4 hours, a second dose is given, generally an appropriate single-serving amount followed by 2 to 3 hours of observation.

7. TOLERANCE ASSAYS

Subject to further funding the following would be conducted

7.1 FROZEN PBMC T-CELL ASSAY

7.1.1 OVERVIEW

Subject of further funding, cryobanked PBMCs will be used in assays monitoring functional changes in T cells. Samples will be cultured in vitro and stimulated with food antigens to see if there is antigen-specific lymphocyte proliferation and to measure cytokines secreted in response to these antigens. Cells from the stimulated cultures will be pelleted and banked for RNA extraction and the supernatants frozen for future secreted cytokine screening.

7.1.2 GENE EXPRESSION PROFILING

Gene expression profiling will be performed using RNA isolated from non-stimulated versus in vitro stimulated cells. In this case, genes expressed in response to food and aeroallergen antigens will be compared in participants believed to be tolerant versus those who go on to develop food allergy. Genes of interest include IL-4, IL-5, IL-13, and IFNγ to determine if an allergic participant's T-cell responses are more T_H2-like; whereby a tolerant participant's T cells may respond to antigen in a more T_H1-like fashion. Alternatively, genes involved in mediating immune regulation by T regulatory cells may also be differentially expressed. Of particular interest is FoxP3, which will also be analyzed and directly compared to intracellular cytokine staining. Recently IRF-4, IRF-5, and IRF-7 have been shown to be associated with Toll-like receptor signalling and inflammatory responses. These, among other genes, are potential candidates for study.

7.1.3 SECRETED CYTOKINES

Cytokines secreted by in vitro stimulated cells will be monitored with the Luminex platform, a multiplex assay currently allowing for detection of 30 cytokines and chemokines from very small volumes of cell culture supernatant or serum. This analysis will be compared with gene expression and ICS data to determine if cytokines produced by T cells are released and to examine the difference between secreted cytokine profiles of tolerant and allergic participants.

7.1.4 ANTIGEN-SPECIFIC PROLIFERATIVE AND CYTOKINE RESPONSES

Antigen-specific proliferative and cytokine responses will be assessed using in vitro cultures with food and aeroallergens as described above. Using CFSE dye as a label, we will determine how many cell divisions have occurred in response to food and aeroallergens in vitro and determine the phenotype of proliferating cells with multicolor flow cytometry. Proliferative responses may be lower in tolerant versus allergic participants if active regulation is occurring for example. Additionally, intracellular cytokine production will be measured with ICS to determine profiles of cytokines secreted by the proliferating and nonproliferating T cells.

7.1.5 REGULATORY T CELLS

Regulatory T cells play an important role in down-modulating active or inflammatory immune responses. In this case, early antigen exposure may induce tolerance by activating antigen specific regulatory T cells. Currently, regulatory T cells are isolated using CD4 and CD25 surface markers, specifically gating on $CD25^+$ hi cells. $CD4^+CD25^+$ cells are mixed with $CD4^+CD25^-$ cells to evaluate the impact of regulatory T cells on antigen-specific proliferative responses by $CD25^-$ cells. Again, if tolerance to food allergens is achieved, there may be increased regulatory T-cell activity. This assay is likely to evolve given the use of FoxP3 and potentially other markers for enumeration and isolation of regulatory T cells.

7.2 PLASMA ALLERGEN-SPECIFIC IGG AND IGE

7.2.1 ELISA-BASED TECHNIQUES

ELISA-based techniques will be used to measure food and aeroallergen-specific IgG4 and IgE during the study using cryobanked serum samples. Previous studies have shown that children who are sensitized but not allergic to specifically peanut have higher IgG4/IgE ratios than those who are allergic to peanut. Interestingly, the fact that both sensitized and allergic children are IgE positive suggests active regulation by T cells, which results in the higher IgG4 levels in the non-allergic sensitized patients. Higher IgG4/IgE ratios found in sensitized versus allergic patients should correlate with food allergen-induced tolerance in this study.

7.2.2 FACILITATED ANTIGEN PRESENTATION INHIBITION

Facilitated antigen presentation (FAP) inhibition is a flow cytometric-based assay that can detect participants who have allergen-specific IgG antibodies that interfere with FAP. In FAP, IgE facilitates the presentation of antigen to B cells, subsequently causing allergy-related, T-cell activation. Immunotherapy induces IgG, which competes for allergen-bound IgE, thus inhibiting allergen/IgE complexes from binding Fc receptors on antigen presentation cells (in this case, a B-cell line). Food allergen consumption will increase food allergen-specific IgG, especially IgG4, which interferes with FAP. The time course and magnitude of changes in plasma FAP inhibitory activity will be compared with clinical symptoms, clinical scores, and allergen-specific IgG levels.

7.3 WHOLE BLOOD DNA–HLA GENOTYPES

There are some indications that HLA class II genetic polymorphism may be associated with susceptibility to peanut allergy. DNA will be isolated from trial participants, subjected to sequence-based class II typing, and genotyped for potential SNPs associated with persons susceptible to allergic responses. DNA will also be analysed for skin barrier gene mutations.

8. Adverse Events

8.1 OVERVIEW

Safety data will be recorded on a CRF specifically designed for this purpose. All serious adverse events (SAEs) will be reported on an SAE report form as well as on individual CRFs. All safety data will be reviewed periodically by the independent data monitoring committee (IDMC). In addition, SAEs will be reported locally. The IDMC has the authority to withdraw any participants and/or terminate the study because of safety findings.

Adverse events that are classified as serious according to the definition of health authorities must be reported promptly and appropriately to the IDMC, the FSA, principal investigators in the trial, local ethics committee and any relevant NHS Trust. This section defines the types of adverse events and outlines the procedures for appropriately collecting, grading, recording, and reporting them.

8.2 INDEPENDENT DATA MONITORING COMMITTEE (IDMC)

Also known as the Data Safety Monitoring Board (DSMB).

8.2.1 TERMS OF REFERENCE

- To monitor and review ongoing data from the trial and undertake interim analyses if appropriate.
- In the light of the above, and ensuring that the safety, rights and well being of the trial participants are paramount, to report (following each IDMC meeting) to the Trial Steering Committee and to recommend on the continuation of the trial.
- To consider any requests for release of interim trial data and to recommend to the TSC on the advisability of this.
- In the event of further funding being required, to provide to the TSC appropriate information and advice on the data gathered to date that will not jeopardise the integrity of the study.

8.2.2 MEMBERSHIP

Membership of the EAT Study IDMC has been organized by the FSA/MRC. As per MRC recommendations the membership consists of four individuals with expertise in the areas of paediatric allergy, paediatric research and trial management. One member is a trial experienced statistician.

8.2.3 GUIDANCE NOTES

- The IDMC will meet at least annually, or more often as appropriate, and meetings will be timed so that reports can be fed into the Trial Steering Committee (TSC) meetings. Meetings will be called for and organised by the Chair of the IDMC. Dates for IDMC meetings will be agreed in advance and only altered with agreement of all members. The IDMC will communicate to the TSC chair to chair. Any oral communication will be backed up by written records.

- The trial statistician will prepare a comprehensive report for the IDMC. This will be prepared and circulated well in advance of the meeting to allow IDMC members time to study the data. Content of the report will be agreed in advance with the IDMC Chair. The trial statistician may be invited by the Chair to attend part of the meeting to present the data; otherwise, no one involved with the trial or TSC will be present to see the unblinded data.
- A confidential report will be made in writing by the Chair of the IDMC providing advice to the chair of the Trial Steering Committee on whether the trial should continue or not or be altered. If the IDMC recommends that the trial should be stopped at any point, the TSC chair will inform the FSA/MRC.
- If at any stage an extension to the grant is needed the IDMC may be requested by the TSC to provide information on the data gathered to date (from this and other studies) and advice on the likelihood that continuation of the trial will allow detection of an important effect. This will be done using methods that do not unblind the trial.
- Before reporting on the results of the trial the IDMC will consider not only the interim results as presented by the trial statistician, but also any major new information from other sources thought to be relevant to the trial. It follows that the IDMC will not automatically follow pre-assigned statistical rules, although it will be guided by statistical considerations.
- Information provided by the IDMC is likely to fall into the following categories:

  (a) Information that might lead to the TSC stopping the trial prematurely in the event of a clear outcome, if this is deemed to be appropriate in the light of the accumulating data from the study, or on the basis of information available from other sources;

  (b) Information that might lead to the TSC modifying the design of the trial, if this is deemed to be appropriate in the light of the accumulating data from the study or on the basis of information available from other sources.

8.3 DEFINITIONS

8.3.1 ADVERSE EVENT

An adverse event is any occurrence or worsening of an undesirable or unintended sign, symptom, laboratory finding, or disease that occurs during participation in the trial.

An adverse event will be followed until it resolves or until 30 days after a participant terminates from the study, whichever comes first.

8.3.2 SERIOUS ADVERSE EVENT

A Serious Adverse Event (SAE) is any untoward and unexpected medical occurrence or effect that:

- Results in death. A death that occurs during the study or that comes to the attention of the investigator during the protocol-defined follow-up after the completion of therapy must be reported whether it is considered treatment related or not.
- Is life-threatening – refers to an event in which, in the view of the investigator, the subject was at risk of death at the time of the event
- Requires inpatient hospitalization or prolongation of existing hospitalization.
- Results in persistent or significant disability or incapacity
- An event that requires intervention to prevent permanent impairment or damage. An important medical event that may not result in death, be life threatening, or require hospitalization may be considered an SAE when, based on appropriate medical judgment, it may jeopardize the participant and may require medical or surgical intervention to prevent one of the outcomes listed above.

Regardless of the relation of the adverse event to study participation, the event must be reported as a serious adverse event if it meets any of the above definitions.

8.3.3 UNEXPECTED ADVERSE EVENT

Standard practice is that an adverse event is considered “unexpected” when its nature (specificity) or severity is not consistent with applicable product information, such as safety information provided in the package insert, the investigator’s brochure, or the protocol. For a nutritional interventional study such as the EAT Study, determining what is unexpected is less clear.

8.4 COLLECTING ADVERSE EVENTS

8.4.1 METHODS OF COLLECTION

Adverse events will be collected from the time the infant is enrolled at three months of age until the time the event resolves or until 30 days after the participant completes the study, whichever comes first.

Adverse events may be discovered through any of these methods:

- Observing the participant at the three month, 12 month and three year visits.
- From responses to the parent completed compliance questionnaires
- From active reporting by the parent via the dedicated study help line (24 hour landline with answer-machine outside of office hours) or via the study email address.

An abnormal value or result from a clinical or laboratory evaluation (e.g., an X-Ray, an ultrasound, or an electrocardiogram) can also indicate an adverse event. If this is the case, then the evaluation that produced the value or result should be repeated until that value or result returns to normal or can be explained and the participant’s safety is not at risk. If an abnormal value or result is determined by the investigator to be clinically significant, it must be recorded as an adverse event on the appropriate form(s).

8.4.2 COLLECTING SERIOUS ADVERSE EVENTS

Serious adverse events will be collected from the time the infant is randomized until 30 days after he/she completes study participation or until 30 days after he/she prematurely withdraws from the study.

8.4.3 RECORDING ADVERSE EVENTS

Throughout the study, the investigator will record all adverse events on the appropriate adverse event CRF regardless of their severity or relation to study medication or study procedure. The investigator will treat participants experiencing adverse events appropriately and observe them at suitable intervals until their symptoms resolve or their status stabilizes.

8.4.4 RECORDING SERIOUS ADVERSE EVENTS

Serious adverse events will be recorded on the adverse event CRF and on the SAE form, and health authorities will be notified as outlined in section 8.6.2.

8.5 GRADING AND ATTRIBUTION OF ADVERSE EVENTS

8.5.1 GRADING CRITERIA

The study site will grade the severity of adverse events experienced by study participants according to the criteria set forth in the National Cancer Institute - Common Terminology Criteria for Adverse Events (NCI-CTCAE) Version 3.0. This document provides a common

language to describe levels of severity, to analyze and interpret data, and to articulate the clinical significance of all adverse events.

Adverse events will be graded on a scale from one to five according to the following standards in the NCI-CTCAE manual:

Grade 1 = mild adverse event.

Grade 2 = moderate adverse event.

Grade 3 = severe and undesirable adverse event.

Grade 4 = life-threatening or disabling adverse event.

Grade 5 = death.

All adverse events will be reported and graded whether they are or are not related to disease progression or treatment.

8.5.2 ATTRIBUTION DEFINITIONS

The relation, or attribution, of an adverse event to study participation will be determined by the site investigator. The site investigator will also record the determination of attribution on the appropriate CRF and/or SAE reporting form. The relation of an adverse event to the study treatment will be determined using the descriptors and definitions provided in Table 19.

Table 20. NCI-CTCAE attribution of adverse events

Code	Descriptor	Definition
**Unrelated Category**		
1	Unrelated	The adverse event is clearly not related to study participation.
**Related Categories**		
2	Unlikely	The adverse event is doubtfully related to study participation.
3	Possible	The adverse event may be related to study participation.
4	Probable	The adverse event is likely related to study participation.
5	Definite	The adverse event is clearly related to study participation.

For additional information and a printable version of the NCI-CTCAE manual, consult the NCI-CTCAE web site: http://ctep.cancer.gov/reporting/ctc.html.

8.6 REPORTING ADVERSE/SERIOUS ADVERSE EVENTS

8.6.1 REPORTING TIMELINE

Depending on the nature of the event the reporting procedures below will be followed. Any questions concerning adverse event reporting will be directed to the Chief Investigator in the first instance.

Non serious AEs

All such events, whether expected or not, will be recorded.

Serious AEs

An SAE form will be completed and emailed immediately to the Chief Investigator.

8.6.2 OPTIONS FOR REPORTING SERIOUS ADVERSE EVENTS

All SAEs will be reported to the Research Ethics Committee. The St Thomas' LREC follows standard practice in defining specific SAEs that are required to be within 15 days of the Chief Investigator becoming aware of the event, using the COREC SAE form. These are events where in the opinion of the Chief Investigator, the event was:

- "related", i.e. resulted from the administration of any of the research procedures; and
- "unexpected", i.e. an event that is not listed in the protocol as an expected occurrence

However, given the difficulty within a nutritional intervention study of determining both what is "unexpected" (see Section 8.3.3) and what is "related", the EAT study team will report all SAEs to the St Thomas LREC within 15 days.

Additionally, at the IDMC meeting of 21st October 2010, it was agreed that all SAEs will be forwarded to the following bodies at the same time:

- The IDMC
- The FSA/MRC
- The GSTT Research & Development Office

In the event of an unexpected death which is regarded as related or possibly related to the study, the researcher will notify the Coroner; even if a Coroner's post-mortem is not needed, a PM will be obtained wherever possible.

9. STATISTICS

9.1 ANALYSIS SAMPLES

The following groups will form samples for analysis.

- ***Intention to Treat analysis***

All children allocated to each arm of the intervention regimen will be analyzed together as representing that treatment arm, whether or not they received or completed the prescribed regimen. This will be done after the three year assessment for the period prevalence of IgE mediated food allergy to the six intervention foods between one and three years of age in both arms (Section 3.3.1).

- ***Per Protocol analysis***

Definition of compliance

The criteria for the per protocol analysis of the period prevalence of IgE mediated food allergy to the six intervention foods between one and three years of age in both arms (Section 3.3.1) will consist of:

Per protocol early introduction group compliance

Criterion A: Exclusive breastfeeding for three months duration (water and/or oral rehydration solution would be an acceptable part of the diet).

Criterion B: Continued breastfeeding up to five months of age

Criterion C: Consumption of at least five of the allergenic foods in at least 75% of the recommended amount, on at least five weeks between three months and six months of age.

Per protocol standard introduction arm compliance

Criterion A: Exclusive breastfeeding for at least three months duration (water and/or oral rehydration solution would be an acceptable part of the diet).

Criterion B: Continued breastfeeding up to five months of age

Criterion C: No consumption of peanut, egg, sesame, wheat or fish before five months

Criterion D: Cow's milk (or goat's milk) consumption of less than 300 mls/day between three months and six months of age.

9.2 ANALYSIS OF ENDPOINTS

9.2.1 ANALYSIS OF PRIMARY ENDPOINT

The main analysis will compare the period prevalence of IgE mediated food allergy to the six intervention foods between one and three years of age in both arms (Section 3.3.1) using a two-tailed, chi-square test at the $p<0.05$ level of significance.

9.2.2 ANALYSIS OF SECONDARY ENDPOINTS

The incidence of adverse events and laboratory abnormalities at three years of age will be compared between the intervention and standard introduction arms using a two-tailed chi-square test at the $p<0.05$ level of significance. Additional specific secondary endpoints are described in section 3.4.

9.2.3 ADDITIONAL ANALYSES

Additional analyses of the risk associated with IgE-mediated food allergy will be performed using multiple logistic regression to identify possible associations of risk with baseline participant characteristics and selected clinical and dietary measures. These analyses will be performed on the incidence of IgE-mediated food allergy at three years of age.

9.3 SAMPLE SIZE

9.3.1 FULL COHORT

The trial has 90% power to detect a 50% reduction (based on a 2-tailed test) in the absolute prevalence of food allergy by three years of age (from an expected prevalence of 6% in the standard introduction group to 3% in the intervention group).

Contract variation September 2010

The trial has 80% power to detect a 50% reduction in the absolute prevalence of food allergy b y three years of age (from 8% in the standard introduction arm to 4% in the intervention group).

9.3.2 HIGH RISK SUBGROUP

The study is also designed so that it has sufficient power to detect a protective effect in the high risk sub-population defined as the 20% of participants with visible eczema at the three month assessment (10% randomized to each group). At the three month assessment there is anticipated to be 2500 participants remaining, 1125 in each group. After allowing for 20% drop out there will be 1000 participants remaining in each group at the final three year assessment. The study has 95% power to detect a reduction from 30% to 10% amongst the 100 (10%) high risk children in the intervention group compared with the 100 (10%) high risk children in the standard introduction group. However the power is still respectable to detect a 50% reduction in prevalence of food allergy, with 71% power to detect a reduction from 30% to 15%.

Contract variation September 2010

The high risk children are defined as being the 25% with eczema present at three months of age, who have an estimated prevalence of food allergy of 30%. It is estimated that with loss to follow up, there will be approximately 300 high risk children amongst those attending the one year assessment (90% of the 25% of the 1302 participants), 150 in each group. The study has 99% power to detect a reduction from 30% to 10% amongst the high risk participants in the intervention group and 85% to detect a reduction from 30% to 15%.

The study is not powered to detect a reduction in allergy to individual foods.

9.4 LOSS TO FOLLOW-UP

The antenatal recruitment target of 3000 mothers has been determined in order to allow for a loss of 500 women (16.7%) between maternal consent to participate at 20 weeks of pregnancy and her infant attending the three month assessment visit. Of the 2500 infants who are then enrolled, it has been estimated that 20% will have been lost to follow-up by the time of the three year final assessment. Thus we estimate that approximately 2200 will attend the one year interim assessment visit and 2000 the three year final assessment clinic.

The 20% estimate is derived from several sources: In the AllergyFlora study (based in a central London teaching hospital with a similar population) the loss to follow-up between enrolment at birth and 18 months of age was 15.6%. The 20% estimate is also identical to the loss to follow-up observed in the much larger German MAS study, where at the 36 month (three year) assessment the participation rate was 1050/1314 children (79.9%). In addition, in our immunotherapy intervention studies done by our team which involve regular injections over a

two to three year period, we have had only a 10% drop-out rate with patients obtained from across the London area.

Contract variation September 2010

1302 infants will be recruited onto the study at three months of age. The drop out figure has been revised to 15% (10% by the one year assessment) based on the low rate observed in the LEAP study and in the EAT study to date. It is therefore estimated that 1172 infants will attend the one year assessment. The final cohort will consist of 1106 participants (553 in each arm) at three years of age.

9.5 RANDOMIZATION, STRATIFICATION, AND BLINDING

Participants will be randomly assigned to treatment using a centrally administered randomization service (ALEA). The randomization will not be stratified given the number of participants. The study participants are unblinded with regard to the intervention. However, the study outcomes are based on objective measures of sensitization (skin prick test results) and specific IgE measurements. The latter are measured by laboratory staff blinded to the child's allocation status). Food allergy at one year and beyond is determined by the gold standard measure, the DBPCFC.

9.6 REPORTING DEVIATIONS FROM THE ORIGINAL STATISTICAL PLAN

The principal features of the study design and the plan for statistical analysis of the data are outlined in this protocol. Any changes in these features will require a protocol amendment which will be subject to review by the IDMC and the study sponsor. These changes will be described in the final report as appropriate.

10. IDENTIFICATION AND ACCESS TO SOURCE DATA

10.1 IDENTIFYING SOURCE DATA

The investigator is required to keep accurate records to ensure that the conduct of the study is fully documented (see section 11). The results of all clinical evaluations will be maintained in the participant's medical records and recorded on clinical CRFs. Safety blood results will be stored on a central database with results provided twice weekly by the GSTT laboratory.

Safety data will be recorded on CRFs specifically designed for this purpose. All the SAEs will be reported on an SAE CRF. All safety data will be reviewed periodically by the IDMC. The IDMC has the authority to withdraw any participants and/or terminate the study because of safety findings.

10.2 PERMITTING ACCESS TO SOURCE DATA

The investigational site participating in this study will maintain the highest degree of confidentiality permitted for the clinical and research information obtained from the participants in this clinical trial. Medical and research records should be maintained at each site in the strictest confidence. However, as a part of the quality assurance and legal responsibilities of an investigation, the investigational site must permit authorized representatives of the sponsor(s) and health authorities to examine (and when required by applicable law, to copy) clinical records for the purpose of quality assurance reviews, audits, and evaluations of the study safety and progress. Unless required by the laws that permit copying of records, only the coded identity associated with documents or with other participant data may be copied (and all personally identifying information must be obscured). Authorized representatives as noted above are bound to maintain the strict confidentiality of medical and research information that is linked to identified individuals. The investigational site will normally be notified before auditing visits occur.

11. QUALITY CONTROL AND QUALITY ASSURANCE

The investigator is required to keep accurate records to ensure that the conduct of the study is fully documented.

Audits and Inspections

The study may be subject to inspection and audit by Kings College London, under their remit as sponsor and other regulatory bodies to ensure adherence to ICH/MRC Principles of Good Clinical Practice[1;2] and the NHS Research Governance Framework for Health and Social Care (2nd edition).[111]

11.1 MANAGEMENT STRUCTURE

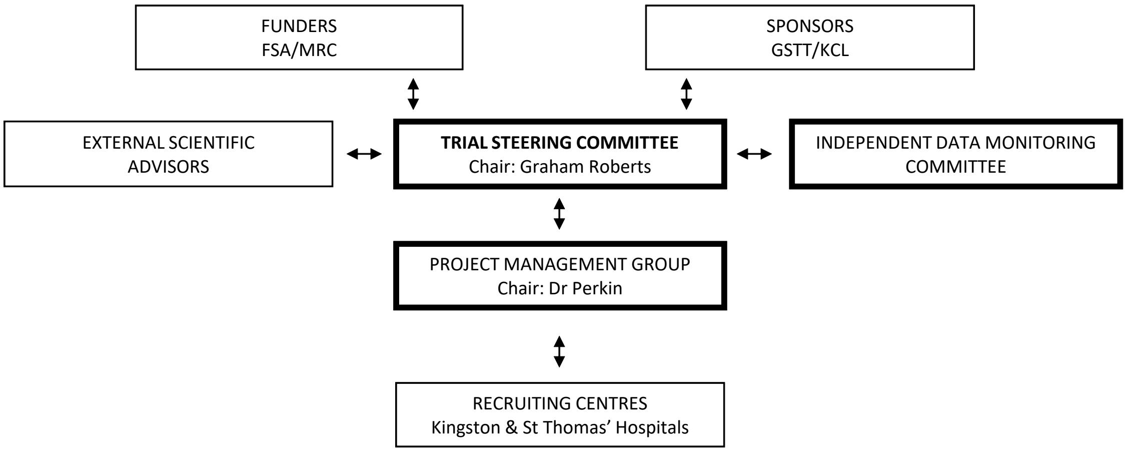

FIGURE 11. MANAGEMENT STRUCTURE

Introduction

The overall management ideals for the EAT Study are:

- That there will be fair representation of all members of the project on the decision making bodies of the project.
- That the management of the study will be completely transparent to its members, and to the FSA and MRC.
- That management will be flexible and responsive to the need for change as the project develops its science base.
- That management will be disciplined and effective.

Management structure (see Figure 10)

The EAT Study will be co-coordinated by Dr Perkin and Professor Lack. Professor Lack will be the designated co-ordinator for communication with the FSA. Dr Perkin will chair the Project Management Group.

The overall management structure consists of:

- Trial Steering Committee (TSC)
- Project (Trial) Management Group (PMG)
- Independent Data Monitoring Committee (IDMC)

The main roles, responsibilities, composition and meeting frequency of these bodies are described below.

11.1.1 TRIAL STEERING COMMITTEE

The TSC is the main decision making body. It has overall responsibility for scientific strategy and direction and has ultimate responsibility for ensuring the project's aims are delivered on time and within budget.

The TSC will include external representatives with appropriate expertise including paediatric nutrition and the design of robust methodologies for collecting dietary data. The members are:

Chair (voting)

Professor Graham Roberts, Professor in Paediatric Allergy, Southampton University

Independent members (voting)

Professor David Strachan, St George's University of London (Vice Chair)

Professor Christine Edwards, University of Glasgow

Mr David Reading, Lay Member

Dr Mary Fewtrell, Reader in Childhood Nutrition, UCL Institute of Child Health

Dependent Members (voting)

Professor Gideon Lack, Kings College London

Dr Michael Perkin, Kings College London

Professor Janet Peacock, Kings College London (Study Statistician)

Professor Ian Kimber, Professor of Toxicology, University of Manchester (on behalf of FSA)

Other members (non-voting)

Dr Kirsty Logan, EAT Study Research Fellow & Study Coordinator, Kings College London

Dr Carsten Flohr, Kings College London

Dr Salma Ayis, Kings College London

Observers (non-voting)

Ms Sarah Hardy, Food Standards Agency

Ms Shuhana Begum, Food Standards Agency

The major roles of the TSC will be:

- To make decisions necessary to ensure successful delivery of the EAT Study.
- To evaluate progress against the agreed timetable and deliverables.
- To administer the budget and monitor spending.
- To develop and implement successful communication between the study staff and external stakeholders (recruitment centres, funders and sponsors).
- To make decisions regarding the allocation and further analyses of biological samples.

The FSA/MRC will be notified of any significant changes to protocol or decisions taken by the TSC that may affect the progress or delivery of the project. In addition the TSC will also be responsible for:

- Development, implementation and evaluation of appropriate policies and procedures to facilitate the protection of knowledge and exploitation of results.
- In consultation with the FSA and MRC as funding bodies, development, implementation and evaluation of appropriate policies and procedures to ensure the effective dissemination of results to appropriate stakeholders.

The TSC will meet as scheduled in the Scope of Work document.

11.1.2 PROJECT (TRIAL) MANAGEMENT GROUP

The PMG is responsible for the day to day management of the EAT Study.

The main roles of the PMG will be:

- To ensure that the goals set by the Trial Steering Committee are met.
- Manage the day to day running of the project.
- Record project activity and monitor that the study milestones are met and that the deliverables are delivered.
- Monitor spending on the study.
- Arrange annual financial audit of spending.
- Ensure filing of all documentation accompanying the study arrangement and finances.
- Prepare reports for the Trial Steering Committee and FSA.
- Organise all meetings and provide administrative support to the TSC chairperson.
- Implementation of the study communication strategy.

The PMG will be chaired by Dr Michael Perkin and include Professor Lack and the study manager, and all EAT staff members present that day. The PMG will convene weekly to discuss all operational business. The study also benefits from a full time project manager on another intervention study so the two project managers will be able to support each other (e.g. whilst on annual leave).

11.1.3 INDEPENDENT DATA MONITORING COMMITTEE

The study has a completely independent IDMC (see Section 8.2).

11.1.4 EXTERNAL SCIENTIFIC ADVISORS

The TSC will seek advice when necessary from individuals who are leading international authorities in areas that can contribute to the design, implementation and analysis of the study. Individual members may be identified to attend specific steering group meetings when appropriate.

11.2 DATA HANDLING

Data Collection

All data will be collated centrally, coordinated by the project manager in the project office...

Data Monitoring (including compliance)

CRFs will be monitored for data accuracy by auditing a random sample of ten prior to Steering Committee meetings. Staff will receive training in the completion of CRF's, fulfilling the clinical trial protocol and training in Good Clinical Practice.

Data Storage

Documents will be stored within a locked room. Documents will be maintained in a legible condition, with prompt retrieval possible.

Sample tracking

We will use a bar code based tracking system for sample management. This specialised software system is designed to track where individual samples are for particular patients. This is invaluable for a multi-sampling large study such as this. The individual aliquots of cells, plasma or serum can be tracked so that the exact number of cells or vials left at any particular time point is known.

Archiving

Essential documents will be archived in an easily accessible way and readily available on request. All essential documents will be boxed and clearly labelled with the following information:

- Project/trial name
- Reference Number
- Site Number (if applicable)
- Chief Investigator
- Lead Site
- Date of Archive

The documents will be stored in a secure room, with appropriate environmental controls (and adequate protection from fire, without water sprinkler systems, water etc.), and access only by authorised personnel. All trial related documents will be centrally archived to prevent accidental damage, amendment, loss or destruction. Any change in the ownership and location of documentation will be documented in order to allow the tracking of the stored records. If archived documents are reviewed at a later date, we will record who and what documents were reviewed and the date they were accessed in an archive index/log.

All trial-related documents will be kept for a minimum of 15 years. For general clinical research, essential documents will be kept for a minimum of ten years. Once this time period has elapsed, a destruction log will be kept for a further five years, listing everything that has been destroyed and the reasons why it was destroyed.

12. Ethical Considerations and Compliance with Good Clinical Practice

12.1 STATEMENT OF COMPLIANCE

We will adhere to the general principles and standards in research as outlined by the General Medical Council in the document: Research: The Role and Responsibilities of Doctors (Feb 2002).

We will adhere to the principles of Good Clinical Practice in Clinical Research produced by the ICH and endorsed by the MRC.[1;2]

This clinical study will also be conducted in accordance with the FSA Joint Code of Practice for Research.

Ethical approval for the study will also be obtained. All relevant trusts within the National Health System in England will also be informed of the study.

12.2 INFORMED CONSENT AND ASSENT

Consent to enter the study will be sought from each participant only after a full explanation has been given, an information leaflet offered and time allowed for consideration. All participants (or their legally acceptable representative) must read, sign, and date a consent form before entering the study or undergoing any study-specific procedures. The right of the participant to refuse to participate without giving reasons will be respected. All participants are free to withdraw at any time from the protocol treatment without giving reasons and without prejudicing further treatment.

The informed consent form must be revised whenever important new safety information is available, whenever the protocol is amended, and/or whenever any new information becomes available that may affect participation in the trial.

12.3 PRIVACY AND CONFIDENTIALITY

A participant's privacy and confidentiality will be respected throughout the study. Each participant will be assigned a sequential identification number, and these numbers rather than names will be used to collect, store, and report participant information.

13. COMMUNICATION POLICY

13.1 PUBLICATION POLICY

The co-principal investigators in consultation with the FSA and MRC will produce a publication policy for collaborators and funders to sign with respect to subsidiary studies that may be performed on the cohort.

13.2 COMMUNICATION WITH PARTICIPANTS

Both the study website and dedicated study newsletters will be utilised to disseminate the results to all the participants.

We will ask families at the final three year visit for permission to remain in contact in order to continue to send them information about the study's findings and plans for the future.

14. References

(1) ICH. Harmonised Tripartitie Guideline for good clinical practice E6(R1). 1996.

(2) Medical Research Council. MRC guidelines for good clinical practice in clinical trials. 1998.

(3) England K, Thorne C, Pembrey L, Tovo PA, Newell ML. Age- and sex-related reference ranges of alanine aminotransferase levels in children: European paediatric HCV network. *J Pediatr Gastroenterol Nutr* 2009; 49(1):71-77.

(4) Thong BY, Hourihane JO. Monitoring of IgE-mediated food allergy in childhood. *Acta Paediatr* 2004; 93(6):759-764.

(5) Asher MI, Montefort S, Bjorksten B, Lai CK, Strachan DP, Weiland SK et al. Worldwide time trends in the prevalence of symptoms of asthma, allergic rhinoconjunctivitis, and eczema in childhood: ISAAC Phases One and Three repeat multicountry cross-sectional surveys. *Lancet* 2006; 368(9537):733-743.

(6) World Health Organization. Global Strategy for Infant and Young Child Feeding. 2002. Geneva.

(7) Department of Health. Infant Feeding Recommendation. 2003.

(8) Department of Health. Weaning - starting solid food. 2008.

(9) Bergmann RL, Diepgen TL, Kuss O, Bergmann KE, Kujat J, Dudenhausen JW et al. Breastfeeding duration is a risk factor for atopic eczema. *Clin Exp Allergy* 2002; 32(2):205-209.

(10) Sears MR, Greene JM, Willan AR, Taylor DR, Flannery EM, Cowan JO et al. Long-term relation between breastfeeding and development of atopy and asthma in children and young adults: a longitudinal study. *Lancet* 2002; 360(9337):901-907.

(11) Zutavern A, von Mutius E, Harris J, Mills P, Moffatt S, White C et al. The introduction of solids in relation to asthma and eczema. *Arch Dis Child* 2004; 89(4):303-308.

(12) Burr ML, Butland BK, King S, Vaughan-Williams E. Changes in asthma prevalence: two surveys 15 years apart. *Arch Dis Child* 1989; 64(10):1452-1456.

(13) COMA working group on the weaning diet. Weaning and the weaning diet. 1994. London.

(14) Grundy J, Matthews S, Bateman B, Dean T, Arshad SH. Rising prevalence of allergy to peanut in children: Data from 2 sequential cohorts. *J Allergy Clin Immunol* 2002; 110(5):784-789.

(15) Committee on Nutrition. Hypoallergenic Infant Formulas. *Pediatrics* 2000; 106(2):346-349.

(16) Greer FR, Sicherer SH, Burks AW. Effects of early nutritional interventions on the development of atopic disease in infants and children: the role of maternal dietary restriction, breastfeeding, timing of introduction of complementary foods, and hydrolyzed formulas. *Pediatrics* 2008; 121(1):183-191.

(17) Agostoni C, Decsi T, Fewtrell M, Goulet O, Kolacek S, Koletzko B et al. Complementary feeding: a commentary by the ESPGHAN Committee on Nutrition. *J Pediatr Gastroenterol Nutr* 2008; 46(1):99-110.

(18) Kramer MS, Kakuma R. Optimal duration of exclusive breastfeeding. *Cochrane Database Syst Rev* 2002;(1):CD003517.

(19) Falth-Magnusson K, Kjellman NI. Development of atopic disease in babies whose mothers were receiving exclusion diet during pregnancy--a randomized study. *J Allergy Clin Immunol* 1987; 80(6):868-875.

(20) Falth-Magnusson K, Kjellman NI. Allergy prevention by maternal elimination diet during late pregnancy--a 5-year follow-up of a randomized study. *J Allergy Clin Immunol* 1992; 89(3):709-713.

(21) Hattevig G, Kjellman B, Sigurs N, Grodzinsky E, Hed J, Bjorksten B. The effect of maternal avoidance of eggs, cow's milk, and fish during lactation on the development of IgE, IgG, and IgA antibodies in infants. *J Allergy Clin Immunol* 1990; 85(1 Pt 1):108-115.

(22) Herrmann M-E, Dannemann A, Grüters A, Radisch B, Dudenhausen JW, Bergmann R et al. Prospective study on the atopy preventive effect of maternal avoidance of milk and eggs during pregnancy and lactation. *European Journal of Pediatrics* 1996; 155(9):770-774.

(23) Zeiger RS, Heller S. The development and prediction of atopy in high-risk children: follow-up at age seven years in a prospective randomized study of combined maternal and infant food allergen avoidance. *J Allergy Clin Immunol* 1995; 95(6):1179-1190.

(24) Hide DW, Matthews S, Tariq S, Arshad SH. Allergen avoidance in infancy and allergy at 4 years of age. *Allergy* 1996; 51(2):89-93.

(25) Saarinen UM, Kajosaari M. Does dietary elimination in infancy prevent or only postpone a food allergy? A study of fish and citrus allergy in 375 children. *Lancet* 1980; 1(8161):166-167.

(26) Kajosaari M. Atopy prophylaxis in high-risk infants. Prospective 5-year follow-up study of children with six months exclusive breastfeeding and solid food elimination. *Adv Exp Med Biol* 1991; 310:453-458.

(27) Fergusson DM, Horwood LJ, Shannon FT. Early solid feeding and recurrent childhood eczema: a 10-year longitudinal study. *Pediatrics* 1990; 86(4):541-546.

(28) Mihrshahi S, Ampon R, Webb K, Almqvist C, Kemp AS, Hector D et al. The association between infant feeding practices and subsequent atopy among children with a family history of asthma. *Clin Exp Allergy* 2007; 37(5):671-679.

(29) Sariachvili MA, Droste JHJ, Dom S, Wieringa MH, Hagendorens MM, Stevens WJ et al. Early exposure to solid foods and the development of eczema in children up to 4 years of age. Pediatr Allergy Immunol. In press 2009.

(30) Poole JA, Barriga K, Leung DY, Hoffman M, Eisenbarth GS, Rewers M et al. Timing of initial exposure to cereal grains and the risk of wheat allergy. *Pediatrics* 2006; 117(6):2175-2182.

(31) Alm B, Aberg N, Erdes L, Mollborg P, Pettersson R, Norvenius SG et al. Early introduction of fish decreases the risk of eczema in infants. *Arch Dis Child* 2009; 94(1):11-15.

(32) Snijders BE, Thijs C, van RR, van den Brandt PA. Age at first introduction of cow milk products and other food products in relation to infant atopic manifestations in the first 2 years of life: the KOALA Birth Cohort Study. *Pediatrics* 2008; 122(1):e115-e122.

(33) Du Toit G., Katz Y, Sasieni P, Mesher D, Maleki SJ, Fisher HR et al. Early consumption of peanuts in infancy is associated with a low prevalence of peanut allergy. *J Allergy Clin Immunol* 2008; 122(5):984-991.

(34) Husby S, Mestecky J, Moldoveanu Z, Holland S, Elson CO. Oral tolerance in humans. T cell but not B cell tolerance after antigen feeding. *J Immunol* 1994; 152(9):4663-4670.

(35) Meglio P, Bartone E, Plantamura M, Arabito E, Giampietro PG. A protocol for oral desensitization in children with IgE-mediated cow's milk allergy. *Allergy* 2004; 59(9):980-987.

(36) Clark AT, Islam S, King Y, Deighton J, Anagnostou K, Ewan PW. Successful oral tolerance induction in severe peanut allergy. *Allergy* 2009.

(37) Kerosuo H, Kullaa A, Kerosuo E, Kanerva L, Hensten-Pettersen A. Nickel allergy in adolescents in relation to orthodontic treatment and piercing of ears. *Am J Orthod Dentofacial Orthop* 1996; 109(2):148-154.

(38) Mortz CG, Lauritsen JM, Bindslev-Jensen C, Andersen KE. Nickel sensitization in adolescents and association with ear piercing, use of dental braces and hand eczema. The Odense Adolescence Cohort Study on Atopic Diseases and Dermatitis (TOACS). *Acta Derm Venereol* 2002; 82(5):359-364.

(39) Van Hoogstraten IM, Andersen KE, Von Blomberg BM, Boden D, Bruynzeel DP, Burrows D et al. Reduced frequency of nickel allergy upon oral nickel contact at an early age. *Clin Exp Immunol* 1991; 85(3):441-445.

(40) Twarog FJ, Weinstein SF, Khaw KT, Strieder DJ, Colten HR. Hypersensitivity to pancreatic extracts in parents of patients with cystic fibrosis. *J Allergy Clin Immunol* 1977; 59(1):35-40.

(41) Strobel S. Dietary manipulation and induction of tolerance. *J Pediatr* 1992; 121(5 Pt 2):S74-S79.

(42) Friedman A. Induction of anergy in Th1 lymphocytes by oral tolerance. Importance of antigen dosage and frequency of feeding. *Ann N Y Acad Sci* 1996; 778:103-110.

(43) Frossard CP, Tropia L, Hauser C, Eigenmann PA. Lymphocytes in Peyer patches regulate clinical tolerance in a murine model of food allergy. *J Allergy Clin Immunol* 2004; 113(5):958-964.

(44) Strid J, Thomson M, Hourihane J, Kimber I, Strobel S. A novel model of sensitization and oral tolerance to peanut protein. *Immunology* 2004; 113(3):293-303.

(45) Quigley MA, Kelly YJ, Sacker A. Infant feeding, solid foods and hospitalisation in the first 8 months after birth. *Arch Dis Child* 2008.

(46) Bolling K, Grant C, Hamlyn B, Thornton A. Infant feeding survey 2005. 2005. The Information Centre for health and social care and the UK Health Departments.

(47) Davies DP. Plasma osmolality and feeding practices of healthy infants in first three months of life. *Br Med J* 1973; 2(5862):340-342.

(48) Taitz LS. Infantile overnutrition among artificially fed infants in the Sheffield region. *Br Med J* 1971; 1(5744):315-316.

(49) Hornell A, Hofvander Y, Kylberg E. Solids and formula: association with pattern and duration of breastfeeding. *Pediatrics* 2001; 107(3):E38.

(50) Nowak-Wegrzyn A, Bloom KA, Sicherer SH, Shreffler WG, Noone S, Wanich N et al. Tolerance to extensively heated milk in children with cow's milk allergy. *J Allergy Clin Immunol* 2008; 122(2):342-7, 347.

(51) Thomas K, Herouet-Guicheney C, Ladics G, Bannon G, Cockburn A, Crevel R et al. Evaluating the effect of food processing on the potential human allergenicity of novel proteins: international workshop report. *Food Chem Toxicol* 2007; 45(7):1116-1122.

(52) Kulig M, Bergmann R, Klettke U, Wahn V, Tacke U, Wahn U. Natural course of sensitization to food and inhalant allergens during the first 6 years of life. *J Allergy Clin Immunol* 1999; 103(6):1173-1179.

(53) Martinez FD, Wright AL, Taussig LM, Holberg CJ, Halonen M, Morgan WJ. Asthma and wheezing in the first six years of life. The Group Health Medical Associates. *N Engl J Med* 1995; 332(3):133-138.

(54) Sears MR, Greene JM, Willan AR, Wiecek EM, Taylor DR, Flannery EM et al. A longitudinal, population-based, cohort study of childhood asthma followed to adulthood. *N Engl J Med* 2003; 349(15):1414-1422.

(55) Illi S, von ME, Lau S, Niggemann B, Gruber C, Wahn U. Perennial allergen sensitisation early in life and chronic asthma in children: a birth cohort study. *Lancet* 2006; 368(9537):763-770.

(56) Morgan WJ, Stern DA, Sherrill DL, Guerra S, Holberg CJ, Guilbert TW et al. Outcome of asthma and wheezing in the first 6 years of life: follow-up through adolescence. *Am J Respir Crit Care Med* 2005; 172(10):1253-1258.

(57) Venter C, Pereira B, Grundy J, Clayton CB, Roberts G, Higgins B et al. Incidence of parentally reported and clinically diagnosed food hypersensitivity in the first year of life. *J Allergy Clin Immunol* 2006; 117(5):1118-1124.

(58) Nowak-Wegrzyn A, Assa'ad AH, Bahna SL, Bock SA, Sicherer SH, Teuber SS. Work Group report: oral food challenge testing. *J Allergy Clin Immunol* 2009; 123(6 Suppl):S365-S383.

(59) Eating Well for 1-4 Year Olds: Practical Guide and pack of printed Eating Well for 1-4 Year Olds: Food Photo Cards. 2010. Caroline Walker Trust.

(60) Wrieden WL, Longbottom PJ, Adamson AJ, Ogston SA, Payne A, Haleem MA et al. Estimation of typical food portion sizes for children of different ages in Great Britain. *Br J Nutr* 2008; 99(6):1344-1353.

(61) Infant and Toddler Forum. 24-3-2011. 24-3-2011.

(62) Caroline Walker Trust. 2011. 24-3-2011.

(63) Dr. Helen Crawley, from the Caroline Walker Trust. 8-3-2011.

(64) Dr. Carina Venter, BSc Dietetics, PG Dip Allergy, PhD, RD, between 28.02.11 - 20.03.11. 28-2-2011.

(65) Ms. Judy More BSc RD RN , between 11.03.11 - 18.03.11 (instrumental in the composite analysis of available data from CWT, NDNS, ALSPAC data and clinical experience to form the portion size determinations on the Infant and Toddler Forum website). 2011.

(66) Ms. Mary Feeney BSc, MSc, Pg Dip RD, Senior Research Dietitian, LEAP Study. 21-3-2011.

(67) Keil T, McBride D, Grimshaw K, Niggemann B, Xepapadaki P, Zannikos K et al. The multinational birth cohort of EuroPrevall: background, aims and methods. *Allergy* 2010; 65(4):482-490.

(68) Ms. Kate Grimshaw BSc(Hons), MSc, RD, R Nutr, between 27.02.11 - 07.03.11. 27-2-2011.

(69) Venter C, Pereira B, Voigt K, Grundy J, Clayton CB, Gant C et al. Comparison of open and double-blind placebo-controlled food challenges in diagnosis of food hypersensitivity amongst children. *J Hum Nutr Diet* 2007; 20(6):565-579.

(70) Ms. Mary Feeney BSc, MSc, Pg Dip RD, Senior Research Dietitian, LEAP Study. 21-3-2011.

(71) Dr Debra Palmer BSc BND PhD Child Nutrition Research Centre, Women's & Children's Health Research Institute, Adelaide, SA, between 18.03.11 - 20.03.11. 18-3-2011.

(72) Strachan DP. Hay fever, hygiene, and household size. *BMJ* 1989; 299(6710):1259-1260.

(73) Perkin MR, Strachan DP. Which aspects of the farming lifestyle explain the inverse association with childhood allergy? *J Allergy Clin Immunol* 2006; 117(6):1374-1381.

(74) Flohr C, Tuyen LN, Quinnell RJ, Lewis S, Minh TT, Campbell J et al. Reduced helminth burden increases allergen skin sensitization but not clinical allergy: a randomized, double-blind, placebo-controlled trial in Vietnam. *Clin Exp Allergy* 2010; 40(1):131-142.

(75) Lack G. Epidemiologic risks for food allergy. *J Allergy Clin Immunol* 2008; 121(6):1331-1336.

(76) Koplin J, Allen K, Gurrin L, Osborne N, Tang ML, Dharmage S. Is caesarean delivery associated with sensitization to food allergens and IgE-mediated food allergy: a systematic review. *Pediatr Allergy Immunol* 2008; 19(8):682-687.

(77) Palmer C, Bik EM, DiGiulio DB, Relman DA, Brown PO. Development of the human infant intestinal microbiota. *PLoS Biol* 2007; 5(7):e177.

(78) Koenig JE, Spor A, Scalfone N, Fricker AD, Stombaugh J, Knight R et al. Succession of microbial consortia in the developing infant gut microbiome. *Proc Natl Acad Sci U S A* 2011; 108 Suppl 1:4578-4585.

(79) Perkin MR. Unpasteurized milk: health or hazard? *Clin Exp Allergy* 2007; 37(5):627-630.

(80) Costello EK, Lauber CL, Hamady M, Fierer N, Gordon JI, Knight R. Bacterial community variation in human body habitats across space and time. *Science* 2009; 326(5960):1694-1697.

(81) Qin J, Li R, Raes J, Arumugam M, Burgdorf KS, Manichanh C et al. A human gut microbial gene catalogue established by metagenomic sequencing. *Nature* 2010; 464(7285):59-65.

(82) Chung H, Kasper DL. Microbiota-stimulated immune mechanisms to maintain gut homeostasis. *Curr Opin Immunol* 2010; 22(4):455-460.

(83) Bjorksten B, Sepp E, Julge K, Voor T, Mikelsaar M. Allergy development and the intestinal microflora during the first year of life. *J Allergy Clin Immunol* 2001; 108(4):516-520.

(84) Watanabe S, Narisawa Y, Arase S, Okamatsu H, Ikenaga T, Tajiri Y et al. Differences in fecal microflora between patients with atopic dermatitis and healthy control subjects. *J Allergy Clin Immunol* 2003; 111(3):587-591.

(85) Wang M, Karlsson C, Olsson C, Adlerberth I, Wold AE, Strachan DP et al. Reduced diversity in the early fecal microbiota of infants with atopic eczema. *J Allergy Clin Immunol* 2008; 121(1):129-134.

(86) Forno E, Onderdonk AB, McCracken J, Litonjua AA, Laskey D, Delaney ML et al. Diversity of the gut microbiota and eczema in early life. *Clin Mol Allergy* 2008; 6:11.

(87) Brown SJ, Asai Y, Cordell HJ, Campbell LE, Zhao Y, Liao H et al. Loss-of-function variants in the filaggrin gene are a significant risk factor for peanut allergy. *J Allergy Clin Immunol* 2011; 127(3):661-667.

(88) Flohr C, England K, Radulovic S, McLean WH, Campbel LE, Barker J et al. Filaggrin loss-of-function mutations are associated with early-onset eczema, eczema severity and transepidermal water loss at 3 months of age. *Br J Dermatol* 2010; 163(6):1333-1336.

(89) Maeda Y, Noda S, Tanaka K, Sawamura S, Aiba Y, Ishikawa H et al. The failure of oral tolerance induction is functionally coupled to the absence of T cells in Peyer's patches under germfree conditions. *Immunobiology* 2001; 204(4):442-457.

(90) Sudo N, Sawamura S, Tanaka K, Aiba Y, Kubo C, Koga Y. The requirement of intestinal bacterial flora for the development of an IgE production system fully susceptible to oral tolerance induction. *J Immunol* 1997; 159(4):1739-1745.

(91) Vael C, Desager K. The importance of the development of the intestinal microbiota in infancy. *Curr Opin Pediatr* 2009; 21(6):794-800.

(92) Ishikawa H, Tanaka K, Maeda Y, Aiba Y, Hata A, Tsuji NM et al. Effect of intestinal microbiota on the induction of regulatory CD25+ CD4+ T cells. *Clin Exp Immunol* 2008; 153(1):127-135.

(93) Adlerberth I, Strachan DP, Matricardi PM, Ahrne S, Orfei L, Aberg N et al. Gut microbiota and development of atopic eczema in 3 European birth cohorts. *J Allergy Clin Immunol* 2007; 120(2):343-350.

(94) Koenig JE, Spor A, Scalfone N, Fricker AD, Stombaugh J, Knight R et al. Succession of microbial consortia in the developing infant gut microbiome. *Proc Natl Acad Sci U S A* 2011; 108 Suppl 1:4578-4585.

(95) Adlerberth I, Jalil F, Carlsson B, Mellander L, Hanson LA, Larsson P et al. High turnover rate of Escherichia coli strains in the intestinal flora of infants in Pakistan. *Epidemiol Infect* 1998; 121(3):587-598.

(96) Thompson-Chagoyan OC, Fallani M, Maldonado J, Vieites JM, Khanna S, Edwards C et al. Faecal Microbiota and Short-Chain Fatty Acid Levels in Faeces from Infants with Cow's Milk Protein Allergy. *Int Arch Allergy Immunol* 2011; 156(3):325-332.

(97) Lack G, Fox D, Northstone K, Golding J. Factors associated with the development of peanut allergy in childhood. *N Engl J Med* 2003; 348(11):977-985.

(98) Sicherer SH, Burks AW, Sampson HA. Clinical features of acute allergic reactions to peanut and tree nuts in children. *Pediatrics* 1998; 102(1):e6.

(99) Sicherer SH, Furlong TJ, Munoz-Furlong A, Burks AW, Sampson HA. A voluntary registry for peanut and tree nut allergy: characteristics of the first 5149 registrants. *J Allergy Clin Immunol* 2001; 108(1):128-132.

(100) Norris JM, Barriga K, Hoffenberg EJ, Taki I, Miao D, Haas JE et al. Risk of celiac disease autoimmunity and timing of gluten introduction in the diet of infants at increased risk of disease. *JAMA* 2005; 293(19):2343-2351.

(101) Weiland SK, Bjorksten B, Brunekreef B, Cookson WO, von ME, Strachan DP. Phase II of the International Study of Asthma and Allergies in Childhood (ISAAC II): rationale and methods. *Eur Respir J* 2004; 24(3):406-412.

(102) Kunz B, et al. Clinical validation and guidelines for the SCORAD index: consensus report of the European Task Force on Atopic Dermatitis. *Dermatology* 1997; 195:10-19.

(103) Charman CR, Venn AJ, Williams HC. The patient-oriented eczema measure: development and initial validation of a new tool for measuring atopic eczema severity from the patients' perspective. *Arch Dermatol* 2004; 140(12):1513-1519.

(104) Sporik R, Hill DJ, Hosking CS. Specificity of allergen skin testing in predicting positive open food challenges to milk, egg and peanut in children. *Clin Exp Allergy* 2000; 30(11):1540-1546.

(105) Hill DJ, Heine RG, Hosking CS. The diagnostic value of skin prick testing in children with food allergy. *Pediatr Allergy Immunol* 2004; 15(5):435-441.

(106) Strippoli MP, Silverman M, Michel G, Kuehni CE. A parent-completed respiratory questionnaire for 1-year-old children: repeatability. *Arch Dis Child* 2007; 92(10):861-865.

(107) WHO. Indicators for assessing infant and young child feeding practices: conclusions of a consensus meeting held 6-8 November 2007 in Washington D.C., USA. 2008. WHO Press.

(108) Nikolovski J, Stamatas GN, Kollias N, Wiegand BC. Barrier function and water-holding and transport properties of infant stratum corneum are different from adult and continue to develop through the first year of life. *J Invest Dermatol* 2008; 128(7):1728-1736.

(109) Lago P, Garetti E, Merazzi D, Pieragostini L, Ancora G, Pirelli A et al. Guidelines for procedural pain in the newborn. *Acta Paediatr* 2009; 98(6):932-939.

(110) Sicherer SH, Eigenmann PA, Sampson HA. Clinical features of food protein-induced enterocolitis syndrome. *J Pediatr* 1998; 133(2):214-219.

(111) Department of Health. Research governance framework for health and social care (2nd edtn). 2005.

Appendix 1. Study Overview Chart – *post contract variation September 2010*

EAT (*Enquiring About Tolerance*) Study Overview

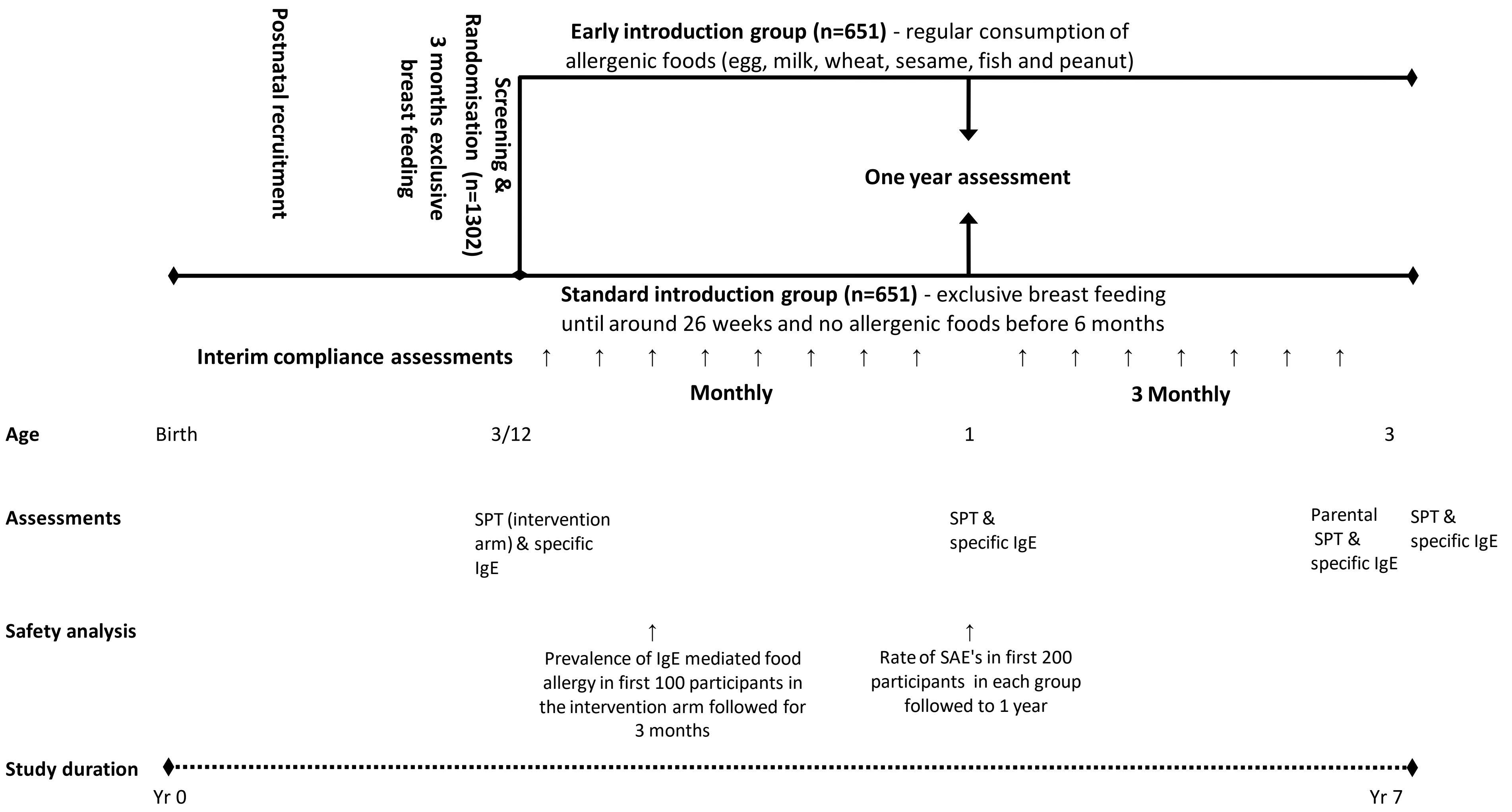

Appendix 2. Study Gantt Chart – *post contract variation September 2010*

Gantt chart: T07051 Randomized controlled trial of early introduction of allergenic foods to induce tolerance in infants

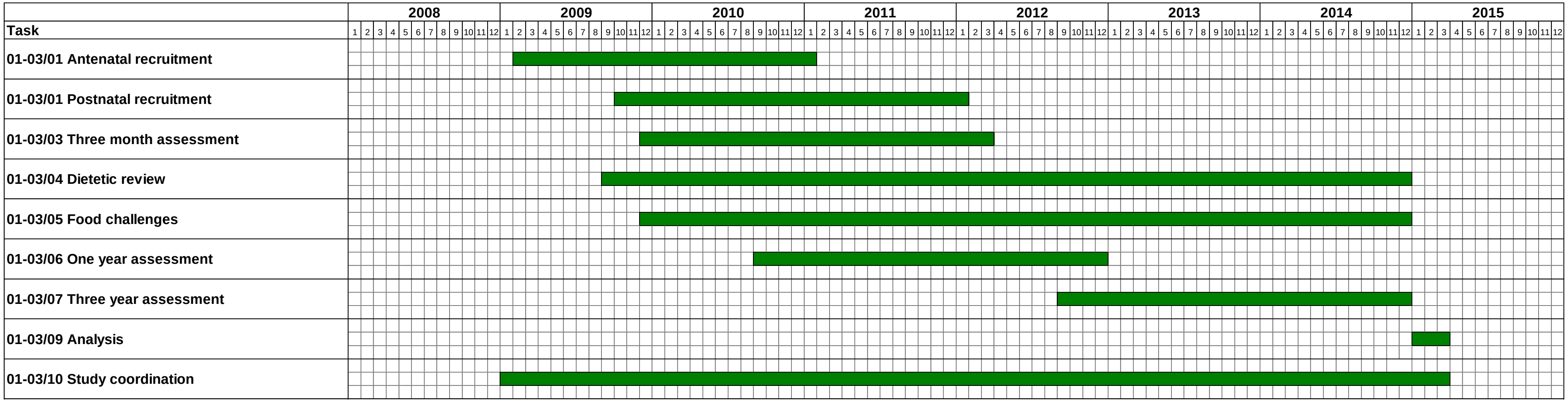

Appendix 3. Schedule of Events

Age	12/40	20/40	34/40	0	3/12 Initial assessment	4-12/12 Monthly	One year assessment	15-36/12 3 Monthly	36/12 Final assessment	Unscheduled visit assessment
Invitation & information (antenatal)	X									
Informed consent (antenatal recruitment)		Mother			Infant					
Informed consent (postnatal recruitment)					Infant					
Informed consent (parental recruitment)					Mother				Father	
General Assessments										
Medical history		X	X		X	X	X	X	X	X
Adverse events						X	X	X	X	X
Concomitant medications			X		X	X	X	X	X	X
TEWL measurement					X		X		X	
Eczema evaluation					X	X	X	X	X	
Rhinitis evaluation						X		X		
Asthma evaluation						X		X		
Anthropometry					X		X		X	
Blood pressure									X	
Skin swab					X		X			
Laboratory assessments										
Haematology					X		X		X	
Serum chemistries					X		X		X	
Serum lipids					X				X	
IgA tissue transglutaminase									X	
Glycosylated haemoglobin									X	
Allergy assessments										
Skin prick testing (parental)									Parental	
Specific IgE (parental)									Parental	
Skin prick testing (child)					Intervention foods*		Extended panel†		Extended panel‡	X
Specific IgE (child)					Intervention foods		Extended panel§		Extended panel§	X
Diet										
Dietary education					X		X		X	X
FFQ		Maternal	Maternal		Maternal					
5 day food diary						6/12	X		X	
Food reaction history						X	X	X	X	X
Compliance assessment						X	X	X	X	X
Immunologic assessments *(subject to funding)*										
Frozen PBMC T-cell assay				X	X		X		X	
Plasma allergen specific IgG and IgE				X	X		X		X	
Whole blood DNA-HLA haplotypes				X	X				X	

* Intervention arm only

† Intervention foods and soya and kiwi. Aero-allergens: house dust mite, cat, dog, 6 grass pollen mix and 3 tree pollen mix

‡ As per 1 year visit. Also tree nuts: Brazil nut, hazel nut, cashew, almond and walnut; and raw egg white

§ Intervention foods and an aeroallergen panel including: D. pteronyssinus, D. farinae, cat and dog dander, horse dander, timothy grass, cladosporum, silver birch, olive, mugwort and nettle
