# Supplementary material for: Association of Early Introduction of Solids With Infant Sleep: A Secondary Analysis of a Randomized Clinical Trial
Source: JAMA Pediatr. 2018 Jul 9;172(8):e180739. doi: 10.1001/jamapediatrics.2018.0739 (PMC6142923; doi:10.1001/jamapediatrics.2018.0739)
Supplement: Supplement 4. — Trial Protocol. Summary of changes to EAT protocol. [file jamapediatr-172-e180739-s004.pdf]

<u>EAT Study Protocol (T07051) – Summary of Changes</u>

Substantial amendment	IRAS data	Protocol number	Notes
Initial submission	02/06/2008	Scope of work document sent	Approved 20/10/08
-	-	1.00 (13/05/08)	Incomplete draft protocol
-	-	1.01 (31/08/08)	Complete draft protocol
	06/02/2009		ISRCTN registered: 14254740
1	03/04/2009	1.02 (01/04/09)	First full protocol
2	14/08/2009	1.03 (14/08/09)	
3	01/10/2010	2.03 (01/10/10)	
4	01/08/2011	3.00 (01/08/11)	
5	01/08/2012	4.00 (01/08/12)	
6	19/03/2014	5.00 (19/03/14)	Current protocol

SA1 Summary of changes

1. Substituting sesame for soya

We have substituted sesame for soya in the foods to be introduced into the infants diet. This is because emerging data from the EuroPrevall study confirms that sesame is a significantly greater cause of IgE mediated food allergy than soya. Early soya introduction also raised the issue of phyto-oestrogen exposure and excluding soya from the six foods avoids this issue.

2. Three month assessment

We are only skin prick testing the intervention arm children for the 6 intervention foods. The rationale is that we want the control mothers to wean their children in the same way that they would have done if they were not participating in the study. If we were to skin prick test the control children to the 6 intervention foods at 3 months of age this would be very likely to have a significant effect on the way a mother weaned her infant.

3. One year assessment

We are going to invite all participants back at one year of age for assessment rather than a high risk sub-group. This overcomes the issue that there would have been some infants in the control arm who might have had transient milk or egg allergy that never had an opportunity to manifest before they attended their 3 year assessment. By skin prick testing all infants at the one year assessment a level playing field is generated with children in both arms of the study having an identical chance of their allergies being diagnosed from one year onwards. Any child with a positive skin prick test to an intervention food at the 1 year (and 3 year) assessments will be invited to undergo a double blind placebo controlled food challenge.

4. Food frequency questionnaire

Whilst the maternal FFQ that we administer is based on validated tools, no such validated FFQ exists for the compliance assessments that take place monthly between 3 months and 1 year of age and 3 monthly until 3 years of age. We have generated a new FFQ based on the EuroPrevall food diaries completed by mothers of infants of the same age. We now wish to undertake a validation study comparing this with food diaries. We will recruit 50 families with infants between 6 months and 1 year of age from St Thomas'. Mothers will complete the FFQ covering the last 2 weeks and then an 8 day food diary completed over a 2 week period. Both the maternal questionnaire and the new FFQ are attached with this application.

5. Background to study

We have updated the lay summary to take into account some new papers and data that have been published since the original submission.

6. Mother's information/consent sheet

The main change to this has been to reformat the section on the current DH guidelines and the undertaking our study which differs from the DH weaning guidelines. Based on feedback from mothers we have laid this out in the form of a DH statement followed by a paragraph explaining our rationale. Mothers have found this to be much more helpful.

7. Errata in the information/consent sheets

We have corrected some errors that had been noted e.g. the insurance company name, the telephone number for the study etc.

8. Research sites

Two of the potential research sites were erroneously combined into one name on the original application "Queen Charlotte's and Chelsea Hospital". This should of course have been "Queen Charlotte's Hospital" and the "Chelsea and Westminster Hospital".

9. GP information sheet

We have also attached the GP information sheet that we are using.

10. Primary and secondary endpoints

Because all participants have an identical chance of their food allergy being diagnosed from one year onwards the primary endpoint has been refined to "The period prevalence of IgE mediated food allergy to the six intervention foods between one and three years of age in both arms." Secondary endpoints have also been revised to include both cumulative criteria and period criteria (between one and three years of age).

11. Staff information leaflet

We have also produced a summary page for staff in the recruitment centres to explain the background to the project.

12. Introduction of "other" allergenic foods

The information leaflet and consent forms state that baby rice and then cow's milk based yoghurt will be introduced and then the other allergenic foods will be introduced from 4 months of age. However, this should state that the other allergenic foods will be subsequently introduced rather than the overly proscriptive threshold of 4 months as infants having successfully being commenced on the baby rice and cow's milk yoghurt will be able to introduce the other allergenic foods after this.

13. Government peanut consumption recommendations

The committee that issued the government with the advice on avoiding peanut consumption in pregnancy, during lactation and during the first 3 years of life for high risk infants met before Christmas and has made a submission to the Department of Health that the guidance be withdrawn. Our amended information sheets with this submission reflect the current advice that peanuts should be avoided in high risk families but this advice will be withdrawn in the next couple of months. We therefore request that we be given permission to remove the sections in the information leaflet and consent form about avoiding peanut once the DH officially agrees with the committee's recommendation and withdraws its peanut advice.

SA2 Summary of changes

(1) Utilising back up recruitment plan

Our original application included details of utilising direct mail outs to pregnant mothers using the Bounty scheme. The LEAP Study, based at St Thomas', has successfully used Bounty mailouts to achieve its recruitment target.

Our original application stated pregnant mothers and this should have stated simply mothers as both antenatal and postnatal mothers can be mailed. Recruitment at the two antenatal centres has fallen short of expected and therefore rather than adding in further recruitment centres (up to four additional centres were listed in the original submission), we will use the Bounty scheme instead to send mail outs to mothers with young infants (5-10 weeks old) in the London and surrounding areas. The changes made to the IRAS form have been simply to clarify where necessary what this thus entails (e.g. to obtaining consent).

We also attach with this application a copy of the flyer that we will be using. This is based on the successful LEAP flyer. This has been approved for use by the Department of Health.

EAT SA3 Summary of changes

1. Revision of cohort size required

It has become apparent that we are recruiting a higher risk cohort than the general paediatric population. A meeting to discuss the implications of this were held with our funders in February this year and we reassessed the sample size required in order to see if the early introduction of allergenic foods reduces allergy. A contract variation with the funders was agreed in September. Based on the current recruitment rate, a further four months recruitment will be required which was agreed in the contract variation. The scientific rationale for the amended sample size calculation is contained in the document: "EAT Study - supporting scientific information for reduced recruitment target".

IRAS sections amended in relation to this: A6-1. Summary of study

Study protocol section: A new section 3.1.13 Bounty Recruitment (Contract Variation September 2010) has been incorporated explaining the rationale for the change in recruitment target. Other sections amended: Section 1.3.9, 2.1, 3.6, 3.11, 9.3, 9.4 & synopsis

2. Removal of the word "weaning" from our material

The word "weaning" is confusing. It means cessation of breastfeeding to some individuals and introduction of complimentary foods to others. This confusion has impacted on mothers considering participating in the study. In order to remove this confusion we have sought to clarify things by introducing a harmonized terminology for our two groups. The group introducing the allergenic foods early is being termed the "early introduction group". The group following the government's standard infant feeding advice is being termed the "standard introduction group". This has the benefit of making it clear that the study is, in essence, a trial of **timing** of introduction of the allergenic foods.

IRAS sections amended in relation to this: A6-1. Summary of study; A6-2. Summary of main issues;

Study protocol section: Section 3.9.2 amended to explain rationale for avoiding the term "weaning". Other sections amended: wording changed throughout protocol to these terms

3. Additional exclusion criteria

Three additional exclusion criteria have been added.

(i) Significant postnatal health concerns.

The protocol previously only mentioned significant congenital disease and not significant postnatal health problems that may have emerged which would preclude an infant participating in the study.

(ii) Any history of stridor irrespective of cause

This has been included in light of the SAE (reported to the LREC on 6th July 2010 (ID: 497857R) where an infant with a history of stridor developed an urticarial reaction with cow's milk and was given IM adrenaline because of his audible stridor. However, the stridor was not related to the reaction to

milk. In light of this it was agreed that any history of stridor should be an exclusion criterion for taking part in the EAT study.

(iii) Infant participating in another study prior to enrolment

This has been included in light of the recent incident where a mother participating in a study with a neonatal unit at another hospital mistakenly believed that unit had supplied her details to our study to invite her to take part. It was decided that participation in another study should be an exclusion criterion for participation in the EAT study.

IRAS sections amended in relation to this: A6-2. Summary of main issues;

Study protocol section: 4.2 amended.

4. Food challenges – frequency of consumption definitions

We have clarified what constitutes frequent versus infrequent consumers with regards to the number of doses they have to have for a food challenge in the protocol and how much food protein should be consumed for each dose.

Study protocol section: 3.6 amended. 6.6.5.3.1 amended

5. Stopping rules

In light of the lower number of participants required, the EAT IDMC suggested that the stopping rule for assessing allergy prevalence in the early introduction arm was changed from being when 200 participants had been followed up for 3 months to when 100 participants had been followed up for 3 months.

Study protocol section: 3.10.2.1 amended.

6. EAT intervention food quantities

The amount of foods that makes up 4grams of food protein was clarified in the protocol.

Study protocol section: 5.1 amended.

7. Analgesia for phlebotomy

Protocol clarified to indicate that at 3 month visit, breastfeeding will be used concurrently with the phlebotomy given the evidence for its analgesic effect.

Study protocol section: 6.4 amended.

8. Skin prick tests

Clarification that if histamine responses are negative they will be repeated (not <3mm). Histamine responses are known to be smaller particularly in young infants.

Study protocol section: 6.5.2 amended.

9. Food challenge supervision

Following a query raised by the EAT IDMC and subsequent correspondence with the St Thomas' LREC, the protocol has been amended to clarify the staff cover arrangements during food challenges.

Study protocol section: 6.6.5.1 amended.

10. Adverse event reporting

Clarification that defining what is expected versus an unexpected adverse event in a nutritional interventional study such as EAT is not clear, hence **all** SAEs will be reported within 15 days to the LREC (and the study sponsors, funders, TSC chair and IDMC chair)

Study protocol section: 8.3.3, 8.6.1 and 8.6.2 amended.

11. Visit windows

The study recruitment having extended to involve inviting mothers from throughout England and Wales has had a significant effect on the ability to maintain visit windows. Adverse weather etc makes it very difficult to rapidly rebook in families who are travelling from distant parts of the country and who need to have sufficient time to prepare to make a trip to London. We therefore propose increasing the 3 month visit window to +4 weeks and the 1 year visit window to +3 months to give families more flexibility.

Study protocol section: 6.1 amended.

12. Data monitoring

The protocol erroneously stated that 10% of CRFs would be audited at EAT Steering Committee meetings. This was meant to state that a random sample of 10 would be audited. 10% when it was planned to have 2500 participants would have been impossible to review.

Study protocol section: 11.2 amended.

EAT SA4 Summary of changes

1. Revision of quantity of food offered to children having food challenges

An audit of the initial children attending for their food challenges has shown a significant number were struggling to complete the challenge related to the quantity of food they were having to consume. The EAT dieticians have therefore carried out an extensive review of portion size and challenge practice in the UK and elsewhere to help guide what we should be giving in our challenges. The conclusion of this is that the amount we are offering is indeed too great and we have reduced it to come into line with the limited data on what are the accepted portion sizes for 1 year olds and 3 year olds. In order to reduce the total quantity of food that the participants are being asked to expect the double blind challenges have had the number of blinded doses reduced from 7 (4 active, 3 placebo) to 5 (3 active, 2 placebo). All infants having open challenges under one year of age will be offered a total of 2 grams of food protein (a standard EAT portion).

Study protocol section: A new section 1.3.10 Amendment to EAT food challenge protocols (June 2011) has been incorporated explaining the rationale for the change. Other sections amended: Section 6.6.7.1, 6.6.7.3.2,

2. Revision of type of food challenge for frequent consuming children

In the original protocol, children who attended the one and three year visits with any level of sensitisation on skin prick testing to any of the six intervention foods were supposed to undergo a double blind food challenge to the food with two hours observation afterwards. This is appropriate for non-consuming or infrequently consuming children. However, for children frequently consuming the food this is not proving to be tenable as a practice. The EAT participants coming from throughout England and Wales and challenges already often have to be scheduled for a separate appointment as many parents to not have enough time at the one and three year scheduled visits to stay for a further four hours to undergo a food challenge to a food their child eats on a regular basis without any clinical problems. Persuading such parents to make a long journey back to the unit to undertake the challenge is also proving difficult. Hence we propose offering frequently consuming participants who are found to be sensitised to the food an open cumulative (one single dose) challenge with one hours observation afterwards. In the extremely unlikely situation that they have a reaction during this challenge they would then be brought back for a double blind challenge.

IRAS sections amended in relation to this: A13.

Study protocol section: Sections amended: 6.6.7.3.1,

3. Other food challenge clarifications

A number of clarifications have been made pertaining to food challenges:

- Infants returning for their food challenge after a positive skin prick test at the 3 month visit will have repeat testing to the same allergens as used before, but also commercial solutions to sesame, milk and egg where applicable. (Protocol section 3.2)

- A minimum time interval will be allowed to elapse before consideration is given to a further challenge (Protocol section 3.5, 6.6.4)
- Certain participants will be challenged to peanut and/or sesame at the one year visit (Protocol sections 3.5, 3.6, Table 16)
- The challenge sequence for participants sensitized to multiple foods has been clarified (Protocol section 6.6.6)
- The definition of what constitutes a high risk participant has been clarified (Protocol section 6.6.7)
- Infants having an indeterminate open challenge under one year of age will return for an incremental open challenge rather than a double blind challenge (Protocol section 6.6.7.1).
- Infants whose parents report an acute food reaction but whose skin prick test at their unscheduled clinic visit is negative will be offered a cumulative open challenge (single dose of 2 grams of food protein) (Protocol section 6.6.7.2)

IRAS sections amended in relation to these clarifications: A6-2, A19.

4. Proposal to collect stool samples from EAT Study participants

There is an accumulating body of evidence that bowel flora plays a significant role in determining subsequent risk of atopy including food allergy. We therefore think it would be a very useful addition to collect a stool sample from EAT participants. Existing participants who have not yet had their one year visit would be asked to bring a dirty nappy to their one year visit and a sample collected by the study team from infants with eczema and/or food allergy/sensitisation at the visit. New participants would be asked to bring a dirty nappy to the 3 month and 1 year visits where samples would be taken. They would also be given a kit to collect a sample from a dirty nappy at 5 months of age which can be posted back to the study team. Full details are included in the protocol.

IRAS sections amended in relation to this: A6-2, A19, A22, Part B Section 5: 1, 2 & 9.

Study protocol section: A new section 1.5 Rationale for Collection of Stool Samples (August 2011) has been incorporated explaining the rationale for the change.

5. Additional exclusion criterion

One additional exclusion criterion has been added.

(i) Significant Vitamin D deficiency (Alkaline phosphatase at screening visit >1000)

The protocol previously only mentioned significant congenital disease and not significant postnatal health problems that may have emerged which would preclude an infant participating in the study.

IRAS sections amended in relation to this: A6-2, A17-2.

Study protocol section: 4.2 amended.

6. Additional laboratory assessments

Two laboratory assessments were excluded from the original protocol:

- The obtaining of skin swabs at the three visits was omitted on the original submission (but present on the LEAP protocol on which the EAT protocol was based).

- A coeliac screen measuring IgA tissue transglutaminase antibody will be undertaken at 3 years of age.

IRAS sections amended in relation to this: A19,A22, Part B Section 5: 1, 2 & 9.

Study protocol section: 6.4 amended.

7. Procedure for suspected coeliac disease

As section has been added to the protocol to clarify when a participant would be investigated for suspected coeliac disease.

Study protocol section: New section 6.6.9 incorporated.

8. Procedure for suspected food protein induced enterocolitis syndrome (FPIES) reactions

As section has been added to the protocol to clarify when a participant would be investigated for FPIES.

Study protocol section: New section 6.6.10 incorporated.

9. Clarification of which team members constitute the project management group

The PMG will be chaired by Dr Michael Perkin and include Professor Lack and the study manager, *and all EAT staff members present that day*.

Study protocol section: Section 11.1.2 amended

EAT SA5 Summary of changes

1. Funders contact details

We have updated the details of the contact staff of our two Funders (the Food Standards Agency and the Medical Research Council).

IRAS sections amended in relation to this: A13

Study protocol section: Title page

2. Participants final allergy status

We have clarified how participants' final allergy status is designated in a new Figure (Figure 10).

Study protocol section: Sections amended: 6.6.7.3.1

3. Raw egg white skin prick test at three year visit

We have included raw egg white in the panel of allergens that we skin prick test the children for at the three year visit. We tested the early introduction group infants with this at the three month visit but think it is important that we know both how this has evolved for those children but also in the control group children.

Study protocol section: Sections amended: 6.5.1.1

4. Parental consents forms update

We have ethical approval from the LREC to skin prick test and take blood from the parents at the three year visit based on the IRAS form completed. However we omitted details about skin prick testing the parents from the consent form that was signed by the mother when the EAT participant was enrolled. We have therefore produced two consent forms, one for the father (to consent to have both the skin prick testing and the blood test) and one for the mother (to consent for the skin prick testing). These will be signed by the parents when they attend with the three year assessment visit.

IRAS sections amended in relation to this: A6.2

Study protocol section: Sections amended: 6.6.7.3.1 & Appendix 3. Schedule of Events

5. Blood pressure

We propose (subject to the approval being confirmed by our own trial steering committee) measuring the children's blood pressure when they attend the three year visit. This is to ensure that the early introduction of the foods has had no effect on early blood pressure.

IRAS sections amended in relation to this: A19

Study protocol section: Sections amended: 6.2 & Appendix 3. Schedule of Events

6. Newsletters

We would like to keep parents informed about the results of the study by sending them newsletters.

IRAS sections amended in relation to this: A53

7. Three year visit window

We propose extending the visit window for the three year assessment to +/- six months instead of +/- three months. The change to postnatal recruitment using Bounty meant that our participants now come from across the length and breadth of England and Wales. Coordinating their final visits in conjunction with mothers being back at work and having to make a large journey for the final visit means that some mothers are finding it difficult to offer dates within our designated visit window.

Study protocol section: Sections amended: 6.1

8. Other food challenge clarifications

A number of clarifications have been made pertaining to food challenges:

- Which participants will not undergo sesame/peanut challenges at the one year visit but be deferred until the three year visit. (Protocol section 3.6)
- Scheduled challenges regime clarified. (Protocol section 6.6.1 & Table 16)
- Challenges for non-intervention foods section clarified (Protocol section 6.6.5)
- Repeat does section clarified (Protocol section 6.6.7.3.2)
- Additional low dose introduced for double blind challenge regime for significantly sensitised children based on LEAP study experience (Protocol section 6.6.7.3.2).

9. EAT Steering committee

Membership of the committee and member details has been updated.

Study protocol section: Section 11.1.2 amended

10. Sample size

The sample size section was not written as clearly as it could be and has been split into two sections for clarity.

Study protocol section: Section 9.3, now divided into 9.3.1 and 9.3.2

EAT SA6 Summary of changes

1. Three year visit window

In our previous substantial amendment (SA5) we extended the visit window for the three year visit to +/- six months. However it has become clear that for the same reasons given previously (quoted in italics below), as well as there being families who have emigrated but are willing to attend the final visit during returns to the UK, that the visit window needs to be extended to + twelve months. We will allow participants back after this window but they will not contribute to the primary analysis.

"The change to postnatal recruitment using Bounty meant that our participants now come from across the length and breadth of England and Wales. Coordinating their final visits in conjunction with mothers being back at work and having to make a large journey for the final visit means that some mothers are finding it difficult to offer dates within our designated visit window."

Study protocol section: Sections amended: 6.1 Visit windows

2. Home visits

For a small minority of mothers we are proposing offering home visits for the final 3 year visit in the EAT study for families who are finding it difficult to commit to coming back to St Thomas' hospital. The reason for this is that the study drop out rate is higher than expected at the 3 year visit primarily because EAT children come from a wide area of the UK. In the majority of cases, both parents are back in paid employment. This has resulted in approximately 20% of children not attending their 3 year visit to date, and consequently if the loss to follow up continues, the study may be underpowered to determine its primary endpoint. The study protocol allows for a 15% drop out.

We would propose to carry out exactly the same assessments at home that are carried out during the 3 year visit on our clinical trials unit at King's College London, including anthropometric measurements, skin prick testing and a blood test. The participant will be visited by a study nurse or study doctor.

The advantage of doing this is that this will make study participation easier for patients who live far away, will increase the retention rate of the study and maintain its power and ability to determine the best strategy for infant nutrition.

The home visit provides a safe environment in which to do this and has been done successfully using the same assessments and testing methods in the LEAP cohort of allergic children that enrolled 640 children with severe eczema and/or egg allergy between the ages of 4-11 months, following them up to the age of 60 months. The LEAP study has successful experience with home visits, both done as part of the study protocol at 21 months of age where 95% of families consented to a home visit, and those done at 5 years of age where families were not willing to attend their final follow up appointment. Children underwent skin prick testing at home and also had blood taken. It should be noted that the LEAP study population is a much higher risk population that is more atopic, with more than 50% presenting with a food allergy at enrolment into the study. There were no safety or other issues regarding these visits. Thus drawing from the LEAP team experience of home visits, we propose instigating EAT home visits.

IRAS sections previously amended in relation to this: A13, A19, A22

Study protocol section: Sections amended: 6.2 General assessments

3. Communication with parents

In our previous Substantial Amendment we secured permission to keep parents informed about the results of the study by sending them newsletters. This was amended in IRAS section A53. We have now amended the protocol to reflect this approval by renaming Section 13 "Publication Policy" of the protocol to "Communication Policy" and have included a new section 13.2 "Communication with Participants".

IRAS section previously amended (in SA5) in relation to this: A53

Study protocol section: Section 13.2 included

4. EAT Steering committee

Membership of the committee and member details has been updated.

Study protocol section: Section 11.1.1 amended

5. FPIES challenges

We have added a table to the protocol to accompany section 6.6.10 on FPIES challenges in order to have a simple summary of this section.

Study protocol section: Table 18 added.

6. Glycosylated haemoglobin

With the 3 year old children coming for their final three year visit from across England and Wales it was not realistic or feasible for them to be fasted prior to attending in order for us to measure fasting insulin and glucose. Instead we are measuring their random glycosylated haemoglobin.

Study protocol section: Section 6.4 & Appendix 3 amended
