# Supplementary material for: Association of Early Introduction of Solids With Infant Sleep: A Secondary Analysis of a Randomized Clinical Trial
Source: JAMA Pediatr. 2018 Jul 9;172(8):e180739. doi: 10.1001/jamapediatrics.2018.0739 (PMC6142923; doi:10.1001/jamapediatrics.2018.0739)
Supplement: Supplement 5. — Trial Protocol. Original SAP. [file jamapediatr-172-e180739-s005.pdf]

Enquiring About Tolerance (EAT) Study

Randomised controlled trial of early introduction of allergenic foods to induce tolerance in infants

Final version 20/08/2012

STATISTICAL ANALYSIS PLAN FOR MAIN PAPER

This document has been edited by Janet Peacock with substantial input from Salma Ayis, Michael Perkin, Kirsty Logan, and Gideon Lack. This version has been reviewed and approved by the EAT study steering committee.

The document is designed to provide a record of and guide to all pre-planned analyses that will be included in the first and main outcome paper of the EAT study.

It is not intended to include the additional analyses that may be performed, either as pre-planned or as a result of hypotheses generated by the primary analyses and that will go in subsequent publications. These will be covered in a separate document.

1. TRIAL DESIGN:

This is a randomized controlled trial of the early introduction of allergenic foods (and other foods) from three months of age in the general population with target accrual of 1302 participants. Mothers were recruited initially antenatally from two large antenatal units (Kingston and St Thomas' Hospitals) (antenatal recruitment ceased November 2009) and subsequently postnatally using invitations mailed to parents who are members of the Bounty Parenting Club.

All infants will be followed up until three years of age by which point the impact of the intervention on the primary outcome (food allergy) and secondary outcomes - asthma (including atopic wheeze), eczema, allergic rhinitis (including aero-allergen sensitization), combined food allergy prevalence (including food sensitization) and the prevalence of combined allergic disease will be known. As described in the EAT Protocol, Figure 5, Overall Study Design

2. TRIAL OBJECTIVES:

To determine whether early introduction of allergenic foods (from three months of age) reduces the prevalence of food allergy (FA), eczema, asthma and allergic rhinitis (AR) by three years of age. This study will provide an informed basis for future infant feeding practices in both atopic and normal infants.

3. TRIAL ARMS

3.1 Early introduction group: Dietetic controlled introduction of allergenic foods from three months of age. A pureed fruit / vegetable or baby rice mixed with breast milk or water commenced first, followed by cow's milk based yoghurt. Subsequently egg, sesame, fish, and peanut introduced in a randomized order, with wheat introduced last. (For further details on ingestion timing and quantities for each food, see protocol).

3.2 Standard introduction group: Standard introduction following current UK Government infant feeding advice (exclusive breastfeeding until around 26 weeks of age) and avoid introducing foods that may cause allergies before six months of age (cow's milk, egg, wheat, peanuts, tree nuts, seeds, fish and shell fish).

4.0 SAMPLE SIZE AND POWER

The trial has 80% power to detect a 50% relative reduction in the absolute prevalence (from 8% in the standard introduction arm to 4% in the intervention arm) of food allergy by three years of age, assuming a 15%

drop out rate. These numbers were used to calculate the final cohort size of 1302 infants (651 infants in each arm) yielding a final cohort size of 1106 infants (553 infants in each arm) after drop out (based on the amended protocol September 2010). The whole cohort is to be followed up until the primary assessment point at three years of age.

The study is also designed so that it has sufficient power to detect a protective effect in the high risk sub-population. The high risk children are defined as being the 25% with eczema present at three months of age, who have an estimated prevalence of food allergy of 30%. It is estimated that with 15% loss to follow up, there will be 276 high risk children completing the study, 138 in each group. The study has 99% power to detect a reduction from 30% to 10% amongst the high risk participants in the intervention group and 85% to detect a reduction from 30% to 15%. See protocol (Protocol section 9.3: Contract variation September 2010). The study is not powered to detect a reduction in allergy to individual foods.

5. LOSS TO FOLLOW UP

1302 infants will be recruited into the study at three months of age. A drop out figure of 15% has been estimated based on the low rate observed in the LEAP study and in the EAT study at the time of the revised protocol. (Protocol change September 2010).

6. RANDOMIZATION AND BLINDING

Participants are randomly assigned to treatment using a centrally administered randomization service (ALEA). The randomization is not stratified given the number of participants. Given the nature of the intervention, the study participants cannot be blinded to their allocation group. However, the study outcomes are based on food challenges that include double-blinded assessment and objective measures of sensitization (skin prick test results) and specific IgE measurements. The latter are measured by laboratory staff blinded to the child's allocation status**.**

7. ENDPOINTS

7.1 Primary Endpoint

The period prevalence of IgE mediated food allergy to the six intervention foods between one and three years of age in both arms.

7.2 Secondary Endpoints

7.2.1 Period (one to three years of age) prevalence food outcomes

1. The period prevalence of all IgE mediated food allergy between one and three years of age in both arms.
2. The period prevalence of all reported food allergy (IgE and non-IgE mediated) between one and three years of age in both arms.
3. The period prevalence of sensitization to food between one and three years of age in both arms. (Defined in Protocol, Section 3.9).
4. The total number of foods (of the six intervention foods) to which IgE mediated food allergy has been diagnosed between one and three years of age in both arms amongst children with IgE mediated allergy to one or more of the six intervention foods.
5. The total number of foods (any foods) to which IgE mediated food allergy has been diagnosed between one and three years of age in both arms amongst children with IgE mediated allergy to one or more foods.

7.2.2 Cumulative (by three years of age) prevalence food outcomes

1. The cumulative prevalence of IgE mediated food allergy to the six intervention foods by three years of age.
2. The cumulative prevalence of all IgE mediated food allergy by three years of age.
3. The cumulative prevalence of all food allergy (IgE and non-IgE mediated) by three years of age.
4. The cumulative prevalence of non-IgE mediated food allergy by three years of age.
5. The cumulative prevalence of sensitization (defined in Section 3.9) to the six foods by three years of age.
6. The total number of foods (of the six intervention foods) to which IgE mediated food allergy has been diagnosed by three years of age amongst children with IgE mediated allergy to one or more of the six intervention foods.
7. The total number of foods (any foods) to which IgE mediated food allergy has been diagnosed by three years of age amongst children with IgE mediated allergy to one or more foods.

7.2.3 Point prevalence food outcomes

1. The point prevalence of IgE mediated food allergy to the intervention foods (excluding sesame and peanut) at the one year assessment.
2. The point prevalence of IgE mediated food allergy to the intervention foods at the three year assessment.

7.2.4 Other allergic disease outcomes

1. The point prevalence of eczema at one year and three years of age and cumulative prevalence of eczema by three years of age.
2. The severity of eczema at one year and three years of age.
3. The prevalence of allergic rhinitis at three years of age.
4. The prevalence of inhalant allergen sensitization at one year and at three years of age by skin prick test.
5. The prevalence of inhalant allergen sensitization at one year and at three years of age by specific IgE measurement.
6. The prevalence of the atopic wheeze phenotype at three years of age.

7.2.5 Composite allergy outcome

1. The prevalence of combined allergic disease (a composite of cumulative IgE mediated food allergy to all foods, atopic wheeze phenotype, eczema and allergic rhinitis) at three years of age.
2. The prevalence of combined allergic disease (a composite of cumulative IgE and non-IgE mediated food allergy to all foods, atopic wheeze phenotype, eczema and allergic rhinitis) at three years of age.

7.3 Safety outcomes

7.3.1.1 Adverse events – gastrointestinal symptoms

For each of these, the number of events will be analysed together with any associated morbidities or treatment as appropriate

1. Diarrhoea
2. Constipation
3. Posseting
4. Vomiting
5. Colic

7.3.1.2 Adverse events – atopic symptoms

1. Eczema
2. Wheeze – number of episodes, associated shortness of breath, A&E attendances, admissions

7.3.1.3 Adverse events – infective symptoms

1. URTI
2. LRTI
3. Bronchiolitis

4. Other infections

7.3.2 Serious adverse events

1. Hospital admissions
2. All serious adverse events

7.3.3 Anthropometric outcomes

1. Weight, absolute and SDS at 3, 12 months and three years
2. Length, absolute and SDS, at 3, 12 months and three years
3. BMI, absolute and SDS , at 3, 12 months and three years
4. Head circumference, absolute and SDS, at 3, 12 months and three years
5. Mid upper arm circumference at 3, 12 months and three years
6. Skin fold thickness, subscapular and triceps, at 3, 12 months and three years

7.3.4 Behavioural outcomes

1. Sleep outcomes (duration, time to settle, number of wakings)
2. Food aversions outcomes

7.3.5 Maternal outcomes

1. Maternal quality of life at 3, 12 months and three years

7.3.6 Dietary outcomes

1. Macro nutrient status at 6, 12 months and three years

7.3.7 Breastfeeding outcomes

1. Percentage of mothers breastfeeding at 6 months
2. Percentage of SIG mothers exclusively breastfeeding to around six months
3. Total duration of breastfeeding

7. STATISTICAL ANALYSIS

8.1.1 Intention to Treat Analysis

These will be the main analyses and the ones that are used to quantify the efficacy of the intervention. Only ITT results will be reported in the abstracts of the main publication and trial report.

All children allocated to each of the two study arms will be analysed together as representing that treatment arm, whether or not they received or completed the prescribed regimen. This will be done after the three year assessment for the period prevalence of IgE mediated food allergy to the six intervention foods between one and three years of age in both arms.

8.1.2 Per Protocol Analysis

These will be reported in the body of the paper after the ITT primary and ITT secondary analyses. They will be included for explanatory purposes only, and will not be reported in the abstract nor regarded as primary or secondary measures of the efficacy of the intervention.

<u>Assessing the 'dose' of potentially allergenic food received</u>

The following algorithm defines a scoring system that quantifies dose in a relatively simple way and yet allows the detection of a dose-response, albeit outside of the original randomization. Hence for each participant regardless of group, their score will be defined as follows:

- Each week up to 6 months of age (26 weeks) that a participant ate a particular food at 75% or more would be scored 1 and lesser consumption would be a zero
- Each food would therefore yield a total score of between 0 and a maximum of 13 (as max weeks=13)
- The scores will be summed over all foods to give a total food consumption score which will be categorized into quintiles
- The relationship between score quintiles and the primary outcome will be explored using logistic regression with a test for trend across the quintiles

8.1.3 Statistical Methodology

Binary outcomes will be presented as proportions and relative risks with 95% confidence intervals, p values being calculated from the chi-square test or Fisher's exact test if numbers are small, with appropriate methods used for the calculation of CIs when numbers are small.

Continuous outcomes will be analysed using methods based on the Normal distribution where possible either if data are Normal or can be suitably transformed. Results will be presented as differences in means with 95% confidence intervals and modelled using multiple regression with baseline values as covariates.

Adverse events will be reported by treatment group, aggregated and given as number of children, total number of events, and incidence rates per year with 95% confidence intervals

Prognostic baseline variables will be inspected for any imbalance between the groups that is judged to be clinically important. If any such imbalance is observed, these variables will be included as covariates in sensitivity analyses for all key outcomes (please see section 8.2 below).

All analyses will be 2-sided with 5% significance level.

The sections below will outline the analyses for the main paper of the trial and describe what material will be reported in each table.

8.2 Description of baseline data: TABLE 1

Key variables collected at baseline will be summarised by treatment group. For continuous variables; means and standard deviations will be calculated and for categorical data, actual numbers and the calculated proportions will be shown. No formal testing of baseline characteristics between treatment groups will be performed in keeping with best practice.

1. Sex of infant
2. Birthweight
3. Gestational age
4. Number of siblings
5. Age of mother
6. Maternal education
7. Maternal and paternal asthma/allergy
8. Maternal/paternal smoking
9. Ethnic origin of mother and father
10. Pet ownership status
11. Mode of delivery
12. Allergenic food consumption during pregnancy
13. Allergenic food consumption during lactation pre enrolment
14. Infant eczema status at enrolment

8.3 ANALYSIS OF ENDPOINTS ITT

8.3.1 Analysis of Primary Endpoint TABLE 2

The main analysis will compare the period prevalence of IgE mediated food allergy to the six intervention foods between one and three years of age in both arms (protocol, Section 3.3.1). Table 1 will also include allergy to each of the six constituent foods by treatment group. The outcomes to be analysed by intervention group, are listed below:

1. Period prevalence of IgE mediated food allergy to one or more of the six intervention foods between one and three years

2. Period prevalence of IgE mediated food allergy to each of the six intervention foods between one and three years:

    i. Cow's milk
    ii. Egg
    iii. Fish

iv. Wheat

v. Peanut

vi. sesame

8.3.2 Analysis of Secondary Endpoints by intervention group: TABLES 3-5
The following secondary outcomes will be reported in these tables:

TABLE 3: Period and cumulative prevalence (one to three years of age) food outcomes as listed below:

1. The period prevalence of all IgE mediated food allergy between one and three years of age in both arms.
2. The period prevalence of all reported food allergy (IgE and non-IgE mediated) between one and three years of age in both arms.
3. The period prevalence of sensitization to food between one and three years of age in both arms (defined in Protocol, Section 3.9).
4. The total number of foods (of the six intervention foods) to which IgE mediated food allergy has been diagnosed between one and three years of age in both arms amongst children with IgE mediated allergy to one or more of the six intervention foods.
5. The total number of foods (any foods) to which IgE mediated food allergy has been diagnosed between one and three years of age in both arms amongst children with IgE mediated allergy to one or more foods.
6. The cumulative prevalence of IgE mediated food allergy to the six intervention foods by three years of age.
7. The cumulative prevalence of all IgE mediated food allergy by three years of age.
8. The cumulative prevalence of all food allergy (IgE and non-IgE mediated) by three years of age.
9. The cumulative prevalence of non-IgE mediated food allergy by three years of age.
10. The cumulative prevalence of sensitization (defined in Section 3.9) to the six foods by three years of age.
11. The total number of foods (of the six intervention foods) to which IgE mediated food allergy has been diagnosed by three years of age amongst children with IgE mediated allergy to one or more of the six intervention foods.
12. The total number of foods (any foods) to which IgE mediated food allergy has been diagnosed by three years of age amongst children with IgE mediated allergy to one or more foods.

TABLE 4: Other allergic diseases and composite allergy outcome by intervention group as listed below:

1. The point prevalence of eczema at one year and three years of age and cumulative prevalence of eczema by three years of age.
2. The severity of eczema at one year and three years of age.
3. The prevalence of allergic rhinitis at three years of age.
4. The prevalence of inhalant allergen sensitization at one year and at three years of age by skin prick test.
5. The prevalence of inhalant allergen sensitization at one year and at three years of age by specific IgE measurement.
6. The prevalence of the atopic wheeze phenotype at three years of age.
7. The prevalence of combined allergic disease (a composite of cumulative IgE mediated food allergy to all foods, atopic wheeze phenotype, eczema and allergic rhinitis) at three years of age.
8. The prevalence of combined allergic disease (a composite of cumulative IgE and non-IgE mediated food allergy to all foods, atopic wheeze phenotype, eczema and allergic rhinitis) at three years of age.

TABLE 5: Safety outcomes and indicative outcomes as listed below by intervention group:

1. Diarrhoea

2. LRTI

3. Constipation

4. Eczema
5. Wheeze
6. URTI
7. Bronchiolitis
8. Other infections
9. Hospital admissions
10. Absolute weight at 3, 12 months and three years
11. Weight standard deviation score at 3, 12 months and three years

8.4 MISSING DATA

Patterns of missing data will be investigated, in particular looking at whether missingness can be considered to be missing completely at random, missing at random, or missing not at random. Sensitivity analyses assuming different missingness patterns will be undertaken – following the general approach of White 2011 (BMJ. 2011; 342: d40; doi: 10.1136/bmj.d40).

8.5 STATISTICAL PROGRAMS

Analysis will be conducted using Stata (version current at the time of analysis).
