# Supplementary material for: Association of Early Introduction of Solids With Infant Sleep: A Secondary Analysis of a Randomized Clinical Trial
Source: JAMA Pediatr. 2018 Jul 9;172(8):e180739. doi: 10.1001/jamapediatrics.2018.0739 (PMC6142923; doi:10.1001/jamapediatrics.2018.0739)
Supplement: Supplement 7. — Trial Protocol. Summary of changes to SAP. [file jamapediatr-172-e180739-s007.pdf]

The EAT Study SAP - Summary of changes

The original statistical analysis plan was completed by Salma Ayis, Michael Perkin, Kirsty Logan, and Gideon Lack (edited by Janet Peacock) and approved on 20th August 2012 by the EAT study Trial Steering Committee. This version was reviewed by the IDMC and a final version produced on 4th April 2013 addressing their comments.

Summary of changes from original version (20/08/2012) to final version (04/04/2013)

8.2 - Slight variation to data collected at baseline with the addition of 'child age at visit'

8.3.1 - Slight variations to the statistical analysis plan to use sensitivity analysis to adjust for a possible window of ± 6 months of age when comparing the period prevalence of IgE mediated food allergy to the six intervention foods between one and three years of age in the two arms, using logistic regression

7.2.1.2 - Period prevalence of food outcomes was updated to 'challenge-proven food allergy' from 'all reported food allergy'

8.1.2 - Per-protocol analysis was changed to include a proposal for comparison of quintiles in mean dose score

9.0 - Addition of further analyses beyond the main results paper and analyses to be documented and approved by the steering committee
